# Identification of breast cancer recurrence risk factors based on functional pathways in tumor and normal tissues

**DOI:** 10.18632/oncotarget.11557

**Published:** 2016-08-23

**Authors:** Xiujie Chen, Lei Liu, Yunfeng Wang, Bo Liu, Diheng Zeng, Qing Jin, Mengjian Li, Denan Zhang, Qiuqi Liu, Hongbo Xie

**Affiliations:** ^1^ College of Bioinformatics Science and Technology, Harbin Medical University, Harbin, Heilongjiang, 150081, PR China

**Keywords:** breast cancer, normal tissue, tumor tissue, recurrence, risk factors

## Abstract

The recurrence of breast cancer (BC) is a serious therapeutic problem, and the risk factors for recurrence urgently need to be identified. In this study, we examined the functional pathways in tumor and normal tissues to more comprehensively identify biomarkers for the risk of BC recurrence. We collected tumor and normal tissue gene expression profiles of recurrent BC patients and non-recurrent BC patients from the TCGA database.We derived an expression interval (mean ± 1.96SD) based on non-recurrent patients rather than a single value, such as a mean or median. If the expression of a gene was significantly different from its normal expression interval, it was considered a differentially expressed gene. Eight pathways that significantly distinguished recurrent and non-recurrent BC patients were obtained based on 65% accuracy, and these pathways were all associated with the immune response and sensitivity to drugs. The genes in these eight pathways were also used to analyze survival, and the significance level reached 0.003 in an independent dataset (*p* = 0.02 in tumor and *p* = 0.03 in normal tissue). Our results reveal that the integration of tumor and normal tissue functional analyses can comprehensively enhance the understanding of BC prognosis.

## INTRODUCTION

Breast cancer (BC) is a highly heterogeneous disease with different clinical manifestations. Although some patients can benefit from adjuvant systemic therapies, a substantial proportion of BC patients have poor therapeutic outcomes [1]. After chemotherapy, many patients develop adverse consequences of heavy metal poisoning, which decrease the success rate of therapy [2], and how to identify patients suitable for chemotherapy remains a challenge in the field. Many clinical and pathological factors are currently employed to predict the prognosis of patients, such as the Gallen guidelines [3], the National Institutes of Health guidelines [4], the Nottingham Prognostic Index guidelines [5] and the computer program Adjuvant! [6]. However, most of these prognosis factors rely on changes in the status of the patient′s tumor, such as changes in the tumor diameter, axillary lymph node status, histologic grade, estrogen receptor (ER) status and progesterone receptor (PR) status [7]. Several other cancer studies have found that precancerous tissue dysfunctions are associated with cancer prognosis, and these abnormal functions are often related to protective antitumor immunity [8]. In addition, the early identification of precancerous normal tissue damage may provide better prognosis biomarkers [9, 10]. To this end, Ye M and Herszényi L considered the difference and uniformity between the precancerous and cancer statuses. Activated leukocyte cell adhesion molecule (ALCAM) has been identified as a novel potential molecular marker of gastric cancer. Ye M analyzed the serum soluble ALCAM (sALCAM) in large numbers of patients with gastric cancer, patients with precancerous lesions, and controls. The expression of ALCAM mRNA in different disease status was significantly different [11]. Herszényi L found that proteolytic enzymes play a major role not only in colorectal cancer (CRC) invasion and metastasis but also in the malignant transformation of precancerous lesions into cancer [12]. This finding indicates that the precancerous and cancer statuses may share common mechanisms that may influence prognosis. these findings show that precancerous and cancer status have not only differences in expression level but also uniformity in predicting prognosis Therefore, only considering the characteristics of cancer and ignoring precancerous characteristics is insufficient to predict prognosis. However, the studies combining these two statuses at the functional level to systematically assess cancer prognosis are rare. Therefore, we herein utilized both tumor and normal tissue from recurrent BC patients and from non-recurrent BC patients, identificated differentially expressed genes of precancerous normal tissue and tumor tissue between two patient groups and the biological processes affected by these genes. Finally, the utility of these genes to predict prognosis was evaluated.

Because breast cancer is highly heterogeneous, even in the patients with the same phenotype (such as good or poor outcome), these tumors also significantly exist differences at the molecular level. Due to this sample heterogeneity, genes that are only specifically expressed in small number groups are often not identified using traditional statistical methods, such as the *t*-test, Mann–Whitney test or the Significance Analysis of Microarrays (SAM) approach [13]. To identify DEGs between groups and genes that are specifically expressed in small number groups, we propose a new algorithm that uses the range of the expression levels in non-recurrent BC patients as a reference instead of comparing the mean expression levels between groups. Randomization was used to identify specific genes that were only altered in subgroups and reflect individual recurrence risk.

Thus, we used the randomization method to identify genes that express significantly beyond the normal interval. We then identified pathways associated with recurrence risk in both normal and tumor tissues. In these pathways, patients with diverse prognosis present different functional level. We finally identified eight risk-associated pathways based on a transcriptional profile analysis that were efficiently able to predict BC patients with different prognosis outcomes. The risk-associated genes were differentially expressed genes in the risk-associated pathways. Calcium signaling, Gap junction, MAPK signaling and Jak-STAT signaling were obtained from tumor status, and explained the prognosis related functions in tumor therapeutic status.Tuberculosis, Salmonella infection, small cell lung cancer and influenza A were obtained in normal status study and showed the relationship between intrinsic genetic background and prognosis. Kaplan-Meier (K-M) survival curves indicated that patients without changes in risk-associated genes survived significantly longer than patients exhibiting changes. These findings demonstrate that the eight risk-associated pathways and genes can efficiently distinguish patients with good prognosis and patients with poor prognosis to predict recurrence risk. Our results can be used to objectively evaluate the need for further chemotherapy.

## RESULTS

### Tumor tissue study

### Differentially expressed gene analysis

For each gene we calculated the value sum beyond normal interval based on good group, and obtained its probability distribution. The number of genes with a *p* value less than 0.05 is 3852 genes. After FDR correction, we obtained 3254 genes, including 1923 up regulated genes and 1331 down regulated genes. (Supplementary Table S1).

### Comparing with the traditional method

We used the R package of the Limma method to analyze the expression profile of tumor tissue and then compared this profile with our results. Specifically, the Limma algorithm returned 2684 differentially expressed genes, 2097 of which were also identified using our method. The normal tissue profiles were also analyzed, and the Limma algorithm identified 4565 differentially expressed genes, 3875 of which were also identified by our method.

**Figure 1:**
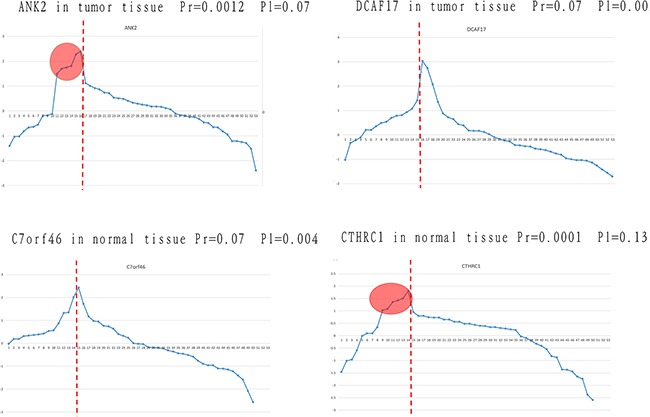
Identification of differentially expressed genes using our approach and traditional methods The four genes that were not consistently identified using the traditional method and our method are listed. The horizontal axis represents samples, and the vertical axis represents gene expression. Samples on the left of the red line are from the poor prognosis group, and those on the right are from the good prognosis group. Pr and Pl represent the *p* value obtained by randomization and the Limma algorithm, respectively.

However, we found that some significant differential expressed genes identified by Limma, i.e., DCAF17 and C7orf46, did not remain differentially expressed after random perturbation, as shown in Figure 1. These genes were considered as significantly expressed due to the mean difference between the two groups (recurrent and non-recurrent) of data. However, according to the expression levels distribution, we found that these genes still fluctuate in the normal range, even if they have a different mean value with the control group (non-recurrent).

ANK2 and DCAF17 were extracted in tumor tissue of patients with different prognosis. After a randomization process, ANK2 was identified as differentially expressed (*P* = 0.0012), but this gene was not identified by the Limma algorithm (*P* = 0.07). Viewing from the expression value distribution, in spite of similar meanvalues between two groups, some samples in the poor prognosis group showed significantly higher expression level than the normal range, which shows that ANK2 may be involved in personalized relapse mechanism. For gene DCAF17, it was considered to be significant differentially expressed genes (*P* = 0.002) by the Limma algorithm, but was not significant after the process of randomization (*P* = 0.07). Although DCAF17 has different mean values between the two groups, it still fluctuate within the normal range. Similar results were obtained in normal tissue study, such as C7orf46 and CTHRC1. In conclusion, for some specific genes that are differentially expressed in small groups, traditional methods cannot identify them although there are differences between the groups at the mean level. Moreover, genes exhibiting a significant difference in their mean levels between groups but still remaining within the normal range were not supposed to be risky genes as well.

### The hierarchical clustering analysis

To verify that the extracted DEGs can effectively differentiate between good and poor outcomes and whether groups of the same outcome can be further divided into subgroups, we applied the unsupervised hierarchical clustering analysis classification method.

**Figure 2:**
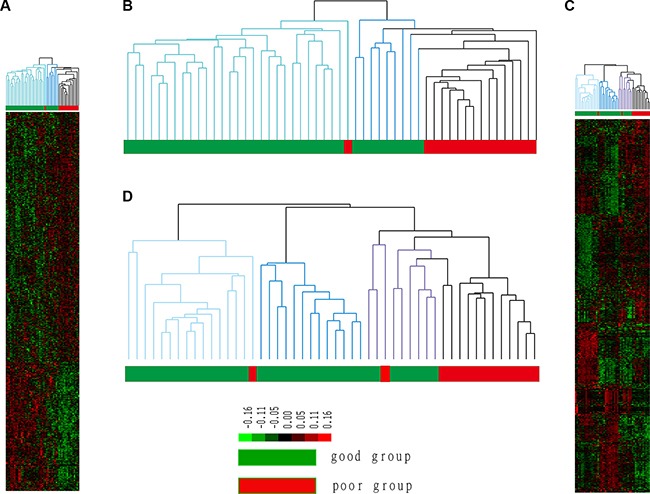
Cluster results for tumor and normal tissue samples The good prognosis group indicates patients who did not experience recurrence, whereas patients in the poor prognosis group experienced recurrence. Groups 1 and 2 represent two subgroups of the good prognosis group in B; group 1 is marked in light blue, and group 2 is marked in dark blue. Groups 1, 2 and 3 represent three subgroups of the good prognosis group in D, and group 3 is marked in purple.

We used all DEGs in the clustering analysis of 53 BC tumor tissue samples, the results of which are shown in Figure 2.

Figure 2A, 2B shows that 14 of 15 recurrent patients (poor prognosis) clustered in the same cluster. Namely, 93% of patients who experienced recurrence clustered together and significantly differed from patients who did not experience recurrence. This result showed that the obtained DEGs can predict prognosis in patients with BC. Notably, non-recurrent patients were also divided into two different subgroups. Group 1 contained 28 samples that exhibited the most significant difference from recurrent samples, which indicated the least risk of recurrence; Group 2 contained 9 samples that were most similar to recurrent samples, indicating a higher risk of recurrence. Thus, patients in Group 2 were identified to be at risk for recurrence.

### Identification of risk-associated pathways

The hierarchical clustering analysis results show that the extracted DEGs can efficiently distinguish recurrent BC patients from BC patients. These genes may be involved in important biological functions that affect therapeutic outcomes. However, even patients with the same clinical phenotype exhibit disease heterogeneity. Moreover, the clustering analysis also identified different subgroups in the same group. These findings all indicate that functional level differences exist not only in patients with different prognoses but also in patients whose clinical signs are similar. To further analyze functional differences between patients whose risk of recurrence differs, we calculated the deviation score of 278 KEGG pathways using DEGs. Patients were classified according to the deviation scores based on up- or down-regulated DEGs (Supplementary Table S2). Finally, we selected four pathways with precision values greater than 65% as risk-associated pathways. These 4 pathways are listed in Table 1.

**Table 1:** Risk-associated pathways identified in tumor tissue

pathway	precise1	precise2	fuzzy
hsa04020 calcium signaling pathway	0.79	0.8	10
hsa04540 Gap junction	0.79	0.73	9
hsa04010 MAPK signaling pathway	0.74	0.73	9
hsa04630 Jak-STAT signaling pathway	0.74	0.8	11

Precise1 and 2 are the precisions of the good and the poor prognosis groups, respectively. Fuzzy represents intersected or isolated patients.

**Figure 3:**
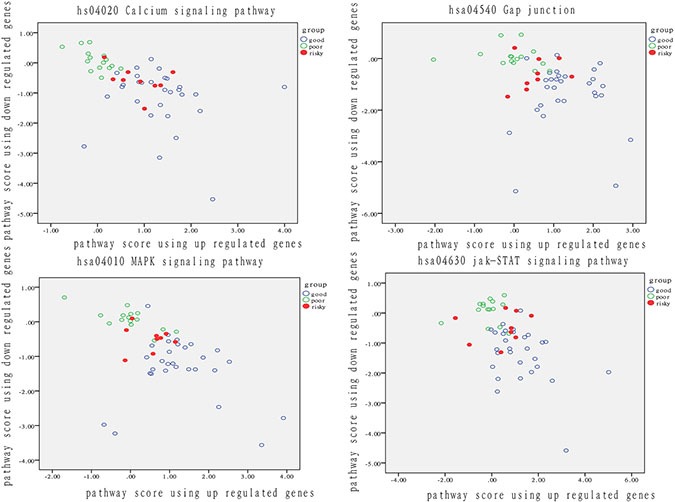
Analysis of patient distribution based on risk-associated pathways in tumor tissue The horizontal axis represents the pathway deviation score based on up-regulated genes; the vertical axis represents the pathway deviation score based on down-regulated genes. The good prognosis group marked in blue represents patients who experienced a good outcome; the poor prognosis group marked in green represents patients who experienced a poor outcome. At-risk patients are marked in red.

Because we also identified a small group of patients at risk for recurrence in the good prognosis group, we analyzed the relationship of these patients with those who had a different outcome. We show the performance of each pathway in Figure 3.

The activity of these four risk-associated pathways significantly varied in both patients with a good prognosis and with a poor prognosis. Therefore, the four pathways can effectively identify the recurrent risk of BC recurrence, with a prediction accuracy exceeding 65%. Notably, nearly all patients at risk for recurrence were located in the middle of good and poor group patients. Even if at-risk patients do not experience recurrence, they resembled patients with a poor outcome at a functional level. When dysfunction of these risky pathway occurs, disease progression may happen on these risky patients. Except for the isolated fuzzy patients, the ratio of risky patients overlapped with intersected fuzzy samples was from 33% to 56%, which highlighted the use of functional pathway in distinguishing patients with diverse degree of recurrent risk.

To further identify the relationship of risk-associated pathways in normal status or tumor status, we searched for shared biological functions and activities. The function of inhibiting of apoptosis can be found in all of the four normal tissue related risky pathways. This finding indicated that apoptosis tends to be more inhibited in the nornal tissue of patients with poor outcomes than in other patients. The inhibition of apoptosis in normal cells leads to dysfunction due to aging and the accumulation of mutations, and the inhibition of tumor cell apoptosis results in aberrant cancer cell proliferation. Moreover, the four tumor status-related, risk-associated pathways also shared multiple biological functions, including apoptosis, proliferation, and the cell cycle. If the dysfunction in normal tissue represents the beginning of a loss of control, then blocking the cell cycle and activating proliferation may represent the associated process and consequence.

### Construction of a classifier model that integrates risk-associated pathways

**Figure 4:**
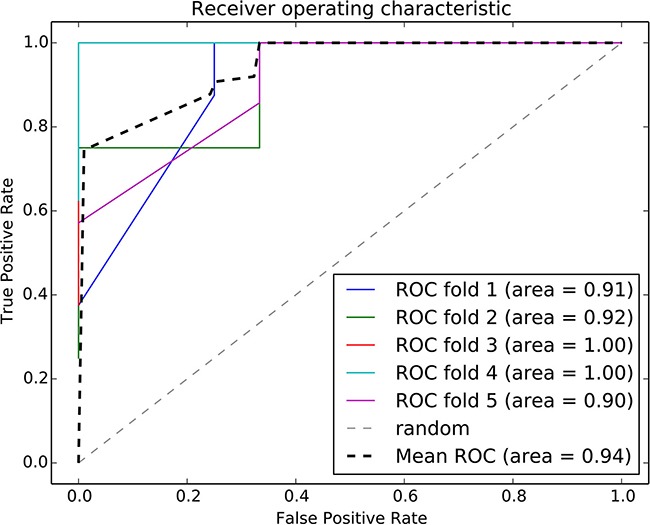
ROC curve of the random forest classifier using risk-associated pathways from tumor tissue Five-fold cross validation was used to evaluate the model performance. Each iteration is marked in a different color, and the average ROC curve is marked in black.

**Figure 5:**
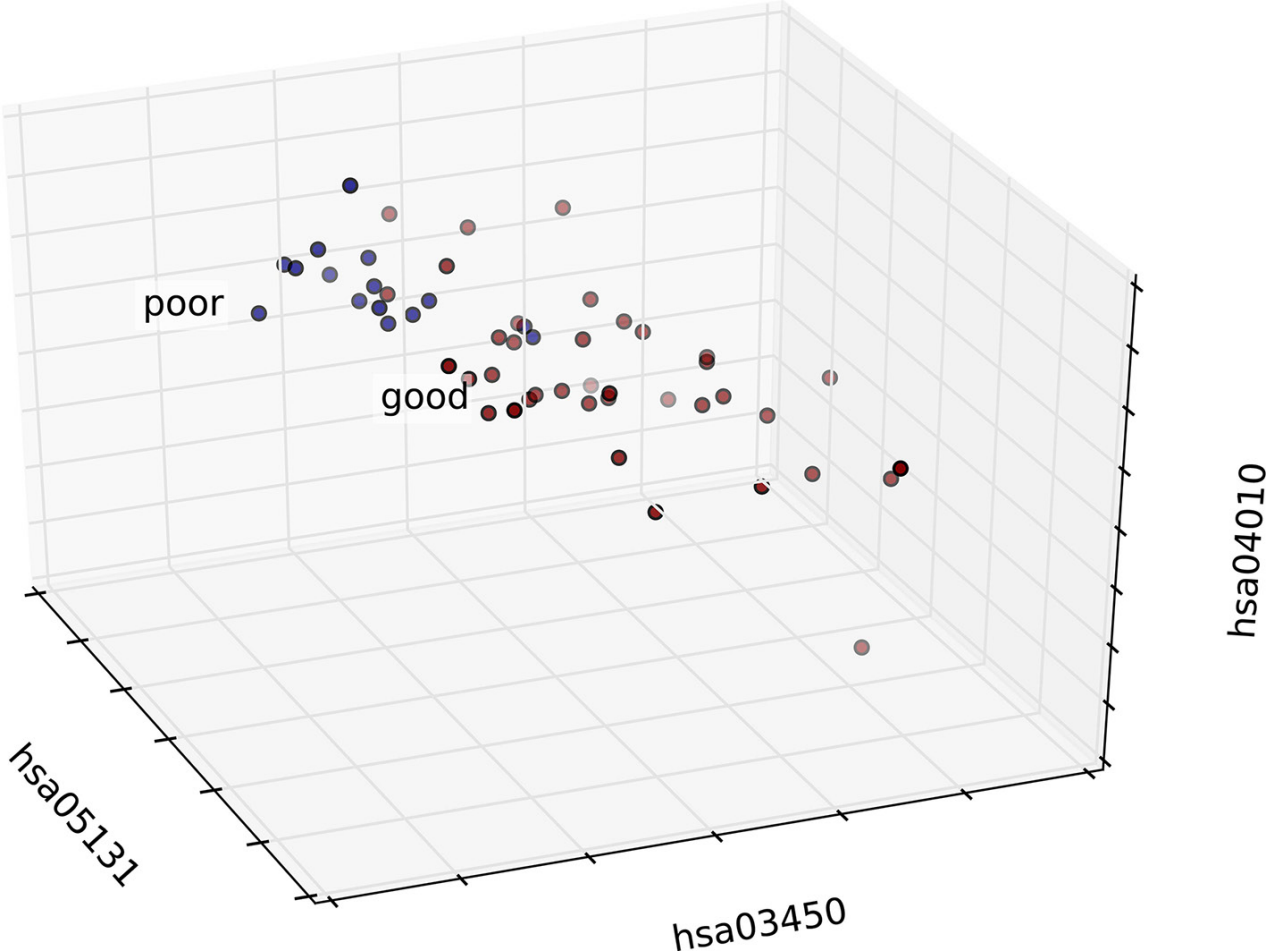
3D graph of patient distribution based on risk-associated pathways in the tumor Patients in the good and poor prognosis groups are marked in red and blue, respectively. The top three features were selected as axes, and each patient was mapped according to the values of these three features.

We integrated the risk-associated pathways derived from the tumor tissue analysis as features, and the random forest classifier reached an accuracy of 94%, as shown in Figure 4. This result demonstrated the effectiveness of the risk-associated pathways in identifying recurrence risk and generated an improved model compared with the use of a single risk-associated pathway. To intuitionally observe differences among patients at the pathway level, we utilized a 3D coordinate system to visualize the patient distribution, as shown in Figure 5.

### Survival analysis based on risk-associated genes in an independent breast cancer dataset

**Figure 6:**
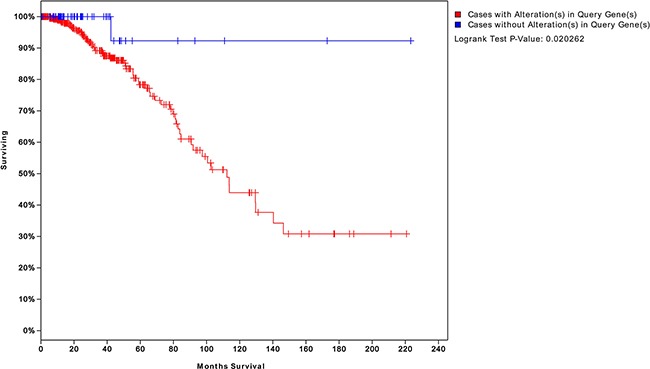
Survival analysis of patients with BC expressing genes risk-associated pathways in the tumor The blue curve represents the survival time of patients with BC not exhibiting changes in the 25 risk-associated genes. The red curve represents survival time of patients exhibiting changes in at least one of these genes. A *P* value of 0.02 indicates that a gene may serve as a biomarker to assess the risk of recurrence and survival time.

We extracted a total of 60 risk-associated DEGs from the four risk-associated pathways as risky genes and compared the survival of patients exhibiting expression level changes in at least one of these genes to that of patients not exhibiting any changes in these levels. Thirty-five genes were removed because they were not related with survival. The results of the survival analysis based on the remaining 25 genes are shown in Figure 6.

### Normal tissue study

### Analysis of differentially expressed genes

To further investigate whether the physiological differences at the functional level in precancerous tissue can affect therapeutic outcome, we also analyzed the transcriptional profiles of normal tissue in patients with good and poor prognoses. After FDR correction, we obtained 3134 genes, of which 1336 were up-regulated and 1798 were down-regulated genes (Supplementary Table S3).

### The hierarchical clustering analysis

The hierarchical clustering result using all DEGs is shown in Figure 2C, 2D. Specifically, 12 of 16 patients who experienced recurrence were assigned to the same cluster, i.e., 75% of patients with recurrent BC differ from patients whose disease did not recur at the physiological level. In addition, the good prognosis group can be further divided into three subgroups (subgroup 1 is marked in light blue, subgroup 2 is marked in dark blue, and subgroup 3 is marked in purple). Subgroup1 contains 16 samples (15 from the good prognosis group and 1 from the poor prognosis group), subgroup 2 contains 18 samples (18 from the good prognosis group), and subgroup 3 contains 11 samples (8 from the good prognosis group and 3 from the poor prognosis group). Patients in subgroups 1 and 2 exhibited more apparent differences compared with the poor prognosis group, whereas patients in subgroup 3 were most similar to patients from the poor prognosis group. Thus, patients in subgroup 3 are at a risk of recurrence even though they mostly experienced good outcomes.

### Identification of risk-associated pathways

**Figure 7:**
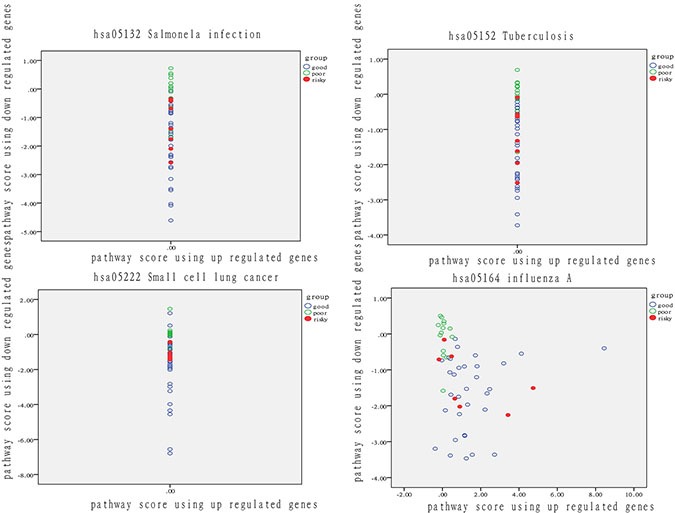
Analysis of patient distribution in normal tissue based on risk-associated pathways The horizontal axis represents the pathway deviation score based on up-regulated genes; the vertical axis represents the pathway deviation score based on down-regulated genes. The good prognosis group marked in blue represents patients with a good outcome; the poor prognosis group marked in green represents patients with a poor outcome. At-risk patients are marked in red.

In nomor tissue, we also ranked the 278 pathways using DEGs according to their discrimination precision and we set 65% as threshold and got 4 pathways listed in Table 2 (Supplementary Table S4). The discrimination performance of each pathway is shown as in Figure 7.

**Table 2:** Risk-associated pathways identified in normal tissue

pathway	precise1	precise2	fuzzy
hsa05152 tuberculosis	0.74	0.75	8
hsa05132 Salmonella infection	0.67	0.69	13
hsa05222 small cell lung cancer	0.77	0.69	10
hsa05164 influenza A	0.79	0.62	11

Precise1 and 2 are the precision of the good and poor prognosis groups, respectively. Fuzzy represents intersected or isolated patients.

These four pathways can effectively predict outcome. At-risk patients are marked in red and were much more similar to patients with a poor outcome than other patients in the good-outcome group. The overlap rate between at risk and fuzzy patients ranged from 29% to 71%. These findings demonstrated that pathway activity also reflects significant differences in the normal tissues of patients. In other words, differences in normal tissue at the physiological level to some extent determine therapeutic outcome.

### Construction of a classifier model that integrates risk-associated pathways

**Figure 8:**
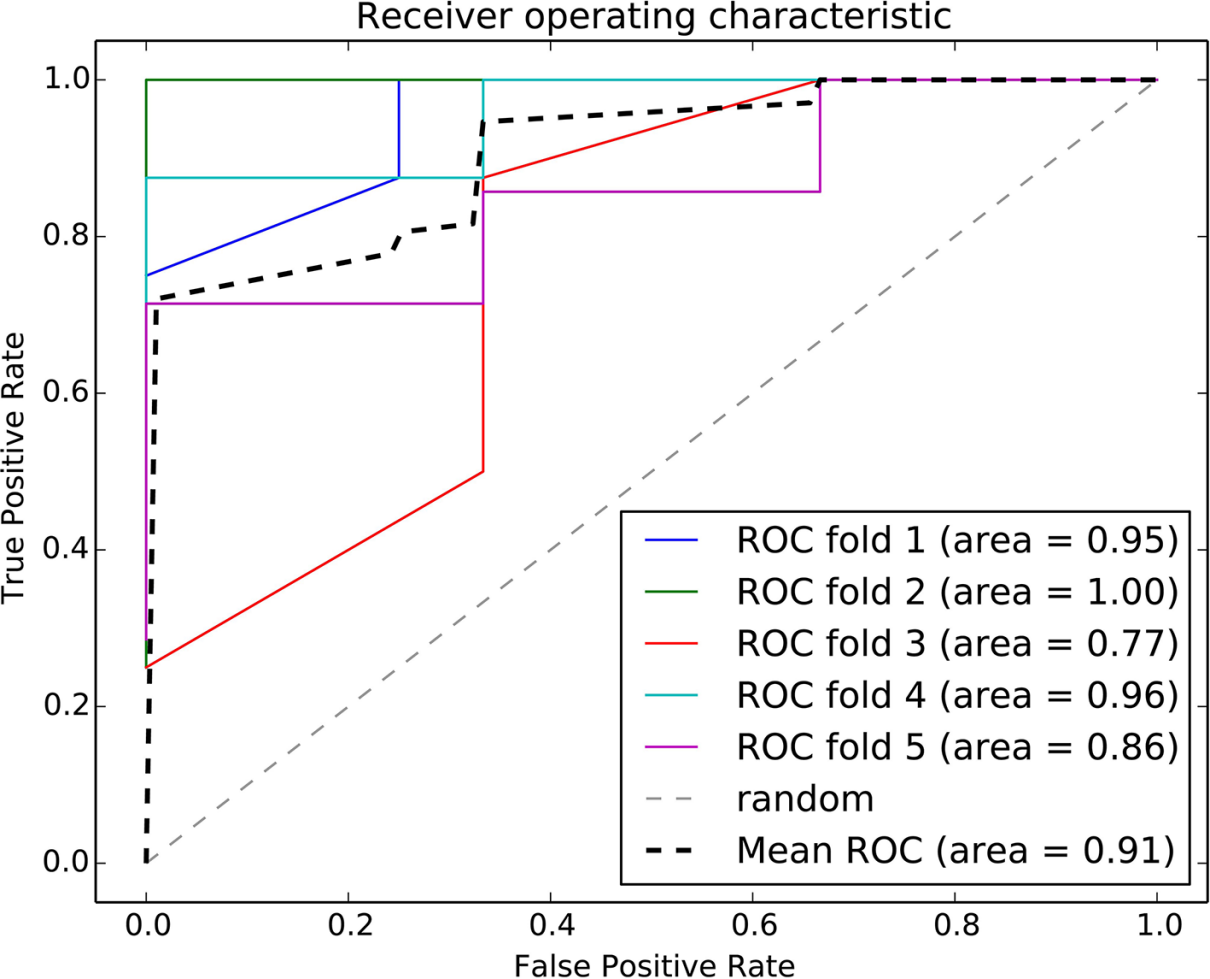
ROC curve of the random forest classifier using normal risk-associated pathways Five-fold cross validation was used to evaluate the model performance. Each iteration is marked in a different color, and the average ROC curve is marked in black.

**Figure 9:**
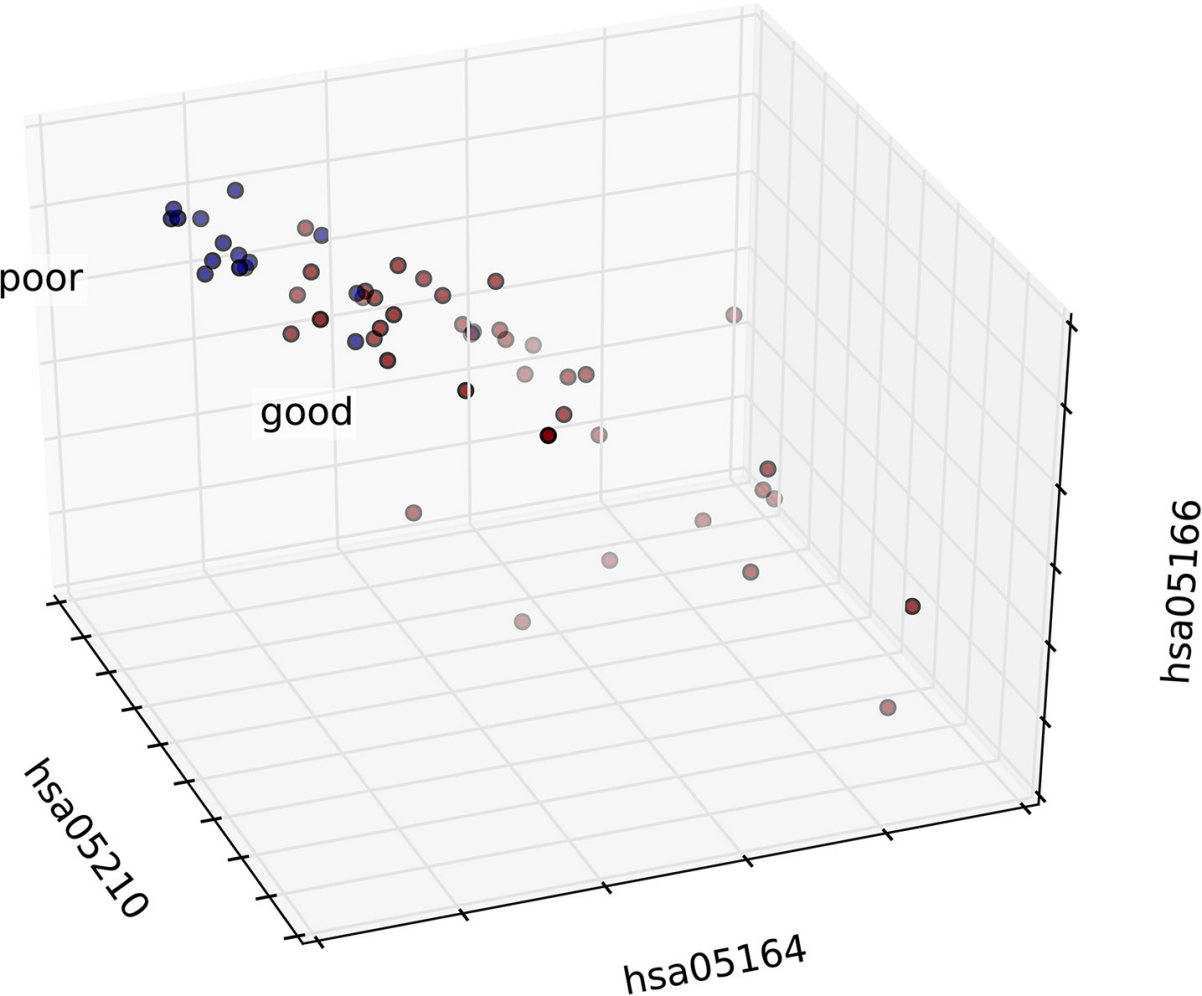
3D graph of the patient distribution based on risk-associated pathways in normal tissue Patients in the good and poor prognosis groups are marked in red and blue, respectively. The top three features were selected as axes, and each patient was mapped according to the values of these three features.

The risk-associated pathways derived from the normal tissue analysis were also used to construct a classifier model. An accuracy of 91% was achieved after a five-fold cross validation, as shown in Figure 8. The patient distribution based on the top three pathways is shown in Figure 9.

### Survival analysis based on risk-associated genes in an independent breast cancer dataset

We identified 12 DEGs in these four risk-associated pathways and compared the survival time of patients with BC who did and did not exhibit changes in the expression of these genes.

**Figure 10:**
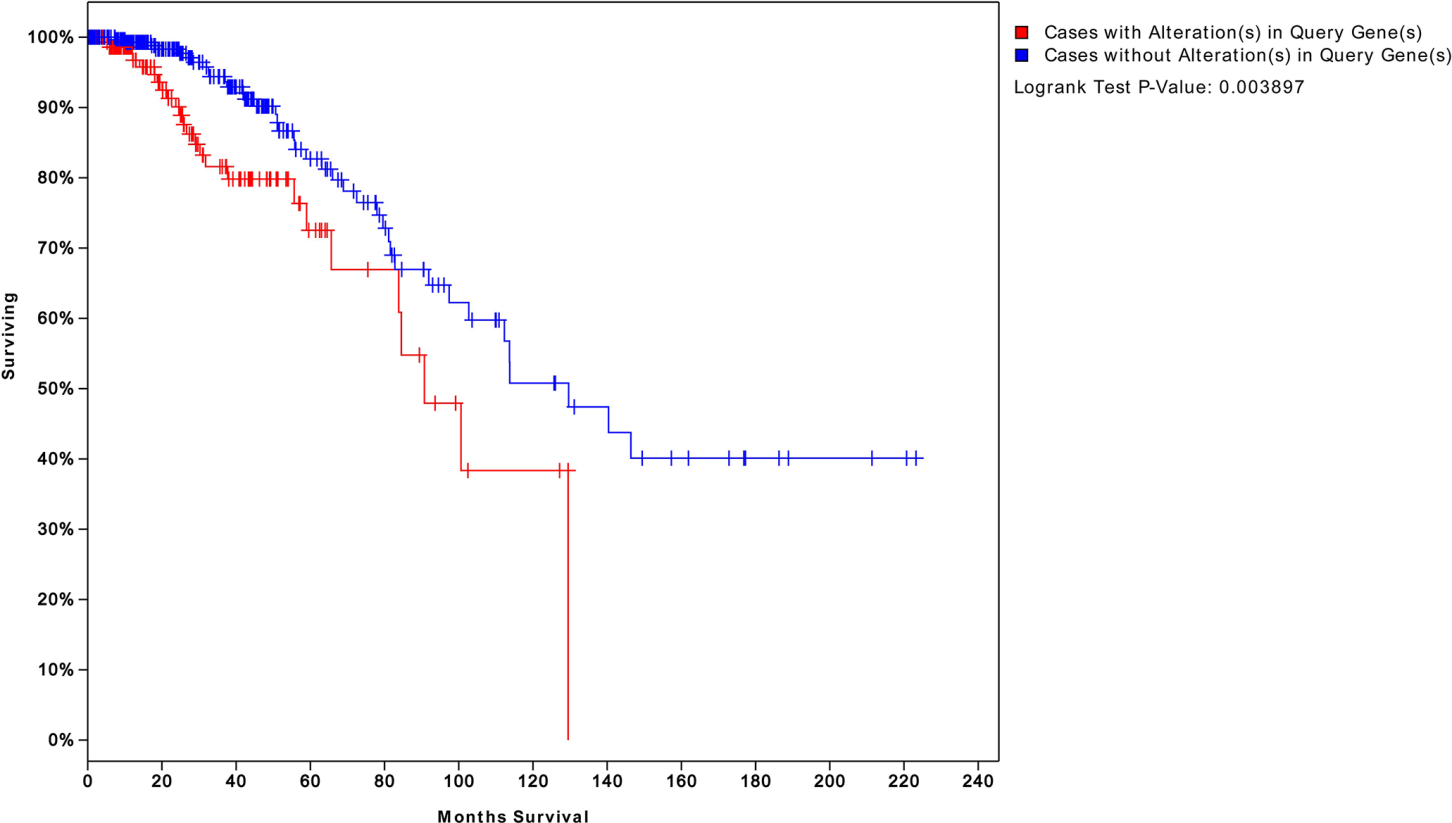
Survival analysis integrating both normal and tumor pathway genes We integrated the risk-associated genes from both normal and tumor tissues. Survival significantly differed between patients who did and did not exhibit changes in the expression levels of these genes (*P* = 0.003). This result suggests that integrating normal and tumor tissue analyses can better predict patient prognosis than an analysis of either status alone.

We obtained four risk-associated pathways from the analysis of tumor tissue. These pathways can effectively predict patients with different prognosis, which demonstrated that the dysregulation of these four pathways may affect biological processes that influence prognosis. Moreover, we analyzed normal tissue from patients and identified four new risk-associated pathways that had not been identified in our analysis of tumor tissue. We integrated the genes from both normal and tumor tissue analyses to assess survival, and the final significance reached 0.003, as shown in Figure 10. The survival analysis suggested a significant relationship between risk-associated pathways and prognosis. Therefore, combining analyses of normal and tumor tissue characteristics is of great significance for systematical and comprehensive cancer research.

### Classification of predictive models based on a cancer hallmark network framework

**Figure 11:**
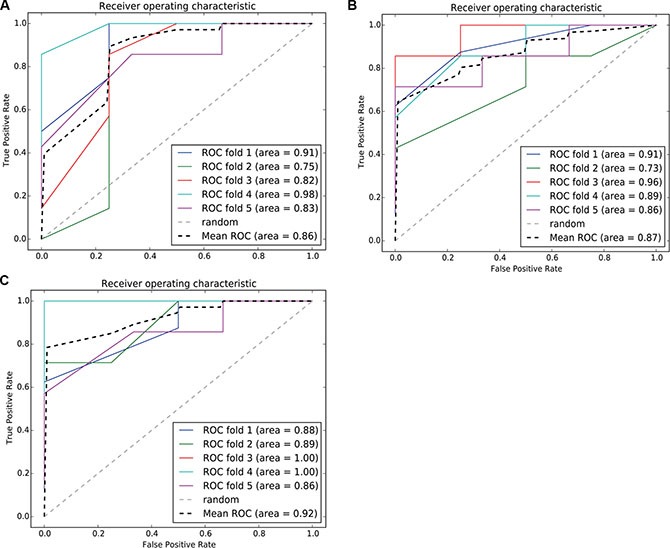
ROC curve of the SVM classification model using risk-associated genes in normal tissue, tumortissue and both tissue Each iteration is marked in a different color, and the average ROC curve is marked in black. (**11A**–**11C**) represents, respectively, the ROC curve of the SVM classification model using risk - associated genes from normal tissue, tomor tissue and integerated both tissues.

Hallmarks of cancer depict the logical framework for conceptualizing the variety of neoplastic diseases. Over the past decades, the analysis on diversity of cancers based on the framework constructed by hallmarks had greatly promoted the understanding of occurrence, development and metastasis of different cancers. To discuss a recent idea of cancer hallmark network framework which is taking about constructing mechanism-based predictive models in cancer. We integrated risk-associated genes identified in normal and tumor tissue analysis and enriched to ten hallmarks [19] to finally obtaining specific hallmarks. We used an SVM classification model with the five-fold cross validation method to predict BC patients with recurrent risk and validate the performance of classification. The results are shown in Figure 11. The classification performance was highest for the integrated hallmark risk gene model, with an AUC of 92%.

At *p* < 0.05, risk-associated genes expressed in normal tissue were significantly enriched in five hallmarks, that is Resisting Cell Death (RCA), Insensitivity to Antigrowth (IA), Limitless Replicative Potential (LRP), Self Sufficiency in Growth Signals (SSGS) and Sustained Angiogenesis (SA), whereas the significantly enriched hallmarks by risk-associated genes expressed in tumor tissue were IA and SSGS (Supplementary Table S5). Based on the enrichment results, we found that the normal tissue obtained the most fundamental trait of cancer cells, RCA, IA and SSGS, which represented the underlying molecular mechanisms to sustain chronic proliferation, resist to cell death, and resist inhibitory signals that might prevent their growth [20]. while sustaining Angiogenesis represents underlying molecular mechanisms for angiogenesis, which represents stimulating the growth of blood vessels to supply nutrients to tumors and evacuate metabolicwastes [20]. Namely, for the corresponding normal tissue of the patients with poor prognosis, it has lost its homeostasis due to loss the normal regulation to cell proliferation, differentiation and death under some tumorigenic factors, which made the normal tissue cells obtain the ability to survive without external environment stimulation, and angiogenesis ability for infinite differentiation cells to provide the necessary nutrients. That showes these hallmarks are related to preliminary stage of cancers [21, 22]. Therefore, “normal cells” exhibiting the above hallmarks will eventually develop into new cancer cells under certain conditions to result in recurrence even if the tumor was removed and the patient received adjuvant systemic therapy.

## DISCUSSION

Breast cancer is a highly heterogeneous cancer and treated with different therapy strategies based on its clinical immunohistochemical classification. However, as the number of patients experiencing resistance, recurrence and metastasis increases, increasing efforts have focused on the heterogeneity of breast cancer patients at the molecular level. In recent years, many studies have identified biomarkers related to the prognosis of breast cancer based on feature extraction, classifier, regression, and other statistical methods. However, most studies have relied on identifying common molecular features involved in the prognosis of patients at the pathological level and did not consider differences among breast cancer patients at the physiological level. We hypothesized that in addition to differences at pathological level in carcinoma tissue, physiological level differences in the normal tissue of patients may also contribute to the risk of recurrence or metastasis.

Considering the heterogeneity of breast cancer, we calculated the normal ranges of gene expression based on the levels measured in non-recurrent patients instead of comparing the mean expression levels between two groups. Using randomization, we identified specific genes that were only altered in subgroups and likely reflect individual recurrence risk by participating in the mechanisms of recurrence. Our method identified specific genes omitted by traditional methods. These genes may only be differentially expressed in small groups; thus, changes in these genes are usually less significant when compared to the mean values of background data. In addition, some genes whose mean expression levels differed between two groups according to traditional methods were also regarded as nonspecific genes because they lacked significance after randomization. Although the mean expression levels of these genes differed between two groups, these levels fluctuated within normal ranges. A hierarchical cluster analysis showed that the genes we identified can effectively predict prognosis. Moreover, even patients with the same prognosis phenotype can also be subdivided into different subgroups, which demonstrated that our method can efficiently identify different risk-associated subgroups and reflect personalized characteristics.

We used both the normal and tumor tissues of patients with diverse outcomes for analysis and identified eight risk-associated pathways, all of which are related to immune regulation and drug response. Our findings demonstrate a remarkable relationship between risk-associated pathways and the prognosis of patients with BC in terms of both recurrence risk and survival time. The risk-associated pathways extracted from tumor and normal tissues mediate various biological functions. Mahmood M found that combining a calcium-channel blocker with an alternative delivery method for the anti-cancer drug reduced cellular resistance to chemotherapeutics [23]. Gielen PR showed that increasing gap junction protein expression in human glioma cells enhanced resistance to TMZ treatment [24]. Moreover, a number of cancer-related biological processes, such as the apoptosis of leukemic cells [25] and oxidative stress resistance [26], are mediated by the MAPK signaling pathway, and the hyperactivation of this pathway can cause drug resistance [27]. Becerra-Díaz M highlighted the importance of the JAK-STAT signaling pathway in regulating drug susceptibility and resistance by studying mice deficient in STAT molecules [28]. The other four risk-associated pathways identified normal tissue are all involved in infection and inflammation, such as the intrinsic immune response [29–32]. Therefore, we speculate that both the intrinsic immune system in normal tissue and the drug response in tumor tissue can influence prognosis of patients with BC. Combining analyses of tumor and normal tissue can help to comprehensively identify cancer prognosis-related functions. Invaluable information may be missed when focusing on only on tumor or normal tissue. Specifically, survival analyses were much more significant when risk-associated genes from both normal and tumor tissue were integrated compared with either single gene set alone.

Intriguingly, the four pathways most significantly differed in normal tissue between the good and poor prognosis groups, and this difference was less significant in cancer tissues. Similarly, the four most significant pathways in cancer tissues were less significant normal tissue. This finding suggests that significant changes occur at both the physiological and pathological levels during malignant transformation. Certain genes or functions significantly differed in normal tissue based on patient outcomes, and these genes and functions were associated with the immune response, cell replication, drug sensitivity and other related functions. These genes and functions exhibit dysfunction during malignant transformation. Therefore, inherent differences at the physiological level are masked, and differences at pathological level are dominant. Thus, we speculate that significant differences in the genes and functions in normal tissue determine the inherent risk of cancer development, and differences in genes and functions in tumor tissue may explain the relationship between acquired dysfunction and recurrence. Combining the inherent and acquired risk factors for breast cancer recurrence may serve as a more comprehensive method for evaluating the prognosis of patients.

All four risk-associated pathways in normal tissue were involved in the inhibition apoptosis, which could be considered the first step of malignant transformation. Aging and the continuous accumulation of mutations result in the dysfunction of normal cells and even their transformation into tumor cells, which is followed blocking the cell cycle and activating proliferation in tumor tissue. Dysfunction in this second stage hinders the killing of cancer cells because of their increased proliferation, whereas normal cells are rendered fragile. This finding demonstrated the importance of identifying cancerous characteristics in normal tissue early. Specifically, the degree of apoptosis inhibition in normal tissue directly correlates with the risk of recurrence or drug resistance because normal cells are more fragile and easier to transform into tumor cells.

In addition to the risk of recurrence itself, we also evaluated prognosis based on the survival time to identify genetic biomarkers that can effectively assess recurrence and survival time. We used genes extracted from risk-associated pathways analyze survival in patients with breast cancer based on mRNA data from TCGA. A KM survival analysis showed that the genes extracted from risk-associated pathways in normal tissue and tumor tissue can effectively predict survival time. These findings all demonstrated that patients with differentially expressed risk-associated genes have a distinctly shorter survival times than patients without any alterations in these genes. Assessing the risk of recurrence can objectively identify the value of chemo- or radiotherapy to patients to ultimately provide a personalized rational therapeutic schedule, which consequently reduces the side effects of radiation and chemotherapy and improves the cure rate.

Classification based on risk-associated pathways also identified fuzzy samples that exhibited intersection or isolation. These patients failed to be classified into any group based on risk-associated pathways. The emergence of these fuzzy samples (isolation) was partly due to differences in personalized gene expression and bifurcation point data, which are usually difficult to distinguish. The other type of fuzzy sample (intersection) exhibited significant overlap with at-risk patients in the clustering analysis. Moreover, age, gender, and health background may also impact feature extraction and classification. Thus, our future work will integrate more patient data and prognosis-related features to provide a more comprehensive prediction method.

## MATERIALS AND METHODS

### Breast cancer patient data

We selected 53 breast cancer (BC) samples that had transcriptional information for both normal tissue and tumor tissue from the TCGA database. According to clinical data, the samples were divided into the good prognosis group without recurrence (38) and the poor prognosis group, in which disease recurred (15). An independent dataset containing mRNA data from 526 BC patients was used to analyze survival time [14]. The cBioPortal database was used to generate a K-M Survival curve using risk-associated genes [15].

### Differential expressed gene analysis

We standardized the expression profiles. To eliminate inherent variations in gene expression, we used the Z-score correction method [16]. We defined the good prognosis group as the control group, and the poor prognosis group as the case group.

A normal interval was calculated based on the distribution of expression values in the good prognosis group (means ± 1.96 standard deviations). Gene expression outside the normal interval may result in a poor outcome. For a gene G, the number of patients in the good prognosis group is n1, the number of patients in the poor prognosis group is n2, and an initial score for G is calculated using the following formula:

$$score\;=\;\sum_{i=1}^{n2}\left(X_{2i}\;-\;X'\right)$$

where

$$X'\;=\;\left\{ \begin{array}{l} X_{\max}\;if\;X_{2i}\;>\;X_{\max}\\ X{\;}_{2i}\;if\;X_{\min}\;<\;X_{2i}\;<\text{​ }X_{\max}\\ X_{\min}\;if\;X_{2i}\;<\;X_{\min}\; \end{array}\right.$$

X_2i_ is the expression value of the ith patient in the poor prognosis group, and X_max_ and X_min_ are two extreme values for the normal interval. Based on the sum of the expression of gene G outside the normal interval in poor prognosis group, the initial score of gene G can be calculated.

After initial score of G was obtained, all samples were randomly permutated, and samples with the number n1 were randomly allocated to the good prognosis group, whereas the remaining n2 samples were allocated to the poor prognosis group. A new random score was obtained using this formula, and the above process was repeated 10000 times. After permutation, the gene score background distribution can be translated into *P* values; genes with a *P* < 0.05 were considered DEGs.

### The hierarchical clustering analysis

To prove that the DEGs we identified not only predict prognosis but also characterize the individual characteristics of small subsets of patients, we used the DEGs in the poor prognosis group to conduct a hierarchical cluster analysis of all samples. Furthermore, the cluster performance was assessed according to the sample label (good or poor outcome) of samples in each cluster. Expression profile data were filtered and standardized. The filtering process selected genes that are expressed in at least 80% of the sample with standard deviations greater than 1. The normalization of both genes and the sample relied on the median standardization center method, and the similarity matrix was based on the correlation-centered method. The cluster 3.0 software was used for standardization and hierarchical clustering [17].

### Risk-associated pathway identification

We downloaded 278 pathways from the KEGG database and assigned a score for each pathway based on the DEGs. Abnormal pathway function is not only reflected in the gene expression but also in the loss of balance in a pathway [18]. To identify pathway dysfunction due to imbalance, we calculated the pathway deviation score using up- and down-regulated genes. For pathway P, the number of up-regulated genes was N1, the number of down-regulated genes was N2, and the score vector T = (U, D) can be obtained using formula 2 for sample S.

$$U\;=\;\frac{\sum_{i=1}^{N1}(x_{i}-\mu {)}^{3}}{N1}$$

$$D\;=\;\frac{\sum_{j-1}^{N2}(x_{j}-\mu {)}^{3}}{N2}$$

For a pathway P, *U* is the deviation score obtained using up-regulated genes, and *D* is the deviation score obtained using down-regulated genes. Xi is the gene expression value in sample S, μ is the mean value in the good prognosis group, and T is the score vector for sample S.

**Figure 12:**
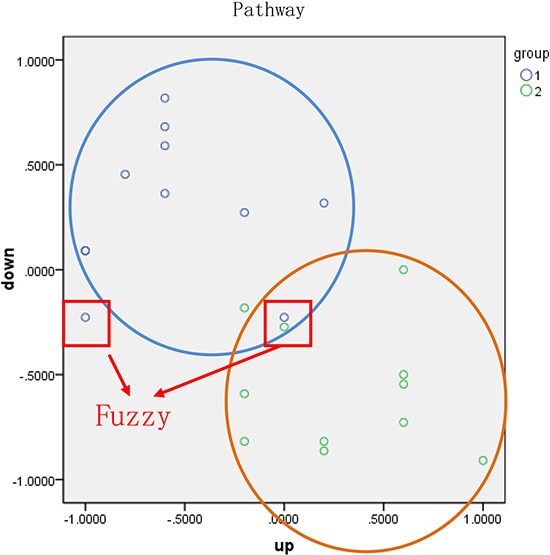
Classification of samples based on functional pathway Blue and green points represent two types of samples. The horizontal and vertical axes represent the pathway score calculated using up- and down-regulated genes, respectively. The blue and orange circles were constructed based on the geometric center and the radius, respectively, of each group.

Finally, we obtained a score vector matrix of 53278 entries. The identification of risk-associated pathways, which can effectively distinguish between two groups of samples, is beneficial for early diagnosis and predicting prognosis. We first found the geometric center of samples in the good and poor prognosis groups and then classified each sample according to its distance from each geometric center. In this study, the geometric center, O, was calculated using the score vector of samples in same group, and the radius, R, was then assigned. The circle centered on O with radius R had to include at least 80% of samples in the same group. Specifically, 80% was set as the threshold to consider the heterogeneity among samples and prevent outliers. We then calculated the classification accuracy according to the percentage of correctly classified/predicted samples, as shown in Figure 12.

The classification accuracy was evaluated based on the proportion of observed samples in all predicted samples. Samples circled in red were classified as fuzzy (fuzzy sample) and included two types of samples, intersection and isolation. To ensure the accuracy of the diagnosis, these fuzzy samples were not assigned to any group. The top four pathways with an average accuracy exceeding 65% were considered risk-associated pathways.

### Building a classifier model that integrates risk-associated pathways

We proposed an original method to evaluate the ability of each risk-associated pathway to predict outcome. We then integrated these risk-associated pathways to build an efficient classifier model using the random forest algorithm. Five-fold cross validation was used to evaluate the model efficiency, and the top three pathway features were used as axes in the 3D visualization analysis.

### Recognition of diagnosis biomarkers based on risk-associated pathways

We extracted DEGs from risk-associated pathways, which can act as biomarkers to effectively identify patients at a high risk of recurrence. However, only predicting the risk of recurrence is insufficient to evaluate therapeutic outcomes because the survival time is another important evaluation criterion. Thus, we compared the survival time of 526 patients with BC from the TCGA dataset who did and did not exhibit changes in risk-associated pathway genes. To optimize and screen for significant biomarkers, genes that did not influence the survival analysis *p* value were removed.

## SUPPLEMENTARY MATERIALS FIGURES AND TABLES












