# Supplementary material for: Identification of breast cancer recurrence risk factors based on functional pathways in tumor and normal tissues
Source: Oncotarget. 2016 Aug 23;8(13):20679–94. doi: 10.18632/oncotarget.11557 (PMC5400536; doi:10.18632/oncotarget.11557)
Supplement: Supplementary file 1 [file oncotarget-08-20679-s001.pdf]

Identification of breast cancer recurrence risk factors based on functional pathways in tumor and normal tissues

Supplementary Materials

Supplementary Table S1: 1923 up-regulated genes and 1331 down-regulated genes from timuor tissue of two group patients. See Supplementary_Table_S1

Supplementary Table S2: The deviation score of 278 KEGG pathways using DEGs from timuor tissue of two group patients. See Supplementary_Table_S2

Supplementary Table S3: 1336 up- regulated and 1798 down-regulated genes from normal tissue of two group patients. See Supplementary_Table_S3

Supplementary Table S4: The deviation score of 278 KEGG pathways using DEGs from normal tissue of two group patients. See Supplementary_Table_S4

Supplementary Table S5: Significantly enriched hallmarks by risk-associated genes expressed in tumor tissue,normal tissue and integrated both tissues. See Supplementary_Table_S5
