# Supplementary material for: Identification of breast cancer recurrence risk factors based on functional pathways in tumor and normal tissues
Source: Oncotarget. 2016 Aug 23;8(13):20679–94. doi: 10.18632/oncotarget.11557 (PMC5400536; doi:10.18632/oncotarget.11557)
Supplement: Supplementary file 2 [file oncotarget-08-20679-s002.docx]

Additional file 1:

| gene | score | | adj.P |
| --- | --- | --- | --- |
| ARHGEF10L | 4.611047 | 0.048849 | |
| RNF11 | -1.69253 | 0.012333 | |
| FGFR1OP2 | -2.87561 | 0.000912 | |
| ATRX | -3.75341 | 0.0001 | |
| ZNF708 | -8.41579 | 0.001735 | |
| SPR | 4.385681 | 0.000301 | |
| ZNF707 | 4.943423 | 0.011984 | |
| NUP98 | -9.59992 | 0.000912 | |
| SLC12A2 | -1.37689 | 0.01029 | |
| HAP1 | -0.56775 | 0.019563 | |
| OPA1 | -2.11267 | 0.026453 | |
| ARFRP1 | 3.802032 | 0.00773 | |
| SLC46A1 | -4.88993 | 0.046509 | |
| ATP2A2 | -1.07319 | 0.014752 | |
| ITGA2 | -8.42401 | 0.016254 | |
| ITGA4 | -2.87271 | 0.036323 | |
| DENND4C | -5.6986 | 0.008834 | |
| SWAP70 | -1.87851 | 0.006523 | |
| LOC100132287 | -3.17055 | 0.045554 | |
| PHLDA3 | 1.546393 | 0.014404 | |
| TACO1 | 6.864881 | 0.022892 | |
| PMM1 | 3.107051 | 0.006631 | |
| NENF | 6.961454 | 0.002776 | |
| MRPL28 | 3.15005 | 0.003831 | |
| FBXL12 | 6.16863 | 0.005326 | |
| CYP27B1 | 2.359035 | 0.004895 | |
| FBXL17 | -6.69362 | 0.027562 | |
| FBXL16 | 0.71499 | 0.019205 | |
| RALGAPA2 | -3.09076 | 0.032739 | |
| GHDC | 0.309245 | 0.020039 | |
| H19 | 2.010851 | 0.010625 | |
| THSD4 | -7.71682 | 0.034132 | |
| ITGAV | -7.52491 | 0.000809 | |
| NOV | -2.83037 | 0.001425 | |
| TRERF1 | -7.25887 | 0.033127 | |
| LRPAP1 | 3.49319 | 0.025339 | |
| AFTPH | -2.22849 | 0.041806 | |
| MUS81 | 0.797066 | 0.038658 | |
| AMMECR1L | -2.8376 | 0.003408 | |
| TMPRSS11F | 12.97966 | 0.046509 | |
| ZC3H15 | -0.21851 | 0.013364 | |
| C14orf118 | -7.00636 | 0.001116 | |
| ART1 | 17.77917 | 0.006742 | |
| COX4I1 | 7.016513 | 0.008945 | |
| ZC3H18 | 3.550387 | 0.028559 | |
| SIPA1 | 0.822296 | 0.030576 | |
| CCDC116 | -1.60461 | 0.010515 | |
| CADPS2 | -8.47054 | 0.046926 | |
| CCDC112 | -5.3561 | 0.006192 | |
| CCDC113 | -7.0934 | 0.032994 | |
| MGRN1 | 4.960614 | 0.018616 | |
| SPHK1 | 2.420608 | 0.028689 | |
| CPEB4 | -4.74152 | 0.018975 | |
| ZNF780A | -4.17212 | 0.037478 | |
| ZNF780B | -3.02103 | 0.031978 | |
| ZNF41 | -6.98519 | 0.049685 | |
| FBRS | 3.971044 | 0.045966 | |
| ZNF414 | 1.469974 | 0.046509 | |
| SP4 | -2.73439 | 0.007399 | |
| TMEM38B | -5.42082 | 0.001322 | |
| IGFBPL1 | -0.14686 | 0.042323 | |
| ATG4A | -1.77276 | 0.018141 | |
| THUMPD2 | -2.28115 | 0.032994 | |
| ATG4D | 3.399041 | 0.03109 | |
| MPHOSPH8 | -6.94004 | 0.003514 | |
| ANKFY1 | -5.10418 | 0.027825 | |
| MLLT4 | -3.79457 | 0.006962 | |
| TAF8 | -5.78856 | 0.007949 | |
| SIX4 | -6.60485 | 0.008172 | |
| LOC283070 | -0.64567 | 0.046926 | |
| SIX3 | 11.03198 | 0.031723 | |
| CNPY3 | 2.539461 | 0.016602 | |
| SYNJ1 | -1.39246 | 0.005004 | |
| C3orf75 | 0.635976 | 0.007069 | |
| MRO | 0.249321 | 0.040621 | |
| EFHA1 | -1.37762 | 0.031477 | |
| GPR75 | -4.11722 | 0.003725 | |
| RFX3 | -1.04612 | 0.0151 | |
| VPS37D | 2.623828 | 0.017082 | |
| APC | -7.14238 | 0.002671 | |
| TMEM179B | 0.715107 | 0.019088 | |
| UQCRC2 | 4.451202 | 0.001839 | |
| TIGD4 | -4.41711 | 0.009612 | |
| STXBP6 | -0.41924 | 0.04623 | |
| RUNDC2C | -4.5469 | 0.015562 | |
| TMEM127 | -7.27724 | 0.021449 | |
| C11orf2 | 0.67164 | 0.004788 | |
| RUNX2 | -6.15687 | 0.035295 | |
| BOP1 | 0.05979 | 0.009841 | |
| CKLF | 0.88758 | 0.027825 | |
| GJA3 | -1.52987 | 0.001631 | |
| EIF2B4 | 6.020342 | 0.035422 | |
| CSNK2A1P | -1.10964 | 0.015328 | |
| LOC389791 | 3.040538 | 0.023014 | |
| KIAA1267 | -2.50128 | 0.035422 | |
| CLIC4 | -1.49823 | 0.034918 | |
| TMEM147 | 8.930323 | 0.016254 | |
| TMEM141 | 2.058472 | 0.048979 | |
| API5 | -4.04078 | 0.004255 | |
| RTKN2 | -4.32995 | 0.01568 | |
| RNF180 | -2.8788 | 0.029443 | |
| KIAA1191 | -3.7212 | 0.039175 | |
| CA5B | -2.59837 | 0.007621 | |
| C19orf22 | 3.235758 | 0.009059 | |
| C19orf20 | 5.344985 | 0.000809 | |
| ZMAT4 | -1.05427 | 0.002151 | |
| C19orf24 | 4.416678 | 0.00288 | |
| C19orf25 | 0.319647 | 0.037615 | |
| C19orf28 | 4.168674 | 0.009727 | |
| C19orf29 | 1.199608 | 0.010964 | |
| ITGB1BP1 | 5.322651 | 0.047891 | |
| ZNF2 | -9.70377 | 0.022171 | |
| ZNF8 | -3.95803 | 0.000402 | |
| OGFR | 5.36141 | 0.000707 | |
| CDC5L | -3.96989 | 0.007178 | |
| AP2A2 | -4.59583 | 0.012101 | |
| FIGNL1 | -1.50697 | 0.03517 | |
| TMOD2 | -1.07458 | 0.049407 | |
| NSUN4 | -6.69263 | 0.010625 | |
| TUBG2 | -6.61976 | 0.023381 | |
| FAM108B1 | -2.51468 | 0.030701 | |
| ZXDA | -3.20316 | 0.027432 | |
| ERGIC3 | 10.96984 | 0.000809 | |
| PDF | 3.829874 | 0.032481 | |
| B3GAT3 | 1.679009 | 0.018975 | |
| CPPED1 | -8.63943 | 0.015792 | |
| CALM2 | -0.43436 | 0.03957 | |
| GSDMD | 0.993629 | 0.001219 | |
| HRH4 | -2.953 | 0.000707 | |
| HRH2 | -3.87529 | 0.00751 | |
| MBNL2 | -3.78557 | 0.00468 | |
| ZNF720 | -5.31218 | 0.001735 | |
| HRH1 | -2.73864 | 0.024223 | |
| CLCN7 | 7.174453 | 0.000707 | |
| BRD4 | 3.325474 | 0.028559 | |
| PTPN11 | -3.22467 | 0.021571 | |
| LOC100192379 | 12.7008 | 0.025963 | |
| PCDH20 | -0.4255 | 0.037076 | |
| C8orf30A | 7.331512 | 0.027056 | |
| MRPL27 | 9.423585 | 0.001839 | |
| C16orf72 | -1.37484 | 0.016964 | |
| C2orf82 | 16.72081 | 0.005004 | |
| DNAJB12 | 5.421899 | 0.029322 | |
| MSMB | 5.98437 | 0.004255 | |
| RNF138 | -5.11068 | 0.000707 | |
| ENOSF1 | 1.099902 | 0.045404 | |
| MCOLN1 | 1.641357 | 0.022656 | |
| ZNF587 | -3.40767 | 0.006302 | |
| LPAR6 | -5.93763 | 0.012101 | |
| LOC441046 | -2.58123 | 0.01452 | |
| MTRF1L | -5.84102 | 0.008504 | |
| MYO1D | -5.46694 | 0.005326 | |
| MYO1C | 0.59844 | 0.034918 | |
| GRN | 7.286452 | 0.000301 | |
| RPL36AL | 4.402357 | 0.034132 | |
| PPIAL4C | 2.840045 | 0.031723 | |
| FAM188A | -7.3565 | 0.001839 | |
| FAM160B1 | -6.44019 | 0.003198 | |
| GALNTL1 | -6.12584 | 0.015442 | |
| LSM11 | -4.11698 | 0.046777 | |
| SF3B1 | -1.74087 | 0.012676 | |
| LGALS3 | 1.40036 | 0.021807 | |
| AKIRIN1 | -6.17661 | 0.000301 | |
| EIF2AK2 | -1.16838 | 0.006631 | |
| TDG | -1.23402 | 0.021807 | |
| NMB | 5.325521 | 0.024103 | |
| OCRL | -4.39184 | 0.015562 | |
| XIST | -5.67844 | 0.047335 | |
| KIAA1033 | -7.94268 | 0.0001 | |
| SH3D20 | 1.287651 | 0.018375 | |
| PRKX | -2.1067 | 0.003198 | |
| C14orf138 | -3.43083 | 0.0397 | |
| C14orf135 | -6.42358 | 0.007621 | |
| C14orf132 | -5.63059 | 0.041273 | |
| SPNS1 | 9.303674 | 0.000301 | |
| MGC70857 | 6.921616 | 0.003302 | |
| RHPN1 | 1.806399 | 0.014752 | |
| SRRM2 | -2.04884 | 0.008504 | |
| SEPX1 | 4.00698 | 0.008615 | |
| PDK3 | -0.90669 | 0.041419 | |
| PDK2 | 4.103047 | 0.00917 | |
| LNPEP | -5.11235 | 0.008172 | |
| ALPI | 13.92848 | 0.030825 | |
| ZNF292 | -1.38448 | 0.037478 | |
| ORC2L | -0.73222 | 0.029567 | |
| PUF60 | 7.380083 | 0.003408 | |
| SCN2B | -2.96639 | 0.031345 | |
| CEP120 | -7.87898 | 0.005109 | |
| NARFL | 6.095202 | 0.002047 | |
| ARHGAP29 | -9.74795 | 0.004363 | |
| NFKBIL2 | 2.447467 | 0.002358 | |
| NFKBIL1 | 0.351979 | 0.003198 | |
| PCGF2 | -1.35659 | 0.040879 | |
| ATG2B | -8.30478 | 0.003091 | |
| RLIM | -0.63214 | 0.032606 | |
| PCGF6 | -0.63963 | 0.00917 | |
| FEM1C | -3.7161 | 0.029693 | |
| GOLGA8B | -4.73285 | 0.019444 | |
| RSAD1 | 4.096308 | 0.023862 | |
| CHD6 | -6.02543 | 0.012216 | |
| CHD9 | -7.51168 | 0.00806 | |
| CHD8 | -3.6713 | 0.022892 | |
| CAP1 | -1.29587 | 0.015908 | |
| C3orf59 | -7.53607 | 0.004895 | |
| RAB3GAP2 | -1.31572 | 0.005004 | |
| PIKFYVE | -5.32907 | 0.002776 | |
| TRPC1 | -5.1182 | 0.046926 | |
| EFHC2 | -1.44094 | 0.046777 | |
| PRDM16 | -2.42117 | 0.027312 | |
| ELP2 | 9.106595 | 0.022656 | |
| TARS2 | 3.523582 | 0.034918 | |
| POLRMT | 2.42754 | 0.001631 | |
| SGSM1 | -8.4428 | 0.005217 | |
| KRIT1 | -4.47157 | 0.03645 | |
| FAHD1 | 3.627787 | 0.012333 | |
| C4orf32 | -4.76732 | 0.034651 | |
| SRD5A3 | 6.340659 | 0.003831 | |
| ZFP1 | -2.47717 | 0.005217 | |
| ZNF510 | -6.52692 | 0.009727 | |
| C10orf91 | 12.74003 | 0.001839 | |
| C10orf93 | -4.3631 | 0.006523 | |
| PSMB7 | 10.02504 | 0.000301 | |
| PSMB6 | 1.277032 | 0.030958 | |
| HDHD3 | 0.853705 | 0.009612 | |
| LRRC23 | 1.588879 | 0.0267 | |
| KLHDC5 | -4.3215 | 0.01568 | |
| CRY2 | -5.53981 | 0.027184 | |
| FAM155A | -3.10957 | 0.007399 | |
| C9orf142 | 2.086605 | 0.002985 | |
| GLTP | 2.503997 | 0.028431 | |
| BEX2 | -3.80841 | 0.042069 | |
| SULT1A3 | 4.22616 | 0.032229 | |
| GNA13 | -2.48911 | 0.027692 | |
| NOL9 | -7.38538 | 0.009841 | |
| SH2B1 | 2.307007 | 0.040494 | |
| IL6ST | -7.1547 | 0.018375 | |
| SBF2 | -10.8027 | 0.001116 | |
| C11orf31 | 8.618556 | 0.001219 | |
| C11orf30 | -1.85084 | 0.006742 | |
| R3HDM1 | -0.41283 | 0.037076 | |
| LOC284276 | -1.45953 | 0.0151 | |
| LOC100134713 | 0.54118 | 0.009612 | |
| LMBRD2 | -11.1034 | 0.000707 | |
| ZFP62 | -3.83181 | 0.001735 | |
| SRFBP1 | -4.0664 | 0.001116 | |
| BCAT1 | -3.89542 | 0.00806 | |
| LOC283267 | -3.32353 | 0.023983 | |
| MACROD2 | -2.43208 | 0.001219 | |
| AKAP5 | -1.88838 | 0.022774 | |
| AKAP8 | 1.400666 | 0.037478 | |
| TUBE1 | -2.75237 | 0.009279 | |
| RLF | -1.12164 | 0.007949 | |
| KRR1 | -1.48138 | 0.044736 | |
| STRN | -4.93936 | 0.002047 | |
| CCL27 | 9.795241 | 0.040494 | |
| CCL26 | 5.219828 | 0.040368 | |
| C8orf42 | -3.09747 | 0.014637 | |
| C8orf40 | 9.482692 | 0.000605 | |
| FAM70A | -5.13342 | 0.043928 | |
| DNAJC4 | 1.568613 | 0.004895 | |
| DNAJC6 | -2.16584 | 0.039053 | |
| DNAJC1 | 7.229326 | 0.008172 | |
| ZNF701 | -1.09968 | 0.027432 | |
| GOSR1 | -2.14002 | 0.024839 | |
| SSB | -1.12936 | 0.012901 | |
| RAP1B | -0.85324 | 0.043642 | |
| RAP1A | -2.47787 | 0.010064 | |
| MAP3K4 | -3.26894 | 0.005217 | |
| ZNF837 | 0.014354 | 0.011302 | |
| SLC23A2 | -0.90412 | 0.023862 | |
| IPO11 | -6.51447 | 0.01279 | |
| ZNF410 | -3.94303 | 0.026332 | |
| FAM91A1 | -0.47494 | 0.025838 | |
| MTCP1NB | 4.446622 | 0.001322 | |
| TPSG1 | 2.637715 | 0.022291 | |
| ZNF573 | -4.30551 | 0.012446 | |
| LIMD1 | -2.71578 | 0.020976 | |
| PEX3 | -1.57851 | 0.024103 | |
| LDLR | -2.52541 | 0.030958 | |
| CP110 | -1.09106 | 0.003831 | |
| C16orf58 | 4.971599 | 0.033127 | |
| C16orf52 | -1.52699 | 0.024471 | |
| CDK5RAP3 | 8.697817 | 0.0001 | |
| NUDT16L1 | 0.732378 | 0.036699 | |
| ITCH | -5.70257 | 0.001839 | |
| CCDC144B | -3.41047 | 0.028945 | |
| CCDC144A | -3.06649 | 0.037478 | |
| BAX | 2.169877 | 0.010402 | |
| PRKCDBP | 3.998568 | 0.025598 | |
| SLC37A2 | 8.600614 | 0.008724 | |
| PMPCA | 4.287935 | 0.041419 | |
| CDK12 | -0.10392 | 0.021571 | |
| SRRT | 0.967868 | 0.048705 | |
| CDK17 | -4.5564 | 0.046646 | |
| GZF1 | -0.67705 | 0.038527 | |
| AASDH | -4.42211 | 0.00288 | |
| ZNF304 | -1.77274 | 0.00806 | |
| ZNF302 | -1.57577 | 0.044206 | |
| PIH1D1 | 0.950463 | 0.019801 | |
| RND2 | -3.93072 | 0.014869 | |
| RND3 | -1.70123 | 0.042323 | |
| PPP3CA | -1.99707 | 0.022044 | |
| PPP3CB | -1.077 | 0.014057 | |
| LIF | -1.06077 | 0.038396 | |
| KIAA1543 | 1.394008 | 0.026087 | |
| LRRC41 | -5.16037 | 0.047891 | |
| LRRC40 | -0.44595 | 0.001014 | |
| LRRC45 | 1.828944 | 0.000503 | |
| LRRC47 | 3.749789 | 0.006302 | |
| HDAC10 | 1.908266 | 0.037339 | |
| B3GNT2 | -1.46859 | 0.004363 | |
| ADRA2C | -1.7114 | 0.018141 | |
| PWWP2A | -13.5957 | 0.000402 | |
| PBRM1 | -5.62742 | 0.007178 | |
| DAAM1 | -5.3868 | 0.003198 | |
| DAAM2 | -3.61324 | 0.035422 | |
| XAF1 | -1.72627 | 0.045554 | |
| GUK1 | 2.831657 | 0.024349 | |
| SNX21 | 2.506581 | 0.018616 | |
| KAZ | 0.874364 | 0.04526 | |
| TASP1 | -2.39404 | 0.007399 | |
| LOC100133331 | -3.9398 | 0.048849 | |
| ANGPT2 | -2.11734 | 0.023623 | |
| ANGPT1 | -1.59951 | 0.048158 | |
| ZFPM2 | -6.0667 | 0.029201 | |
| C1orf53 | 0.706155 | 0.030199 | |
| C1orf56 | 3.72748 | 0.00751 | |
| C1orf55 | -3.46328 | 0.005756 | |
| TRIM7 | 8.906093 | 0.000402 | |
| RGP1 | -4.8539 | 0.013479 | |
| FCF1 | -7.88176 | 0.007399 | |
| GUCA1B | -1.64249 | 0.03957 | |
| C17orf106 | 0.375215 | 0.017788 | |
| C17orf101 | 6.312885 | 0.004043 | |
| ATR | -3.25165 | 0.014057 | |
| ADAM33 | -6.11815 | 0.01256 | |
| PDE11A | -2.81271 | 0.038527 | |
| RAB30 | -3.00538 | 0.022171 | |
| AAMP | 2.428539 | 0.028195 | |
| LRRFIP1 | -3.30898 | 0.025838 | |
| FLJ46111 | 4.270378 | 0.009841 | |
| TTC22 | 0.020533 | 0.021209 | |
| ZNF572 | 0.597619 | 0.045554 | |
| CARS | -1.09655 | 0.046777 | |
| SYCP2 | -4.21179 | 0.041419 | |
| NDUFS1 | -2.67713 | 0.012446 | |
| NSMCE1 | 4.439998 | 0.004363 | |
| PRKAA1 | -2.39438 | 0.027184 | |
| C3orf39 | 4.944009 | 0.013709 | |
| USH1G | 6.283274 | 0.031978 | |
| MATN2 | -0.95549 | 0.028559 | |
| KBTBD10 | -1.76247 | 0.025472 | |
| FFAR2 | -2.13602 | 0.016845 | |
| DDX21 | -1.11684 | 0.009498 | |
| ZNF90 | 2.729009 | 0.024103 | |
| UNC13B | -0.29229 | 0.006962 | |
| FAM3C | -1.7264 | 0.011418 | |
| CCDC76 | -6.02235 | 0.001528 | |
| CCDC75 | -4.95642 | 0.034524 | |
| CGRRF1 | -10.0215 | 0.009388 | |
| SLAIN2 | -6.43147 | 0.005004 | |
| ZFYVE26 | -1.87601 | 0.044736 | |
| STOML1 | 1.406832 | 0.016723 | |
| NT5E | -4.47097 | 0.007399 | |
| ZNF431 | -3.13393 | 0.006742 | |
| LGALS9C | -0.37526 | 0.023136 | |
| MAP1A | -3.64917 | 0.045404 | |
| MAP1B | -2.33999 | 0.049407 | |
| IGSF9B | -2.40717 | 0.037874 | |
| LBX2 | 2.013805 | 0.015215 | |
| MAP1S | 0.733952 | 0.020853 | |
| ZNF430 | -3.85017 | 0.045554 | |
| ZSCAN5A | -0.61115 | 0.03826 | |
| FRMD5 | -0.64668 | 0.021329 | |
| FRMD6 | -9.98896 | 0.004469 | |
| PLCD4 | -1.30606 | 0.014404 | |
| MED13L | -6.29957 | 0.007621 | |
| PTCD3 | -3.19553 | 0.029443 | |
| PTCD1 | 1.701603 | 0.034264 | |
| HLTF | -2.11076 | 0.009612 | |
| AGFG1 | -2.14264 | 0.0001 | |
| RPL18A | 0.437038 | 0.016845 | |
| DACT3 | -4.91081 | 0.033758 | |
| VPS41 | -6.50217 | 0.006852 | |
| SLC15A4 | -3.88365 | 0.009727 | |
| NAA16 | -5.79783 | 0.021449 | |
| EPB41L5 | -9.65223 | 0.003514 | |
| ACSS3 | 2.445663 | 0.014985 | |
| ACSS2 | 7.744434 | 0.012101 | |
| FLJ43860 | 19.5848 | 0.006302 | |
| VPS4B | -4.92041 | 0.023014 | |
| GMEB2 | 2.047133 | 0.049954 | |
| VHL | -7.17917 | 0.005326 | |
| LRRN1 | -6.66277 | 0.010849 | |
| ALMS1P | -0.74686 | 0.034524 | |
| RECK | -2.03934 | 0.03109 | |
| MRPL34 | 5.346143 | 0.023014 | |
| ZNF813 | -6.96734 | 0.003831 | |
| SPTBN5 | 3.771447 | 0.032606 | |
| SNORD116-28 | -11.4545 | 0.000605 | |
| KLF13 | -6.2261 | 0.000503 | |
| KLF15 | 3.247669 | 0.025472 | |
| SLC44A2 | 12.44408 | 0.001528 | |
| SLC44A5 | -4.52459 | 0.009059 | |
| PCNX | -2.94696 | 0.025219 | |
| HDAC1 | 3.118135 | 0.043102 | |
| NDUFS6 | 0.892153 | 0.003938 | |
| NDUFS7 | 2.174594 | 0.001631 | |
| FAM198B | -6.18085 | 0.015215 | |
| ZRANB3 | -10.8754 | 0.001322 | |
| HBB | 3.34084 | 0.018616 | |
| MUC20 | 3.912259 | 0.011752 | |
| PPT2 | 4.430017 | 0.031477 | |
| INPP4A | -5.93219 | 0.008945 | |
| LOC145474 | 8.27932 | 0.019444 | |
| PHC3 | -1.45895 | 0.026937 | |
| GPR108 | 6.086279 | 0.001943 | |
| UBE2G2 | -0.80428 | 0.013479 | |
| SAFB | 0.211308 | 0.003091 | |
| MGMT | 1.919196 | 0.049407 | |
| H2AFX | 6.732778 | 0.020617 | |
| RNF6 | -4.11857 | 0.019563 | |
| RNF7 | 3.799985 | 0.011867 | |
| RYBP | -6.14837 | 0.017318 | |
| NCAPG2 | -1.91447 | 0.036951 | |
| CLTB | 2.364776 | 0.013018 | |
| PPIG | -9.4422 | 0.000503 | |
| RECQL | -5.77861 | 0.002255 | |
| PRKRIR | -0.89975 | 0.029443 | |
| UQCRC1 | 1.750363 | 0.013018 | |
| RECQL4 | 1.317536 | 0.008504 | |
| HYDIN | -8.48628 | 0.000201 | |
| GNL3L | -4.74299 | 0.025963 | |
| RASA1 | -4.53056 | 0.027312 | |
| RABEP1 | -3.83074 | 0.027825 | |
| ANKS1B | -5.99037 | 0.008615 | |
| C1orf77 | 3.593745 | 0.031345 | |
| ANKS1A | -8.95008 | 0.000402 | |
| TSTA3 | 1.397868 | 0.030069 | |
| GNRH1 | 6.616611 | 0.013134 | |
| RPL12 | 5.187982 | 0.00773 | |
| NDUFA7 | 0.515002 | 0.023983 | |
| SFRS12 | -1.93439 | 0.049815 | |
| SFRS11 | -1.26777 | 0.004149 | |
| UPK3BL | 5.308101 | 0.006413 | |
| AAK1 | -10.3584 | 0.0001 | |
| PBX4 | 6.137916 | 0.006852 | |
| KLC4 | 1.719035 | 0.01802 | |
| TMEM167B | -1.94566 | 0.014985 | |
| ZDHHC13 | -7.22926 | 0.00751 | |
| HIPK2 | -4.78243 | 0.04569 | |
| HIPK1 | -2.81692 | 0.013018 | |
| HIPK4 | 3.620768 | 0.014175 | |
| RPS3 | 4.697725 | 0.035926 | |
| LEMD3 | -4.47071 | 0.013479 | |
| NCAN | 13.65656 | 0.038396 | |
| SLC16A6 | -4.41053 | 0.00806 | |
| OSR2 | 1.957336 | 0.037615 | |
| LARP1 | -2.84471 | 0.005109 | |
| LARP4 | -3.99467 | 0.017437 | |
| TNFSF15 | -1.84713 | 0.024596 | |
| GPATCH3 | 0.094835 | 0.015215 | |
| DDX46 | -7.59789 | 0.00362 | |
| TMEM45B | -5.97704 | 0.008172 | |
| CDKN1A | -2.99366 | 0.029567 | |
| UBE2D1 | -3.15219 | 0.000402 | |
| MLL3 | -2.34814 | 0.003198 | |
| GUF1 | -0.08119 | 0.033506 | |
| STX10 | 2.210607 | 0.007399 | |
| UTP11L | -1.29637 | 0.040879 | |
| VEGFB | 0.489932 | 0.04297 | |
| CCDC51 | 1.497876 | 0.025219 | |
| CCDC57 | 3.674202 | 0.011073 | |
| RAD18 | -0.97419 | 0.022171 | |
| RPL13AP3 | 5.799195 | 0.03109 | |
| FBXO30 | -1.52388 | 0.04925 | |
| FBXO38 | -8.18273 | 0.008394 | |
| MPG | 1.958767 | 0.001425 | |
| SAT2 | 3.755048 | 0.012676 | |
| PELP1 | 2.184591 | 0.045119 | |
| MBOAT4 | 1.420841 | 0.015562 | |
| SPSB3 | 5.727697 | 0.004363 | |
| HINT2 | 3.493412 | 0.005004 | |
| HINT3 | -0.03689 | 0.027692 | |
| CDC34 | 4.908237 | 0.000605 | |
| CDC37 | 2.343183 | 0.003302 | |
| BRWD1 | -0.53802 | 0.013595 | |
| S100A3 | 5.838578 | 0.048705 | |
| LONRF3 | -0.98558 | 0.026576 | |
| BIRC2 | -2.82026 | 0.013364 | |
| CDCA7L | -5.88508 | 0.030452 | |
| ZNF365 | -5.36514 | 0.0316 | |
| APBA3 | 1.134404 | 0.001322 | |
| PGLS | 5.025453 | 0.003302 | |
| TUB | -9.05702 | 0.001116 | |
| MORC3 | -2.31208 | 0.000605 | |
| CCNA1 | -0.6705 | 0.003514 | |
| ATP13A3 | -0.07325 | 0.003938 | |
| LRRIQ1 | -1.46664 | 0.009612 | |
| TNRC6A | -3.09785 | 0.019686 | |
| ADAT1 | -4.82866 | 0.014869 | |
| ADAT3 | 4.445837 | 0.004149 | |
| E2F3 | -0.39318 | 0.017082 | |
| SCAF1 | 1.429351 | 0.019563 | |
| ACBD3 | -2.47056 | 0.043239 | |
| ACBD6 | 2.564738 | 0.030958 | |
| ACBD4 | 4.062954 | 0.005326 | |
| C2orf29 | -0.14966 | 0.049546 | |
| C2orf28 | 11.18816 | 0.000707 | |
| RPGR | -7.25034 | 0.002985 | |
| OSTF1 | 1.412933 | 0.04706 | |
| ZNF331 | -2.04256 | 0.009727 | |
| GNG13 | 3.174147 | 0.045119 | |
| LOC121952 | -0.93145 | 0.024966 | |
| URB1 | -1.58664 | 0.043928 | |
| RPLP1 | 5.368495 | 0.001943 | |
| HPCAL4 | -0.72189 | 0.003938 | |
| SEC24D | -8.75592 | 0.023623 | |
| REV3L | -5.57454 | 0.010964 | |
| SEC24A | -3.49147 | 0.004788 | |
| SEC24C | -0.47555 | 0.018258 | |
| PPIA | 4.102142 | 0.025963 | |
| CSMD2 | -5.05784 | 0.016845 | |
| IPP | -8.72541 | 0.004469 | |
| C16orf93 | 1.407972 | 0.04569 | |
| LOC100130581 | 0.581782 | 0.048026 | |
| LAMB2 | 2.313987 | 0.000605 | |
| CYSLTR1 | -1.297 | 0.022171 | |
| SRXN1 | -3.2164 | 0.013595 | |
| CCDC9 | 0.190586 | 0.031345 | |
| MGA | -2.3587 | 0.044065 | |
| RSPH3 | -3.02869 | 0.035926 | |
| NSD1 | -6.61428 | 0.002776 | |
| R3HCC1 | 1.99697 | 0.010176 | |
| LOC168474 | -3.38495 | 0.012101 | |
| PLA2G15 | 0.952005 | 0.032994 | |
| C7orf11 | 2.729377 | 0.021807 | |
| C7orf13 | 2.848083 | 0.022418 | |
| AZI2 | -5.05254 | 0.000503 | |
| AZI1 | 1.989603 | 0.001839 | |
| TARS | -1.47509 | 0.003831 | |
| NUDT17 | 1.994794 | 0.040368 | |
| NUDT14 | 1.562732 | 0.007287 | |
| NUDT18 | 2.924984 | 0.002567 | |
| UBE2Q2P1 | -3.58797 | 0.031845 | |
| PHACTR4 | -5.29453 | 0.001839 | |
| MTMR15 | -2.58963 | 0.039053 | |
| CDADC1 | -5.04517 | 0.018258 | |
| L3MBTL | -8.88765 | 0.00751 | |
| DRG2 | 0.735921 | 0.030323 | |
| ZNF205 | 2.403817 | 0.001839 | |
| SNAPC3 | -0.84413 | 0.026087 | |
| GNPTG | 3.246359 | 0.002462 | |
| CDC42EP5 | 4.20984 | 0.036699 | |
| ZNF354C | -11.0228 | 0.000201 | |
| TBKBP1 | 0.229144 | 0.037615 | |
| AHCY | 0.050475 | 0.048566 | |
| ABI2 | -2.39849 | 0.009841 | |
| NKTR | -5.51855 | 0.012101 | |
| RHOH | -5.48368 | 0.013824 | |
| ZBED5 | -8.4057 | 0.0001 | |
| CHN2 | -10.2389 | 0.000301 | |
| IL27 | -1.02942 | 0.021807 | |
| AXL | -8.98371 | 0.00468 | |
| ZNF548 | -0.06247 | 0.035045 | |
| ELOF1 | 3.498393 | 0.00362 | |
| CLINT1 | -3.33235 | 0.005435 | |
| BRCA2 | -3.8827 | 0.020733 | |
| ZNF439 | -10.0327 | 0.011073 | |
| AHRR | -1.95658 | 0.019444 | |
| ADCY1 | 1.581542 | 0.049111 | |
| KIF22 | 2.664726 | 0.010964 | |
| RNASEH2C | 1.119327 | 0.011529 | |
| C12orf4 | -1.81109 | 0.006523 | |
| XRCC3 | 0.964319 | 0.027825 | |
| XRCC2 | -6.89485 | 0.002358 | |
| SMURF2 | -3.82676 | 0.017082 | |
| ZNF669 | -4.3732 | 0.045404 | |
| MIER1 | -6.34624 | 0.000809 | |
| MIER2 | 3.070251 | 0.004895 | |
| NCRNA00116 | 2.109502 | 0.007287 | |
| LMNB2 | 0.993259 | 0.025219 | |
| MRPS24 | 4.173337 | 0.008834 | |
| CTSL2 | 3.45533 | 0.030452 | |
| INSIG2 | -5.87237 | 0.000605 | |
| NXPH4 | 3.879296 | 0.02882 | |
| NDUFB11 | 2.12741 | 0.025339 | |
| KCNA6 | -0.02771 | 0.040621 | |
| JMJD8 | 3.912043 | 0.008282 | |
| ZHX1 | -4.44032 | 0.005648 | |
| CCDC39 | -5.15451 | 0.002567 | |
| FAM128B | 1.123423 | 0.044466 | |
| FAM128A | 0.318676 | 0.044736 | |
| SLC16A8 | 10.62604 | 0.004043 | |
| SARS2 | 2.513599 | 0.036066 | |
| HS3ST1 | -4.94957 | 0.046509 | |
| CYFIP2 | -11.3837 | 0.010625 | |
| WDR35 | -0.49024 | 0.017788 | |
| PXDNL | -1.73026 | 0.004255 | |
| MRPS34 | 2.048939 | 0.000707 | |
| RPS15AP10 | -4.14926 | 0.031221 | |
| GTPBP3 | 2.651474 | 0.023381 | |
| GTPBP2 | -1.79046 | 0.040747 | |
| PARP10 | 1.815217 | 0.001322 | |
| POR | 5.725529 | 0.021807 | |
| NPEPL1 | 9.045303 | 0.001839 | |
| LRFN3 | 5.274283 | 0.045966 | |
| LRFN2 | 2.252306 | 0.013249 | |
| LYRM7 | -4.26742 | 0.023744 | |
| DMXL1 | -6.58734 | 0.000201 | |
| MTM1 | -4.48737 | 0.006962 | |
| TRIM22 | -4.10737 | 0.044736 | |
| CSNK1G2 | 0.028933 | 0.0267 | |
| CSNK1G3 | -5.30371 | 0.038658 | |
| CSNK1G1 | -0.90578 | 0.006302 | |
| SERPINB2 | 5.849693 | 0.039974 | |
| FAM76A | -1.23378 | 0.037615 | |
| CX3CR1 | -1.62232 | 0.048566 | |
| LOC613037 | 0.478273 | 0.042069 | |
| C6orf62 | -1.60605 | 0.002462 | |
| ATP8B4 | -5.02163 | 0.034524 | |
| ELP3 | 4.110176 | 0.008282 | |
| TRIM58 | -2.66518 | 0.012216 | |
| ASPHD2 | -8.75456 | 0.001528 | |
| FBXL4 | -2.03632 | 0.021925 | |
| DEK | -2.07657 | 0.029074 | |
| FBXL7 | -5.78288 | 0.038138 | |
| KLHDC4 | 0.943883 | 0.002567 | |
| UBAP1 | -0.56757 | 0.01256 | |
| ASXL1 | -4.36636 | 0.005648 | |
| HDDC3 | 2.936568 | 0.011418 | |
| ZBTB39 | -5.44102 | 0.006302 | |
| ZBTB38 | -2.75453 | 0.016845 | |
| NIPSNAP3B | -2.78437 | 0.031221 | |
| C1orf133 | 4.62508 | 0.023381 | |
| ZBTB34 | -2.71885 | 0.024223 | |
| KLHL32 | -2.85405 | 0.002671 | |
| KLHL30 | 6.579091 | 0.020271 | |
| TMEM92 | -1.6005 | 0.023983 | |
| TAB2 | -5.56032 | 0.005756 | |
| AGAP11 | -0.3938 | 0.044206 | |
| SGTB | -0.46852 | 0.037748 | |
| TRIM44 | -5.82169 | 0.001116 | |
| FXC1 | -2.43357 | 0.007178 | |
| TRAF6 | -3.28204 | 0.006962 | |
| REV1 | -10.0702 | 0.004255 | |
| ATP5D | 3.112762 | 0.003302 | |
| AURKAIP1 | 1.43788 | 0.04526 | |
| CITED4 | 3.258089 | 0.002255 | |
| PFDN5 | 1.149817 | 0.031978 | |
| OGT | -5.88659 | 0.009727 | |
| CSNK1A1L | -8.34617 | 0.013479 | |
| PRPF39 | -6.08082 | 0.003198 | |
| RIN1 | 3.106213 | 0.010964 | |
| NSUN3 | -4.04378 | 0.008172 | |
| RPS11 | 7.090546 | 0.039307 | |
| RPS17 | 5.700667 | 0.034132 | |
| RPS15 | 5.772019 | 0.000809 | |
| CLPP | 6.745152 | 0.000402 | |
| SLC16A4 | -1.93273 | 0.048284 | |
| RIPK1 | -5.85584 | 0.049685 | |
| LATS1 | -5.61807 | 0.009727 | |
| ODZ2 | -4.39584 | 0.048849 | |
| CRABP1 | -0.30719 | 0.025219 | |
| ZNF417 | -3.18203 | 0.00751 | |
| GGT7 | 1.631672 | 0.034524 | |
| HAUS8 | 6.409462 | 0.012676 | |
| ACTN4 | 9.473557 | 0.004043 | |
| HAUS7 | 5.543426 | 0.003514 | |
| PTPRN | -0.5189 | 0.014985 | |
| CPD | -4.6587 | 0.010064 | |
| GAPVD1 | -2.7453 | 0.04379 | |
| YIPF6 | -2.49264 | 0.010064 | |
| RPL7L1 | -2.08441 | 0.029443 | |
| CYP46A1 | -2.1853 | 0.005326 | |
| MRPL41 | 3.346344 | 0.008282 | |
| LOC127841 | 5.659311 | 0.045821 | |
| ABLIM1 | -1.47226 | 0.038138 | |
| MAMSTR | 2.105579 | 0.034782 | |
| ABLIM3 | -6.02722 | 0.022291 | |
| HMGN2 | 6.082115 | 0.011302 | |
| RAB6A | -5.85633 | 0.004043 | |
| DULLARD | 2.058221 | 0.047891 | |
| TOX3 | -10.4266 | 0.03109 | |
| MAP1LC3A | 4.24059 | 0.005866 | |
| GPS1 | 1.944496 | 0.02509 | |
| ZNF346 | -7.0884 | 0.004895 | |
| SMAP2 | -6.14775 | 0.027184 | |
| ARL5A | -2.3678 | 0.001322 | |
| ARL5B | -1.20712 | 0.008394 | |
| KRT16 | 6.378821 | 0.023257 | |
| KRT15 | 5.380367 | 0.009059 | |
| KRT13 | 13.80913 | 0.027949 | |
| GABRD | 1.264079 | 0.023136 | |
| GIGYF1 | 0.579757 | 0.014175 | |
| NBPF3 | -6.41168 | 0.005435 | |
| NAPEPLD | -2.47691 | 0.041143 | |
| HGSNAT | 6.193966 | 0.018733 | |
| TM9SF3 | -3.19657 | 0.017437 | |
| C10orf12 | -2.86338 | 0.008834 | |
| N4BP2 | -4.02259 | 0.018141 | |
| NAT6 | 0.2274 | 0.035546 | |
| FAM41C | -4.21454 | 0.003938 | |
| MFSD6L | -2.43705 | 0.03109 | |
| COX5B | 10.10118 | 0.001116 | |
| WDR11 | -4.20698 | 0.004043 | |
| KIAA2026 | -9.84419 | 0.000402 | |
| CPSF1 | 2.915064 | 0.017198 | |
| MAD1L1 | 0.000307 | 0.002151 | |
| ACAA1 | 3.616312 | 0.009841 | |
| PSIMCT-1 | -4.41633 | 0.0397 | |
| SLC35F1 | -1.19121 | 0.039053 | |
| MBD5 | -4.98174 | 0.005435 | |
| LOC653566 | -5.53961 | 0.008394 | |
| TSPAN10 | 8.488403 | 0.001014 | |
| TSPAN12 | -1.18324 | 0.005974 | |
| REXO1 | 1.348731 | 0.006742 | |
| FAM13A | -1.85466 | 0.027184 | |
| REXO4 | 1.610099 | 0.027825 | |
| SLC25A16 | -0.56384 | 0.003198 | |
| SLC25A14 | 2.713259 | 0.048425 | |
| SLC25A12 | -7.85626 | 0.004043 | |
| ZNF321 | -2.36477 | 0.003091 | |
| CCDC12 | 1.059849 | 0.01029 | |
| TMUB1 | 2.610117 | 0.0151 | |
| ZNF320 | -5.41847 | 0.000912 | |
| JAZF1 | -5.94812 | 0.017552 | |
| FAM100A | 4.088786 | 0.001219 | |
| SYNCRIP | -2.18097 | 0.023136 | |
| ZNF280D | -1.25989 | 0.036574 | |
| ZNF280C | -5.23094 | 0.011867 | |
| VIT | -0.89769 | 0.010849 | |
| PARN | 3.217177 | 0.034392 | |
| FUS | 0.386855 | 0.026818 | |
| ALG1 | 6.837759 | 0.000503 | |
| STAM | -0.43473 | 0.007178 | |
| FUK | 3.477842 | 0.013018 | |
| TTC14 | -5.38517 | 0.009841 | |
| NOSIP | 4.67857 | 0.004788 | |
| GCC2 | -8.76405 | 0.005217 | |
| TBC1D8B | -2.62191 | 0.012676 | |
| CLDN3 | 1.00782 | 0.032994 | |
| TBC1D19 | -7.47421 | 0.004788 | |
| MPHOSPH10 | -0.47157 | 0.018616 | |
| IRF6 | -4.01444 | 0.005974 | |
| IRF9 | -6.85718 | 0.030452 | |
| C6orf47 | 1.11073 | 0.010849 | |
| CLK1 | -2.07537 | 0.035674 | |
| CLK4 | -5.88563 | 0.00362 | |
| LOC90784 | -8.26503 | 0.01568 | |
| MRPL23 | 2.530118 | 0.005004 | |
| TMEM150C | -2.23813 | 0.020386 | |
| SPATA13 | -6.29031 | 0.002255 | |
| C1orf151 | 3.867109 | 0.037076 | |
| KLHL18 | -2.14422 | 0.042712 | |
| TRIM66 | -5.4632 | 0.004895 | |
| SNRNP70 | 2.977434 | 0.007399 | |
| OMD | -5.62311 | 0.020853 | |
| C2orf63 | -2.74065 | 0.027312 | |
| C2orf60 | -4.48462 | 0.001839 | |
| C2orf67 | -4.3132 | 0.009841 | |
| NPTX2 | -0.3564 | 0.029693 | |
| NAGPA | 4.413974 | 0.001116 | |
| FLJ45340 | -4.23911 | 0.005217 | |
| SLU7 | -5.96928 | 0.002985 | |
| SERTAD1 | 3.817547 | 0.003725 | |
| LOC728190 | -8.58611 | 0.002776 | |
| GIN1 | -5.10118 | 0.028559 | |
| TTPA | -0.69829 | 0.003198 | |
| TFAP4 | 6.450989 | 0.000912 | |
| PAQR5 | -5.02601 | 0.015562 | |
| SNORD116-20 | -3.94087 | 0.014175 | |
| CYBA | 1.725531 | 0.00288 | |
| MSMP | -3.38106 | 0.018375 | |
| FAM126B | -2.3622 | 0.003091 | |
| TCTEX1D2 | 1.277981 | 0.04297 | |
| ZNF653 | 3.788716 | 0.006631 | |
| HSF1 | 7.544301 | 0.007069 | |
| HSF2 | -0.82521 | 0.042323 | |
| PRPF4B | -3.19507 | 0.007949 | |
| C7orf50 | 1.585249 | 0.001528 | |
| C7orf53 | -3.88458 | 0.000605 | |
| C7orf59 | 1.46153 | 0.014985 | |
| PRSS50 | 3.955829 | 0.046646 | |
| POC1A | 1.035955 | 0.03957 | |
| RAPH1 | -4.73506 | 0.043371 | |
| TAOK1 | -2.05715 | 0.012101 | |
| PARK7 | 3.269631 | 0.013479 | |
| ZNF473 | -0.29572 | 0.020976 | |
| GUSB | 7.391228 | 0.013249 | |
| PRSS53 | 3.621399 | 0.020733 | |
| TELO2 | 0.918788 | 0.006084 | |
| MDM4 | -8.51102 | 0.00784 | |
| ROR2 | -9.63453 | 0.018375 | |
| NR1D2 | -4.58916 | 0.028074 | |
| KPNA5 | -4.71209 | 0.000605 | |
| KIN | -0.85886 | 0.020386 | |
| ZNF397OS | -1.02248 | 0.015215 | |
| TTC30A | -2.9504 | 0.01802 | |
| SPATA18 | -2.82989 | 0.023623 | |
| FRS2 | -0.31107 | 0.0435 | |
| GOLT1B | -1.53832 | 0.049954 | |
| CRK | -2.40765 | 0.038138 | |
| RPS16 | 8.356189 | 0.037748 | |
| TNFRSF19 | -5.57374 | 0.017552 | |
| GLDN | -0.53636 | 0.007621 | |
| PC | 2.302907 | 0.031978 | |
| ROMO1 | 1.869688 | 0.009059 | |
| DHRS12 | 2.252437 | 0.022774 | |
| ASPSCR1 | 2.303502 | 0.003514 | |
| PPP1R9B | 3.86703 | 0.000809 | |
| TBC1D15 | -0.43443 | 0.017437 | |
| C6orf217 | -0.54828 | 0.031978 | |
| GCFC1 | -1.83411 | 0.002567 | |
| ARNTL2 | -3.0357 | 0.02509 | |
| WDR83 | 0.641613 | 0.006192 | |
| S100A4 | 5.284237 | 0.027312 | |
| IGBP1 | 2.919508 | 0.001839 | |
| CD63 | 3.553442 | 0.013134 | |
| NDUFA13 | 0.449219 | 0.027562 | |
| S100A7 | 6.995681 | 0.046777 | |
| GABPA | -5.18149 | 0.001014 | |
| AOX1 | -4.16634 | 0.006631 | |
| LMF1 | 9.229452 | 0.002151 | |
| SLC16A14 | -7.83067 | 0.002047 | |
| USP9X | -2.57721 | 0.004363 | |
| CGGBP1 | -2.15985 | 0.007399 | |
| UFSP1 | 1.488768 | 0.00751 | |
| PHGDH | 3.188317 | 0.03957 | |
| DGKZ | 1.997759 | 0.01802 | |
| C10orf32 | -5.7354 | 0.046364 | |
| HSPB1 | 0.198048 | 0.014175 | |
| HSPB8 | -0.35981 | 0.037211 | |
| INO80D | -3.984 | 0.005435 | |
| INO80B | 4.915551 | 0.003831 | |
| DIS3L2 | -2.63577 | 0.011867 | |
| KIAA0913 | -2.08174 | 0.013134 | |
| CMPK1 | -3.1252 | 0.005217 | |
| LMF2 | 3.529825 | 0.021807 | |
| TEX264 | 3.476412 | 0.000503 | |
| PTDSS2 | 2.981679 | 0.017437 | |
| KRTCAP3 | 0.301858 | 0.002671 | |
| LIG4 | -4.63346 | 0.000605 | |
| LIG1 | 3.219232 | 0.029693 | |
| BDP1 | -5.70317 | 0.001014 | |
| MRS2P2 | -1.60873 | 0.017902 | |
| ALS2CR4 | -1.38436 | 0.001014 | |
| KCTD17 | 0.384941 | 0.022418 | |
| TNFSF13B | -2.16942 | 0.049685 | |
| ZKSCAN3 | -6.89168 | 0.003091 | |
| ZKSCAN5 | -4.90885 | 0.013709 | |
| GLIS3 | -4.38196 | 0.044065 | |
| GLIS1 | -4.27484 | 0.022044 | |
| FNBP4 | -10.4612 | 0.003198 | |
| SLC25A36 | -2.01072 | 0.025219 | |
| TMEM106B | -6.10696 | 0.026937 | |
| TMEM182 | -2.37586 | 0.01452 | |
| TMEM181 | -1.52035 | 0.029822 | |
| TMEM187 | 1.337068 | 0.011984 | |
| IL10RB | 2.930316 | 0.005004 | |
| RIBC1 | 2.179673 | 0.040747 | |
| PRH1 | -5.17554 | 0.044065 | |
| PGBD4 | -6.41538 | 0.012101 | |
| ZMAT2 | 3.509346 | 0.005974 | |
| TAF3 | -5.12701 | 0.016964 | |
| ELMO2 | -0.87757 | 0.009279 | |
| AGPS | -3.12714 | 0.024719 | |
| CCNG2 | -2.91457 | 0.030452 | |
| CNO | 3.120807 | 0.041557 | |
| PIK3C2A | -11.7705 | 0.000301 | |
| LOC644172 | -1.94993 | 0.029074 | |
| PRKCE | -9.87582 | 0.00362 | |
| N4BP1 | -1.17855 | 0.037874 | |
| HIST2H2AA3 | 4.675278 | 0.016845 | |
| VPS26A | -0.30418 | 0.020039 | |
| ZC3H7A | -2.10381 | 0.030199 | |
| OXCT1 | -3.09519 | 0.005866 | |
| KIF16B | -5.39795 | 0.037339 | |
| CIT | 0.039685 | 0.041143 | |
| TAF6L | 1.415738 | 0.045966 | |
| SNUPN | 6.087383 | 0.025838 | |
| SHPRH | -7.82141 | 0.002255 | |
| C1orf170 | 2.769639 | 0.039834 | |
| MLANA | -0.84843 | 0.039053 | |
| GPC3 | -2.77447 | 0.010964 | |
| BBS5 | -3.59724 | 0.022171 | |
| BBS7 | -4.40823 | 0.015442 | |
| C2orf49 | -3.2022 | 0.000503 | |
| C2orf42 | -1.44656 | 0.018489 | |
| AGL | -1.33409 | 0.008394 | |
| AGA | 1.694463 | 0.04379 | |
| C13orf18 | -3.35159 | 0.01452 | |
| PIK3CA | -2.15311 | 0.032863 | |
| RAB11B | 1.076521 | 0.006962 | |
| TMEM54 | 0.697587 | 0.016845 | |
| POLR3GL | 1.881275 | 0.002047 | |
| MEG3 | -5.08764 | 0.025598 | |
| RPL18 | 6.872133 | 0.009498 | |
| ZNF674 | -10.2301 | 0.0001 | |
| ZNF673 | -6.79992 | 0.003831 | |
| MAZ | 5.543726 | 0.020039 | |
| VPS8 | -3.04131 | 0.033506 | |
| MAF | -5.48256 | 0.037748 | |
| LOC100190986 | -3.69367 | 0.016372 | |
| HAS2AS | -1.04694 | 0.022171 | |
| JAM3 | -3.30075 | 0.023381 | |
| LSR | 5.10231 | 0.0235 | |
| RASEF | -4.96083 | 0.036066 | |
| KCNIP4 | -4.00145 | 0.04623 | |
| KCNIP3 | -5.8068 | 0.006523 | |
| LOC729799 | -8.15213 | 0.001322 | |
| TMEM102 | 3.612116 | 0.005109 | |
| ANAPC1 | -3.01506 | 0.015215 | |
| ANAPC2 | 1.335633 | 0.016139 | |
| ANAPC4 | -4.77183 | 0.009951 | |
| MINPP1 | -3.72797 | 0.033632 | |
| METTL2B | -3.21385 | 0.018616 | |
| VAPA | -5.04811 | 0.017437 | |
| NPHP3 | -6.19836 | 0.014752 | |
| MKI67IP | -0.86 | 0.025598 | |
| MOBP | 8.053177 | 0.018975 | |
| C6orf186 | -2.54253 | 0.026087 | |
| MYLK2 | 6.582116 | 0.021571 | |
| RGPD4 | -4.50855 | 0.008945 | |
| RGS17 | -7.6284 | 0.0001 | |
| KLRAQ1 | -0.61344 | 0.023381 | |
| RNASET2 | 5.965996 | 0.020386 | |
| RAB2B | -7.5282 | 0.001116 | |
| GCHFR | 3.463155 | 0.015562 | |
| RPL7A | 0.440138 | 0.043371 | |
| AASS | -3.9387 | 0.005004 | |
| ETNK2 | -5.54788 | 0.027692 | |
| MNDA | -3.52587 | 0.018733 | |
| HEXDC | 1.396693 | 0.016723 | |
| DHRS7B | 6.183321 | 0.010736 | |
| FPGT | -5.31926 | 0.033999 | |
| GCNT1 | -7.06087 | 0.002255 | |
| MAF1 | 9.934083 | 0.000605 | |
| EIF4E3 | -7.27809 | 0.039053 | |
| ASAH2B | -3.41841 | 0.009841 | |
| FAR1 | -3.1174 | 0.019563 | |
| WDR70 | -0.34759 | 0.038396 | |
| NQO2 | 1.771435 | 0.005435 | |
| PRPF8 | -2.13659 | 0.035295 | |
| FLJ35220 | 2.485409 | 0.018616 | |
| PRPF6 | 7.524269 | 0.009059 | |
| PCF11 | -3.42582 | 0.013595 | |
| NID1 | -3.15362 | 0.039307 | |
| C5orf42 | -8.16748 | 0.004469 | |
| IL31RA | 8.528322 | 0.023623 | |
| NCRNA00171 | -3.01033 | 0.037478 | |
| NCRNA00176 | 1.517373 | 0.04925 | |
| ARRDC1 | 1.004658 | 0.020157 | |
| ARRDC2 | 1.1374 | 0.002358 | |
| FAM45A | -2.19695 | 0.034918 | |
| LOC388796 | 3.581344 | 0.032994 | |
| FAM122C | -6.36297 | 0.004895 | |
| KCNG2 | 8.594811 | 0.024839 | |
| NUBPL | -2.74058 | 0.026937 | |
| NPB | 8.416583 | 0.02882 | |
| CEP97 | -2.8513 | 0.036825 | |
| MFSD2A | -1.41414 | 0.014175 | |
| HEXA | 7.799937 | 0.006413 | |
| ARID2 | -5.22594 | 0.013824 | |
| LOC652276 | -1.28169 | 0.025963 | |
| NLRP1 | -3.8534 | 0.041419 | |
| NPW | 4.640781 | 0.0472 | |
| CALML4 | -0.79536 | 0.000605 | |
| PFN4 | 6.325944 | 0.000707 | |
| SHISA2 | -2.55687 | 0.003091 | |
| TMEM219 | 3.046263 | 0.004149 | |
| MCTP2 | -1.1672 | 0.004255 | |
| WDR27 | -11.2371 | 0.004363 | |
| TPM4 | 4.450791 | 0.005542 | |
| LOC678655 | 2.267389 | 0.041557 | |
| SMNDC1 | -7.84231 | 0.000707 | |
| DDX19A | -2.21362 | 0.036825 | |
| NDOR1 | 1.677395 | 0.006742 | |
| PPAN | 4.782427 | 0.003514 | |
| PPP1CA | 10.60626 | 0.001219 | |
| RNF38 | -1.03112 | 0.031723 | |
| LYSMD3 | -5.28621 | 0.037748 | |
| THPO | -1.35357 | 0.035045 | |
| DCXR | 4.54648 | 0.013939 | |
| SMARCA1 | -7.98313 | 0.001735 | |
| BAZ2B | -12.5158 | 0.001322 | |
| RAB1A | -0.47362 | 0.044336 | |
| WDR47 | -6.73654 | 0.020502 | |
| OST4 | 4.311703 | 0.044466 | |
| NUDC | 0.744573 | 0.011418 | |
| PAF1 | 7.354241 | 0.001219 | |
| RUFY3 | -3.4211 | 0.000503 | |
| RUFY2 | -8.06879 | 0.000301 | |
| SKIL | -4.09462 | 0.004363 | |
| GCGR | 15.4561 | 0.005866 | |
| MYBPH | 7.239187 | 0.045821 | |
| DPH3 | -0.14665 | 0.033632 | |
| MUM1L1 | -6.64993 | 0.014985 | |
| WBP5 | -7.3677 | 0.029074 | |
| CANT1 | -4.04163 | 0.027056 | |
| SLK | -4.63284 | 0.001014 | |
| DCN | -5.55565 | 0.016964 | |
| GPAM | -0.10605 | 0.026087 | |
| DCI | 9.139986 | 0.0001 | |
| SDF4 | 1.514022 | 0.001631 | |
| PERP | 3.837357 | 0.013824 | |
| CUL5 | -3.99567 | 0.005542 | |
| CUL3 | -4.8883 | 0.012101 | |
| TRIM25 | -4.25831 | 0.040368 | |
| CREB1 | -6.22167 | 0.008394 | |
| FLRT3 | -7.12721 | 0.010402 | |
| GNPAT | 2.301912 | 0.032102 | |
| PTPN13 | -8.51786 | 0.020853 | |
| RHOT1 | -1.62563 | 0.041557 | |
| VTA1 | -2.05002 | 0.007287 | |
| KAT5 | 0.720163 | 0.014637 | |
| ZNF517 | 2.508015 | 0.018141 | |
| ZNF514 | -7.14863 | 0.002985 | |
| NBEAL1 | -5.24581 | 0.002567 | |
| AES | 3.261822 | 0.006192 | |
| DNAJC27 | -3.44315 | 0.024596 | |
| DNAJC24 | -8.65053 | 0.000201 | |
| DNAJC21 | -1.4916 | 0.04379 | |
| ARHGAP5 | -8.27876 | 0.0001 | |
| SETD1A | 5.485836 | 0.043239 | |
| RGS19 | 4.971746 | 0.023014 | |
| TMEM79 | 6.568786 | 0.03645 | |
| ZYX | 6.924809 | 0.00773 | |
| MRPL4 | 8.506507 | 0.019444 | |
| RALGAPA1 | -10.614 | 0.001735 | |
| ZNF611 | -4.51069 | 0.001116 | |
| WBSCR16 | 2.817625 | 0.015562 | |
| ZNF614 | -3.09695 | 0.03826 | |
| ZNF616 | -2.89216 | 0.021329 | |
| EIF4EBP3 | 9.453743 | 0.000605 | |
| EDDM3B | 7.193007 | 0.039053 | |
| RSBN1 | -3.13776 | 0.012446 | |
| CBLN4 | -4.26542 | 0.008834 | |
| CBLN3 | -5.06898 | 0.012676 | |
| EVI5 | -1.38605 | 0.049407 | |
| ATXN10 | -0.07457 | 0.029822 | |
| PSIP1 | -2.42317 | 0.006523 | |
| G2E3 | -8.36693 | 0.0001 | |
| RILPL1 | 1.712139 | 0.008724 | |
| CAMK1D | -4.89031 | 0.016602 | |
| ECM2 | -2.94842 | 0.011302 | |
| ECM1 | 4.182948 | 0.0267 | |
| ANKRD30A | -7.63791 | 0.039053 | |
| ANKRD30B | -2.3757 | 0.026087 | |
| RGS1 | -1.13365 | 0.016372 | |
| RGS9 | -2.16555 | 0.01992 | |
| SERTAD2 | -4.67932 | 0.00288 | |
| XIAP | -7.97554 | 0.0001 | |
| POLD3 | -1.25337 | 0.005866 | |
| EPDR1 | -4.49473 | 0.02882 | |
| KIAA0664P3 | -7.5077 | 0.015328 | |
| UBR3 | -8.4394 | 0.000402 | |
| UBR2 | -0.52399 | 0.026453 | |
| PTP4A3 | 2.489558 | 0.032606 | |
| PTP4A2 | -1.39274 | 0.047478 | |
| REC8 | -0.43235 | 0.013018 | |
| TNNI3 | 6.044763 | 0.039053 | |
| SUZ12 | -3.88399 | 0.039053 | |
| IFI30 | 3.841074 | 0.033256 | |
| PLA2R1 | -6.7351 | 0.013364 | |
| ABHD3 | 5.967977 | 0.013364 | |
| EIF4G2 | -3.38804 | 0.001014 | |
| GRB2 | -0.77333 | 0.033632 | |
| ULBP3 | -0.81593 | 0.014869 | |
| SRPX | -3.70967 | 0.012101 | |
| LSM4 | 3.676069 | 0.04569 | |
| LSM7 | 5.77473 | 0.000201 | |
| POLR2D | -0.40751 | 0.003408 | |
| POLR2A | -3.2364 | 0.0267 | |
| MFSD7 | 1.438079 | 0.017902 | |
| POLR2B | -4.08916 | 0.00751 | |
| POLR2J | 2.471754 | 0.015908 | |
| TLK1 | -8.16451 | 0.0001 | |
| SLFN13 | -2.62889 | 0.048158 | |
| FAM120B | -4.24265 | 0.006852 | |
| MAPK14 | -5.30508 | 0.008504 | |
| CACNB1 | -1.19228 | 0.028559 | |
| C10orf75 | 0.802248 | 0.038783 | |
| ASH1L | -6.04606 | 0.009727 | |
| WDR8 | 2.873118 | 0.043371 | |
| USO1 | -1.59044 | 0.043642 | |
| PLCD1 | 5.348152 | 0.015792 | |
| HIST1H1C | 0.955704 | 0.017198 | |
| PICK1 | 0.350171 | 0.017788 | |
| NHP2 | 2.915417 | 0.023257 | |
| KIF20B | -2.25462 | 0.04775 | |
| MCC | -6.78965 | 0.004788 | |
| CAB39 | -4.8457 | 0.000707 | |
| ANKHD1 | -0.72034 | 0.024839 | |
| GNAT2 | -1.7881 | 0.026332 | |
| BCL2L11 | -0.99108 | 0.036951 | |
| BCL2L10 | 5.780532 | 0.043239 | |
| ESCO1 | -4.34485 | 0.024839 | |
| NUP43 | -4.18085 | 0.006413 | |
| IL1R1 | -7.61432 | 0.010176 | |
| ADRM1 | 8.708392 | 0.000605 | |
| RPL3 | 0.45495 | 0.026206 | |
| CHD1 | -1.40423 | 0.032863 | |
| ISYNA1 | 1.628209 | 0.013939 | |
| GTF2H3 | -6.1765 | 0.017082 | |
| GTF2H1 | -2.77169 | 0.042712 | |
| RPL36 | 5.466358 | 0.006413 | |
| RPL32 | 5.031729 | 0.030701 | |
| ATF7 | -3.44725 | 0.001219 | |
| ATF1 | -2.49482 | 0.03109 | |
| ATF2 | -2.84715 | 0.000605 | |
| LOC642852 | -3.12843 | 0.026576 | |
| CTTNBP2 | -3.5182 | 0.013018 | |
| SFRS13A | -2.20777 | 0.010849 | |
| KIAA0317 | -9.03878 | 0.005756 | |
| ANKIB1 | -0.8315 | 0.031845 | |
| BLOC1S1 | 0.483834 | 0.022291 | |
| SIVA1 | 0.89238 | 0.005542 | |
| MIB1 | -1.96544 | 0.016487 | |
| B3GALT5 | -5.32601 | 0.000912 | |
| B3GALT6 | 1.269949 | 0.040236 | |
| LEPROTL1 | 3.907496 | 0.013939 | |
| CTNNAL1 | -4.35891 | 0.041932 | |
| SNRNP25 | 5.522222 | 0.000605 | |
| APOC1 | 3.962792 | 0.005866 | |
| LOC641367 | -4.34474 | 0.007621 | |
| PTAR1 | -0.51772 | 0.032863 | |
| CHIC1 | -7.69792 | 0.018141 | |
| RAB3GAP1 | -3.44341 | 0.000201 | |
| SENP7 | -7.05227 | 0.001943 | |
| SENP1 | -4.20269 | 0.016372 | |
| ING5 | -1.76067 | 0.016254 | |
| ING2 | 5.319207 | 0.003725 | |
| ING3 | -0.90666 | 0.021092 | |
| ECHDC2 | 7.138139 | 0.001322 | |
| DAG1 | 5.096243 | 0.013595 | |
| ACP5 | 2.894692 | 0.010849 | |
| ACP6 | 4.322822 | 0.012216 | |
| SDCCAG1 | -3.91838 | 0.027562 | |
| ANKRD32 | -2.6013 | 0.024349 | |
| GLS | -0.33472 | 0.032229 | |
| CDIPT | 5.885429 | 0.002358 | |
| NCOA3 | -0.52076 | 0.024966 | |
| LCOR | -4.79068 | 0.024719 | |
| MECR | 4.6223 | 0.015562 | |
| BSG | 4.016775 | 0.016845 | |
| YTHDC2 | -4.4767 | 0.021449 | |
| NSFL1C | 2.546687 | 0.045554 | |
| PRDM15 | -1.04925 | 0.027692 | |
| SLC27A3 | 4.251836 | 0.001943 | |
| ERO1L | -0.98405 | 0.025219 | |
| ZNF638 | -2.09332 | 0.006413 | |
| AK1 | 0.695784 | 0.023744 | |
| TBCB | 3.788729 | 0.016964 | |
| ZNF474 | -1.22941 | 0.031477 | |
| OXTR | -0.40735 | 0.001014 | |
| SMARCAD1 | -1.98628 | 0.039434 | |
| OR1N1 | 21.2831 | 0.01568 | |
| ZNF493 | -5.7731 | 0.04925 | |
| EPS8 | -9.98242 | 0.001322 | |
| RRP8 | -4.64416 | 0.022418 | |
| WDR89 | -1.87777 | 0.028195 | |
| FCGRT | 6.129567 | 0.003091 | |
| SLC24A6 | 3.74725 | 0.000402 | |
| MDC1 | -2.14961 | 0.044336 | |
| RNF126 | 1.292263 | 0.02882 | |
| BRMS1L | -4.05111 | 0.001943 | |
| MEX3D | 5.812542 | 0.008172 | |
| SFTPD | 1.369455 | 0.006962 | |
| FRYL | -4.95899 | 0.007399 | |
| SFTPB | 13.13366 | 0.017552 | |
| MAP9 | -10.1492 | 0.002255 | |
| UBXN10 | -8.51815 | 0.038003 | |
| DNTTIP1 | 6.745853 | 0.014057 | |
| UBLCP1 | -11.7756 | 0.000201 | |
| DYM | -1.13735 | 0.045821 | |
| MTMR9 | -2.61679 | 0.033758 | |
| MTMR6 | -5.68869 | 0.017437 | |
| YIPF2 | 5.878323 | 0.005866 | |
| TSC22D4 | 3.603466 | 0.016025 | |
| FCHSD2 | -5.21936 | 0.012333 | |
| HIGD2B | 8.543758 | 0.029822 | |
| CD2BP2 | 2.506994 | 0.017318 | |
| NDUFC1 | 6.46059 | 0.036699 | |
| BCKDK | 3.005892 | 0.013134 | |
| EXOSC7 | 0.662812 | 0.026206 | |
| EXOSC4 | 10.92304 | 0.000912 | |
| EXOSC5 | 6.197384 | 0.002358 | |
| SURF1 | 1.475408 | 0.038003 | |
| TULP4 | -3.41174 | 0.005326 | |
| FARP2 | -3.40928 | 0.019444 | |
| WDR36 | -2.77281 | 0.024349 | |
| BTG4 | 14.93619 | 0.04569 | |
| BTG1 | 8.089387 | 0.01452 | |
| PSMB10 | 2.842927 | 0.004149 | |
| SCYL2 | -2.61761 | 0.024349 | |
| STXBP2 | 0.17135 | 0.014175 | |
| STXBP5 | -4.28266 | 0.000201 | |
| SMOC2 | -2.98638 | 0.0472 | |
| FANCM | -3.46698 | 0.036198 | |
| KIAA1671 | -1.75652 | 0.005866 | |
| GLYATL1 | -4.49639 | 0.008282 | |
| ARSB | -0.8514 | 0.04379 | |
| LRRFIP2 | 3.470963 | 0.047478 | |
| MYST1 | 4.284839 | 0.031845 | |
| EXOC5 | -5.78927 | 0.000809 | |
| IRS1 | -4.79343 | 0.04297 | |
| CDK4 | 6.169637 | 0.041419 | |
| CDK8 | -4.52296 | 0.030958 | |
| LPAR1 | -10.9308 | 0.001425 | |
| ZFHX3 | -11.8327 | 0.000301 | |
| OGN | -8.30225 | 0.005542 | |
| MTX3 | -4.00929 | 0.022418 | |
| PRKCI | -0.95344 | 0.024839 | |
| ATP10D | -6.57522 | 0.006302 | |
| TNNC2 | 5.525797 | 0.037339 | |
| HIST1H3F | 6.060789 | 0.016372 | |
| HIST1H3G | 8.306198 | 0.009498 | |
| PMF1 | 6.551563 | 0.001219 | |
| SCRIB | 5.324798 | 0.001943 | |
| TYMP | 0.377075 | 0.028559 | |
| RIF1 | -4.7792 | 0.003198 | |
| ZNF790 | -5.0898 | 0.003831 | |
| ZNF791 | -0.88392 | 0.04925 | |
| WWP2 | 5.850641 | 0.0235 | |
| DAPL1 | 7.637863 | 0.044861 | |
| PPIL4 | -12.153 | 0.000301 | |
| MYADM | -5.85846 | 0.002985 | |
| CCDC99 | -0.85577 | 0.036066 | |
| CCDC94 | 2.376647 | 0.017082 | |
| CCDC91 | -4.90848 | 0.020502 | |
| CTSA | 7.8031 | 0.002462 | |
| CTSF | 4.882063 | 0.040879 | |
| RPL10 | 5.213993 | 0.004255 | |
| RPL11 | 4.202289 | 0.01452 | |
| GPATCH8 | -4.17664 | 0.048566 | |
| RPL13 | 3.933792 | 0.027562 | |
| PRKAR2B | -6.46413 | 0.019205 | |
| NAA10 | 3.938012 | 0.001322 | |
| VPS4A | 4.554541 | 0.032102 | |
| SORBS3 | 2.22177 | 0.005974 | |
| METT10D | -4.99338 | 0.001528 | |
| ZGPAT | 3.425881 | 0.001116 | |
| USP28 | -1.39715 | 0.030452 | |
| SORBS2 | -4.60053 | 0.044466 | |
| MBL1P | -0.0763 | 0.041143 | |
| AFF4 | -5.4228 | 0.001425 | |
| AFF1 | -3.47944 | 0.005866 | |
| ALDH16A1 | 0.694573 | 0.008724 | |
| SAE1 | 2.516215 | 0.02509 | |
| ERP27 | 2.046198 | 0.04775 | |
| ERP29 | 4.536563 | 0.001735 | |
| UBA6 | -2.52233 | 0.025472 | |
| PDP1 | -2.48837 | 0.014752 | |
| TRRAP | -6.16383 | 0.004255 | |
| LOC283174 | -4.23781 | 0.033256 | |
| UBE2C | 0.386272 | 0.028689 | |
| UBE2Z | 9.086243 | 0.025717 | |
| UBE2W | -4.46099 | 0.000402 | |
| SCG2 | -2.14393 | 0.038658 | |
| TIMM44 | 2.405274 | 0.006742 | |
| PNO1 | -0.20199 | 0.033506 | |
| PDE3A | -0.79724 | 0.01029 | |
| BMP2K | -0.287 | 0.018141 | |
| C20orf134 | 3.125781 | 0.002255 | |
| RABEP2 | 1.466984 | 0.031723 | |
| NDUFA6 | 6.820527 | 0.014175 | |
| NDUFA4 | 6.245618 | 0.019686 | |
| C21orf70 | 4.229173 | 0.004149 | |
| NDUFA8 | 3.780703 | 0.005004 | |
| PLGLB2 | -4.79482 | 0.01568 | |
| BIRC6 | -3.77252 | 0.001425 | |
| AIP | 2.627886 | 0.020733 | |
| EIF6 | 3.627401 | 0.011752 | |
| LASS4 | 4.293537 | 0.012216 | |
| ANKRD19 | 3.210873 | 0.01029 | |
| ANKRD17 | -2.10989 | 0.039974 | |
| ANKRD12 | -5.5835 | 0.032102 | |
| POLR2G | 2.508556 | 0.030069 | |
| TMEM37 | 0.852496 | 0.031978 | |
| PDPK1 | -9.50682 | 0.0001 | |
| DMC1 | 1.210293 | 0.043239 | |
| NT5C2 | -1.68447 | 0.005326 | |
| MEA1 | 5.707842 | 0.018616 | |
| REP15 | -6.40175 | 0.005542 | |
| HAGHL | 0.285358 | 0.01279 | |
| FAM53C | -4.72916 | 0.000605 | |
| FAM53A | 1.347286 | 0.000605 | |
| ACRC | -1.97067 | 0.049407 | |
| PRUNE2 | -0.45724 | 0.03517 | |
| PARG | -4.33706 | 0.008504 | |
| PEX11G | 0.721919 | 0.014869 | |
| MLH3 | -5.44496 | 0.009841 | |
| HIBADH | 5.397929 | 0.029822 | |
| NHLRC2 | -7.06926 | 0.003198 | |
| PAFAH1B1 | -0.51009 | 0.014175 | |
| POLR2F | 5.25787 | 0.005217 | |
| VAMP1 | -3.53948 | 0.043642 | |
| SEC23IP | -2.94856 | 0.025339 | |
| DOHH | 4.879745 | 0.000201 | |
| SNRNP200 | -1.88775 | 0.00917 | |
| MEPCE | 5.972774 | 0.001631 | |
| DDX5 | -0.13662 | 0.010064 | |
| DDX6 | -1.2186 | 0.032739 | |
| MYO15A | -1.99658 | 0.034782 | |
| NUP205 | -2.45709 | 0.018489 | |
| HLA-H | 1.494534 | 0.017669 | |
| F8A1 | 9.997335 | 0.006192 | |
| HLA-A | 2.765851 | 0.014637 | |
| ARID4A | -6.30116 | 0.006852 | |
| ARID4B | -5.97336 | 0.008724 | |
| HAUS6 | -0.27677 | 0.007621 | |
| HAUS4 | 1.486629 | 0.008394 | |
| HAUS3 | -3.89674 | 0.007178 | |
| DLX5 | 10.00849 | 0.0151 | |
| TFEC | -1.35329 | 0.02882 | |
| ZNF805 | -0.03428 | 0.016372 | |
| TMEM160 | 3.287231 | 0.000912 | |
| TNFAIP2 | 4.388245 | 0.041419 | |
| IPO8 | -2.44726 | 0.006852 | |
| IPO7 | -6.65129 | 0.002047 | |
| NRBP1 | 4.181526 | 0.048425 | |
| RAB8A | 4.113043 | 0.004363 | |
| C6orf165 | -3.06043 | 0.0151 | |
| PAQR3 | -4.24334 | 0.025963 | |
| NME2P1 | 7.320414 | 0.001943 | |
| SIL1 | 3.97857 | 0.008834 | |
| HCG18 | -2.19242 | 0.024349 | |
| PMVK | 2.132173 | 0.021807 | |
| AWAT1 | 14.25548 | 0.046364 | |
| SAMD8 | -2.108 | 0.000402 | |
| C5orf22 | -2.43808 | 0.014637 | |
| C5orf24 | -9.42362 | 0.000912 | |
| GRIN2A | -2.02584 | 0.033632 | |
| C9orf66 | -7.13759 | 0.027312 | |
| C9orf68 | -6.70628 | 0.003198 | |
| C9orf69 | 0.846785 | 0.042586 | |
| UQCR10 | 6.636622 | 0.006523 | |
| KLHDC8B | 3.270451 | 0.000605 | |
| USP16 | -0.97383 | 0.006084 | |
| UNC45A | 4.950869 | 0.009841 | |
| ZCCHC12 | -2.19262 | 0.021209 | |
| JPH1 | -8.8299 | 0.001528 | |
| C11orf75 | 5.492959 | 0.030452 | |
| TYK2 | 2.836908 | 0.00751 | |
| TMEM87B | -3.91891 | 0.034392 | |
| ARHGEF12 | -1.86846 | 0.035674 | |
| PAPPA | -2.71444 | 0.046926 | |
| NEGR1 | -3.34285 | 0.03109 | |
| CYP4X1 | -6.15499 | 0.007287 | |
| TNKS2 | -5.38164 | 0.013249 | |
| CEP76 | -3.08133 | 0.013709 | |
| RHBDD1 | -7.92825 | 0.003198 | |
| ZNF776 | -4.06269 | 0.017669 | |
| ZNF770 | -1.36742 | 0.024349 | |
| FBXW11 | -12.8472 | 0.0001 | |
| CCDC142 | -5.60634 | 0.029201 | |
| OR2W3 | -1.26484 | 0.016723 | |
| CHCHD3 | 5.236865 | 0.00917 | |
| CHCHD5 | 2.369912 | 0.018141 | |
| CHCHD8 | 2.034727 | 0.022539 | |
| RFK | -4.52304 | 0.01029 | |
| GDF15 | -3.02259 | 0.017669 | |
| GDF11 | -3.76524 | 0.005217 | |
| DLG1 | -1.95776 | 0.032739 | |
| TMTC1 | -1.56245 | 0.015562 | |
| SLC39A3 | 7.786752 | 0.001116 | |
| WDR18 | 5.439039 | 0.0001 | |
| PMS2L3 | -5.20038 | 0.012101 | |
| ZNF845 | -5.00433 | 0.017788 | |
| LOC647288 | -0.70811 | 0.032863 | |
| ZNF28 | -2.68756 | 0.003514 | |
| GBF1 | -2.3693 | 0.035546 | |
| ZC3H3 | 7.3891 | 0.000402 | |
| JTB | 6.825995 | 0.003831 | |
| DNM1P35 | 2.656466 | 0.022291 | |
| PRPH2 | -0.19081 | 0.01992 | |
| ZMYND17 | -3.34414 | 0.011302 | |
| ZDHHC20 | -5.20217 | 0.004255 | |
| NAA35 | -2.99347 | 0.008172 | |
| NAA30 | -10.4773 | 0.008504 | |
| RPS10P7 | -4.13059 | 0.018489 | |
| OCEL1 | 4.83659 | 0.000809 | |
| LOC283314 | -4.26128 | 0.012216 | |
| GMCL1 | -6.20188 | 0.001322 | |
| AP1S1 | 2.950768 | 0.041419 | |
| AP1S3 | -4.2694 | 0.000201 | |
| NDE1 | 4.533538 | 0.012216 | |
| CAD | 4.705948 | 0.044206 | |
| C20orf118 | -2.51683 | 0.033506 | |
| LOC150786 | -6.34546 | 0.001219 | |
| C20orf112 | -6.05469 | 0.0316 | |
| C12orf62 | 1.668704 | 0.008945 | |
| C12orf65 | 3.824151 | 0.013824 | |
| ARMCX1 | -3.20719 | 0.024596 | |
| PTMA | 2.910969 | 0.007621 | |
| GHR | -5.83543 | 0.012101 | |
| DYNLT1 | -2.79406 | 0.013134 | |
| NIPBL | -5.38749 | 0.001943 | |
| DYNLT3 | -1.30477 | 0.038396 | |
| STRAP | -1.09862 | 0.002358 | |
| C1orf85 | 4.705816 | 0.007287 | |
| ZNF234 | -7.56302 | 0.002776 | |
| TOMM7 | 5.302712 | 0.026087 | |
| SIGLEC11 | -0.15534 | 0.037339 | |
| GTF2F1 | 1.106856 | 0.003302 | |
| KIFC2 | 3.615874 | 0.002985 | |
| KIFC3 | 1.248425 | 0.030199 | |
| C10orf125 | 1.500108 | 0.027692 | |
| SFN | 1.630919 | 0.018141 | |
| EID3 | -1.98421 | 0.042836 | |
| PPIP5K2 | -4.79234 | 0.006631 | |
| ADAR | -0.6419 | 0.0397 | |
| NRGN | 1.957764 | 0.030701 | |
| ZNF185 | 1.442096 | 0.034264 | |
| ZNF184 | -2.83669 | 0.014404 | |
| ZNF181 | -2.21499 | 0.027562 | |
| ZNF180 | -3.33661 | 0.026332 | |
| ZNF182 | -8.64801 | 0.027184 | |
| TRA2B | -2.38109 | 0.006192 | |
| ZNF25 | -7.45797 | 0.000707 | |
| INTS10 | 1.228771 | 0.041273 | |
| ZNF22 | -15.8258 | 0.000301 | |
| BLVRB | 5.096134 | 0.000402 | |
| UBL4A | 0.880712 | 0.042323 | |
| TCERG1 | -3.27152 | 0.021449 | |
| SENP6 | -3.186 | 0.012446 | |
| SEMA4G | -3.77142 | 0.016723 | |
| FAM35B | -4.45264 | 0.001425 | |
| FAM35A | -5.3847 | 0.001014 | |
| ZNF827 | -6.73518 | 0.029822 | |
| RANBP9 | -1.43323 | 0.026818 | |
| RANBP3 | 1.500334 | 0.011641 | |
| RANBP2 | -2.22807 | 0.004363 | |
| ZNF131 | -2.09046 | 0.000301 | |
| TXN2 | 3.030411 | 0.019323 | |
| AGAP1 | -2.37624 | 0.00784 | |
| AGAP5 | -5.49669 | 0.003198 | |
| TSPO | 5.19551 | 0.039053 | |
| RANBP6 | -3.96921 | 0.00784 | |
| BASP1 | -3.53092 | 0.027056 | |
| GDPD1 | -6.86088 | 0.005326 | |
| TBCEL | -3.297 | 0.011867 | |
| C17orf90 | 3.47751 | 0.002671 | |
| HOXD4 | -2.27704 | 0.003514 | |
| UIMC1 | -6.4715 | 0.020976 | |
| HDGFRP2 | 2.248597 | 0.000301 | |
| ZDHHC24 | 4.684957 | 0.004788 | |
| HTR1D | 3.882504 | 0.036066 | |
| KIAA0652 | -3.16865 | 0.014637 | |
| CHST12 | 1.450903 | 0.021329 | |
| CHST15 | -9.56616 | 0.032739 | |
| CHST14 | 2.021937 | 0.026576 | |
| HTR1B | -1.24356 | 0.04775 | |
| MMP2 | -3.81696 | 0.019801 | |
| GXYLT1 | -0.5319 | 0.031221 | |
| TRAPPC2L | 4.165156 | 0.010625 | |
| AGER | -3.89907 | 0.046509 | |
| CAPRIN1 | -3.28585 | 0.013134 | |
| CCNT2 | -4.16233 | 0.00806 | |
| CREBZF | -7.27219 | 0.000503 | |
| C9orf86 | 0.697749 | 0.005974 | |
| ZMYM6 | -1.63232 | 0.03645 | |
| C9orf80 | -9.42512 | 0.002567 | |
| C9orf82 | -5.1856 | 0.001631 | |
| WNK3 | -0.84901 | 0.020039 | |
| NR2C2 | -6.73642 | 0.010964 | |
| LOC134466 | -1.66301 | 0.043928 | |
| RFNG | 1.885113 | 0.008282 | |
| USP34 | -1.74612 | 0.002151 | |
| SHMT1 | 2.935526 | 0.012333 | |
| USP37 | -2.06914 | 0.035295 | |
| ST20 | 6.937452 | 0.000301 | |
| PPFIBP1 | -5.83791 | 0.044736 | |
| RPL23AP64 | -5.78405 | 0.001014 | |
| RRP1 | 5.353609 | 0.020157 | |
| MRPL19 | -0.95354 | 0.0235 | |
| DOCK4 | -3.05555 | 0.032229 | |
| NCRNA00095 | -4.78655 | 0.004363 | |
| FLJ41941 | 12.24412 | 0.040747 | |
| LRRC66 | -0.22001 | 0.011867 | |
| SLC9A2 | -4.5288 | 0.024719 | |
| FAM98C | 5.424799 | 0.001219 | |
| CYP4Z1 | -3.29904 | 0.006962 | |
| SPINT3 | 18.6993 | 0.044065 | |
| TWF2 | 1.446699 | 0.006084 | |
| C11orf58 | -8.09462 | 0.013018 | |
| JMJD7-PLA2G4B | 0.866635 | 0.024349 | |
| RPSAP52 | -2.47712 | 0.038527 | |
| CTNND2 | -5.50685 | 0.04526 | |
| C18orf22 | 1.03524 | 0.006742 | |
| TRUB1 | -9.34994 | 0.000605 | |
| TRUB2 | 3.158139 | 0.012676 | |
| CSNK1A1 | -3.59986 | 0.016487 | |
| SASS6 | -5.88593 | 0.003302 | |
| ATL3 | -2.05227 | 0.024223 | |
| CCDC85B | 3.027273 | 0.006852 | |
| CORO1B | 4.557832 | 0.030069 | |
| PIN1 | 2.184125 | 0.000707 | |
| LOC338651 | -7.85563 | 0.009727 | |
| RPGRIP1L | -4.21882 | 0.003408 | |
| FAU | 1.496103 | 0.023744 | |
| HNRNPA2B1 | 3.296614 | 0.035045 | |
| TRIM9 | -1.65123 | 0.01802 | |
| TTC36 | 1.609515 | 0.001219 | |
| DCP2 | -1.80412 | 0.048026 | |
| AMY2A | 13.49423 | 0.00806 | |
| PIP5K1C | 2.772582 | 0.01029 | |
| LOC441454 | -3.69136 | 0.013824 | |
| NCAPH2 | 3.526768 | 0.031978 | |
| C6orf108 | 2.44955 | 0.002985 | |
| CFDP1 | 1.309202 | 0.037874 | |
| TSPAN6 | -5.72524 | 0.029201 | |
| C14orf142 | 3.536659 | 0.045554 | |
| ZMYM5 | -6.81833 | 0.002151 | |
| C14orf147 | -2.58245 | 0.003725 | |
| HECTD1 | -4.32334 | 0.000503 | |
| MAN2B2 | 1.331595 | 0.032739 | |
| TNFRSF14 | 0.814739 | 0.037211 | |
| CCS | 3.325241 | 0.011187 | |
| NKX6-1 | 5.43235 | 0.049407 | |
| KCTD13 | 3.909981 | 0.045966 | |
| C12orf49 | -5.41653 | 0.001219 | |
| IGHMBP2 | 8.230931 | 0.014869 | |
| NFAT5 | -1.95215 | 0.030701 | |
| PNCK | -0.75164 | 0.041806 | |
| UBB | 6.955487 | 0.006413 | |
| ANKRD52 | -6.75119 | 0.006084 | |
| ANKRD58 | 1.677299 | 0.001425 | |
| MAT2A | -0.37478 | 0.045554 | |
| HEG1 | -3.78951 | 0.04761 | |
| KBTBD4 | -6.77553 | 0.043928 | |
| AMN | 12.95914 | 0.031978 | |
| SSNA1 | 2.237857 | 0.002358 | |
| GUCY1A2 | -1.16917 | 0.020976 | |
| MYST2 | 7.016538 | 0.016602 | |
| MED30 | 5.009325 | 0.042452 | |
| NHSL2 | -4.73037 | 0.016964 | |
| RSRC1 | -1.18621 | 0.015908 | |
| SERAC1 | -5.80419 | 0.001839 | |
| PHAX | -6.24951 | 0.002151 | |
| MGAT2 | -3.45739 | 0.04297 | |
| RBM26 | -2.07811 | 0.011641 | |
| RBM27 | -8.30428 | 0.000201 | |
| RBM25 | -3.26699 | 0.001425 | |
| EPHA8 | 7.305217 | 0.011073 | |
| ZMYM1 | -2.79181 | 0.010515 | |
| KDM6A | -7.2 | 0.001943 | |
| FGF13 | -7.81349 | 0.049111 | |
| SEMA3D | -4.00436 | 0.014869 | |
| STYXL1 | 2.624966 | 0.013595 | |
| TNFRSF18 | 1.419826 | 0.006084 | |
| RAPGEF5 | -0.59537 | 0.027692 | |
| ARL9 | -1.53312 | 0.003938 | |
| ARL2 | 1.550798 | 0.018616 | |
| ARL6 | -7.30658 | 0.004363 | |
| RRM1 | -0.99434 | 0.038783 | |
| LOC399959 | -1.80429 | 0.037211 | |
| KCNJ13 | -1.11888 | 0.006084 | |
| TFAM | -5.46657 | 0.001631 | |
| FAM35B2 | -3.00461 | 0.002462 | |
| TMX1 | -4.01352 | 0.047335 | |
| TPTE2P1 | -1.91779 | 0.001631 | |
| C1QTNF7 | -3.85723 | 0.019323 | |
| CREB3L3 | 7.148035 | 0.038658 | |
| VDAC3 | 4.671901 | 0.032994 | |
| C17orf79 | 0.460382 | 0.041273 | |
| MFAP3 | -8.73015 | 0.012333 | |
| C6orf120 | -3.12241 | 0.012333 | |
| VAC14 | 7.15369 | 0.002255 | |
| LOC646851 | -1.01535 | 0.04775 | |
| NDUFB2 | 6.358224 | 0.029693 | |
| APRT | 3.072926 | 0.034651 | |
| ZFYVE20 | -0.78166 | 0.005435 | |
| C19orf53 | 6.468575 | 0.002985 | |
| C19orf52 | 7.190355 | 0.002985 | |
| C19orf55 | -1.88902 | 0.024719 | |
| C19orf54 | 4.202011 | 0.014752 | |
| C11orf45 | 2.801546 | 0.048425 | |
| C11orf46 | -6.72866 | 0.003514 | |
| PUM2 | -1.39573 | 0.043642 | |
| RAD1 | -3.78231 | 0.002985 | |
| SFXN1 | -2.57768 | 0.018616 | |
| CCT7 | 3.315436 | 0.02509 | |
| TIMM16 | 3.154248 | 0.029943 | |
| TIMM10 | 5.821998 | 0.005974 | |
| ARL13B | -9.28165 | 0.003408 | |
| SPOPL | -5.29294 | 0.019686 | |
| BMS1P5 | -3.29946 | 0.042586 | |
| BMS1P4 | -8.12489 | 0.006523 | |
| FAM50A | 0.880269 | 0.001219 | |
| ZNF654 | -4.6971 | 0.000402 | |
| 2-Mar | 1.591454 | 0.010402 | |
| UHMK1 | -3.9585 | 0.013595 | |
| THOC2 | -6.02966 | 0.020271 | |
| SLC35F5 | -3.94651 | 0.004149 | |
| SLC4A3 | -11.1415 | 0.00468 | |
| DCTN3 | 1.359718 | 0.044206 | |
| DCTN6 | 2.404523 | 0.025472 | |
| SLC4A8 | -6.4762 | 0.032356 | |
| ASPA | -1.31519 | 0.023381 | |
| KRBA2 | -1.55323 | 0.0267 | |
| ASPH | -5.84519 | 0.016487 | |
| ZC3HAV1 | -4.20608 | 0.001322 | |
| VANGL1 | -2.25203 | 0.021329 | |
| PREP | -0.5535 | 0.004363 | |
| SSTR1 | -1.70147 | 0.01256 | |
| C10orf88 | -4.19694 | 0.023257 | |
| SLC43A1 | 2.459694 | 0.013479 | |
| TMEM203 | 4.677745 | 0.009951 | |
| ANKRD45 | -0.59882 | 0.019444 | |
| ANKRD44 | -6.33611 | 0.024596 | |
| CTNNB1 | -6.09927 | 0.022171 | |
| TMEM50A | 3.965799 | 0.032606 | |
| HMOX2 | 3.263105 | 0.009059 | |
| C18orf45 | -2.80916 | 0.04569 | |
| CNOT8 | -3.1007 | 0.00806 | |
| CNOT1 | -3.13035 | 0.036323 | |
| ITGB1 | -6.06195 | 0.005974 | |
| ITGB6 | -8.96807 | 0.006413 | |
| ATN1 | 2.203459 | 0.028074 | |
| SCXB | 13.26848 | 0.000809 | |
| TNIP2 | 0.290876 | 0.001322 | |
| MYO6 | -1.41704 | 0.045119 | |
| MAN1B1 | 2.577773 | 0.024103 | |
| URGCP | 1.017105 | 0.017669 | |
| USP35 | -3.04037 | 0.042069 | |
| IMP3 | 6.503329 | 0.005326 | |
| MRVI1 | -1.45752 | 0.034132 | |
| RBM12B | -2.67464 | 0.024966 | |
| ZAK | -6.63566 | 0.006192 | |
| PSMC3 | 1.836169 | 0.016845 | |
| LOC645332 | 7.359405 | 0.006084 | |
| FAM164A | -5.45458 | 0.001528 | |
| C14orf162 | 3.718109 | 0.003408 | |
| SNX6 | -8.80751 | 0.000301 | |
| CCDC124 | 2.849359 | 0.000605 | |
| CCDC123 | -0.98015 | 0.042586 | |
| SNHG7 | 1.173519 | 0.0267 | |
| ITPKA | 9.128191 | 0.001839 | |
| MGEA5 | -0.90713 | 0.031477 | |
| PHKG2 | 4.501122 | 0.006742 | |
| NRTN | 9.414744 | 0.013134 | |
| ITPK1 | 0.882452 | 0.01452 | |
| C12orf29 | -0.8984 | 0.045404 | |
| ZNF343 | -4.42732 | 0.027056 | |
| TUBB2C | 3.322263 | 0.001735 | |
| RNASEH2A | 4.85168 | 0.017318 | |
| NEIL2 | 3.996218 | 0.01802 | |
| MUT | -4.20679 | 0.018854 | |
| CHAD | 2.556521 | 0.011752 | |
| AHI1 | -2.18936 | 0.004788 | |
| AKR1C1 | 6.199258 | 0.029693 | |
| ZNF771 | 6.066212 | 0.007621 | |
| SPDYC | 8.213029 | 0.028074 | |
| WHAMML2 | -5.47432 | 0.003514 | |
| GRIA3 | -2.09086 | 0.011418 | |
| HNRNPL | 6.092902 | 0.016845 | |
| ATP6V0C | 2.703899 | 0.029074 | |
| CENPM | 2.355629 | 0.030323 | |
| NAT15 | 5.033488 | 0.007178 | |
| HNRNPD | 0.430913 | 0.034918 | |
| ALDH2 | 2.559106 | 0.002985 | |
| FAM55C | -5.27246 | 0.027825 | |
| C18orf1 | -10.1599 | 0.005217 | |
| NPFF | -2.26979 | 0.010625 | |
| UQCR11 | 6.83115 | 0.000402 | |
| CXorf38 | -1.85414 | 0.018854 | |
| OPRL1 | 1.945547 | 0.04526 | |
| ASAH1 | 4.909871 | 0.021571 | |
| PRR3 | -3.10202 | 0.01568 | |
| PRR5 | 2.397975 | 0.006962 | |
| DVL2 | 0.037782 | 0.011867 | |
| ABCB9 | 1.070676 | 0.023983 | |
| JSRP1 | 6.785299 | 0.019088 | |
| PLEKHA1 | -9.16962 | 0.000503 | |
| MTSS1 | 3.579072 | 0.029943 | |
| TOPBP1 | -0.54821 | 0.037615 | |
| RSPRY1 | -5.11449 | 0.04775 | |
| DNLZ | 1.621209 | 0.00751 | |
| LIN7C | -4.67509 | 0.008172 | |
| SLC30A4 | -2.86347 | 0.020157 | |
| SLC30A7 | -0.32636 | 0.005004 | |
| SLC30A6 | -2.1426 | 0.022418 | |
| EML1 | -7.47172 | 0.009727 | |
| ZYG11B | -1.531 | 0.019686 | |
| SPEF2 | -7.28006 | 0.017198 | |
| SFRS3 | -2.29924 | 0.005756 | |
| NUPL1 | -3.81808 | 0.01279 | |
| NUPL2 | -6.0688 | 0.008615 | |
| RSPO1 | -4.31524 | 0.018975 | |
| DHODH | 3.178342 | 0.044991 | |
| ZNF275 | -4.02267 | 0.034918 | |
| KCNV1 | 12.32558 | 0.048849 | |
| FAM98B | -4.04804 | 0.027949 | |
| XKR8 | 2.933408 | 0.010849 | |
| CIAO1 | -3.22473 | 0.022171 | |
| OR2A9P | 2.714359 | 0.04526 | |
| STK35 | -1.96703 | 0.027562 | |
| MAML1 | -2.59208 | 0.025717 | |
| TUBB8 | 0.644597 | 0.012446 | |
| C19orf70 | 6.230515 | 0.000707 | |
| APOLD1 | -1.35101 | 0.045404 | |
| SH3BGRL3 | 4.933573 | 0.043102 | |
| C17orf59 | 0.56046 | 0.04297 | |
| C9orf16 | 6.980214 | 0.0001 | |
| LCLAT1 | -0.41681 | 0.01568 | |
| CD80 | -2.85495 | 0.031345 | |
| ABCC6P2 | 4.795863 | 0.009498 | |
| LOC284441 | -2.98748 | 0.005004 | |
| BCL7C | 2.210333 | 0.01992 | |
| ZBED1 | -3.1909 | 0.0397 | |
| RPE | -3.59244 | 0.019444 | |
| ZNF562 | -6.4282 | 0.000201 | |
| LRP2 | -8.64557 | 0.010849 | |
| GALNT10 | -9.96306 | 0.037748 | |
| SSH2 | -0.06134 | 0.041557 | |
| LOC554202 | -0.46103 | 0.01279 | |
| S100P | 1.098858 | 0.03109 | |
| SSH3 | 5.41238 | 0.010064 | |
| MAP2 | -1.41386 | 0.036951 | |
| C8G | 8.240115 | 0.013364 | |
| PLEKHF2 | -2.94484 | 0.012216 | |
| APEX2 | 6.936412 | 0.016487 | |
| RPL37A | 4.118065 | 0.03957 | |
| SERPINI1 | -0.55787 | 0.023983 | |
| ESRRA | 1.223809 | 0.018975 | |
| TMEM208 | 2.946533 | 0.023136 | |
| ZNF718 | -2.11639 | 0.031345 | |
| EBPL | 3.198841 | 0.0472 | |
| ZNF713 | -4.44755 | 0.049546 | |
| NME1-NME2 | 0.351704 | 0.024596 | |
| ZFP2 | -11.5054 | 0.003725 | |
| TLE2 | 4.903544 | 0.007399 | |
| PTBP2 | -0.89027 | 0.016964 | |
| FAM19A2 | -1.71604 | 0.022044 | |
| ZBTB6 | -4.01683 | 0.017437 | |
| GPR160 | -6.12252 | 0.028074 | |
| TBX1 | -2.45752 | 0.0397 | |
| ZWILCH | -2.0525 | 0.034132 | |
| NR1I2 | 8.805672 | 0.028314 | |
| FBXO5 | -4.17337 | 0.003514 | |
| FBXO6 | 4.586586 | 0.00751 | |
| MACF1 | -3.83157 | 0.009388 | |
| PHLPP2 | -3.80582 | 0.011867 | |
| FAM83H | 4.413459 | 0.014637 | |
| PTPRN2 | -0.37933 | 0.016025 | |
| TIAL1 | -2.78788 | 0.006631 | |
| NAIP | -1.82609 | 0.037748 | |
| HACE1 | -0.21378 | 0.02882 | |
| HEXIM1 | 1.789025 | 0.012676 | |
| DENND5A | -11.6453 | 0.000605 | |
| HEXIM2 | 4.328415 | 0.000912 | |
| C14orf105 | 13.09111 | 0.040236 | |
| C14orf106 | -3.38441 | 0.005326 | |
| C14orf109 | 2.944919 | 0.017788 | |
| SMEK2 | -0.17817 | 0.036066 | |
| IDS | -4.06865 | 0.00917 | |
| RRS1 | 3.266271 | 0.033256 | |
| CCDC101 | 8.224867 | 0.002358 | |
| CCDC107 | 1.089162 | 0.007399 | |
| LOC641298 | -6.95266 | 0.029567 | |
| POLD4 | 5.733277 | 0.033378 | |
| REEP3 | -2.2817 | 0.020976 | |
| ARMCX3 | -9.42922 | 0.000912 | |
| REEP6 | 0.072633 | 0.002151 | |
| FUBP1 | -8.94814 | 0.003938 | |
| ARL6IP4 | 0.188966 | 0.001425 | |
| ARMCX2 | -5.38175 | 0.033256 | |
| USF2 | 2.372005 | 0.001116 | |
| DICER1 | -3.4389 | 0.005435 | |
| AARS | 5.867397 | 0.024103 | |
| NCRNA00032 | 14.31681 | 0.04379 | |
| UBOX5 | -2.80728 | 0.003938 | |
| ANKRD2 | 6.733088 | 0.048026 | |
| ANKRD9 | 3.88248 | 0.002567 | |
| YME1L1 | -0.13756 | 0.000605 | |
| HNRNPA1L2 | 0.421287 | 0.0472 | |
| TSGA14 | -4.73933 | 0.029201 | |
| GTF2E2 | 6.411798 | 0.046926 | |
| RCC1 | 0.28291 | 0.019205 | |
| SPATA6 | -6.88727 | 0.011302 | |
| DUOXA2 | 7.661815 | 0.028945 | |
| SFRS2IP | -4.3979 | 0.003198 | |
| DRAP1 | 2.762678 | 0.006852 | |
| ABCD1 | 5.907778 | 0.001839 | |
| ABCD3 | -4.38251 | 0.032356 | |
| C2CD3 | -0.79093 | 0.018854 | |
| TOM1 | 2.755615 | 0.004575 | |
| LIN52 | -6.17981 | 0.021925 | |
| TRAPPC5 | 4.790294 | 0.001631 | |
| PPAN-P2RY11 | -11.8489 | 0.002567 | |
| SIM2 | -1.26164 | 0.04706 | |
| SBSN | 10.0934 | 0.04379 | |
| ST6GALNAC3 | -1.73975 | 0.026332 | |
| DGAT1 | 4.371202 | 0.002151 | |
| TNFRSF21 | -1.84501 | 0.03109 | |
| MTCP1 | 1.909414 | 0.033879 | |
| TBC1D10B | 7.855995 | 0.023381 | |
| TIMM17B | 4.533367 | 0.005326 | |
| SLC35D3 | -0.07376 | 0.001322 | |
| C20orf43 | 9.331553 | 0.020157 | |
| PPIB | 8.630443 | 0.01029 | |
| KIAA0586 | -3.25723 | 0.004575 | |
| WBSCR22 | 3.207306 | 0.024966 | |
| GDAP1 | -1.55502 | 0.010402 | |
| ZSWIM6 | -3.66268 | 0.003938 | |
| ZDHHC21 | -5.445 | 0.000503 | |
| DNALI1 | -7.82997 | 0.032994 | |
| ZSWIM1 | -3.29767 | 0.047478 | |
| NOL8 | -4.84415 | 0.03517 | |
| CHTF18 | 1.713474 | 0.025838 | |
| C19orf10 | 3.591073 | 0.021685 | |
| TMEM52 | 1.95537 | 0.025472 | |
| LOC145783 | 2.771081 | 0.024596 | |
| CDC40 | -1.2896 | 0.005326 | |
| TECR | 4.310569 | 0.000707 | |
| BIN3 | 4.14965 | 0.000809 | |
| CDCA7 | -6.74343 | 0.003725 | |
| RADIL | 3.686313 | 0.023014 | |
| ZNF717 | -8.68587 | 0.043642 | |
| RSF1 | -1.81072 | 0.006413 | |
| PCDH19 | -6.24888 | 0.009727 | |
| FLJ10213 | -9.80542 | 0.004363 | |
| RALB | -6.00673 | 0.01568 | |
| TFEB | 1.504919 | 0.032739 | |
| PCK2 | 4.027382 | 0.002151 | |
| SYTL1 | 0.627596 | 0.001528 | |
| SYTL3 | -4.11785 | 0.023381 | |
| SPRED2 | -5.5396 | 0.0472 | |
| WDFY1 | -4.55373 | 0.044596 | |
| ASTL | 8.014194 | 0.015908 | |
| ZMPSTE24 | -1.61519 | 0.008172 | |
| ZNF550 | -2.72297 | 0.034651 | |
| THOC7 | 6.884695 | 0.036699 | |
| THOC6 | 2.634884 | 0.015215 | |
| RHBDL1 | 4.775711 | 0.011418 | |
| HSD3B7 | 7.540807 | 0.000201 | |
| WHSC2 | 0.157912 | 0.007621 | |
| COPS8 | -3.20152 | 0.030958 | |
| FOXP1 | -4.72791 | 0.023014 | |
| RCCD1 | 8.062476 | 0.005866 | |
| COPS5 | 7.691164 | 0.000912 | |
| YTHDF3 | -1.38092 | 0.023381 | |
| PARP11 | -7.65072 | 0.025472 | |
| MYCN | -0.39677 | 0.013364 | |
| PLCH1 | -9.15684 | 0.007287 | |
| NLK | -0.73757 | 0.027184 | |
| FDFT1 | 1.59383 | 0.00773 | |
| NUDT6 | 4.332289 | 0.04526 | |
| TWSG1 | -6.36558 | 0.00773 | |
| NTHL1 | 2.485181 | 0.004895 | |
| LARS | -2.84671 | 0.038138 | |
| DDRGK1 | 0.5048 | 0.04526 | |
| FAM168A | -8.3735 | 0.002567 | |
| TEX11 | -2.01184 | 0.025598 | |
| MALAT1 | -3.14302 | 0.002255 | |
| TTC3 | -0.07932 | 0.010964 | |
| EVC | -11.6878 | 0.012901 | |
| UGDH | -3.40076 | 0.009388 | |
| BBX | -4.52435 | 0.002047 | |
| CXADR | -10.8607 | 0.004363 | |
| LY6G5B | -2.8546 | 0.04706 | |
| MFN1 | -1.63881 | 0.04101 | |
| KTN1 | -5.97626 | 0.002671 | |
| C20orf194 | -2.9517 | 0.026576 | |
| LRP2BP | -1.96083 | 0.044596 | |
| PURB | -1.2955 | 0.004575 | |
| ST3GAL5 | -5.76976 | 0.006742 | |
| NINJ2 | 1.512168 | 0.027562 | |
| OSBPL8 | -1.82025 | 0.030701 | |
| TUFM | 4.687947 | 0.002151 | |
| CEP135 | -15.2897 | 0.000201 | |
| ARHGAP39 | 4.751726 | 0.009059 | |
| MON1B | -3.8398 | 0.039834 | |
| ZNF836 | -2.66454 | 0.023862 | |
| CACNA1G | -2.11299 | 0.004255 | |
| SFRP4 | -1.48332 | 0.017198 | |
| SLC15A1 | 12.32361 | 0.017437 | |
| RBM41 | -7.49688 | 0.01029 | |
| RBM42 | 4.435451 | 0.000809 | |
| RBM44 | -4.01694 | 0.001219 | |
| PQBP1 | 2.588606 | 0.012101 | |
| TTC15 | 7.353468 | 0.005217 | |
| OAT | -10.5029 | 0.001014 | |
| RICTOR | -3.86484 | 0.023623 | |
| PPM1G | 7.795253 | 0.000605 | |
| EGR1 | -0.17926 | 0.044336 | |
| PJA1 | -6.28592 | 0.00806 | |
| LOC728643 | 1.950595 | 0.017552 | |
| PJA2 | -9.19358 | 0.002567 | |
| PTPN4 | -7.36971 | 0.002567 | |
| LRCH2 | -1.70543 | 0.046926 | |
| AGBL5 | 2.975574 | 0.033127 | |
| AGBL3 | -5.11472 | 0.016845 | |
| UBE3C | -4.09211 | 0.005004 | |
| ADI1 | 4.809762 | 0.01256 | |
| CNTD2 | 4.278271 | 0.02509 | |
| ZEB1 | -5.21741 | 0.023623 | |
| CEMP1 | 5.803122 | 0.000707 | |
| EIF2C3 | -7.15165 | 0.000605 | |
| EXPH5 | -2.78779 | 0.008945 | |
| DET1 | 2.183679 | 0.024966 | |
| KCNJ3 | 2.650276 | 0.032739 | |
| KCNJ2 | -1.37014 | 0.012333 | |
| AGPAT1 | -5.15993 | 0.000809 | |
| KIAA1217 | -6.49751 | 0.011187 | |
| CDC37L1 | -4.35281 | 0.042192 | |
| TCIRG1 | 6.581931 | 0.000912 | |
| PCDHB12 | -5.18954 | 0.010176 | |
| UBXN6 | 1.983151 | 0.013018 | |
| SIGIRR | 2.198812 | 0.004575 | |
| ANXA8L2 | 2.962367 | 0.043642 | |
| C19orf36 | 1.427924 | 0.029201 | |
| WAPAL | -4.54723 | 0.002567 | |
| SPAG1 | -2.87792 | 0.040368 | |
| C3orf33 | -1.19506 | 0.030323 | |
| NOXA1 | 0.664785 | 0.034651 | |
| ARHGDIA | 2.131695 | 0.023257 | |
| UBR1 | -1.75865 | 0.039974 | |
| PRMT7 | 3.049956 | 0.032481 | |
| NUCB1 | 5.415421 | 0.001322 | |
| KCNMB4 | -4.5911 | 0.030958 | |
| SOCS5 | -3.70373 | 0.006742 | |
| SOCS4 | -11.9251 | 0.001322 | |
| SOCS6 | -1.55104 | 0.002462 | |
| LOC728819 | -5.8959 | 0.001322 | |
| CCNF | 1.519686 | 0.039834 | |
| CCNC | -0.93255 | 0.030199 | |
| RBM7 | -4.4891 | 0.003091 | |
| GREM1 | -5.45473 | 0.039307 | |
| NCKAP5 | -5.98257 | 0.048566 | |
| PTN | -1.4142 | 0.018854 | |
| JMY | -3.99078 | 0.006523 | |
| SLC3A2 | 8.738249 | 0.00806 | |
| CRMP1 | -0.6698 | 0.011867 | |
| FN3K | 1.846357 | 0.003408 | |
| FAM108A1 | 3.285842 | 0.004363 | |
| MRPL10 | 6.672728 | 0.005756 | |
| MRPL11 | 2.479671 | 0.036198 | |
| MRPL12 | 2.817239 | 0.016372 | |
| DCUN1D4 | -4.85306 | 0.005109 | |
| LRIG2 | -3.66347 | 0.037615 | |
| BMPR1A | -2.90001 | 0.029201 | |
| CASKIN2 | 0.451485 | 0.011187 | |
| HBA1 | 4.991724 | 0.044861 | |
| SACS | -2.97108 | 0.031221 | |
| NCOR1 | -4.44629 | 0.049407 | |
| HBA2 | 3.210889 | 0.004788 | |
| REPS1 | -1.25167 | 0.016964 | |
| LYSMD4 | 7.284179 | 0.036198 | |
| SIAH2 | 2.833814 | 0.040494 | |
| TAF1C | 2.188669 | 0.037478 | |
| PDZRN4 | -3.57595 | 0.004363 | |
| TFR2 | 2.703633 | 0.003831 | |
| HIVEP1 | -2.49806 | 0.048705 | |
| FAM115A | -4.90193 | 0.040236 | |
| ZNF318 | -4.56337 | 0.005866 | |
| RPS19BP1 | 1.612279 | 0.036198 | |
| PGRMC2 | 2.700153 | 0.044991 | |
| PGRMC1 | 1.824592 | 0.042323 | |
| ALS2 | -6.79236 | 0.001425 | |
| DIS3 | -0.00292 | 0.028074 | |
| RGPD3 | -4.30899 | 0.009612 | |
| SDHB | 2.161438 | 0.023862 | |
| SHARPIN | 6.82517 | 0.006852 | |
| SAC3D1 | 1.7252 | 0.006413 | |
| AP1M2 | 1.45248 | 0.013939 | |
| AP1M1 | 2.973267 | 0.016845 | |
| FKBP14 | -1.07748 | 0.025472 | |
| CCDC148 | -6.61662 | 0.011641 | |
| DLG2 | -0.49034 | 0.029322 | |
| SRPK1 | -1.17535 | 0.018258 | |
| RAB3IP | -3.70366 | 0.034918 | |
| SYS1 | 0.881177 | 0.038138 | |
| FAM114A2 | -5.48365 | 0.026332 | |
| RENBP | 1.725889 | 0.013595 | |
| FBXO11 | -7.28276 | 0.0001 | |
| RAB11FIP2 | -6.34375 | 0.000707 | |
| PCBP1 | 0.551009 | 0.004575 | |
| CHSY3 | -3.35371 | 0.030323 | |
| GMIP | 0.066207 | 0.025472 | |
| ALS2CL | 0.421854 | 0.015562 | |
| STUB1 | 2.495707 | 0.001943 | |
| POLH | -10.9153 | 0.000201 | |
| ARHGAP12 | -6.18502 | 0.025717 | |
| TUBB4Q | 6.439985 | 0.00784 | |
| STARD7 | -2.24344 | 0.035045 | |
| SIRT2 | 7.37487 | 0.046509 | |
| SIRT6 | 1.115588 | 0.0267 | |
| ZDBF2 | -1.2357 | 0.024966 | |
| MRI1 | 3.427074 | 0.020502 | |
| PRDM2 | -2.84092 | 0.012446 | |
| PPP1R3F | 0.982885 | 0.026332 | |
| ZNF175 | -5.50441 | 0.04379 | |
| DSE | -4.07538 | 0.013364 | |
| PDAP1 | 3.17919 | 0.018854 | |
| GSTO1 | 0.719924 | 0.036323 | |
| SLC38A4 | -1.35791 | 0.039053 | |
| ERMP1 | -5.17917 | 0.011187 | |
| TTC37 | -3.71483 | 0.022774 | |
| ARSH | -3.02091 | 0.00806 | |
| MTHFSD | 3.41908 | 0.032481 | |
| BCLAF1 | -2.3698 | 0.004043 | |
| PLA2G5 | -1.35072 | 0.009059 | |
| HMGB2 | 2.931237 | 0.039307 | |
| HMGB1 | -1.08507 | 0.036198 | |
| ZNF81 | -3.61434 | 0.003938 | |
| CAPZA2 | -3.79741 | 0.044065 | |
| GEMIN5 | -4.81972 | 0.020976 | |
| C4orf48 | 2.261829 | 0.016487 | |
| C15orf58 | 3.259592 | 0.032481 | |
| C10orf84 | -0.76733 | 0.044736 | |
| DDX12 | 3.485013 | 0.018141 | |
| GJB3 | 2.818371 | 0.036066 | |
| GJB5 | 5.226725 | 0.008172 | |
| MYRIP | -5.75828 | 0.011302 | |
| PSMC5 | 7.346679 | 0.000912 | |
| TRIP10 | 2.642432 | 0.019323 | |
| TRIP12 | -4.26993 | 0.010515 | |
| ZNF543 | -1.70866 | 0.042836 | |
| NCLN | 4.586095 | 0.000912 | |
| C9orf152 | -9.27273 | 0.04706 | |
| DNAL1 | -6.95997 | 0.006742 | |
| LIN7A | 2.343909 | 0.030576 | |
| HNRPLL | -7.90379 | 0.001425 | |
| 5-Mar | -1.82169 | 0.000912 | |
| 7-Mar | -4.10887 | 0.000201 | |
| 6-Mar | -3.98003 | 0.017198 | |
| 3-Mar | 0.992892 | 0.039974 | |
| FOXI1 | 0.81929 | 0.049954 | |
| EIF3K | 11.51146 | 0.000605 | |
| EIF3D | 2.43647 | 0.039053 | |
| EIF3G | 4.674304 | 0.001116 | |
| SETD5 | -1.39354 | 0.016723 | |
| NOL12 | 4.302796 | 0.006523 | |
| CDKN2D | 2.075521 | 0.029693 | |
| WDR7 | -1.87006 | 0.012216 | |
| CLRN1OS | 6.749904 | 0.049546 | |
| EDEM3 | -0.51381 | 0.024349 | |
| TCF4 | -3.93169 | 0.04379 | |
| NME2 | 13.13045 | 0.000201 | |
| TRMT11 | -2.14726 | 0.011187 | |
| SMG1 | -3.1199 | 0.013479 | |
| NFATC4 | -4.33203 | 0.033879 | |
| ZNF75D | -8.42874 | 0.004788 | |
| AP2B1 | -4.31651 | 0.008834 | |
| GFER | 0.387809 | 0.033256 | |
| NUBP1 | 5.554244 | 0.001116 | |
| SCOC | -2.08734 | 0.025472 | |
| SAMD5 | -0.71605 | 0.023136 | |
| PLEK2 | 0.711761 | 0.006302 | |
| ZBTB43 | -1.55633 | 0.021329 | |
| ZNF37A | -5.14588 | 0.030958 | |
| RALY | 2.064357 | 0.001735 | |
| PCDH17 | -1.47083 | 0.047478 | |
| ALKBH6 | 1.751126 | 0.038527 | |
| ALKBH7 | 4.067383 | 0.008172 | |
| ALKBH4 | 0.400211 | 0.027312 | |
| ALKBH3 | -14.4527 | 0.002047 | |
| RALA | -2.08759 | 0.039974 | |
| C8orf58 | 2.471497 | 0.043642 | |
| C8orf55 | 9.173177 | 0.001735 | |
| C8orf51 | 3.069156 | 0.012333 | |
| ARHGDIG | 6.193345 | 0.032356 | |
| ZNF714 | -5.30808 | 0.04569 | |
| EPB49 | 0.122579 | 0.049954 | |
| DNAJB7 | -3.40628 | 0.013939 | |
| AMDHD2 | 6.246557 | 0.0001 | |
| RAB21 | -0.13223 | 0.028689 | |
| TMED7-TICAM2 | -13.0397 | 0.006192 | |
| PHIP | -2.47664 | 0.010064 | |
| ZNF808 | -2.07955 | 0.028431 | |
| HELZ | -1.60143 | 0.01279 | |
| TNIP1 | 3.836948 | 0.002255 | |
| GRIK5 | -0.78162 | 0.024966 | |
| RNF141 | -5.89979 | 0.009059 | |
| MSH3 | -2.2856 | 0.040368 | |
| MSH6 | -1.89833 | 0.011187 | |
| CD320 | 1.744038 | 0.001839 | |
| STOX1 | 3.733694 | 0.011641 | |
| C16orf45 | 1.627525 | 0.039974 | |
| RNF19A | -0.51234 | 0.0235 | |
| NFXL1 | -1.17983 | 0.002671 | |
| C16orf42 | 1.23879 | 0.00288 | |
| GREM2 | -2.30232 | 0.008945 | |
| NT5M | 1.219065 | 0.022291 | |
| HIST2H2AC | 3.459213 | 0.016139 | |
| WDR72 | 4.515429 | 0.040236 | |
| C19orf2 | -2.43565 | 0.029074 | |
| C19orf6 | 2.87123 | 0.003198 | |
| OCIAD2 | 2.702172 | 0.021925 | |
| PPP1R3C | -4.30815 | 0.037478 | |
| TMEM191A | 1.988013 | 0.000707 | |
| TGS1 | -0.4073 | 0.021571 | |
| YTHDC1 | -4.25219 | 0.011418 | |
| INPP5A | -2.9373 | 0.002776 | |
| NME3 | 3.035762 | 0.004363 | |
| RAI14 | -3.25815 | 0.016723 | |
| PID1 | -2.19477 | 0.008615 | |
| NOSTRIN | -7.14943 | 0.00917 | |
| RCBTB1 | -3.40367 | 0.028314 | |
| EPB42 | 11.66033 | 0.015908 | |
| HELB | -1.36715 | 0.048979 | |
| RBAK | -1.97543 | 0.011418 | |
| PNPLA2 | 1.671154 | 0.001839 | |
| PNPLA6 | 1.393485 | 0.034524 | |
| PNPLA8 | -1.85636 | 0.039053 | |
| NCKAP1 | -2.62413 | 0.007949 | |
| WEE1 | -2.67562 | 0.016139 | |
| KATNAL1 | -6.47459 | 0.009059 | |
| KIAA0753 | -5.54548 | 0.019686 | |
| STEAP2 | -0.98213 | 0.048425 | |
| TSFM | 1.229898 | 0.035422 | |
| C1orf43 | 3.19529 | 0.034132 | |
| UPF2 | -0.18593 | 0.037211 | |
| GPR88 | -1.77016 | 0.039434 | |
| GPR82 | -1.49918 | 0.021571 | |
| GPR83 | -1.97719 | 0.011418 | |
| SLC38A10 | 1.724745 | 0.00288 | |
| ZNF295 | -2.4614 | 0.014985 | |
| RNPEPL1 | 1.716181 | 0.01992 | |
| ZC3HAV1L | -6.3363 | 0.00784 | |
| ADAM22 | -6.67531 | 0.014404 | |
| ADAM28 | -1.00377 | 0.048849 | |
| GSTM1 | 4.056111 | 0.02509 | |
| GSTM2 | 3.556727 | 0.01256 | |
| GSTM4 | 3.260693 | 0.034918 | |
| EFHD2 | 4.989484 | 0.001631 | |
| AEBP2 | -5.76057 | 0.003198 | |
| ZNF322A | -4.64648 | 0.016845 | |
| RCHY1 | -5.01786 | 0.008394 | |
| DKFZp686O24166 | -4.16285 | 0.003091 | |
| ANXA5 | 1.411408 | 0.027825 | |
| MEST | -4.58013 | 0.003302 | |
| MCM7 | 4.193123 | 0.011529 | |
| MCM5 | 7.515409 | 0.009727 | |
| DUSP19 | -1.42805 | 0.048425 | |
| DUSP10 | -7.44873 | 0.027949 | |
| EPC2 | -3.20812 | 0.001219 | |
| EPC1 | -1.57709 | 0.028314 | |
| SF3A2 | 2.249076 | 0.013134 | |
| MS4A7 | -4.70958 | 0.016139 | |
| ZNF141 | -8.54858 | 0.002671 | |
| ZNF142 | -3.73705 | 0.000503 | |
| ZNF146 | -1.46077 | 0.000503 | |
| ZNF148 | -2.72367 | 0.016723 | |
| COX8A | 1.416617 | 0.034264 | |
| C20orf20 | 5.096503 | 0.011984 | |
| LOC283922 | -6.9736 | 0.001219 | |
| C20orf27 | 4.827695 | 0.006084 | |
| TMF1 | -1.84098 | 0.022171 | |
| PMS1 | -5.12984 | 0.000301 | |
| C9orf173 | 5.900284 | 0.033758 | |
| BRMS1 | 3.536868 | 0.02882 | |
| GRIP1 | -8.48198 | 0.003514 | |
| SLC19A2 | 2.09839 | 0.014057 | |
| TDO2 | -6.13892 | 0.010736 | |
| MAP2K2 | 2.582217 | 0.001116 | |
| TAF13 | -3.39469 | 0.001528 | |
| SLC35A3 | -1.94136 | 0.044736 | |
| MPDZ | -2.18747 | 0.026206 | |
| FAF2 | -4.56458 | 0.004363 | |
| PAK4 | 2.798892 | 0.031477 | |
| TMEM44 | 2.861701 | 0.009951 | |
| CYHR1 | 4.791673 | 0.008394 | |
| HOXD13 | 3.854986 | 0.041557 | |
| ZNF737 | -4.56997 | 0.030199 | |
| ZNF296 | 2.91098 | 0.049546 | |
| SORCS1 | -3.25562 | 0.003091 | |
| CARS2 | 4.732482 | 0.003198 | |
| GPKOW | 0.435341 | 0.010402 | |
| TNPO1 | -6.1273 | 0.006523 | |
| MAPK3 | 5.735709 | 0.017082 | |
| MAPK6 | -0.15611 | 0.013134 | |
| BCO2 | -0.61291 | 0.017669 | |
| MAPK8 | -5.73291 | 0.000201 | |
| MAPK9 | -7.36777 | 0.010515 | |
| WTAP | -2.08215 | 0.023623 | |
| DAZAP1 | 4.411553 | 0.007621 | |
| ATP8A2 | -0.02982 | 0.007178 | |
| PGPEP1L | 15.57537 | 0.038138 | |
| STAG3L1 | -4.67132 | 0.046926 | |
| KIAA1804 | -2.6366 | 0.030958 | |
| BCOR | -5.56531 | 0.048284 | |
| BCAP31 | 8.552316 | 0.0001 | |
| EPM2AIP1 | -2.98097 | 0.049407 | |
| FDX1L | 3.72849 | 0.010849 | |
| C1orf109 | -1.23849 | 0.010736 | |
| KCTD9 | 3.50785 | 0.033127 | |
| HCFC2 | -5.01626 | 0.002671 | |
| ZNF432 | -2.26393 | 0.012101 | |
| CABLES2 | 5.106525 | 0.041681 | |
| MTUS2 | -4.02198 | 0.002567 | |
| ESF1 | -3.4685 | 0.009388 | |
| FOXD4L2 | -0.65739 | 0.048425 | |
| PACS1 | 2.793919 | 0.046646 | |
| RNF165 | -2.00411 | 0.009951 | |
| RNF169 | -6.66422 | 0.005217 | |
| C8orf77 | 3.381655 | 0.033127 | |
| BRAF | -2.05437 | 0.040368 | |
| PGAP1 | -5.75765 | 0.001014 | |
| ANP32B | 2.55838 | 0.006302 | |
| FDXR | 2.357477 | 0.007069 | |
| SP3 | -8.31552 | 0.000301 | |
| ALMS1 | -4.94754 | 0.00468 | |
| ATRNL1 | -1.12969 | 0.005435 | |
| MRPL51 | 5.732675 | 0.02882 | |
| MALT1 | -2.27663 | 0.033879 | |
| PRKG1 | -8.72903 | 0.005648 | |
| PRKRIP1 | 1.470644 | 0.013709 | |
| ZNF688 | 2.747023 | 0.003198 | |
| STK11 | 0.80946 | 0.000912 | |
| OPN1SW | 6.354765 | 0.026087 | |
| PRKG2 | -8.5135 | 0.0001 | |
| ZNF684 | -3.55168 | 0.033256 | |
| GMCL1L | -5.54313 | 0.013249 | |
| GSTM3 | 2.095594 | 0.04623 | |
| MMP13 | -6.12429 | 0.018854 | |
| DSTYK | -4.56252 | 0.008834 | |
| HSCB | 3.817419 | 0.014057 | |
| NF1 | -4.59845 | 0.032356 | |
| GPAA1 | 4.08223 | 0.008282 | |
| FAM133B | -2.52461 | 0.016372 | |
| KCNK12 | 9.731383 | 0.01429 | |
| LOC100129716 | -3.1498 | 0.007399 | |
| ZNF594 | -5.65584 | 0.016964 | |
| ZNF420 | -5.29537 | 0.019323 | |
| NPY1R | 0.36273 | 0.032229 | |
| NEDD1 | -6.27881 | 0.004469 | |
| SMC3 | -4.23123 | 0.022539 | |
| SMC2 | -3.47246 | 0.037339 | |
| P2RY11 | 0.094881 | 0.008945 | |
| SPDYE5 | -3.84401 | 0.028689 | |
| THOP1 | 3.972548 | 0.000605 | |
| ODF3B | 4.492152 | 0.016845 | |
| WWC2 | -6.46971 | 0.044206 | |
| SLC7A2 | -3.58221 | 0.044206 | |
| PRDX5 | 2.621054 | 0.006962 | |
| ALG11 | -4.81953 | 0.019686 | |
| PRDX4 | 3.641083 | 0.048158 | |
| INSR | 6.758746 | 0.001425 | |
| BANF1 | 3.105792 | 0.014637 | |
| RBMS1 | -4.59214 | 0.033127 | |
| PLA2G12A | -3.93699 | 0.018375 | |
| SCN2A | -0.64004 | 0.013479 | |
| NUBP2 | 3.213245 | 0.006742 | |
| TMEM217 | -1.88148 | 0.01452 | |
| SFRS7 | -2.78838 | 0.00751 | |
| SHISA9 | -8.91408 | 0.016964 | |
| ZNF678 | -10.9613 | 0.0001 | |
| SYNJ2 | -7.30252 | 0.000605 | |
| STK38L | -5.12683 | 0.000707 | |
| MKLN1 | -4.86729 | 0.009059 | |
| EEFSEC | 2.104591 | 0.019323 | |
| TIMM13 | 1.605525 | 0.001735 | |
| SLC41A3 | 3.478478 | 0.032229 | |
| SLC41A2 | -4.41299 | 0.01256 | |
| SCARB2 | -3.23708 | 0.004788 | |
| LYRM2 | -0.13017 | 0.037615 | |
| ZNF33A | -4.75588 | 0.011752 | |
| WNT7B | -7.27028 | 0.040101 | |
| CPT1A | -2.72912 | 0.043371 | |
| CNTN2 | -3.8141 | 0.00362 | |
| CNTN3 | -12.563 | 0.0001 | |
| ANXA2 | -4.58591 | 0.037615 | |
| DDX51 | 1.474152 | 0.045404 | |
| CSRNP2 | -0.92642 | 0.011984 | |
| CDC42BPA | -4.80885 | 0.045404 | |
| COX6C | 3.520935 | 0.017318 | |
| NUPR1 | 5.585045 | 0.001943 | |
| UBE2E1 | -0.4139 | 0.021449 | |
| RNASEN | -1.67429 | 0.006192 | |
| DNAH6 | -5.36219 | 0.005326 | |
| DNAH5 | -8.13259 | 0.006413 | |
| DNAH9 | -0.67145 | 0.032606 | |
| MCM3 | 5.916629 | 0.041143 | |
| FLG | -3.35117 | 0.00288 | |
| TMEM130 | -1.45792 | 0.047335 | |
| TMEM131 | -4.65662 | 0.013018 | |
| CCDC46 | -4.56502 | 0.043102 | |
| SPRY3 | -2.21799 | 0.027692 | |
| SPRY4 | -2.17973 | 0.002358 | |
| CDKL2 | -1.00199 | 0.011984 | |
| FBXO27 | 4.085471 | 0.007287 | |
| MCM9 | -0.78358 | 0.029322 | |
| FBXO24 | 1.053965 | 0.043239 | |
| DYSFIP1 | 16.82431 | 0.003408 | |
| ACPL2 | -2.98096 | 0.022044 | |
| FCRLB | 6.186447 | 0.046509 | |
| C11orf83 | 1.199601 | 0.015328 | |
| MUCL1 | -0.91219 | 0.020039 | |
| FBXW5 | 1.624064 | 0.00917 | |
| C1D | -2.11255 | 0.006084 | |
| NFATC2IP | 7.574875 | 0.049954 | |
| TET3 | -3.45429 | 0.000809 | |
| KRI1 | 2.355392 | 0.002255 | |
| TRIM35 | 4.050854 | 0.025219 | |
| FIBP | 0.661485 | 0.048026 | |
| LRRC16A | -3.87614 | 0.018733 | |
| ZNF227 | -2.05099 | 0.049685 | |
| UBXN4 | -3.20065 | 0.000912 | |
| CLN3 | 5.150272 | 0.019686 | |
| SLMO1 | 1.875763 | 0.045554 | |
| AUP1 | 2.034593 | 0.006962 | |
| PIN4 | 3.325754 | 0.010964 | |
| OR51I1 | 16.8389 | 0.019563 | |
| LIMA1 | -8.08635 | 0.023257 | |
| BMPR2 | -6.22235 | 0.009498 | |
| ZBTB20 | -2.92439 | 0.036198 | |
| HRAS | 1.080828 | 0.039053 | |
| CCDC163P | -4.19105 | 0.043928 | |
| C16orf89 | -1.52107 | 0.011302 | |
| SYT13 | -5.86609 | 0.006413 | |
| SYT17 | -8.20349 | 0.022539 | |
| JMJD1C | -1.07078 | 0.000912 | |
| SH2D3A | 1.665524 | 0.00362 | |
| ZNF143 | -1.96656 | 0.041273 | |
| DDT | 3.354723 | 0.019801 | |
| PCDHGA4 | -3.86674 | 0.033506 | |
| RNF160 | -3.07953 | 0.004255 | |
| ATP6V1G1 | 4.406404 | 0.002255 | |
| GPR173 | -0.40963 | 0.010515 | |
| VPS28 | 6.293765 | 0.000605 | |
| SERP2 | -5.23154 | 0.022171 | |
| NUAK1 | -5.30201 | 0.042192 | |
| RPS24 | 4.968836 | 0.041932 | |
| LEAP2 | -2.14817 | 0.029693 | |
| CHMP1A | 2.707283 | 0.0151 | |
| UCHL1 | 7.459229 | 0.01992 | |
| CHAF1A | 5.76437 | 0.011187 | |
| HSD11B1L | 3.229971 | 0.027692 | |
| NFYA | -0.85957 | 0.001322 | |
| ZNF354B | -4.69777 | 0.002358 | |
| MSL3L2 | -3.01734 | 0.019801 | |
| SCGB2A2 | -2.1444 | 0.04706 | |
| DDX3X | -5.07142 | 0.0001 | |
| SOD1 | 3.004823 | 0.040236 | |
| TERC | 16.61121 | 0.00917 | |
| VWA2 | -6.96743 | 0.022418 | |
| KIAA0240 | -5.54293 | 0.022418 | |
| PRDM8 | -3.81822 | 0.049407 | |
| LSG1 | -1.06929 | 0.038902 | |
| PYGB | 5.53776 | 0.010515 | |
| AKT2 | 4.365743 | 0.005866 | |
| TTC9B | 15.14022 | 0.000605 | |
| PKD1L1 | -1.79249 | 0.000809 | |
| ANAPC11 | 3.106439 | 0.00773 | |
| ENTPD2 | 3.643784 | 0.043102 | |
| SNAPC5 | 2.364827 | 0.040236 | |
| RNF41 | -6.00699 | 0.001219 | |
| HYAL3 | 3.487488 | 0.030452 | |
| SPC24 | 4.363263 | 0.001839 | |
| ZFP14 | -3.94144 | 0.01992 | |
| LOC100216545 | 0.639115 | 0.046364 | |
| DCTN1 | 2.722001 | 0.003514 | |
| NAT8 | -0.94807 | 0.034782 | |
| RRN3P2 | -4.23917 | 0.0316 | |
| KIF3B | -4.48778 | 0.006523 | |
| KIF3A | -1.08559 | 0.032994 | |
| TMEM9B | -2.02776 | 0.033256 | |
| UBXN1 | 1.05119 | 0.001839 | |
| HNRNPH1 | -1.23927 | 0.016025 | |
| SNRNP48 | -3.67138 | 0.028074 | |
| PAPOLG | -2.67493 | 0.000605 | |
| PGM2L1 | -1.40104 | 0.00288 | |
| HCFC1R1 | 0.231539 | 0.020617 | |
| FAM127B | 3.898132 | 0.005866 | |
| SEL1L | -9.5662 | 0.000809 | |
| MCM3APAS | -6.8141 | 0.010064 | |
| KIAA2018 | -2.98099 | 0.002776 | |
| KIAA2013 | 4.254201 | 0.024719 | |
| ZG16 | 15.04948 | 0.037211 | |
| OSGIN1 | 4.962617 | 0.030825 | |
| PAIP1 | -2.51344 | 0.001735 | |
| ZNF519 | -4.41143 | 0.003831 | |
| MLYCD | 4.395369 | 0.014752 | |
| TEAD1 | -2.72977 | 0.047891 | |
| CCDC22 | 2.165506 | 0.011418 | |
| CNTNAP1 | -3.72385 | 0.011984 | |
| DCAF17 | -2.31893 | 0.0001 | |
| DCAF15 | 5.246267 | 0.005974 | |
| APOB48R | 3.284806 | 0.014404 | |
| TXK | -0.59466 | 0.031477 | |
| PLCL2 | -1.51703 | 0.046646 | |
| UTP23 | -0.08473 | 0.003514 | |
| KIAA1432 | -2.9001 | 0.001116 | |
| NMD3 | -0.97334 | 0.018616 | |
| RANBP3L | -1.87713 | 0.038396 | |
| TAZ | 2.959277 | 0.021329 | |
| G3BP1 | -5.40512 | 0.018854 | |
| G3BP2 | -5.85921 | 0.00806 | |
| USP31 | -0.58331 | 0.04623 | |
| APBB2 | -6.77666 | 0.029822 | |
| ANXA2P2 | -5.92467 | 0.015562 | |
| IRAK1 | 6.978844 | 0.011418 | |
| TAF1A | -5.47314 | 0.010736 | |
| TXNDC9 | -1.4641 | 0.006302 | |
| CEP290 | -2.63263 | 0.032356 | |
| TCF15 | 1.993696 | 0.041806 | |
| SEPT7P2 | -2.74938 | 0.023623 | |
| GABBR1 | -7.72804 | 0.010176 | |
| C6orf72 | -3.33689 | 0.018854 | |
| SSTR2 | -1.58534 | 0.045966 | |
| IRGQ | -1.55973 | 0.005435 | |
| PDLIM2 | 2.137334 | 0.027184 | |
| CHRAC1 | 2.893296 | 0.008172 | |
| FTL | 5.154754 | 0.016372 | |
| HES4 | 4.657836 | 0.000402 | |
| KLHL23 | -5.42081 | 0.00362 | |
| KLHL28 | -9.8936 | 0.004575 | |
| TAF15 | 1.757338 | 0.009612 | |
| SART1 | 0.063767 | 0.000605 | |
| NDUFV1 | 2.95098 | 0.006084 | |
| ZNF213 | 4.653943 | 0.01568 | |
| PCIF1 | 7.736748 | 0.021209 | |
| GGN | 2.333234 | 0.036066 | |
| WDR62 | 1.727836 | 0.047335 | |
| RAD23A | 4.192215 | 0.037211 | |
| GPR152 | 9.301483 | 0.016723 | |
| SSBP4 | 2.952659 | 0.004895 | |
| COBRA1 | 2.362646 | 0.012446 | |
| TM4SF1 | -3.09476 | 0.01029 | |
| HSD17B8 | 1.701131 | 0.001839 | |
| HMGCL | 1.233263 | 0.009279 | |
| TOX4 | -3.83489 | 0.025598 | |
| TRDMT1 | -4.32716 | 0.001219 | |
| PRMT3 | -2.03049 | 0.027949 | |
| NUP155 | -2.9985 | 0.035801 | |
| HGS | 6.274186 | 0.026453 | |
| LOC220729 | -4.49298 | 0.006852 | |
| C7orf68 | 4.702044 | 0.013709 | |
| C7orf63 | -6.49733 | 0.002776 | |
| ZNF462 | -1.44473 | 0.010964 | |
| PDZD8 | -5.69925 | 0.003302 | |
| ZNF397 | -3.36992 | 0.008834 | |
| LOC387647 | -4.4385 | 0.008504 | |
| PKP2 | -3.92423 | 0.029074 | |
| TOPORS | -2.46489 | 0.028945 | |
| FAM173A | 3.398412 | 0.025598 | |
| STARD3NL | -2.61495 | 0.004043 | |
| TEP1 | -5.95529 | 0.007287 | |
| LYPD5 | 10.50027 | 0.002462 | |
| ILVBL | 5.16759 | 0.021449 | |
| TNFRSF25 | 0.807704 | 0.017437 | |
| CNOT6 | -2.54898 | 0.001219 | |
| MBLAC1 | 0.197243 | 0.04526 | |
| CRELD2 | 2.090823 | 0.034264 | |
| TOP1MT | 9.22721 | 0.000301 | |
| ARL4D | 2.589346 | 0.041557 | |
| NMRAL1 | 6.711623 | 0.000301 | |
| ZFC3H1 | -1.19789 | 0.010402 | |
| MRAP | 9.367401 | 0.049407 | |
| GALNT7 | -6.1118 | 0.002776 | |
| DGAT2L6 | 13.45684 | 0.044336 | |
| CLOCK | -3.72972 | 0.00288 | |
| RFC3 | -0.70227 | 0.024103 | |
| CCDC64B | 2.848099 | 0.012901 | |
| CBX8 | 5.476059 | 0.018616 | |
| CBX5 | -2.33667 | 0.014175 | |
| FASTKD2 | -1.99971 | 0.001219 | |
| FASTKD1 | -2.26388 | 0.009727 | |
| NLRP9 | -0.45748 | 0.018854 | |
| WDR24 | 5.260948 | 0.003725 | |
| MMGT1 | -3.29165 | 0.04925 | |
| RAB13 | 5.094961 | 0.010176 | |
| PPP1R12A | -4.60586 | 0.013709 | |
| GNAQ | -4.57678 | 0.030576 | |
| NAGLU | 0.917185 | 0.04101 | |
| C15orf61 | 6.806995 | 0.025219 | |
| KIF1B | -4.48092 | 0.001735 | |
| SHOC2 | -0.99759 | 0.006631 | |
| AATF | 4.623404 | 0.01256 | |
| TMSB15B | -3.71706 | 0.016025 | |
| TPI1 | 5.375997 | 0.047478 | |
| USP40 | -3.49042 | 0.036825 | |
| ERCC3 | -2.14369 | 0.011418 | |
| ERCC4 | -2.68703 | 0.013709 | |
| MRPS12 | 6.435423 | 0.011529 | |
| MRPS11 | 5.170903 | 0.013249 | |
| PFKM | -5.47465 | 0.010402 | |
| TNNI2 | 8.128407 | 0.00751 | |
| FAM125A | 1.546966 | 0.020039 | |
| KCND1 | -1.66962 | 0.030199 | |
| KCND2 | -0.74031 | 0.038658 | |
| ABHD14A | 2.184884 | 0.043642 | |
| SSR2 | 3.193308 | 0.045554 | |
| SSR4 | 7.327634 | 0.001219 | |
| LSMD1 | 3.112629 | 0.017318 | |
| USP46 | -8.63589 | 0.000201 | |
| CASP8AP2 | -2.79791 | 0.006742 | |
| APOC4 | 8.190901 | 0.025963 | |
| APOC2 | 0.214023 | 0.039434 | |
| MAP4K3 | -2.79443 | 0.038003 | |
| PYCARD | 3.160312 | 0.017082 | |
| KLHDC10 | -4.76954 | 0.031221 | |
| ZFPM1 | 0.643377 | 0.024349 | |
| LAS1L | 13.37305 | 0.0001 | |
| SLC25A23 | 5.341802 | 0.013364 | |
| SLC25A27 | -2.02807 | 0.010064 | |
| PRMT10 | -0.97685 | 0.04761 | |
| MAGIX | 2.405596 | 0.002985 | |
| SPTY2D1 | -6.45001 | 0.005648 | |
| GPBP1L1 | -4.61028 | 0.000301 | |
| COPB1 | -3.30831 | 0.00288 | |
| INSRR | -0.13558 | 0.014637 | |
| KDM4C | -0.68684 | 0.046646 | |
| CARM1 | 6.611443 | 0.017318 | |
| GLB1 | 4.461599 | 0.036574 | |
| SFRS18 | -7.59997 | 0.000301 | |
| USP15 | -3.31077 | 0.001322 | |
| FAM22D | -2.74026 | 0.016025 | |
| RPAP3 | -2.61897 | 0.030199 | |
| TOMM20 | 3.518171 | 0.048705 | |
| RPUSD1 | 8.5953 | 0.002985 | |
| SNORA5A | 12.07188 | 0.035926 | |
| KIAA0100 | -1.34946 | 0.043928 | |
| SNX17 | 5.780134 | 0.017788 | |
| SNX15 | 3.468214 | 0.018258 | |
| SNX13 | -6.85668 | 0.002047 | |
| TOMM22 | 5.858248 | 0.019686 | |
| DPM3 | 4.081918 | 0.020976 | |
| APOA1BP | 6.38827 | 0.030701 | |
| OCLM | -2.00745 | 0.04623 | |
| GPR37L1 | 5.558075 | 0.047478 | |
| STK32B | -2.1297 | 0.021329 | |
| SPATA20 | 1.08603 | 0.012333 | |
| GPD2 | -4.36458 | 0.033632 | |
| LOC100170939 | -1.53583 | 0.009612 | |
| SON | -2.47502 | 0.007178 | |
| C2orf71 | 11.12566 | 0.034132 | |
| C2orf72 | -7.05608 | 0.010402 | |
| C2orf79 | 2.746671 | 0.018258 | |
| GAA | 5.276227 | 0.001943 | |
| NUMB | -5.04047 | 0.007621 | |
| SCAND1 | 6.502726 | 0.005974 | |
| SERPINA6 | 8.560663 | 0.01279 | |
| MS4A14 | -0.72272 | 0.04775 | |
| EXOC6B | -7.51531 | 0.017669 | |
| CYC1 | 10.74037 | 0.000809 | |
| HAGH | 2.611581 | 0.015442 | |
| SPATA2L | 0.296179 | 0.009612 | |
| CKAP5 | -1.99773 | 0.008394 | |
| SYNGAP1 | -6.03883 | 0.001014 | |
| GOLGA6L10 | -5.25059 | 0.003198 | |
| SPINT2 | 5.839289 | 0.010064 | |
| NAPG | -7.30404 | 0.00751 | |
| NDST2 | -3.46936 | 0.002462 | |
| CYP39A1 | -0.62383 | 0.04297 | |
| SCAND2 | -3.50712 | 0.034918 | |
| NME1 | 6.701607 | 0.003725 | |
| PDCD2 | -2.04892 | 0.041419 | |
| LOC441455 | -3.78826 | 0.003831 | |
| C7orf47 | 3.897015 | 0.014175 | |
| RHOT2 | 2.202398 | 0.006631 | |
| GFRA3 | 12.66589 | 0.034782 | |
| PCMT1 | -2.09942 | 0.007949 | |
| PVRL1 | -0.18679 | 0.049111 | |
| NBEA | -14.3884 | 0.002776 | |
| OSM | -4.34663 | 0.019686 | |
| OS9 | 3.334065 | 0.014985 | |
| LNX2 | -3.70041 | 0.031221 | |
| STAG2 | -2.88187 | 0.038138 | |
| AP1G1 | -1.33857 | 0.042586 | |
| ZNF385B | -0.44167 | 0.045966 | |
| DCAF4L2 | 13.0973 | 0.02882 | |
| EHD3 | -5.19427 | 0.006084 | |
| GMFB | -7.87544 | 0.000301 | |
| LOC100271831 | 6.01526 | 0.005542 | |
| LOC100271836 | -2.67312 | 0.038527 | |
| TMED7 | -4.41994 | 0.03645 | |
| TMED4 | 3.423718 | 0.028689 | |
| TMED3 | 5.682224 | 0.04101 | |
| REEP4 | 10.40283 | 0.000707 | |
| TMED1 | 3.28083 | 0.006302 | |
| BAIAP2L2 | 7.070609 | 0.044991 | |
| HNRNPM | 0.571859 | 0.01029 | |
| SNX9 | -7.4468 | 0.004575 | |
| SBNO1 | -4.9408 | 0.012333 | |
| USPL1 | -3.92158 | 0.000605 | |
| MPHOSPH9 | -5.09084 | 0.025963 | |
| ZNF174 | 3.583885 | 0.032739 | |
| NDUFS8 | 4.774412 | 0.008282 | |
| LOC150381 | 0.129798 | 0.038527 | |
| RAB39 | -0.83488 | 0.018733 | |
| DOK6 | -4.15671 | 0.037615 | |
| DOK4 | 3.405401 | 0.020853 | |
| C6orf227 | 10.2628 | 0.020976 | |
| C8orf73 | 5.175963 | 0.006413 | |
| CLASP1 | -4.49964 | 0.001425 | |
| ARMC2 | -0.88149 | 0.005756 | |
| YPEL3 | 4.861744 | 0.009498 | |
| CD160 | -2.99828 | 0.038396 | |
| CD164 | -0.45897 | 0.039834 | |
| C5orf56 | -6.23818 | 0.009059 | |
| C5orf53 | -2.23893 | 0.039974 | |
| C5orf51 | -3.22901 | 0.038003 | |
| KHSRP | 5.917682 | 0.007949 | |
| ZDHHC17 | -6.33066 | 0.002151 | |
| RAP1GAP2 | -6.27936 | 0.030452 | |
| MAP2K4 | -5.06492 | 0.001943 | |
| NET1 | -3.93427 | 0.007399 | |
| TBC1D25 | -4.34792 | 0.016254 | |
| TMEM185B | -2.97557 | 0.036574 | |
| CLEC2A | 16.2196 | 0.049685 | |
| CTBP1 | 0.124691 | 0.015562 | |
| GPRASP1 | -0.00333 | 0.046364 | |
| XPO1 | -3.8597 | 0.001014 | |
| XPO4 | -7.32128 | 0.000402 | |
| LPIN3 | 2.232685 | 0.023744 | |
| SCARNA10 | 8.130184 | 0.04761 | |
| TMTC2 | -8.8563 | 0.006413 | |
| SPRR2E | 16.5454 | 0.02509 | |
| HPDL | 8.607589 | 0.007399 | |
| ATAD5 | -4.44522 | 0.037478 | |
| ATAD1 | -7.93042 | 0.001839 | |
| IBTK | -1.65065 | 0.026453 | |
| TOP1 | -5.68078 | 0.003091 | |
| C22orf29 | -1.26983 | 0.04379 | |
| MRPL54 | 1.912963 | 0.005435 | |
| PELI3 | 5.705248 | 0.014057 | |
| ATE1 | -5.98079 | 0.039834 | |
| HTR7P1 | -0.06759 | 0.011641 | |
| GET4 | 2.056583 | 0.035674 | |
| FLJ30679 | 7.53214 | 0.027825 | |
| BDKRB2 | -6.10364 | 0.033378 | |
| YAF2 | -3.31148 | 0.025472 | |
| C6orf35 | -4.82369 | 0.003408 | |
| EEA1 | -3.8518 | 0.006084 | |
| ZCCHC10 | -5.50911 | 0.005648 | |
| SLIT3 | -3.37904 | 0.013595 | |
| RRAS | 3.867677 | 0.027562 | |
| HOMER1 | -3.07579 | 0.028689 | |
| HOMER3 | 2.067261 | 0.017198 | |
| MSL2 | -3.29605 | 0.007287 | |
| RPL29 | 3.26938 | 0.012333 | |
| THAP6 | -5.72735 | 0.035801 | |
| QTRTD1 | -0.47746 | 0.044065 | |
| NAP1L3 | -5.98426 | 0.024471 | |
| CCRL1 | -2.70477 | 0.031221 | |
| FAM58A | 4.172165 | 0.027184 | |
| HDAC2 | -0.33101 | 0.032102 | |
| MUC16 | 5.470352 | 0.014404 | |
| HDAC8 | 3.445835 | 0.020976 | |
| SLC16A13 | 6.658664 | 0.030576 | |
| FNBP1L | -3.80896 | 0.002462 | |
| CHM | -4.08551 | 0.036825 | |
| TBL3 | 1.4285 | 0.029943 | |
| SH3BGRL | -5.62964 | 0.01029 | |
| MANBA | 2.45695 | 0.046088 | |
| UPK3B | 11.11428 | 0.01429 | |
| WNK1 | -2.63725 | 0.021571 | |
| DBP | 4.066079 | 0.021925 | |
| ZBTB33 | -1.60249 | 0.001425 | |
| ZNF423 | -6.36885 | 0.036066 | |
| LOC284837 | 4.282244 | 0.032481 | |
| SERINC1 | -4.56299 | 0.012216 | |
| TREX2 | 8.921332 | 0.009059 | |
| MXD3 | 2.905885 | 0.013595 | |
| POP7 | 3.118777 | 0.049407 | |
| SDF2L1 | 1.721656 | 0.021209 | |
| BVES | -1.21816 | 0.028689 | |
| ZBTB49 | -2.56726 | 0.046926 | |
| EPS8L3 | 17.25599 | 0.01452 | |
| ABTB1 | 2.481719 | 0.008945 | |
| KIAA0494 | -6.13552 | 0.047478 | |
| LGR5 | -5.564 | 0.000809 | |
| ZUFSP | -3.21706 | 0.006084 | |
| ELAVL1 | 7.201198 | 0.006192 | |
| PRPF40A | -0.37939 | 0.000503 | |
| RPS6KB2 | 4.195772 | 0.026206 | |
| TBPL1 | -1.45582 | 0.023744 | |
| SALL1 | -8.56796 | 0.012216 | |
| TET2 | -0.16388 | 0.017318 | |
| MOBKL3 | -0.31046 | 0.00784 | |
| FSD1L | -5.01801 | 0.048705 | |
| ATXN2L | 7.31632 | 0.014057 | |
| F2RL3 | 1.725569 | 0.045966 | |
| MED21 | -1.76739 | 0.044466 | |
| OTUB1 | 1.768646 | 0.033127 | |
| NPTN | -5.60971 | 0.035801 | |
| LOC146880 | 2.421362 | 0.030199 | |
| TRIM54 | 13.94333 | 0.004149 | |
| TRAPPC6A | 1.317473 | 0.004788 | |
| APOE | 2.719124 | 0.004149 | |
| MED23 | -9.34441 | 0.000605 | |
| SR140 | -1.47169 | 0.01802 | |
| SUMO1P3 | -1.08344 | 0.044206 | |
| KATNB1 | 2.139179 | 0.038658 | |
| SCGB3A1 | 4.801899 | 0.030825 | |
| DCAF5 | -6.62471 | 0.019205 | |
| ZFX | -10.2264 | 0.000605 | |
| ZFR | -2.33442 | 0.000809 | |
| POLR3D | 1.435711 | 0.045821 | |
| POLR3E | 1.226159 | 0.023744 | |
| POLR3C | 4.681397 | 0.0435 | |
| POLR3A | -1.40585 | 0.038138 | |
| TXNDC2 | -0.66242 | 0.048705 | |
| POLR3K | 3.800185 | 0.020617 | |
| PKP4 | -8.44606 | 0.001116 | |
| DNMT1 | 6.346532 | 0.018854 | |
| PSD3 | 1.630504 | 0.014637 | |
| TPST1 | -7.8731 | 0.005756 | |
| LOC728758 | 3.965905 | 0.009727 | |
| WDR60 | -5.53656 | 0.03957 | |
| KIF5B | -7.59333 | 0.0001 | |
| MED26 | 4.466587 | 0.024966 | |
| TRIM52 | -5.75334 | 0.010515 | |
| KAT2A | 2.10962 | 0.043642 | |
| NCRNA00160 | 2.014768 | 0.048849 | |
| SESTD1 | -1.53957 | 0.023983 | |
| FOXJ3 | -0.99904 | 0.001322 | |
| USP49 | -7.54941 | 0.000707 | |
| USP47 | -7.32115 | 0.002255 | |
| PYCRL | 7.17742 | 0.005974 | |
| USP42 | -2.09819 | 0.042069 | |
| RAD50 | -4.66925 | 0.04379 | |
| SCRN2 | 2.022884 | 0.005866 | |
| NANOS3 | 7.809351 | 0.021209 | |
| UEVLD | -5.53158 | 0.008615 | |
| MMACHC | -5.60664 | 0.002985 | |
| C10orf47 | 1.580877 | 0.005648 | |
| C10orf46 | -1.83486 | 0.048849 | |
| NEU3 | -8.41806 | 0.000809 | |
| USP8 | -1.60387 | 0.039307 | |
| FAM199X | -9.32841 | 0.000809 | |
| PPP1R16A | 4.151238 | 0.002776 | |
| RPS7 | 8.590973 | 0.005435 | |
| RPS9 | 0.337865 | 0.018141 | |
| DHX57 | -1.14111 | 0.022418 | |
| TMEM205 | 1.153988 | 0.006523 | |
| HMGCS1 | -0.14558 | 0.033127 | |
| PHF5A | 5.384232 | 0.027562 | |
| IGSF22 | -3.12034 | 0.035674 | |
| GALM | 2.626644 | 0.045966 | |
| ZSCAN20 | -2.11902 | 0.012676 | |
| ADRB1 | 8.45473 | 0.04775 | |
| FCGR3B | -1.00654 | 0.022892 | |
| NCRNA00081 | -2.07571 | 0.005756 | |
| PRICKLE2 | -8.67069 | 0.013824 | |
| NANS | 0.641698 | 0.044596 | |
| RPL24 | 8.394323 | 0.010964 | |
| GLT25D1 | 4.40999 | 0.020157 | |
| SLC16A11 | 5.95485 | 0.022171 | |
| ATG5 | -0.03175 | 0.040101 | |
| MPND | 15.23179 | 0.0001 | |
| USE1 | 3.471176 | 0.012676 | |
| CD24 | -7.133 | 0.002151 | |
| GABARAPL3 | 5.754718 | 0.029822 | |
| ZNF358 | 2.03862 | 0.002776 | |
| OAZ1 | 1.570093 | 0.039974 | |
| FXYD5 | 6.003232 | 0.025339 | |
| FXYD3 | 1.908018 | 0.019205 | |
| MAFK | 1.759673 | 0.043239 | |
| PLIN3 | 9.198908 | 0.000301 | |
| MED13 | -0.61931 | 0.029822 | |
| RBP7 | 2.892201 | 0.037211 | |
| KRAS | -2.27147 | 0.010964 | |
| TTC9 | -4.74359 | 0.005326 | |
| SMPX | 11.63291 | 0.043239 | |
| EEF1D | 1.82734 | 0.001839 | |
| C20orf165 | 2.133895 | 0.034132 | |
| RPL8 | 4.899037 | 0.002671 | |
| HR | 4.578535 | 0.019205 | |
| FEN1 | 2.080393 | 0.034392 | |
| ADAM9 | -2.64099 | 0.014869 | |
| INTS6 | -2.20669 | 0.038783 | |
| NR1I3 | -1.61232 | 0.032229 | |
| FLYWCH1 | 2.747 | 0.00773 | |
| ANKRD26 | -4.05915 | 0.030069 | |
| PTGES | 1.757674 | 0.011867 | |
| ACO2 | 1.552525 | 0.034782 | |
| TMEM66 | 2.814082 | 0.025598 | |
| ZNF70 | -2.67327 | 0.001631 | |
| LOC284232 | -4.65939 | 0.027562 | |
| PYROXD2 | 3.235809 | 0.022539 | |
| RPS14 | 5.862174 | 0.015215 | |
| OGFOD2 | 2.222485 | 0.004575 | |
| EID2 | 7.941453 | 0.013364 | |
| NEURL2 | 4.005821 | 0.001631 | |
| APAF1 | -6.18117 | 0.001839 | |
| RPL5 | 3.173612 | 0.045404 | |
| MANBAL | 4.371148 | 0.034918 | |
| ADO | -1.02782 | 0.005326 | |
| RASGRF2 | -4.71959 | 0.009727 | |
| MEIG1 | 4.655469 | 0.014057 | |
| TMC5 | -15.1079 | 0.000912 | |
| ZNF487 | -2.73154 | 0.001528 | |
| ZIC3 | 13.5543 | 0.041557 | |
| DECR2 | 2.02287 | 0.011187 | |
| ACVR1 | -3.07663 | 0.011302 | |
| JUND | 1.225082 | 0.024471 | |
| HIST1H2AH | 7.826536 | 0.037874 | |
| HIST1H2AE | 1.126387 | 0.003725 | |
| HIST1H2AD | 1.660227 | 0.041143 | |
| THAP11 | 2.092769 | 0.043371 | |
| PRPF19 | 0.85687 | 0.01429 | |
| TCFL5 | 6.511383 | 0.017318 | |
| FAM179B | -9.92287 | 0.000402 | |
| TRAF2 | 1.261665 | 0.03517 | |
| KILLIN | -3.62628 | 0.009388 | |
| MOCS1 | -5.06418 | 0.024966 | |
| SRP54 | -0.62069 | 0.011302 | |
| SLC33A1 | -0.36986 | 0.04623 | |
| COMMD8 | -2.87172 | 0.000301 | |
| COMMD2 | -0.53836 | 0.0151 | |
| COMMD3 | 6.740251 | 0.003831 | |
| COMMD1 | 3.505458 | 0.048026 | |
| COMMD4 | 6.460854 | 0.005109 | |
| COMMD5 | 8.514327 | 0.006631 | |
| ZMYM2 | -9.92056 | 0.000301 | |
| ZMYM4 | -5.4103 | 0.012446 | |
| WIPF2 | -0.50148 | 0.009951 | |
| SHFM1 | 7.983898 | 0.012101 | |
| JAK2 | -2.04626 | 0.004255 | |
| LPAR4 | -0.50399 | 0.006742 | |
| C20orf201 | 11.60343 | 0.018616 | |
| C6orf155 | -3.90311 | 0.006302 | |
| ANO6 | -3.60345 | 0.003938 | |
| POLR1A | -6.15375 | 0.002985 | |
| POLR1B | -0.40162 | 0.041932 | |
| HIP1R | 2.360492 | 0.031221 | |
| TCP11L1 | -2.4129 | 0.019323 | |
| DUS1L | 5.507138 | 0.000809 | |
| WDR44 | -7.93439 | 0.001943 | |
| GRM5 | -1.6941 | 0.041557 | |
| GRM6 | 7.702077 | 0.042586 | |
| SLC25A26 | 2.984181 | 0.001735 | |
| ZNF507 | -3.59395 | 0.019444 | |
| LOC729020 | -5.09142 | 0.033758 | |
| CHUK | -11.3803 | 0.000201 | |
| POLR2E | 1.564157 | 0.0435 | |
| EML3 | 0.44834 | 0.042069 | |
| EFCAB4A | 0.327761 | 0.049685 | |
| NCRNA00182 | -4.11261 | 0.042586 | |
| MKRN1 | 3.984559 | 0.00362 | |
| DYRK2 | -0.17479 | 0.04101 | |
| NR2F6 | 1.998111 | 0.029074 | |
| ELL2 | -3.22466 | 0.014752 | |
| RPIA | 6.791737 | 0.039434 | |
| HCRT | 14.7586 | 0.006631 | |
| TRPS1 | -9.60915 | 0.001014 | |
| CTTN | 10.93642 | 0.025838 | |
| FAM109A | 4.298163 | 0.045404 | |
| BSND | 10.08964 | 0.035801 | |
| EIF2S1 | -4.57733 | 0.010964 | |
| LPIN2 | -3.17985 | 0.045966 | |
| KLF3 | -3.68218 | 0.004469 | |
| EFR3A | -4.89713 | 0.019686 | |
| HEATR3 | -1.67425 | 0.014869 | |
| ASCC3 | -0.31489 | 0.04925 | |
| KLHDC7B | 6.293316 | 0.034651 | |
| PHF21A | -2.79218 | 0.009841 | |
| MAP3K12 | -8.35062 | 0.008504 | |
| MAP3K13 | -7.38329 | 0.004895 | |
| ADNP2 | -3.18747 | 0.030323 | |
| ATP13A2 | 1.561962 | 0.039834 | |
| RSPH4A | -6.01353 | 0.001839 | |
| FAM60A | -6.08106 | 0.010736 | |
| DCTPP1 | 2.913863 | 0.025472 | |
| RG9MTD2 | -0.87081 | 0.022418 | |
| DHRS7 | 4.501058 | 0.028689 | |
| DHRS4 | 3.579523 | 0.021449 | |
| DHRS2 | 2.626648 | 0.028074 | |
| DYRK1A | -2.49924 | 0.000503 | |
| CCNL1 | -4.88747 | 0.010515 | |
| NOMO3 | 2.849583 | 0.001631 | |
| NOMO1 | 4.657262 | 0.006192 | |
| IMPA2 | 2.189163 | 0.041419 | |
| PTGES2 | 2.417403 | 0.00288 | |
| SLC1A5 | 0.194836 | 0.033378 | |
| ZBTB8A | -4.46121 | 0.004469 | |
| CEACAM1 | -6.0548 | 0.000402 | |
| CEACAM6 | -5.30209 | 0.002671 | |
| RAPGEF4 | -6.86991 | 0.002255 | |
| KIAA0415 | 0.093319 | 0.028559 | |
| AGMAT | 1.699732 | 0.030069 | |
| RAPGEF6 | -4.42582 | 0.005756 | |
| TRNAU1AP | 2.013585 | 0.027949 | |
| MOCOS | 4.215535 | 0.014057 | |
| TMEM67 | -7.3364 | 0.003514 | |
| CYP4B1 | -3.9057 | 0.008504 | |
| TIGD5 | 5.906681 | 0.000201 | |
| ADHFE1 | 1.020137 | 0.017082 | |
| PPDPF | 1.983761 | 0.010625 | |
| H1FX | 6.939885 | 0.000201 | |
| VKORC1 | 4.388123 | 0.008172 | |
| ZNF214 | -5.17907 | 0.023257 | |
| PRKRA | -1.27192 | 0.012676 | |
| S1PR5 | 3.380277 | 0.049546 | |
| RASSF8 | -4.56399 | 0.023136 | |
| VEGFC | -3.93129 | 0.034392 | |
| LOC407835 | 1.813766 | 0.000201 | |
| SF3B3 | 3.74746 | 0.045554 | |
| ALOX12 | 2.575697 | 0.014175 | |
| SEMA6A | -4.03312 | 0.003408 | |
| ADCK5 | 3.703288 | 0.029074 | |
| ADCK2 | 3.767296 | 0.009841 | |
| ZBTB11 | -6.54186 | 0.000605 | |
| DNAJC10 | -5.11256 | 0.001322 | |
| EDF1 | 0.010644 | 0.006302 | |
| SUPT16H | -2.4228 | 0.042712 | |
| MLL | -2.1963 | 0.007949 | |
| LMAN1 | -2.56633 | 0.020502 | |
| MGC16384 | -4.67777 | 0.0235 | |
| LMAN2 | 2.900941 | 0.037478 | |
| NAT8L | 1.428023 | 0.039175 | |
| LOC642846 | 3.938516 | 0.010176 | |
| FAM195A | 2.867799 | 0.008834 | |
| ZNF17 | -0.65866 | 0.012676 | |
| ADD3 | -1.99515 | 0.035045 | |
| PPP4R1 | -2.46377 | 0.005435 | |
| PPP4R2 | -5.64122 | 0.013824 | |
| CWC22 | -3.25582 | 0.003198 | |
| MAP3K9 | -2.47728 | 0.020733 | |
| MAP3K2 | -3.28179 | 0.002567 | |
| MAP3K1 | -3.53736 | 0.024471 | |
| VPS54 | -3.132 | 0.002567 | |
| FNDC3A | -1.78906 | 0.034392 | |
| TLL1 | -9.28365 | 0.000402 | |
| PSRC1 | 2.774193 | 0.036066 | |
| SURF6 | 1.277934 | 0.030825 | |
| EME1 | 2.013991 | 0.011641 | |
| PCNXL2 | -4.61977 | 0.046926 | |
| ECH1 | 7.711455 | 0.001528 | |
| TAPBPL | 4.361447 | 0.006852 | |
| ACSF3 | 1.515477 | 0.018375 | |
| MAN2A1 | -10.7674 | 0.000707 | |
| FAM38A | 3.023053 | 0.018258 | |
| NDUFB10 | 2.32358 | 0.005974 | |
| FAM38B | -4.14552 | 0.014637 | |
| DECR1 | 1.868534 | 0.034264 | |
| CDHR3 | -1.04073 | 0.013018 | |
| C6orf170 | -2.03933 | 0.027432 | |
| TECTA | -5.97227 | 0.008615 | |
| PKM2 | 0.697082 | 0.040621 | |
| TMEM232 | -1.44256 | 0.036323 | |
| FARS2 | 0.087783 | 0.025472 | |
| RPS15A | 8.687133 | 0.001943 | |
| BTBD10 | -4.86645 | 0.010064 | |
| DUS3L | 2.592004 | 0.007178 | |
| EDA2R | -5.45533 | 0.003198 | |
| SALL2 | -8.10095 | 0.044065 | |
| FAIM | 4.335652 | 0.046926 | |
| C5orf36 | -9.56063 | 0.008282 | |
| C5orf33 | -4.00911 | 0.00784 | |
| FARSB | -0.52526 | 0.030069 | |
| FIS1 | 0.273797 | 0.003091 | |
| C9orf72 | -1.90801 | 0.0316 | |
| CUTA | 4.354314 | 0.019563 | |
| ZSCAN23 | -2.87529 | 0.000912 | |
| TTC33 | -2.24162 | 0.038396 | |
| MOSPD2 | -6.70112 | 0.027432 | |
| MOSPD1 | -0.57835 | 0.010849 | |
| PAN3 | -5.62386 | 0.005435 | |
| PCDHGA12 | -6.40532 | 0.022539 | |
| DPP8 | -1.96406 | 0.018616 | |
| POLE4 | 9.454051 | 0.001219 | |
| GBA | 4.892726 | 0.028195 | |
| DPP7 | 0.664159 | 0.003091 | |
| GLTSCR2 | 1.240027 | 0.027312 | |
| C11orf66 | -5.69673 | 0.011529 | |
| HS1BP3 | 8.377295 | 0.009612 | |
| NOC3L | -0.55628 | 0.01429 | |
| PCP2 | 3.266729 | 0.000605 | |
| HIAT1 | -5.88608 | 0.011418 | |
| TMEM167A | -0.90016 | 0.028945 | |
| CTRC | 12.06714 | 0.03957 | |
| TRMT61A | 1.800972 | 0.000201 | |
| DHX15 | -2.07845 | 0.011302 | |
| LDOC1 | 2.101418 | 0.015792 | |
| ZNF557 | -3.42152 | 0.025598 | |
| CCDC88C | -5.80895 | 0.049954 | |
| DGCR5 | 3.816431 | 0.010064 | |
| ZNF782 | -1.10349 | 0.011529 | |
| ZNF781 | -1.10865 | 0.024349 | |
| RNASE4 | -4.64584 | 0.013134 | |
| QTRT1 | 3.598844 | 0.000605 | |
| C18orf19 | -0.8192 | 0.007621 | |
| C22orf40 | 6.35036 | 0.024719 | |
| C18orf16 | -2.01184 | 0.000201 | |
| CTR9 | -2.29197 | 0.014985 | |
| SCARB1 | 0.489663 | 0.034264 | |
| ZNF561 | -1.08259 | 0.029567 | |
| CCR6 | -0.92714 | 0.01802 | |
| STMN2 | -3.94902 | 0.009612 | |
| DCLK1 | -4.55193 | 0.004895 | |
| NOX5 | 4.274246 | 0.027692 | |
| AZU1 | 11.18265 | 0.027825 | |
| FAM161A | -2.92203 | 0.027825 | |
| UBE3A | -2.71312 | 0.029201 | |
| MIAT | -6.26685 | 0.005866 | |
| IPW | -6.48276 | 0.025219 | |
| SDAD1 | -0.35825 | 0.014057 | |
| FGF7 | -0.74314 | 0.002151 | |
| TTYH1 | 9.707142 | 0.036066 | |
| SNF8 | 11.28286 | 0.000201 | |
| SPPL2B | 2.055584 | 0.001116 | |
| ZNF593 | 2.439184 | 0.030199 | |
| CWF19L1 | -2.8663 | 0.023014 | |
| SPRR2D | 15.55792 | 0.027825 | |
| NAA25 | -0.98699 | 0.006192 | |
| RILP | 2.650741 | 0.011984 | |
| CNKSR1 | 0.181866 | 0.019563 | |
| SGCB | -8.99404 | 0.000301 | |
| DUSP9 | 7.521445 | 0.00773 | |
| DUSP8 | -5.95679 | 0.016723 | |
| DUSP5 | -5.00591 | 0.040101 | |
| DUSP6 | -1.03163 | 0.015328 | |
| DUSP1 | -2.20247 | 0.026576 | |
| ELL | 0.85795 | 0.039974 | |
| HDX | -3.43422 | 0.014869 | |
| LRRN4CL | -2.97336 | 0.010176 | |
| MFSD9 | -2.29141 | 0.035546 | |
| STX4 | 0.53024 | 0.00362 | |
| MTF1 | -3.65298 | 0.006084 | |
| TAB3 | -4.69627 | 0.00806 | |
| CNTN1 | -5.86873 | 0.005326 | |
| FBL | 8.871197 | 0.004895 | |
| RPL35 | 3.43437 | 0.022291 | |
| AUTS2 | -4.92377 | 0.013249 | |
| PLA2G1B | 10.95975 | 0.014057 | |
| ARFGAP1 | 3.310396 | 0.031845 | |
| IKBKAP | -2.36093 | 0.004149 | |
| FAM83B | 1.08993 | 0.032102 | |
| FAM83A | 3.644034 | 0.048158 | |
| C12orf75 | -4.28373 | 0.026576 | |
| ATP7A | -3.04897 | 0.020853 | |
| C8orf4 | -4.19106 | 0.004788 | |
| DGKH | -2.9419 | 0.04379 | |
| DGKD | -1.6723 | 0.047891 | |
| SRP72 | -3.81828 | 0.011984 | |
| HSPA13 | -0.20232 | 0.002776 | |
| PEMT | 7.349738 | 0.001735 | |
| PKD2 | -4.8267 | 0.021685 | |
| ANXA8L1 | 19.8002 | 0.005217 | |
| DDX54 | 0.115788 | 0.000605 | |
| CSRNP3 | -1.41917 | 0.027692 | |
| CTAGE1 | -4.91999 | 0.032356 | |
| C10orf118 | -8.74462 | 0.000402 | |
| C10orf119 | -1.53315 | 0.004043 | |
| ZNF195 | -5.64006 | 0.003514 | |
| LRP6 | -6.08443 | 0.000707 | |
| CNDP1 | 5.787844 | 0.009059 | |
| AHR | -4.60523 | 0.01029 | |
| GADD45GIP1 | 1.183554 | 0.001943 | |
| COLEC12 | -7.85004 | 0.004469 | |
| PDS5A | -2.47756 | 0.048566 | |
| PDS5B | -2.71199 | 0.015908 | |
| TSTD2 | -4.71903 | 0.011073 | |
| NR2C2AP | 2.16628 | 0.035801 | |
| UBXN2B | -4.6371 | 0.004363 | |
| EDARADD | -11.1875 | 0.0001 | |
| ZNF225 | -5.43015 | 0.028195 | |
| KIF6 | -2.4788 | 0.007287 | |
| ALDH8A1 | -0.29755 | 0.019444 | |
| PLN | -3.24315 | 0.04623 | |
| RTTN | -3.85236 | 0.006084 | |
| OTUD5 | 3.512353 | 0.012676 | |
| AP3M1 | -4.39015 | 0.016025 | |
| AKAP10 | -4.30339 | 0.02882 | |
| AKAP11 | -5.59773 | 0.000809 | |
| MFSD6 | -3.58655 | 0.010736 | |
| S100Z | -1.99523 | 0.046364 | |
| SPCS2 | -6.61586 | 0.004788 | |
| UBAC1 | 4.703401 | 0.026332 | |
| ACER2 | -6.91405 | 0.0472 | |
| ACER3 | -1.9343 | 0.01429 | |
| ESYT1 | -3.1467 | 0.026453 | |
| CLRN3 | 6.439502 | 0.040879 | |
| APPBP2 | -0.01794 | 0.022418 | |
| E4F1 | 0.549035 | 0.033506 | |
| CRYBB1 | 3.736478 | 0.01992 | |
| EXOC1 | -3.1956 | 0.002255 | |
| MGAT4A | -2.83968 | 0.016254 | |
| C9orf95 | 3.471058 | 0.006962 | |
| TNRC6C | -5.06459 | 0.034264 | |
| USP25 | -5.18433 | 0.013018 | |
| TMEM134 | 3.478322 | 0.016602 | |
| ADAMTS3 | -1.16814 | 0.012216 | |
| ARHGEF3 | -5.64791 | 0.009059 | |
| CENPJ | -5.85982 | 0.034782 | |
| RAMP1 | 4.383769 | 0.033999 | |
| SSH1 | -6.97174 | 0.010402 | |
| CENPV | 0.739095 | 0.039307 | |
| SPRED1 | -3.68875 | 0.008282 | |
| ATP5G1 | 8.699996 | 0.017788 | |
| PHACTR2 | -1.46845 | 0.018733 | |
| PLEKHA3 | -4.29033 | 0.023136 | |
| NETO1 | -0.12206 | 0.009841 | |
| PLEKHA9 | -6.10776 | 0.001631 | |
| SEC62 | -0.60296 | 0.011418 | |
| DHX38 | 3.065848 | 0.048849 | |
| DHX34 | 3.496066 | 0.028689 | |
| DHX36 | -1.70001 | 0.015562 | |
| ZNF765 | -3.82706 | 0.001735 | |
| LOC100009676 | 4.586118 | 0.040621 | |
| ZNF767 | -7.10819 | 0.019563 | |
| ZNF766 | -0.89844 | 0.006413 | |
| TPT1 | 4.315541 | 0.01256 | |
| RPP21 | 2.364988 | 0.041273 | |
| RPP25 | 5.30165 | 0.005004 | |
| ECSIT | 3.719911 | 0.003938 | |
| DOPEY1 | -2.85949 | 0.00917 | |
| TREM2 | 0.217566 | 0.0472 | |
| SMAD5 | -7.1026 | 0.001839 | |
| RPL41 | 7.375236 | 0.003725 | |
| C9orf53 | 10.57853 | 0.045554 | |
| KRTAP5-6 | 22.341 | 0.024966 | |
| CEP250 | 0.656916 | 0.005004 | |
| CELF1 | -9.17152 | 0.000809 | |
| UHRF2 | -1.82675 | 0.002567 | |
| FAM149B1 | -0.15433 | 0.020853 | |
| KEAP1 | 4.014779 | 0.001014 | |
| SLC5A4 | -2.89094 | 0.009059 | |
| TIA1 | -3.25104 | 0.017318 | |
| UBN2 | -5.00582 | 0.01568 | |
| UBN1 | -0.79183 | 0.031477 | |
| RAVER1 | 7.054305 | 0.005866 | |
| RB1 | -10.0104 | 0.034392 | |
| ZBTB2 | -1.67577 | 0.015442 | |
| ZBTB1 | -5.05775 | 0.00362 | |
| CASC4 | -4.9503 | 0.018975 | |
| COL24A1 | -5.27786 | 0.030576 | |
| TAF1 | -3.92576 | 0.027312 | |
| BOLA1 | 7.594985 | 0.00773 | |
| C21orf82 | 3.739446 | 0.025339 | |
| C21orf84 | 4.995822 | 0.026818 | |
| NDUFB7 | 1.420088 | 0.005542 | |
| C21orf88 | -3.9387 | 0.000402 | |
| FAM160A2 | -7.90831 | 0.004788 | |
| C12orf57 | 1.466469 | 0.035926 | |
| C12orf51 | -4.07658 | 0.017318 | |
| STAP2 | 3.689954 | 0.004575 | |
| SH3BP4 | -6.40942 | 0.022539 | |
| ANKRD49 | -2.35351 | 0.022418 | |
| SCARNA5 | 7.94156 | 0.021092 | |
| SCARNA2 | 5.543498 | 0.00784 | |
| PHB | 11.71364 | 0.001631 | |
| GPR150 | 7.932563 | 0.010176 | |
| GSC | 3.987843 | 0.011984 | |
| PGGT1B | -3.01148 | 0.029322 | |
| SNHG6 | 4.310102 | 0.020853 | |
| RNF213 | -3.57109 | 0.022892 | |
| FUNDC2 | 3.402874 | 0.005217 | |
| STX2 | -9.84799 | 0.011867 | |
| ADORA1 | 8.966307 | 0.000605 | |
| CC2D1A | 5.734431 | 0.003091 | |
| 7-Sep | -5.59612 | 0.003938 | |
| C10orf137 | -4.61922 | 0.009498 | |
| YPEL2 | -4.69577 | 0.021925 | |
| GSN | 0.196876 | 0.041143 | |
| FGF22 | 13.73263 | 0.022418 | |
| ATP8B2 | -4.93314 | 0.010402 | |
| SH3GL1 | 4.55653 | 0.001014 | |
| SLC10A3 | 4.48329 | 0.013939 | |
| MPST | 2.601394 | 0.005542 | |
| FHL2 | -4.23088 | 0.027949 | |
| UGGT1 | -3.21905 | 0.008615 | |
| ZNF248 | -6.59661 | 0.000402 | |
| VASH2 | -5.56066 | 0.001528 | |
| HMGXB3 | -8.79051 | 0.000707 | |
| DIP2A | -2.21121 | 0.009612 | |
| DIP2B | -1.88797 | 0.036198 | |
| DIP2C | -1.81341 | 0.039834 | |
| MYLPF | 11.05753 | 0.02509 | |
| YES1 | -4.97492 | 0.006523 | |
| STAM2 | -8.80249 | 0.004363 | |
| ZCCHC4 | -2.17225 | 0.042069 | |
| C17orf68 | -7.78309 | 0.001322 | |
| EFCAB4B | -6.20027 | 0.017552 | |
| ARID3B | -5.3769 | 0.023136 | |
| C6orf138 | -3.06817 | 0.021092 | |
| PUS7L | -8.4534 | 0.001322 | |
| MUDENG | -6.13237 | 0.024596 | |
| ADPRHL1 | 3.711768 | 0.000503 | |
| ATP2B1 | -6.79987 | 0.009612 | |
| C19orf47 | 7.2808 | 0.016723 | |
| CAPN7 | -0.79639 | 0.011641 | |
| CAPN1 | 2.281734 | 0.009727 | |
| SPAG7 | 0.453825 | 0.016602 | |
| SLC2A12 | -0.09973 | 0.04101 | |
| HTR7 | -4.43058 | 0.011302 | |
| KLHL2 | -0.39252 | 0.022171 | |
| KLHL3 | -5.5672 | 0.001219 | |
| WAC | -0.08705 | 0.033758 | |
| KLHL9 | -3.43185 | 0.006742 | |
| FUT11 | -3.7161 | 0.004469 | |
| ZNF696 | 6.610485 | 0.01279 | |
| MKRN3 | 4.343982 | 0.016487 | |
| KATNA1 | -1.74604 | 0.018733 | |
| KDELR3 | 0.797816 | 0.042452 | |
| HIRIP3 | 1.484677 | 0.000503 | |
| ZFP30 | -4.48108 | 0.003198 | |
| ZNF354A | -0.63783 | 0.022892 | |
| KIAA0947 | -2.93375 | 0.001735 | |
| CMAH | -3.30801 | 0.011984 | |
| WHAMML1 | -10.2305 | 0.003514 | |
| TMEM11 | 6.351557 | 0.006523 | |
| PLEC | 2.634448 | 0.034524 | |
| ZNF546 | -4.03114 | 0.020976 | |
| KDM5A | -1.91572 | 0.022656 | |
| TMEM18 | 8.82469 | 0.000809 | |
| NDUFA11 | 6.857887 | 0.000605 | |
| PRDM13 | 14.52289 | 0.020157 | |
| PHLDB2 | -3.81598 | 0.016723 | |
| AGTRAP | 3.708793 | 0.005109 | |
| MTP18 | 4.745631 | 0.022774 | |
| DDB1 | 0.666662 | 0.037478 | |
| ZNF527 | -5.86794 | 0.020271 | |
| ZNF525 | -0.79449 | 0.044991 | |
| YJEFN3 | 2.073776 | 0.025717 | |
| AVL9 | -5.22698 | 0.049407 | |
| UBQLN2 | -4.57445 | 0.006413 | |
| DAPK3 | 3.772942 | 0.001839 | |
| DPY19L3 | -6.00986 | 0.003514 | |
| MYNN | -0.94327 | 0.025472 | |
| UBL5 | 5.563171 | 0.022044 | |
| BPTF | -3.37543 | 0.044206 | |
| UBL7 | 1.120573 | 0.025219 | |
| UBL3 | -7.58038 | 0.011302 | |
| FKBP2 | 0.628509 | 0.001528 | |
| FKBP8 | 0.087522 | 0.038396 | |
| GABARAP | 2.391618 | 0.006413 | |
| FCHO1 | 1.742549 | 0.017902 | |
| BET1L | -6.78007 | 0.016025 | |
| GRB10 | -4.50624 | 0.031477 | |
| FDXACB1 | -2.85649 | 0.024223 | |
| GPR172A | 5.218314 | 0.025219 | |
| CSRP1 | -2.01471 | 0.029943 | |
| ZNF460 | -0.68866 | 0.0001 | |
| HSD17B14 | 4.504874 | 0.033127 | |
| SLC26A6 | 3.391167 | 0.024719 | |
| PIWIL1 | -0.55806 | 0.000201 | |
| PANK3 | -7.49086 | 0.001735 | |
| PANK2 | -1.88358 | 0.013249 | |
| ETAA1 | -7.48346 | 0.009951 | |
| KIF2A | -6.87558 | 0.001116 | |
| PADI1 | 14.13373 | 0.001425 | |
| HEATR5A | -4.59155 | 0.000503 | |
| NFASC | -2.02729 | 0.042452 | |
| TMEM22 | -0.29211 | 0.047891 | |
| ZNRF3 | -0.70728 | 0.034782 | |
| CBL | -4.24714 | 0.017788 | |
| GPX1 | 1.489435 | 0.005756 | |
| GPX4 | 13.45871 | 0.0001 | |
| NPDC1 | 1.070769 | 0.048979 | |
| TNFAIP6 | -4.9 | 0.013134 | |
| PLCL1 | -2.4964 | 0.031221 | |
| PRKCSH | 5.693686 | 0.000402 | |
| STT3B | -5.13814 | 0.048566 | |
| RBM16 | -8.58793 | 0.0001 | |
| HSD11B2 | 2.687959 | 0.037748 | |
| KCTD11 | 1.32996 | 0.043928 | |
| CXorf27 | 16.95207 | 0.024596 | |
| CXorf23 | -13.1062 | 0.0001 | |
| ZFYVE21 | 1.024605 | 0.049815 | |
| RFT1 | 2.830914 | 0.03645 | |
| LRRC14 | 1.563845 | 0.01429 | |
| PSMD4 | 4.894327 | 0.004788 | |
| PSMD7 | 2.368504 | 0.013249 | |
| ZNF12 | -3.14704 | 0.027184 | |
| WDR33 | -0.36371 | 0.049111 | |
| PLEKHG4B | -5.53142 | 0.029074 | |
| BAT4 | 2.220398 | 0.007399 | |
| BANP | 1.521335 | 0.040879 | |
| KIAA1737 | -2.86638 | 0.044736 | |
| SCN8A | -5.21762 | 0.008282 | |
| RGAG4 | -9.36572 | 0.00468 | |
| KIAA1731 | -3.43386 | 0.028559 | |
| COPS7A | 0.100205 | 0.027312 | |
| DKK3 | -3.97486 | 0.031978 | |
| HPS3 | -3.17088 | 0.048425 | |
| NFE2L2 | -3.97579 | 0.01429 | |
| ZNF264 | -0.88351 | 0.000503 | |
| ZNF260 | -1.5467 | 0.001528 | |
| LOC440905 | -5.55287 | 0.011984 | |
| SCAND3 | -1.84411 | 0.040236 | |
| ROBLD3 | 2.631908 | 0.015908 | |
| ZC3H11A | -1.30371 | 0.035546 | |
| SIGLEC16 | -2.33811 | 0.016602 | |
| ARID1B | -6.8613 | 0.000503 | |
| LOC100130776 | 1.643602 | 0.016025 | |
| RHOD | 3.384391 | 0.005435 | |
| DHRS4L2 | 3.843551 | 0.044596 | |
| YIPF1 | -2.63354 | 0.028945 | |
| YIPF5 | -6.71881 | 0.000912 | |
| CYB5R3 | 3.350497 | 0.035422 | |
| RBBP5 | -0.30728 | 0.036951 | |
| TCEANC | -3.98492 | 0.034524 | |
| BTF3L4 | -0.84211 | 0.025598 | |
| C19orf62 | 5.482262 | 0.011867 | |
| C19orf60 | 2.724853 | 0.001735 | |
| C11orf52 | 2.540031 | 0.034524 | |
| PRR24 | 2.90474 | 0.032739 | |
| SORL1 | -2.82593 | 0.034264 | |
| NLGN4X | -5.84975 | 0.002151 | |
| RAG1 | -5.2171 | 0.011187 | |
| EIF4H | 5.666774 | 0.024471 | |
| EIF4E | -4.45774 | 0.012333 | |
| HDAC5 | 3.426668 | 0.002567 | |
| HDAC4 | -2.66399 | 0.015442 | |
| TMEM161A | 3.853451 | 0.038003 | |
| KCTD16 | -3.38953 | 0.004895 | |
| CDT1 | 0.322629 | 0.014175 | |
| CSTB | 7.532261 | 0.013595 | |
| CMC1 | 4.71287 | 0.006962 | |
| JOSD2 | 0.948161 | 0.009727 | |
| ENDOD1 | 3.393325 | 0.042069 | |
| AMIGO1 | -3.01145 | 0.03957 | |
| CDYL2 | -5.47228 | 0.0435 | |
| USP12 | -9.26064 | 0.0001 | |
| USP5 | 1.550376 | 0.049815 | |
| MRM1 | 0.970529 | 0.043102 | |
| ZNF721 | -4.75127 | 0.046509 | |
| CEBPA | 2.98821 | 0.006302 | |
| CEBPD | 2.203029 | 0.010515 | |
| XRCC5 | -1.99642 | 0.047891 | |
| LOC100131691 | 1.569321 | 0.041419 | |
| CEBPZ | -0.51566 | 0.00917 | |
| PITPNB | -1.53406 | 0.024596 | |
| C14orf33 | -0.24636 | 0.029567 | |
| MTHFS | 2.930483 | 0.009612 | |
| TRIO | -6.76067 | 0.003831 | |
| SLC1A1 | -4.28512 | 0.006962 | |
| RNF138P1 | -4.55826 | 0.00751 | |
| EPB41L4A | -11.5757 | 0.011187 | |
| MYL6 | 2.026804 | 0.042452 | |
| LINGO3 | 3.563166 | 0.011984 | |
| TSNARE1 | 7.997397 | 0.006084 | |
| CHSY1 | -3.34308 | 0.022656 | |
| SFTPC | 21.0085 | 0.016139 | |
| MPP5 | -9.87624 | 0.008504 | |
| MAPK8IP1 | -1.64419 | 0.018375 | |
| FAM110B | -5.38439 | 0.047478 | |
| GP9 | 16.7311 | 0.018975 | |
| EXTL2 | -3.55337 | 0.008394 | |
| STAU2 | -1.27658 | 0.036066 | |
| GPT | 3.893706 | 0.01279 | |
| LOC440354 | -5.49219 | 0.028945 | |
| MAN1A1 | -3.74101 | 0.037211 | |
| IKBKG | 1.259275 | 0.00288 | |
| CRTC2 | 5.323816 | 0.018616 | |
| FAM13B | -5.32787 | 0.049546 | |
| SUMF2 | 10.02764 | 0.000201 | |
| SDHAF2 | 0.555556 | 0.030958 | |
| FNDC3B | -0.99017 | 0.040101 | |
| SURF2 | 0.855135 | 0.001839 | |
| ACSF2 | 4.391075 | 0.000201 | |
| GRLF1 | -3.8094 | 0.046509 | |
| CCDC130 | 2.932465 | 0.017788 | |
| GALK1 | 3.441481 | 0.000707 | |
| WIZ | 3.097095 | 0.040879 | |
| POLE3 | 7.337073 | 0.000605 | |
| NXNL2 | 0.928962 | 0.018975 | |
| STARD4 | -4.11689 | 0.018975 | |
| APH1A | 1.847123 | 0.027825 | |
| STAT2 | -5.56458 | 0.005756 | |
| HEATR7A | 5.451234 | 0.008394 | |
| HTATSF1 | 6.05626 | 0.024223 | |
| AKR1B1 | 1.30169 | 0.025963 | |
| BTAF1 | -1.93779 | 0.001943 | |
| MAP3K10 | 0.718408 | 0.005004 | |
| PDE4B | -4.51196 | 0.035295 | |
| PDE4D | -1.40261 | 0.011641 | |
| PEG3 | -4.99248 | 0.000402 | |
| JAKMIP3 | -3.80625 | 0.030825 | |
| LOC642826 | -6.79828 | 0.016372 | |
| TRAPPC6B | -8.63522 | 0.000809 | |
| NR1H3 | 3.215103 | 0.016964 | |
| SOCS7 | -0.92734 | 0.01802 | |
| TPRG1 | 2.162824 | 0.030958 | |
| TMEM231 | -2.39213 | 0.047335 | |
| SPANXB2 | 15.38528 | 0.031477 | |
| PHF6 | -6.25071 | 0.001322 | |
| PPP2R5E | -4.08964 | 0.003302 | |
| ALDOC | -3.39252 | 0.024471 | |
| SLC2A4RG | 2.288744 | 0.021807 | |
| SNORD116-4 | -13.1027 | 0.000503 | |
| EP400NL | -4.33573 | 0.013939 | |
| KIAA1715 | -0.98467 | 0.011984 | |
| GLI4 | 5.640596 | 0.002462 | |
| ENPP1 | -10.217 | 0.013939 | |
| GIPC1 | 3.819243 | 0.026332 | |
| FZR1 | 0.407192 | 0.006962 | |
| CD2AP | -5.6981 | 0.000912 | |
| ZZEF1 | -8.5208 | 0.006742 | |
| CS | -1.10879 | 0.037076 | |
| GLUL | 2.289575 | 0.035674 | |
| LONP1 | 2.440491 | 0.008615 | |
| DNM2 | 7.87875 | 0.000402 | |
| ZKSCAN1 | -4.07314 | 0.003408 | |
| ZNF630 | -7.12877 | 0.041681 | |
| CNFN | 2.496211 | 0.00362 | |
| C9orf102 | -4.88057 | 0.022044 | |
| ZNF160 | -2.60836 | 0.006413 | |
| ZNF281 | -3.93243 | 0.011984 | |
| CNIH | -0.51907 | 0.046646 | |
| EVPL | 3.619786 | 0.000707 | |
| STRN3 | -16.3923 | 0.0001 | |
| PCNP | -2.86674 | 0.001631 | |
| BTBD9 | -2.83098 | 0.04761 | |
| BTBD6 | 1.271692 | 0.013939 | |
| BTBD3 | -2.89563 | 0.001219 | |
| BTBD2 | 1.077047 | 0.008615 | |
| RPL29P2 | 11.26921 | 0.018616 | |
| IKZF5 | -3.06519 | 0.005326 | |
| SPATS2 | -0.29311 | 0.014404 | |
| NFIA | -8.19144 | 0.013824 | |
| TCEAL7 | -3.47598 | 0.008615 | |
| TCEAL8 | -5.60759 | 0.04623 | |
| RGS16 | -2.87915 | 0.008172 | |
| MINA | -8.84585 | 0.013018 | |
| STRA13 | 1.261625 | 0.005542 | |
| IGFL2 | -1.47871 | 0.003091 | |
| BTN2A1 | -2.99855 | 0.022539 | |
| ELF4 | 5.766274 | 0.048566 | |
| CAND1 | -0.78319 | 0.003198 | |
| BRPF3 | -2.5964 | 0.021571 | |
| LDHD | 3.936293 | 0.001943 | |
| ACAP2 | -1.36574 | 0.006852 | |
| C8orf84 | -2.10323 | 0.003408 | |
| NPY5R | 6.582124 | 0.041419 | |
| GSTK1 | 1.243948 | 0.028689 | |
