# Supplementary material for: Identification of breast cancer recurrence risk factors based on functional pathways in tumor and normal tissues
Source: Oncotarget. 2016 Aug 23;8(13):20679–94. doi: 10.18632/oncotarget.11557 (PMC5400536; doi:10.18632/oncotarget.11557)
Supplement: Supplementary file 4 [file oncotarget-08-20679-s004.docx]

Additional file 3:

| gene | score | adj.P |
| --- | --- | --- |
| RNF11 | -1.19453 | 0.008556 |
| REM1 | 0.799447 | 0.023433 |
| FGFR1OP2 | -6.1437 | 0.001835 |
| PMM2 | 5.50952 | 0.041916 |
| HAP1 | -9.99152 | 0.029911 |
| COL7A1 | -15.8027 | 0.008337 |
| SPPL2A | -5.11531 | 0.009212 |
| STK40 | 6.107982 | 0.014682 |
| ITGA3 | -7.51823 | 0.048086 |
| DENND4B | -3.41354 | 0.021895 |
| LOC100132287 | -3.89038 | 0.018136 |
| FBXL13 | -5.74409 | 0.003604 |
| FBXL17 | -3.60229 | 0.028054 |
| FAM169A | -9.07426 | 0.002665 |
| GHDC | 2.597557 | 0.006595 |
| SDK2 | -12.0269 | 0.048086 |
| COL4A5 | -10.1442 | 0.023789 |
| COL4A3 | -7.67582 | 0.023911 |
| SLC35D2 | -3.75138 | 0.012974 |
| CHST4 | -1.30763 | 0.044891 |
| LIPG | -14.122 | 0.00225 |
| FAM134C | 3.860398 | 0.029911 |
| SERPING1 | -8.20427 | 0.022717 |
| TMPRSS11D | 19.29009 | 0.002354 |
| ZC3H10 | 3.226273 | 0.006595 |
| C14orf115 | 16.1742 | 0.017212 |
| ZC3H15 | -3.48832 | 0.043209 |
| TCEA3 | 6.155439 | 0.006051 |
| MGC57346 | -5.07067 | 0.012304 |
| COX4I2 | 0.297766 | 0.043084 |
| CCDC110 | -6.84632 | 0.010422 |
| CCDC111 | -3.85567 | 0.002042 |
| KGFLP2 | -6.67154 | 0.016521 |
| CRTAP | 2.196749 | 0.044634 |
| CPEB2 | -5.52501 | 0.02474 |
| BEGAIN | -6.37245 | 0.04036 |
| OSGEP | 5.679353 | 0.014801 |
| TLN2 | 2.709276 | 0.03591 |
| IFT80 | -4.22962 | 0.002977 |
| SP4 | -6.28552 | 0.01629 |
| TMEM38B | -8.96406 | 0.002769 |
| CFH | -4.72207 | 0.02635 |
| CFI | -5.61056 | 0.042306 |
| PHYHIPL | -9.44179 | 0.005087 |
| ATP6V0E2 | 4.347271 | 0.009323 |
| RNF128 | -14.7953 | 0.004023 |
| AAAS | 6.919876 | 0.036784 |
| CNPY3 | 1.347998 | 0.014801 |
| CNPY2 | 5.944186 | 0.01377 |
| KNDC1 | -2.7105 | 0.035641 |
| HMG20B | 5.286365 | 0.001835 |
| C3orf75 | 4.555561 | 0.010641 |
| EFHA1 | -0.13778 | 0.011529 |
| EFHA2 | -10.9846 | 0.017212 |
| ZNF222 | -1.79678 | 0.005408 |
| TIGD4 | -5.83046 | 0.032516 |
| GGCX | 3.250496 | 0.021778 |
| TMEM5 | -1.65627 | 0.048479 |
| KHDRBS1 | -5.37707 | 0.015606 |
| TMEM211 | 10.76951 | 0.025241 |
| TTC7A | 2.308437 | 0.008117 |
| UQCRHL | 7.112827 | 0.00256 |
| C11orf2 | 3.864228 | 0.012414 |
| OSTM1 | -1.78875 | 0.009875 |
| RUNX2 | -4.61504 | 0.036409 |
| C15orf29 | -3.81165 | 0.019306 |
| STK17A | -2.49695 | 0.00573 |
| KDM3A | -8.65273 | 0.000503 |
| TMC1 | 4.086862 | 0.038409 |
| EIF2B4 | 5.804228 | 0.018366 |
| EIF2B5 | 5.003438 | 0.003081 |
| SLC36A4 | -8.29153 | 0.046338 |
| ZNF876P | -4.60277 | 0.021778 |
| MLF1 | -1.75231 | 0.004555 |
| C9orf122 | -14.2549 | 0.019776 |
| RIT1 | -0.2476 | 0.007791 |
| TMEM141 | 2.701751 | 0.033627 |
| RTKN2 | -1.61611 | 0.040752 |
| P2RX7 | -7.81338 | 0.026725 |
| SBF1P1 | 3.486913 | 0.043857 |
| FAM32A | 3.885686 | 0.013314 |
| KIAA1191 | -5.77283 | 0.000503 |
| ALOX12P2 | -7.12424 | 0.02733 |
| FER1L5 | -0.99752 | 0.044504 |
| C19orf21 | -4.98944 | 0.022361 |
| ZMAT4 | -0.63416 | 0.038035 |
| C19orf29 | 0.000163 | 0.01175 |
| ITGB1BP1 | 5.500552 | 0.016636 |
| RPL31 | -3.24954 | 0.018714 |
| AP2A2 | 2.819978 | 0.029407 |
| CADPS | -1.99557 | 0.031659 |
| NSUN7 | -12.1716 | 0.014801 |
| NSUN6 | -5.03419 | 0.018136 |
| FAM66C | -9.14697 | 0.005514 |
| CALM1 | 7.68197 | 0.031029 |
| UPK1B | 21.05213 | 0.001526 |
| DLGAP1 | -12.9844 | 0.006377 |
| MBNL2 | -7.93674 | 0.044117 |
| PLK3 | -6.78367 | 0.00573 |
| TTC8 | -13.8824 | 0.000301 |
| ZNF720 | -1.78604 | 0.001217 |
| PCDHGA1 | -6.29498 | 0.049145 |
| FBXL18 | 0.290196 | 0.004979 |
| BRD4 | 2.063117 | 0.026974 |
| DNAJA2 | -1.43343 | 0.020705 |
| PTPN12 | -3.3195 | 0.011639 |
| CH25H | -9.84458 | 0.001114 |
| MRPL27 | 7.34275 | 0.048882 |
| C16orf72 | -2.50034 | 0.028796 |
| C2orf84 | 3.274601 | 0.01629 |
| C2orf86 | 4.56661 | 0.028676 |
| CHD1L | 1.48488 | 0.031786 |
| DNAJB13 | -0.48805 | 0.039971 |
| RNF138 | -2.29817 | 0.021895 |
| CISD1 | -0.20274 | 0.041524 |
| TIPARP | -1.47224 | 0.008775 |
| TMEM115 | 3.231554 | 0.045019 |
| CAMKK2 | 5.209883 | 0.008556 |
| CAMKK1 | 0.920049 | 0.03591 |
| TMEM123 | -3.30682 | 0.024994 |
| MTRF1L | -6.30838 | 0.002042 |
| MYO1D | -3.78562 | 0.045294 |
| MYO1C | 5.253644 | 0.029165 |
| RPL36AL | 4.370752 | 0.040623 |
| CHMP7 | 0.750533 | 0.000301 |
| BCR | 2.007101 | 0.005621 |
| HOXA11 | 2.04169 | 0.028181 |
| GALNTL6 | -0.50081 | 0.01434 |
| LSM11 | -4.80918 | 0.003709 |
| SUN1 | -7.74625 | 0.046338 |
| LGALS1 | 2.959762 | 0.01343 |
| TDG | -1.62485 | 0.000402 |
| EREG | -1.4672 | 0.005514 |
| KIAA1522 | -12.7156 | 0.023433 |
| MPDU1 | 7.183994 | 0.007138 |
| LOX | -5.28082 | 0.04727 |
| C14orf138 | -7.52233 | 0.000706 |
| C14orf135 | -2.55267 | 0.019306 |
| LPCAT3 | -3.08553 | 0.01503 |
| PIGA | -5.62484 | 0.000706 |
| CALCRL | -1.82107 | 0.022717 |
| PIGH | -3.24718 | 0.024499 |
| NDFIP2 | -5.05842 | 0.005301 |
| PDK1 | -5.48354 | 0.00256 |
| PTGS2 | -12.872 | 0.0001 |
| DDHD2 | -2.30674 | 0.028676 |
| MGST3 | 4.404938 | 0.029783 |
| ZFAND1 | -1.87447 | 0.012191 |
| MYO5B | -11.8302 | 0.03141 |
| SLC39A10 | -3.79317 | 0.018948 |
| SLC39A14 | -3.49135 | 0.025484 |
| CEP120 | -5.24974 | 0.029407 |
| DDIT3 | -0.23489 | 0.020705 |
| DDIT4 | 4.27708 | 0.02635 |
| NKRF | -2.40737 | 0.025609 |
| PPPDE1 | -3.29298 | 0.009656 |
| BMP2 | -6.65488 | 0.03489 |
| GPR4 | -2.52829 | 0.036663 |
| FEM1C | -5.40135 | 0.049921 |
| TERF1 | -9.81968 | 0.0001 |
| NDUFAF1 | 5.461367 | 0.026974 |
| FEM1A | 4.525281 | 0.006703 |
| LOC283050 | -4.33498 | 0.007138 |
| DYNC1LI2 | 2.72502 | 0.016059 |
| EFHC2 | -4.27329 | 0.042692 |
| PLSCR1 | -3.48872 | 0.006377 |
| MPZ | 5.222352 | 0.022127 |
| AKR1B10 | 16.06211 | 0.000604 |
| C4orf36 | -5.55298 | 0.030665 |
| C4orf33 | -2.43729 | 0.002872 |
| ZFP1 | -4.07484 | 0.048086 |
| C4orf39 | -7.36459 | 0.038677 |
| C10orf99 | 26.3354 | 0.001629 |
| C10orf93 | -5.31168 | 0.030532 |
| C15orf41 | -7.31482 | 0.036913 |
| ADH7 | 28.63071 | 0.001217 |
| ADH4 | -2.78178 | 0.046464 |
| ZNF138 | -2.24082 | 0.031659 |
| SPRYD3 | 7.768755 | 0.000201 |
| PSMB7 | 6.042133 | 0.047815 |
| PSMB6 | 2.398896 | 0.046338 |
| PSMB1 | 3.333643 | 0.02474 |
| PFKFB2 | -1.01969 | 0.016754 |
| HDHD3 | 1.172713 | 0.007898 |
| LRRC23 | 3.671154 | 0.046212 |
| KCNK5 | 2.441893 | 0.005087 |
| BDNFOS | -6.8794 | 0.043341 |
| CRY1 | -1.62379 | 0.04727 |
| FAM155A | -3.41903 | 0.011196 |
| BEX5 | -9.79541 | 0.014913 |
| BEX4 | -11.5203 | 0.004343 |
| BEX2 | -16.1694 | 0.011862 |
| BEX1 | -1.3122 | 0.039571 |
| FOXN2 | -3.58428 | 0.01175 |
| GRIN2B | 0.754504 | 0.046731 |
| GNA13 | -3.73273 | 0.011418 |
| PRKAR1A | -4.11541 | 0.004343 |
| SBF2 | -7.61359 | 0.032895 |
| OGDH | 4.601461 | 0.037786 |
| ADD1 | 3.704329 | 0.023313 |
| CDC73 | -6.77779 | 0.029911 |
| EN2 | -2.92683 | 0.045294 |
| LOC100134713 | -8.45092 | 0.000604 |
| RPL13AP20 | 3.328636 | 0.028676 |
| SRFBP1 | -3.46631 | 0.017441 |
| BCAT2 | 2.634231 | 0.043341 |
| BCAT1 | -4.33612 | 0.014801 |
| LOC283267 | -9.40468 | 0.001526 |
| AKAP6 | -6.19522 | 0.038809 |
| DBNL | 1.433585 | 0.009987 |
| AKAP8 | -4.36808 | 0.044117 |
| PPIP5K1 | -2.96769 | 0.019541 |
| TUBE1 | -10.3103 | 0.000706 |
| CETN2 | 5.608343 | 0.019306 |
| DIO3 | -0.7186 | 0.049404 |
| ALPK2 | 0.962846 | 0.039436 |
| TRIOBP | 0.136275 | 0.047815 |
| C8orf47 | -5.6121 | 0.039971 |
| C8orf42 | -12.5871 | 0.004661 |
| BRF1 | 0.123523 | 0.011082 |
| DNAJC9 | -5.92499 | 0.041524 |
| DNAJC5 | 3.208863 | 0.026597 |
| DNAJC6 | -7.74876 | 0.000301 |
| GOSR2 | 0.235438 | 0.006161 |
| SLC18A2 | -3.13175 | 0.040493 |
| GOSR1 | -2.12455 | 0.022717 |
| CCDC97 | 2.245665 | 0.005621 |
| RALBP1 | 7.258399 | 0.001217 |
| FAM91A1 | -3.66805 | 0.000503 |
| PMEPA1 | 8.139566 | 0.008447 |
| TRIM8 | 5.689603 | 0.03141 |
| LOC643719 | 3.491571 | 0.024499 |
| RAB33A | -1.21764 | 0.031285 |
| CP110 | -5.28869 | 0.000604 |
| SLC17A7 | 0.102781 | 0.026725 |
| MDH1B | -11.1621 | 0.012414 |
| SETDB2 | -4.03814 | 0.003499 |
| C16orf52 | -13.2098 | 0.000201 |
| ITCH | -7.81718 | 0.043724 |
| ZNF207 | -0.90597 | 0.022958 |
| BHMT2 | -5.50618 | 0.025484 |
| CDK19 | -1.15761 | 0.003604 |
| GFI1B | 5.899947 | 0.033133 |
| SRRT | 0.177505 | 0.002872 |
| LOC283856 | -2.08375 | 0.024864 |
| GZF1 | -1.88318 | 0.000503 |
| STS | 4.220625 | 0.042558 |
| MMP24 | 0.823245 | 0.010752 |
| PIH1D1 | 8.975037 | 0.001012 |
| NCL | 10.51585 | 0.000604 |
| CTNNBIP1 | 1.981898 | 0.018366 |
| ZNF821 | 0.59322 | 0.023193 |
| LIF | -12.722 | 0.0001 |
| UBTF | 2.676779 | 0.000301 |
| NCF1 | 0.033352 | 0.021431 |
| LRRC47 | 8.483579 | 0.005408 |
| LRRC49 | -6.12744 | 0.006269 |
| GPATCH4 | 0.744951 | 0.019188 |
| B3GNT4 | -4.33 | 0.037035 |
| FNDC8 | 2.588981 | 0.023433 |
| PWWP2B | 0.604166 | 0.031152 |
| PWWP2A | -3.71934 | 0.013088 |
| PCYT1A | 4.709802 | 0.029783 |
| PWP2 | 2.407265 | 0.001939 |
| MTCH2 | 8.582383 | 0.015606 |
| OBFC2A | -6.56023 | 0.006051 |
| SLC9A3R2 | 1.59654 | 0.008117 |
| FAM189A2 | -11.6883 | 0.033873 |
| NES | 2.431218 | 0.010641 |
| OXT | 12.69357 | 0.005621 |
| C1orf52 | -5.64374 | 0.001217 |
| C1orf55 | -8.48853 | 0.001423 |
| ICMT | 0.949986 | 0.037285 |
| TRIM7 | 1.175653 | 0.009987 |
| TBK1 | -0.5636 | 0.006487 |
| GUCA1B | 0.298737 | 0.047403 |
| C17orf104 | -3.80531 | 0.004236 |
| C17orf100 | -10.8894 | 0.009101 |
| SMPD2 | 4.807463 | 0.000402 |
| AAMP | 0.013589 | 0.006377 |
| FUNDC2P2 | 9.329675 | 0.0001 |
| CARS | 3.8315 | 0.025241 |
| IL6R | -8.96332 | 0.013887 |
| ENO1 | 8.243616 | 0.00256 |
| ACOX2 | 0.794184 | 0.049145 |
| SLC22A13 | 5.787842 | 0.029042 |
| SLC22A11 | -4.47082 | 0.002146 |
| CFD | 0.37331 | 0.018016 |
| PTK7 | -10.4495 | 0.048612 |
| PRKAA2 | -5.46693 | 0.039571 |
| C3orf35 | -2.67067 | 0.040111 |
| IL17REL | -0.21147 | 0.001114 |
| MATN3 | -2.83682 | 0.017554 |
| FFAR2 | -1.1843 | 0.049921 |
| BDH1 | 0.206955 | 0.011082 |
| DDX25 | -2.39556 | 0.036535 |
| UNC13B | 1.630294 | 0.02462 |
| UNC13C | -3.54211 | 0.029165 |
| FAM3C | -4.8017 | 0.01343 |
| FBXO15 | -7.64721 | 0.004555 |
| PROX1 | -9.54774 | 0.004343 |
| CLK2P | 0.241714 | 0.025361 |
| CCDC73 | -6.12391 | 0.001012 |
| TMEM109 | 1.025893 | 0.001114 |
| TMEM105 | -4.30537 | 0.025241 |
| SYT3 | -2.81032 | 0.019306 |
| SARS | 11.1039 | 0.001939 |
| STOML2 | 4.992615 | 0.007574 |
| RPN1 | 4.485122 | 0.03463 |
| AHNAK | 1.403482 | 0.038809 |
| ZNF431 | -1.77461 | 0.025743 |
| MAP1A | -3.95886 | 0.043591 |
| PDCL3 | 8.116481 | 0.020237 |
| CCM2 | 3.015301 | 0.006269 |
| MAP1S | 1.275743 | 0.020705 |
| ZSCAN5A | -9.85953 | 0.009656 |
| FRMD5 | -5.53381 | 0.014801 |
| DSCR3 | -7.747 | 0.001012 |
| ZNF433 | -3.17971 | 0.01208 |
| SIX5 | 2.082094 | 0.019188 |
| SIX2 | 6.290958 | 0.021072 |
| KRT4 | 19.85625 | 0.001732 |
| CEND1 | 3.991976 | 0.048219 |
| VPS41 | -7.37403 | 0.001526 |
| NMNAT2 | -6.74429 | 0.020471 |
| ALOX12B | 3.973457 | 0.007357 |
| NAA16 | -2.72044 | 0.01175 |
| RHOXF1 | 5.477182 | 0.001012 |
| VHL | -4.95847 | 0.039061 |
| PPP1R15A | -4.14597 | 0.011305 |
| MRPL37 | 3.13024 | 0.030787 |
| SPTBN1 | 7.072203 | 0.009656 |
| KRTDAP | 21.0587 | 0.000402 |
| SLC44A2 | 2.599629 | 0.001423 |
| ZBTB7B | 0.515329 | 0.021072 |
| MSI1 | -9.14899 | 0.03413 |
| C4orf12 | -8.35338 | 0.00132 |
| OR2L13 | -4.23816 | 0.01264 |
| NDUFS5 | 4.753052 | 0.03894 |
| C1S | -7.88534 | 0.032027 |
| HBD | 10.5648 | 0.013202 |
| HBB | 6.400906 | 0.014113 |
| ATP9A | 2.940021 | 0.035785 |
| HBZ | 14.33808 | 0.002769 |
| INPP4A | -14.835 | 0.000604 |
| PHC1 | -6.77524 | 0.00091 |
| MFAP3L | -4.27195 | 0.035392 |
| SAFB | 2.011102 | 0.0001 |
| MGMT | 4.133417 | 0.004979 |
| TRNP1 | -9.26438 | 0.037536 |
| RNF6 | -5.16545 | 0.011862 |
| RNF5 | 5.520791 | 0.048219 |
| RNF2 | -2.80967 | 0.001526 |
| APP | -11.8861 | 0.011971 |
| RYBP | -9.53933 | 0.000604 |
| CLTA | 6.036548 | 0.039971 |
| CLTB | 1.161773 | 0.013654 |
| DPEP3 | 2.818678 | 0.006487 |
| USP27X | -2.51705 | 0.029659 |
| EPM2A | 4.48953 | 0.035015 |
| UBE2NL | 1.990151 | 0.005514 |
| MAP7D1 | 3.703572 | 0.018016 |
| KCP | 6.988239 | 0.012527 |
| ACTR2 | -4.58903 | 0.013887 |
| POLDIP3 | 2.910321 | 0.00256 |
| RASA3 | 5.568215 | 0.003186 |
| ZXDB | -5.44117 | 0.003499 |
| NKX3-1 | -5.58175 | 0.002769 |
| RPL12 | 3.533435 | 0.04476 |
| SPRR1B | 29.48535 | 0.000402 |
| ADAM10 | -4.25058 | 0.03413 |
| ADAM12 | 1.161422 | 0.01457 |
| CPNE8 | -10.2415 | 0.019893 |
| TRIT1 | -3.73313 | 0.023313 |
| SFRS12 | -1.61767 | 0.018016 |
| SFRS11 | -0.81318 | 0.030787 |
| FLJ45445 | -4.22007 | 0.022841 |
| RABEPK | 6.171655 | 0.026597 |
| AAK1 | -4.27597 | 0.049921 |
| KLC4 | 0.50477 | 0.029659 |
| RGS9BP | 2.722328 | 0.006161 |
| UCP2 | 1.181734 | 0.029042 |
| ZFR2 | -0.79567 | 0.023313 |
| PLCB1 | -7.19658 | 0.008005 |
| ZDHHC13 | -10.6802 | 0.004023 |
| ZDHHC12 | 4.464844 | 0.042306 |
| HIPK4 | 0.714244 | 0.023077 |
| LEMD2 | 1.855799 | 0.005194 |
| QARS | 9.501298 | 0.001732 |
| SLC16A6 | -6.68179 | 0.00225 |
| C3orf15 | -11.5558 | 0.004872 |
| C3orf14 | -9.80216 | 0.0226 |
| LARP4 | -2.03741 | 0.018948 |
| LARP7 | -1.75301 | 0.027578 |
| TNFSF15 | -5.53557 | 0.014456 |
| GPATCH3 | 4.651935 | 0.002354 |
| HSD17B7P2 | -3.47919 | 0.005408 |
| DDX49 | 5.527315 | 0.001012 |
| ELAVL2 | -5.8478 | 0.004555 |
| DPYSL4 | 1.335859 | 0.032895 |
| UBE2D1 | -8.37933 | 0.001629 |
| EDNRA | -3.87489 | 0.0226 |
| APOB | -3.51809 | 0.013202 |
| FAM151A | 11.16281 | 0.005301 |
| NFIL3 | -6.47596 | 0.023553 |
| VEGFB | 2.212905 | 0.038809 |
| CCDC51 | 4.387539 | 0.005087 |
| CCDC52 | -3.05728 | 0.007247 |
| CCDC59 | -0.52832 | 0.018948 |
| TMEM128 | -0.11395 | 0.038162 |
| NUFIP2 | -1.56444 | 0.019306 |
| RPL13AP6 | 5.572475 | 0.010097 |
| CABP4 | 3.625213 | 0.048219 |
| FBXO34 | -2.56708 | 0.028547 |
| FBXO36 | -2.89775 | 0.006377 |
| FBXO32 | -10.4899 | 0.024264 |
| FBXO39 | -7.16917 | 0.001629 |
| SPAG17 | 12.88027 | 0.001629 |
| PELP1 | 5.829727 | 0.00132 |
| MBOAT2 | -0.65038 | 0.049921 |
| SPSB2 | 1.32821 | 0.006921 |
| HINT2 | 1.33558 | 0.038543 |
| IL23A | -3.57464 | 0.006269 |
| CDC37 | 2.121124 | 0.00132 |
| ALAS2 | 9.020997 | 0.016754 |
| LONRF1 | -8.10828 | 0.032644 |
| CDCA7L | -6.75446 | 0.042431 |
| ZNF365 | -5.64988 | 0.01377 |
| MORC4 | 4.567482 | 0.015833 |
| TXNL4A | 3.051761 | 0.046596 |
| WRB | -2.56509 | 0.025609 |
| CCNA1 | -13.8354 | 0.000808 |
| ATP13A3 | -2.36411 | 0.04556 |
| FLJ26850 | -0.82616 | 0.039971 |
| C1orf110 | 18.02567 | 0.002977 |
| C1orf114 | -0.87909 | 0.003186 |
| ADAT3 | 0.848288 | 0.020122 |
| E2F5 | -7.26753 | 0.019776 |
| E2F1 | 6.908165 | 0.034256 |
| CFLP1 | -4.30734 | 0.018483 |
| ACBD3 | -2.58571 | 0.009544 |
| ACBD7 | -4.59645 | 0.04727 |
| ACBD5 | -2.62355 | 0.045948 |
| EEF2 | 4.515807 | 0.006487 |
| C2orf28 | 2.745595 | 0.042306 |
| RPGR | -3.776 | 0.013202 |
| ZNF334 | -8.28628 | 0.022481 |
| CTBS | -2.26897 | 0.008228 |
| WNT10B | 5.356588 | 0.030164 |
| ZNF331 | -3.58755 | 0.001835 |
| RCN2 | -2.47191 | 0.012755 |
| SEC24A | -7.71437 | 0.02781 |
| SEC24C | 4.662369 | 0.008775 |
| RNF19B | -0.67106 | 0.025867 |
| ACCN2 | 1.083888 | 0.041653 |
| MAGT1 | -5.38693 | 0.02635 |
| SMC5 | -2.95286 | 0.033753 |
| FHAD1 | -6.71793 | 0.001835 |
| ARFGAP2 | 1.921284 | 0.019541 |
| ARFGAP3 | -2.27756 | 0.012755 |
| C16orf3 | -2.12355 | 0.025743 |
| LAMB2 | 0.940132 | 0.000201 |
| COPZ1 | 8.687309 | 0.021431 |
| NEK4 | -4.70152 | 0.010752 |
| C21orf2 | 1.0515 | 0.037159 |
| G0S2 | -6.14212 | 0.037911 |
| RSPH9 | -8.55969 | 0.008337 |
| CCDC6 | -3.09437 | 0.047403 |
| CCDC9 | 0.022693 | 0.00132 |
| GSS | 4.845691 | 0.026848 |
| TMEM189 | 3.204494 | 0.039571 |
| RHD | 0.714332 | 0.033383 |
| ADSS | -2.78238 | 0.007466 |
| R3HCC1 | 1.959337 | 0.00132 |
| GAS2L1 | 1.385582 | 0.011639 |
| NDFIP1 | -1.0819 | 0.010206 |
| SYTL5 | -11.6464 | 0.002665 |
| ZNF341 | 1.799753 | 0.026848 |
| TRIML1 | 15.18357 | 0.014682 |
| NUDT12 | -1.84048 | 0.031659 |
| NUDT14 | 1.345838 | 0.021072 |
| STX8 | 3.694199 | 0.017094 |
| PHACTR4 | -5.33119 | 0.016636 |
| LOC388242 | 2.956346 | 0.033627 |
| LOC90246 | -8.03471 | 0.015606 |
| DRG2 | 3.315776 | 0.036409 |
| TMPRSS11A | 11.71325 | 0.028181 |
| LOC348926 | -7.0315 | 0.044117 |
| AP1B1 | 2.864743 | 0.009212 |
| TXLNB | -4.40354 | 0.002665 |
| AHCY | 4.172942 | 0.049534 |
| ABI2 | -12.1032 | 0.001217 |
| SLC22A4 | -8.17484 | 0.004343 |
| DHDDS | 7.60351 | 0.024864 |
| CHN2 | -0.47998 | 0.027932 |
| LRRC39 | -4.77069 | 0.009212 |
| JKAMP | -2.67561 | 0.00413 |
| PTPLAD1 | -0.24171 | 0.043857 |
| XRCC3 | 1.022198 | 0.009101 |
| ZNF669 | -5.43811 | 0.001939 |
| RALGPS2 | -5.44502 | 0.016754 |
| MIER1 | -3.93583 | 0.02474 |
| MIER3 | -2.72398 | 0.023911 |
| DDX60 | -0.30198 | 0.028426 |
| AMN1 | -0.78975 | 0.002665 |
| IL13RA1 | -3.71896 | 0.010752 |
| LOC100144603 | 5.250298 | 0.006921 |
| CTSL2 | -10.4866 | 0.010861 |
| INSIG2 | -5.36752 | 0.0001 |
| SMN1 | -6.51151 | 0.001114 |
| DGCR2 | 1.064223 | 0.011418 |
| PPCS | 2.682442 | 0.049656 |
| H2AFY2 | -9.70034 | 0.031659 |
| NAMPT | -3.89183 | 0.002872 |
| KCNA5 | 3.46814 | 0.035392 |
| KCNA2 | 9.56325 | 0.001526 |
| OPTN | 0.89936 | 0.033753 |
| JMJD5 | 5.666957 | 0.000808 |
| ZHX2 | 5.983941 | 0.005087 |
| ZHX1 | -4.82514 | 0.026725 |
| PLA2G2A | -2.42991 | 0.012414 |
| SLC26A10 | -4.07956 | 0.025241 |
| SARS2 | 4.637234 | 0.001012 |
| HS3ST6 | 13.83239 | 0.012755 |
| CYFIP1 | 2.048278 | 0.001732 |
| LOC151658 | 26.9955 | 0.002146 |
| TRIM24 | -0.46714 | 0.043341 |
| C11orf90 | 3.337842 | 0.042949 |
| MED22 | 4.365735 | 0.000201 |
| PXDNL | 9.14246 | 0.029659 |
| LHPP | 0.258592 | 0.009656 |
| OR2C1 | 1.3743 | 0.002042 |
| DMXL2 | -5.381 | 0.039703 |
| GTPBP8 | -2.0416 | 0.018714 |
| BPNT1 | -1.37936 | 0.01457 |
| PCDHA7 | -1.44272 | 0.020951 |
| BCL9 | -1.22159 | 0.006377 |
| BCL3 | -0.06993 | 0.019071 |
| ZNF282 | 2.399192 | 0.007682 |
| CDX2 | 14.66134 | 0.02635 |
| ZNF665 | -6.04591 | 0.010422 |
| ZP1 | 1.417229 | 0.034381 |
| KLHL22 | 1.014574 | 0.015258 |
| NEK9 | 3.365581 | 0.044891 |
| CSNK1G3 | -4.09831 | 0.003918 |
| SERPINB3 | 12.70724 | 0.01377 |
| FAM76B | -2.87848 | 0.046212 |
| C18orf10 | 7.54608 | 0.02415 |
| EXD2 | 0.603132 | 0.030532 |
| KLHDC3 | 6.747009 | 0.002354 |
| KLHDC1 | -7.23808 | 0.045685 |
| HTR2C | 12.01335 | 0.032767 |
| HTR2B | -4.4084 | 0.013088 |
| C8orf22 | 16.21632 | 0.021431 |
| KLHL30 | 1.341556 | 0.030665 |
| KIAA0895 | -16.0653 | 0.001114 |
| SS18 | -4.17471 | 0.002977 |
| MRPS36 | -1.28511 | 0.022127 |
| DIO3OS | -2.99893 | 0.028181 |
| GPR143 | -11.8513 | 0.008005 |
| SLC7A9 | -0.69434 | 0.027578 |
| PFDN5 | 8.562399 | 0.016175 |
| PFDN6 | 3.815048 | 0.005087 |
| MAGEA11 | 12.66467 | 0.020951 |
| NFKBID | -3.50862 | 0.026476 |
| TAB1 | 0.003668 | 0.012527 |
| WWOX | 1.453227 | 0.002665 |
| RNH1 | 0.481815 | 0.037159 |
| RPS15 | 5.50203 | 0.045431 |
| RPS18 | 2.96985 | 0.037159 |
| LAMA2 | -7.57374 | 0.014113 |
| LOC100128164 | 5.215109 | 0.015946 |
| SLC16A4 | -10.4311 | 0.020588 |
| SPRR1A | 23.08622 | 0.001835 |
| TUBG1 | 5.686912 | 0.017212 |
| PLCXD3 | -6.97544 | 0.022241 |
| HAUS8 | 3.19343 | 0.044504 |
| ACTN4 | 2.303887 | 0.015718 |
| HAUS7 | 1.390436 | 0.046731 |
| PTPRM | -4.89855 | 0.026974 |
| MRPL41 | 0.603094 | 0.047 |
| BANK1 | 0.146758 | 0.042692 |
| DOC2A | -5.23429 | 0.040493 |
| RRP7A | 0.434332 | 0.01343 |
| MAMSTR | -0.28012 | 0.020471 |
| GPR156 | -4.18836 | 0.041524 |
| GPR155 | -6.09301 | 0.006377 |
| F8 | -4.71066 | 0.006813 |
| HMGN2 | 3.462238 | 0.049534 |
| HMGN1 | -7.86647 | 0.01343 |
| GSTZ1 | 6.031687 | 0.000706 |
| DULLARD | 3.81482 | 0.012755 |
| ARL5A | -4.99275 | 0.007357 |
| CBY1 | 7.407601 | 0.001835 |
| ARL5B | -7.45017 | 0.003918 |
| TM9SF1 | 4.597534 | 0.048219 |
| KRT13 | 19.22615 | 0.001939 |
| EIF4A2 | -1.7623 | 0.01503 |
| SPTA1 | 8.155277 | 0.020471 |
| SLC26A8 | -3.36005 | 0.048882 |
| TM9SF3 | -2.49952 | 0.006921 |
| PCDHB15 | -9.45725 | 0.004766 |
| HS2ST1 | -1.61995 | 0.015718 |
| C10orf11 | 0.680645 | 0.03413 |
| N4BP2 | -6.79287 | 0.037285 |
| NR4A1 | -8.61371 | 0.006487 |
| PTPRS | 4.159343 | 0.028676 |
| SDC3 | 0.869241 | 0.042172 |
| COX5B | 5.26705 | 0.019071 |
| TH1L | 6.247473 | 0.007028 |
| LOC401588 | -6.64193 | 0.0001 |
| NMT1 | 1.220435 | 0.044253 |
| IFT81 | -6.32484 | 0.016175 |
| ACAA1 | 0.480198 | 0.014227 |
| ARIH1 | -2.29638 | 0.046874 |
| AGPAT3 | 9.348259 | 0.000808 |
| SUFU | 3.637083 | 0.024994 |
| MBD4 | -0.91791 | 0.024383 |
| CMTM8 | -4.64346 | 0.024994 |
| MBD3 | 1.641922 | 0.036283 |
| 1-Dec | 15.39515 | 0.039304 |
| TSPAN10 | 1.401124 | 0.009766 |
| TSPAN12 | -2.64999 | 0.033259 |
| TSPAN16 | 16.4383 | 0.030037 |
| SFTPA2 | 5.607313 | 0.035142 |
| REXO4 | 5.385862 | 0.000503 |
| SLC25A14 | -6.77203 | 0.001012 |
| SLC25A12 | -4.23553 | 0.039703 |
| RNF216 | 0.746413 | 0.031529 |
| ZNF321 | -3.14064 | 0.020353 |
| EAF1 | -1.98517 | 0.011082 |
| CCDC11 | -5.24466 | 0.029284 |
| CCDC12 | 1.487681 | 0.035785 |
| ZNF280C | -5.24659 | 0.017212 |
| GNG4 | -2.03206 | 0.009656 |
| DBF4 | -2.5109 | 0.022841 |
| ALG8 | 8.437266 | 0.011529 |
| ALG1 | 7.611439 | 0.042692 |
| COX6B1 | 5.07426 | 0.035515 |
| STAM | -1.96876 | 0.016754 |
| KIAA1683 | -5.83502 | 0.018136 |
| NOSIP | 6.094368 | 0.009766 |
| GCC2 | -5.29546 | 0.001939 |
| CLDN1 | -11.3145 | 0.002042 |
| RASGEF1A | -11.8953 | 0.018829 |
| MAGEB4 | -1.29568 | 0.013088 |
| KIAA0114 | -9.80941 | 0.004236 |
| IRF1 | -1.14836 | 0.030665 |
| C6orf47 | 1.835378 | 0.001114 |
| SNX25 | -5.53111 | 0.000604 |
| GPER | 1.783796 | 0.011639 |
| ONECUT2 | -1.45098 | 0.003186 |
| METT11D1 | -6.53364 | 0.023911 |
| PARP4 | -3.5294 | 0.025484 |
| MRPL20 | 5.279216 | 0.025867 |
| U2AF1L4 | -2.53292 | 0.018483 |
| PTPRE | -9.87225 | 0.004661 |
| ZBTB10 | -9.19399 | 0.002146 |
| RCOR1 | -2.13634 | 0.045144 |
| C1orf151 | 4.360784 | 0.019188 |
| KLHL14 | -4.83245 | 0.041385 |
| KLHL15 | -3.80085 | 0.006487 |
| TRIM62 | -0.69911 | 0.032516 |
| TRIM66 | -0.79743 | 0.038284 |
| C2orf63 | -7.52251 | 0.002146 |
| C2orf60 | -0.92251 | 0.027096 |
| C2orf65 | 5.158277 | 0.036663 |
| ZNF853 | -6.39026 | 0.047403 |
| C2orf69 | -4.8988 | 0.001629 |
| IER2 | -0.80487 | 0.021431 |
| PRCD | -7.19051 | 0.015258 |
| RRN3P1 | -8.50477 | 0.004555 |
| SND1 | 5.9388 | 0.007791 |
| PAQR5 | -3.6056 | 0.015606 |
| PYDC1 | 8.785091 | 0.044376 |
| CYBA | 2.511049 | 0.02462 |
| HMHA1 | 2.618128 | 0.010971 |
| RAB15 | -14.0246 | 0.022361 |
| PHGR1 | 4.754875 | 0.037911 |
| EGR3 | -8.32923 | 0.002042 |
| TCTEX1D4 | -3.11752 | 0.000402 |
| HSF2 | -1.89548 | 0.008228 |
| N4BP2L2 | -0.99889 | 0.020588 |
| C7orf53 | -7.34599 | 0.029783 |
| C7orf58 | -4.20475 | 0.004661 |
| PRHOXNB | 20.82626 | 0.006703 |
| VPS39 | 3.906189 | 0.042043 |
| PARK7 | 6.557267 | 0.004872 |
| RORB | -0.5645 | 0.026848 |
| RORA | -9.16404 | 0.00329 |
| MDM2 | -0.02231 | 0.00132 |
| ROR2 | -12.63 | 0.028676 |
| LOC646982 | -5.85468 | 0.013202 |
| KIN | -1.39784 | 0.028304 |
| SPATA18 | -8.34923 | 0.037662 |
| FRS2 | -0.59137 | 0.019424 |
| GOLT1B | -2.93304 | 0.019893 |
| CRK | 1.663499 | 0.029911 |
| SLC25A15 | -3.99913 | 0.008447 |
| GLDC | -0.73985 | 0.004448 |
| B4GALT2 | 2.941538 | 0.03227 |
| B4GALT7 | 3.621324 | 0.006269 |
| FAM174A | -1.31762 | 0.030284 |
| TBC1D15 | -0.27463 | 0.015718 |
| EHMT1 | 1.978707 | 0.025609 |
| EHMT2 | 3.455397 | 0.005944 |
| ADIPOR2 | 7.556404 | 0.026228 |
| WDR83 | 0.126787 | 0.007682 |
| CD63 | 1.157079 | 0.039061 |
| TPD52L2 | 5.372505 | 0.027096 |
| HSPA1B | 11.50397 | 0.005621 |
| WDR12 | -0.74353 | 0.041653 |
| WDR17 | -11.0335 | 0.001526 |
| AOX1 | -5.59964 | 0.005944 |
| C1orf212 | 2.927907 | 0.018016 |
| C1orf216 | 1.645966 | 0.013314 |
| SLC16A14 | -5.20595 | 0.019424 |
| WASL | -6.27106 | 0.003604 |
| ZMYND19 | 2.966416 | 0.00132 |
| PHGDH | 4.628842 | 0.00132 |
| HSPB6 | -6.59027 | 0.0226 |
| PKN3 | 0.304854 | 0.01343 |
| TPH1 | -1.03604 | 0.001012 |
| IDH3B | 3.36425 | 0.006269 |
| RHOBTB1 | -4.38154 | 0.002977 |
| FAM43B | 6.524595 | 0.001114 |
| ZNF784 | -4.03262 | 0.027209 |
| TEX261 | 3.879832 | 0.046731 |
| LMF2 | 1.159512 | 0.029284 |
| CMPK2 | -2.3861 | 0.043463 |
| LIG4 | -6.0778 | 0.001629 |
| CSNK1E | -6.78008 | 0.034761 |
| BDP1 | -6.68274 | 0.028796 |
| ALS2CR8 | -3.4614 | 0.016405 |
| FNTB | 13.11593 | 0.001012 |
| DPY19L2P4 | -0.71108 | 0.005301 |
| ZKSCAN4 | 0.507541 | 0.009101 |
| LOC100240726 | -3.70214 | 0.032148 |
| FNBP4 | -2.30857 | 0.004343 |
| SLC25A37 | -7.52006 | 0.001526 |
| TMEM106B | -6.19558 | 0.026725 |
| FAM23A | -5.19563 | 0.021072 |
| GNG2 | 1.396304 | 0.044117 |
| RIBC1 | 2.838106 | 0.028426 |
| TMEM188 | -3.54375 | 0.022958 |
| PRH1 | -6.42034 | 0.005194 |
| ZMAT2 | 5.497372 | 0.022481 |
| TAF6 | 0.982758 | 0.043984 |
| TSPAN32 | -1.19956 | 0.021072 |
| TSPAN33 | 1.058662 | 0.029659 |
| CCNG1 | -4.34833 | 0.014456 |
| LY6G6F | 14.7554 | 0.043084 |
| LRRCC1 | -3.68427 | 0.001114 |
| PDCD4 | -1.66536 | 0.048882 |
| LOC285033 | 3.088206 | 0.001217 |
| HDLBP | 6.717176 | 0.0001 |
| PRKCE | -9.64927 | 0.008883 |
| C6orf26 | -6.0476 | 0.009101 |
| VPS26A | -0.65873 | 0.002977 |
| VPS26B | 0.178362 | 0.00256 |
| PRKCD | 11.54748 | 0.001526 |
| SLC48A1 | 2.07328 | 0.021192 |
| SSRP1 | 5.105892 | 0.001217 |
| LOC100128292 | -7.20776 | 0.001732 |
| IL12A | -1.87758 | 0.017554 |
| TAF6L | 0.418114 | 0.004661 |
| SNUPN | 3.078814 | 0.012974 |
| C1orf175 | -11.2155 | 0.003186 |
| C1orf177 | -3.81791 | 0.003186 |
| MTA2 | 3.844344 | 0.003499 |
| LRP11 | -3.10366 | 0.02415 |
| GPC6 | -4.44884 | 0.010752 |
| SEC22B | 10.14992 | 0.019893 |
| BBS2 | -5.8053 | 0.014801 |
| ELANE | 3.049115 | 0.028181 |
| PDCD11 | 1.767929 | 0.02781 |
| GPR182 | -7.75837 | 0.001939 |
| ZSWIM4 | -7.91791 | 0.025361 |
| LOC91450 | 5.255443 | 0.044376 |
| SCLY | 6.668365 | 0.025743 |
| RER1 | 4.065984 | 0.03463 |
| SCG5 | -7.41647 | 0.038543 |
| MGC15885 | 7.61091 | 0.026725 |
| FAM84A | -7.27251 | 0.031786 |
| RPL18 | 3.18796 | 0.034381 |
| ZNF674 | -2.00661 | 0.026476 |
| ZNF673 | -3.53834 | 0.000706 |
| MAF | -7.79306 | 0.011529 |
| AG2 | -0.08423 | 0.011196 |
| ALDH3A1 | 5.356921 | 0.003604 |
| EDC3 | 1.067771 | 0.03413 |
| MGST1 | 3.264383 | 0.048086 |
| C9orf130 | -5.80662 | 0.045294 |
| BACH1 | -9.41446 | 0.000808 |
| GMDS | 4.517407 | 0.00132 |
| LOC220594 | -3.18639 | 0.016175 |
| OTUD7B | 1.93261 | 0.041137 |
| ANAPC2 | 0.346 | 0.024864 |
| RPS6KA3 | -10.2311 | 0.01457 |
| RPS6KA6 | -9.62357 | 0.019424 |
| PISD | 4.836916 | 0.037786 |
| PSMD13 | 6.292693 | 0.046074 |
| HBQ1 | 21.55448 | 0.000503 |
| GANAB | 3.507067 | 0.004023 |
| VAPA | -0.1985 | 0.01629 |
| MKI67IP | -1.72203 | 0.040111 |
| NPHP1 | -5.83663 | 0.022361 |
| CTH | -0.51735 | 0.006703 |
| ARF5 | 4.857703 | 0.047671 |
| C6orf182 | -4.15043 | 0.025241 |
| MYLK3 | -4.24637 | 0.025867 |
| RGPD1 | -5.29222 | 0.006051 |
| MOB2 | 2.756017 | 0.026725 |
| RNASET2 | 1.466873 | 0.01377 |
| RAB2B | -10.2733 | 0.000301 |
| TSPYL5 | -5.22387 | 0.01629 |
| RPL7A | 5.348567 | 0.003918 |
| AASS | -10.0483 | 0.020353 |
| PEX14 | 2.652876 | 0.006703 |
| ETNK1 | -7.41983 | 0.000706 |
| DHRS7B | 0.965959 | 0.00573 |
| CA14 | -3.64434 | 0.004448 |
| CA13 | -12.0797 | 0.004872 |
| CA11 | -7.90265 | 0.025743 |
| WDR78 | -5.23468 | 0.020353 |
| ACTR1A | 3.573787 | 0.004343 |
| WDR73 | -4.57519 | 0.01629 |
| WDR74 | 6.016856 | 0.000706 |
| TRHDE | -2.9206 | 0.028547 |
| SFMBT2 | -8.97717 | 0.012755 |
| C5orf44 | -3.30358 | 0.012755 |
| C5orf45 | -4.67659 | 0.006813 |
| C5orf43 | -1.80721 | 0.006051 |
| NCRNA00176 | -0.04428 | 0.033259 |
| ARRDC2 | 6.744766 | 0.004236 |
| ELK3 | -5.71519 | 0.041787 |
| PAK3 | -8.40558 | 0.035785 |
| FAM122B | -4.39937 | 0.0001 |
| FAM122C | -1.6925 | 0.002146 |
| SH3BP2 | -2.23935 | 0.037285 |
| KCNG1 | -9.70553 | 0.033503 |
| MFSD2A | -5.17444 | 0.002665 |
| ZIC1 | 2.400945 | 0.009987 |
| SPRR3 | 23.11159 | 0.002457 |
| HIF1A | -2.80297 | 0.025241 |
| ARGLU1 | -2.7401 | 0.027455 |
| CALML5 | 1.253376 | 0.001629 |
| PFN2 | -3.94189 | 0.036913 |
| SHISA2 | -3.44723 | 0.030164 |
| COX11 | -3.57573 | 0.001939 |
| GAS2 | -6.87745 | 0.001629 |
| ARPP21 | 14.58512 | 0.001526 |
| ANKRD13A | -3.58705 | 0.004555 |
| KIAA1467 | -2.94525 | 0.005944 |
| ALCAM | -9.95841 | 0.002872 |
| C22orf39 | 4.312722 | 0.032644 |
| CYB5D2 | 1.26495 | 0.0001 |
| C22orf31 | 2.16588 | 0.045814 |
| TAX1BP1 | -2.04425 | 0.000706 |
| SMNDC1 | -4.47615 | 0.00329 |
| SSX2IP | -9.80314 | 0.008775 |
| ZNF267 | -2.14972 | 0.006813 |
| PPAN | 1.487644 | 0.00132 |
| PPP1CA | 11.55115 | 0.01377 |
| MPPED2 | -0.90874 | 0.017212 |
| LYSMD3 | -6.67108 | 0.01503 |
| CLGN | -3.14076 | 0.005087 |
| SELENBP1 | 6.287338 | 0.001629 |
| BAZ2B | -5.04026 | 0.029532 |
| GOLGA6L9 | -7.90059 | 0.001114 |
| FYN | 0.696963 | 0.02733 |
| PAF1 | 5.865537 | 0.000808 |
| XAGE1D | 15.79473 | 0.007791 |
| RUFY1 | 6.705921 | 0.001217 |
| RUFY3 | -0.13287 | 0.026848 |
| RUFY2 | -5.48626 | 0.007138 |
| NLE1 | 7.422303 | 0.001526 |
| ROD1 | -3.84089 | 0.021778 |
| KRT78 | 15.10019 | 0.029783 |
| SPAST | -7.2973 | 0.001217 |
| MUM1L1 | -3.18171 | 0.000706 |
| RNF34 | 2.416979 | 0.036913 |
| WIBG | 7.211494 | 0.013887 |
| WBP5 | -6.94004 | 0.014913 |
| TM2D3 | -2.25719 | 0.004555 |
| DCK | -2.61581 | 0.047403 |
| ACSM3 | -2.98911 | 0.030037 |
| ACSM5 | -2.20533 | 0.041006 |
| SDF4 | 0.093991 | 0.038543 |
| CREB3 | 6.313521 | 0.010097 |
| PER2 | -0.92349 | 0.038809 |
| FLRT3 | -8.72693 | 0.02415 |
| RHOT1 | -1.34818 | 0.012304 |
| KAT5 | 3.623209 | 0.012755 |
| F2R | -5.80105 | 0.013887 |
| AES | 3.479358 | 0.001423 |
| DNAJC27 | -6.5864 | 0.002977 |
| DNAJC24 | -0.89731 | 0.043084 |
| ARHGAP8 | -11.011 | 0.049145 |
| AEN | -4.64623 | 0.040623 |
| TSPYL2 | -4.16813 | 0.013202 |
| PRLR | -13.1305 | 0.011082 |
| ZFP36 | -8.23626 | 0.005514 |
| ANXA11 | 5.530324 | 0.001217 |
| ANXA13 | -14.8664 | 0.001423 |
| KCNQ4 | 3.985026 | 0.00132 |
| FAM86A | 6.251567 | 0.035265 |
| MGC12982 | 0.738503 | 0.02462 |
| MRPL2 | 2.012465 | 0.028922 |
| RPUSD2 | 5.986387 | 0.001114 |
| ZFAT | 3.948334 | 0.001939 |
| ZNF611 | -3.30218 | 0.046731 |
| WBSCR16 | 2.381377 | 0.00225 |
| RUVBL1 | 3.870595 | 0.006813 |
| ZNF614 | -1.9277 | 0.034505 |
| EDDM3A | 14.15946 | 0.002354 |
| EDDM3B | 7.835246 | 0.019893 |
| CTDP1 | 1.25712 | 0.001217 |
| LRRC37A3 | -5.33621 | 0.009323 |
| SLC39A7 | 1.201435 | 0.002977 |
| LCAT | -6.04895 | 0.01767 |
| EVI5 | -8.43188 | 0.001114 |
| FSCN3 | -5.45342 | 0.00225 |
| G2E3 | -4.44557 | 0.039703 |
| RILPL2 | 0.868666 | 0.005621 |
| TMPO | -2.7454 | 0.015374 |
| LDHAL6A | -8.71057 | 0.003604 |
| HHAT | -7.60953 | 0.007247 |
| FMR1 | -10.4354 | 0.000201 |
| LYPD3 | -11.0192 | 0.047815 |
| RGS1 | -4.95034 | 0.048751 |
| RGS2 | -4.71242 | 0.044634 |
| LOC100133669 | -14.2173 | 0.004236 |
| RGS9 | -8.84262 | 0.029407 |
| TRDN | 3.054048 | 0.034256 |
| SERTAD2 | -1.45612 | 0.030284 |
| IL1RAP | -6.22872 | 0.013542 |
| ARAP1 | 0.513238 | 0.016754 |
| XIAP | -4.06922 | 0.012974 |
| EPDR1 | 2.754302 | 0.034256 |
| DOLK | 0.791891 | 0.040752 |
| NALCN | -12.1581 | 0.011418 |
| PTP4A1 | -9.40862 | 0.0001 |
| REC8 | -0.4162 | 0.012191 |
| GPR3 | -4.59994 | 0.01767 |
| SUZ12 | -5.01712 | 0.005621 |
| MRPS9 | 5.510046 | 0.007574 |
| C9orf24 | 6.885115 | 0.019424 |
| OTUD1 | -1.09495 | 0.045294 |
| TWF1 | -1.51554 | 0.009433 |
| MBTPS2 | -7.27014 | 0.021431 |
| WDR43 | -1.67509 | 0.028922 |
| WDR59 | -6.20607 | 0.034381 |
| DR1 | -6.80329 | 0.014801 |
| HOXC9 | 2.625892 | 0.033753 |
| PPP2CB | -1.76919 | 0.042949 |
| HOXC5 | 4.388043 | 0.020471 |
| CD151 | 3.550596 | 0.005944 |
| SSU72 | 1.418371 | 0.019541 |
| SLC25A32 | -3.32059 | 0.004343 |
| LSM4 | 8.548425 | 0.032644 |
| COL27A1 | -5.52078 | 0.036913 |
| PTPN23 | 2.069578 | 0.01825 |
| POLR2D | -1.81867 | 0.000201 |
| POLR2L | 1.234571 | 0.022127 |
| AKR7A2 | 6.935438 | 0.006813 |
| TLK1 | -8.74885 | 0.000301 |
| ASCC2 | 2.112048 | 0.021661 |
| PEA15 | 7.273061 | 0.00573 |
| FZD2 | 1.001962 | 0.033627 |
| FZD3 | -15.8201 | 0.003709 |
| CACNB1 | -3.62915 | 0.047671 |
| C10orf79 | -8.8259 | 0.028426 |
| B3GALNT2 | -2.57409 | 0.009323 |
| B3GALNT1 | -4.33427 | 0.013088 |
| TACC2 | 5.004224 | 0.001217 |
| NHP2 | 4.856886 | 0.031659 |
| MRTO4 | 3.095422 | 0.003709 |
| ESCO1 | -2.9757 | 0.018366 |
| CTU2 | 0.160989 | 0.023193 |
| C22orf13 | 3.968639 | 0.004979 |
| MSX1 | -3.34541 | 0.041524 |
| HSD11B1 | -1.11415 | 0.040878 |
| RPL3 | 1.955468 | 0.020353 |
| GTF2H3 | -8.09714 | 0.02415 |
| GTF2H4 | 9.352075 | 0.000301 |
| MAPKAP1 | 8.79262 | 0.007357 |
| RPL36 | 4.146661 | 0.022361 |
| SRCRB4D | 3.722301 | 0.03894 |
| ATF1 | -4.08873 | 0.008665 |
| ATF3 | -12.3504 | 0.004555 |
| PSPC1 | -6.29378 | 0.019071 |
| SFRS13A | -1.7985 | 0.025743 |
| SFRS13B | -4.75476 | 0.016405 |
| B3GALT6 | 4.508559 | 0.001732 |
| B3GALT2 | -1.10656 | 0.030408 |
| LOC100129534 | 0.458155 | 0.033627 |
| HCN1 | -0.9933 | 0.041524 |
| HCN4 | -0.15004 | 0.046596 |
| SNRNP25 | 6.093936 | 0.02474 |
| LOC390595 | -5.56079 | 0.033503 |
| CHIC2 | -7.1953 | 0.000604 |
| CHIC1 | -9.84927 | 0.000503 |
| PEPD | 1.004758 | 0.003918 |
| FKBP15 | 2.162434 | 0.022127 |
| TBC1D2B | -2.35868 | 0.032027 |
| SENP7 | -9.62536 | 0.001012 |
| SAMM50 | 1.937195 | 0.026228 |
| C21orf59 | 3.904479 | 0.005194 |
| ING3 | -4.78007 | 0.032644 |
| POC1B | -0.83044 | 0.043341 |
| DAG1 | 2.952875 | 0.029165 |
| ACP2 | 3.60471 | 0.023553 |
| SLC2A14 | -2.91634 | 0.006487 |
| SLC2A13 | -7.59798 | 0.009544 |
| ANKRD37 | -7.01566 | 0.000808 |
| GLS | -8.75074 | 0.01767 |
| BSN | -2.92804 | 0.038162 |
| LOC442459 | 4.056864 | 0.048352 |
| MECR | 5.655423 | 0.006921 |
| YTHDC2 | -2.22542 | 0.039571 |
| IL1RN | -0.88166 | 0.020951 |
| SLC27A3 | 1.382215 | 0.002977 |
| ERO1L | -0.62386 | 0.015374 |
| AK7 | -8.07524 | 0.031529 |
| ACTBL2 | -3.88967 | 0.005408 |
| MAP4 | 10.06521 | 0.008447 |
| RRP8 | 3.126162 | 0.026228 |
| MPI | 6.981071 | 0.003604 |
| TMEM55A | -5.28879 | 0.005087 |
| RRP9 | 1.683917 | 0.037911 |
| TRA2A | -2.76502 | 0.021661 |
| KIAA0141 | -2.13398 | 0.025609 |
| FBN1 | 2.333858 | 0.024864 |
| PACRG | -13.2003 | 0.007138 |
| LACTB2 | -0.53554 | 0.01208 |
| RNF121 | 7.252282 | 0.0001 |
| PDE1A | -7.23574 | 0.037035 |
| EEPD1 | 2.657089 | 0.001835 |
| SFTPD | 8.860358 | 0.0001 |
| SFTPB | 18.01122 | 0.002769 |
| KDM1A | -4.47934 | 0.048352 |
| IMPDH1 | 3.54734 | 0.006377 |
| NECAP1 | -1.06252 | 0.034381 |
| MAP9 | -6.19347 | 0.003395 |
| UBXN10 | -8.89384 | 0.010971 |
| MTMR7 | -1.12746 | 0.00132 |
| MTMR6 | -6.71115 | 0.015946 |
| YIPF2 | 2.953602 | 0.048751 |
| TSC22D3 | 1.008495 | 0.030532 |
| FCHSD2 | -5.26282 | 0.012191 |
| MTMR9L | -2.23142 | 0.049009 |
| SV2A | -8.83209 | 0.036535 |
| KIAA1161 | 4.144283 | 0.007247 |
| FOLH1 | -7.35987 | 0.047942 |
| MESDC2 | 9.015644 | 0.009433 |
| EXOSC9 | -1.71637 | 0.008447 |
| ADAMTSL4 | -3.92928 | 0.019306 |
| ADAMTSL5 | 3.497657 | 0.039186 |
| SURF1 | 3.345036 | 0.018136 |
| UPF1 | 0.610811 | 0.000808 |
| DEPDC6 | -5.35273 | 0.037662 |
| EEF1E1 | -8.05203 | 0.005301 |
| LOC388428 | 15.96937 | 0.005944 |
| STXBP3 | -0.25692 | 0.001012 |
| S1PR3 | -1.18799 | 0.023553 |
| NOC2L | 1.901081 | 0.019893 |
| FANCG | 1.012497 | 0.015606 |
| ARSD | -7.94832 | 0.036663 |
| ARSE | -7.74967 | 0.030665 |
| HNRNPA1 | -3.36381 | 0.045685 |
| DUS2L | 5.11878 | 0.014456 |
| EXOC5 | -5.13143 | 0.00573 |
| SLC45A3 | -7.24012 | 0.030532 |
| IRS1 | -5.82397 | 0.040493 |
| CDK2 | -5.94346 | 0.019424 |
| CEP57 | -2.98711 | 0.048882 |
| LIMS3 | -6.33051 | 0.019893 |
| LIMS1 | -5.68258 | 0.015374 |
| ARSJ | -12.8792 | 0.012755 |
| ZFHX3 | 3.923302 | 0.025484 |
| AMD1 | -3.68846 | 0.006813 |
| ATP10A | 0.996319 | 0.009987 |
| BZW1 | -0.77719 | 0.023433 |
| TSGA10IP | 8.007658 | 0.01264 |
| LOC154761 | -3.75641 | 0.020471 |
| TYMP | 0.747705 | 0.044891 |
| MGC16703 | -0.97298 | 0.041137 |
| PPIL2 | 2.793371 | 0.046874 |
| PPIL6 | -5.38517 | 0.000402 |
| MYADM | -0.94763 | 0.014913 |
| CCDC99 | -1.77116 | 0.013314 |
| CCDC91 | -6.66175 | 0.021431 |
| CCDC92 | 4.323576 | 0.017094 |
| CTSZ | 4.304373 | 0.007028 |
| RPL17 | -2.35881 | 0.041006 |
| RPL10 | 2.691943 | 0.048086 |
| RPL11 | 6.380352 | 0.014801 |
| RPL13 | 2.792291 | 0.042692 |
| FGFR4 | -5.84812 | 0.007791 |
| CXCL1 | -5.4085 | 0.032895 |
| CXCL2 | -10.9364 | 0.010206 |
| CXCL5 | -3.15524 | 0.039571 |
| CXCL6 | -2.8625 | 0.019424 |
| METT10D | -2.86572 | 0.017441 |
| CSRP2BP | 2.299255 | 0.042949 |
| AP2S1 | 4.369164 | 0.040752 |
| AFF4 | -4.20919 | 0.047132 |
| ALDH16A1 | 3.236774 | 0.00091 |
| SAE1 | 7.018975 | 0.007574 |
| ERP27 | -6.35169 | 0.020588 |
| ERP29 | 6.686083 | 0.000402 |
| PHKA1 | -5.64061 | 0.004661 |
| UBA6 | -2.97445 | 0.019306 |
| PWP1 | 3.914161 | 0.047671 |
| PHF23 | 2.391577 | 0.009987 |
| UBE2W | -1.9634 | 0.002354 |
| TAC1 | -3.99037 | 0.026725 |
| SCG2 | -0.64949 | 0.01629 |
| ILK | 2.437706 | 0.027932 |
| ADAMTS8 | 1.5297 | 0.031659 |
| TIMM44 | 0.708225 | 0.005194 |
| PDE3A | -2.24293 | 0.040232 |
| C20orf134 | 0.796189 | 0.004448 |
| NDUFA5 | -2.16671 | 0.016636 |
| MAP1LC3B2 | -1.43193 | 0.034005 |
| NDUFA8 | 5.202536 | 0.033383 |
| IMPG2 | -6.28058 | 0.020237 |
| KIAA1967 | 0.523562 | 0.013202 |
| SVIP | -10.5033 | 0.005087 |
| NBR1 | -5.53667 | 0.008992 |
| IL6 | -6.22118 | 0.006161 |
| GNS | 5.629137 | 0.047942 |
| DTX2 | 4.470259 | 0.014227 |
| DTX3 | -10.9325 | 0.027096 |
| OXR1 | -5.1756 | 0.03894 |
| ANKRD19 | 1.966954 | 0.047 |
| RB1CC1 | -6.62824 | 0.007028 |
| POLR2G | 6.601657 | 0.030164 |
| TMEM33 | -2.02247 | 0.03591 |
| MEA1 | 8.072109 | 0.004872 |
| OTOP3 | 20.6599 | 0.002665 |
| TTLL1 | 7.650711 | 0.00132 |
| REP15 | -9.7992 | 0.001012 |
| POLR2J4 | -0.90644 | 0.018601 |
| FAM53C | -3.62188 | 0.013654 |
| FAM53A | 3.727842 | 0.00091 |
| C10orf108 | -8.10928 | 0.009323 |
| ANO10 | 6.208699 | 0.007028 |
| GSTCD | -1.40468 | 0.008992 |
| PRUNE2 | -6.87923 | 0.009656 |
| SFRS6 | -4.87146 | 0.018601 |
| PEX11G | 2.865741 | 0.00091 |
| PARS2 | 0.465467 | 0.020829 |
| MCF2L2 | -3.32305 | 0.004236 |
| KCNK1 | -11.8237 | 0.003709 |
| PI4K2B | -3.58885 | 0.021545 |
| NHLRC1 | 0.466566 | 0.027455 |
| NHLRC4 | 2.318997 | 0.027096 |
| CXorf57 | -12.0404 | 0.003499 |
| SUSD4 | -10.2223 | 0.01825 |
| POLR2F | 1.79817 | 0.026476 |
| PTX3 | -6.43845 | 0.030037 |
| DOHH | 0.695785 | 0.018136 |
| OTUD6B | -7.80628 | 0.000201 |
| SNRNP200 | 1.255334 | 0.028796 |
| DDX5 | -2.19826 | 0.001114 |
| MYO15B | -4.45398 | 0.009544 |
| MYO15A | -4.99212 | 0.003186 |
| SLC7A8 | -1.11333 | 0.034005 |
| F8A1 | 1.504564 | 0.032148 |
| FMN2 | -4.1257 | 0.035265 |
| NFKBIZ | -4.1838 | 0.034005 |
| ARID4B | -6.18488 | 0.001835 |
| CRYZ | -5.91064 | 0.003814 |
| HAUS4 | 0.869042 | 0.034005 |
| HAUS2 | -5.36256 | 0.006487 |
| DLX1 | 7.784414 | 0.024264 |
| PACSIN2 | 3.56913 | 0.000706 |
| SNX16 | -2.36359 | 0.035015 |
| C13orf1 | -2.63606 | 0.000301 |
| HIST1H2BH | 4.880982 | 0.015606 |
| TMEM160 | 0.432559 | 0.00091 |
| TNFAIP1 | -1.96708 | 0.008117 |
| RTF1 | -2.17984 | 0.006377 |
| SILV | -2.08335 | 0.033753 |
| SDCBP | -2.0664 | 0.006161 |
| LMBR1 | -3.63675 | 0.007466 |
| RAB8B | -8.49407 | 0.002042 |
| RAB8A | 7.892416 | 0.00573 |
| NR6A1 | -4.0793 | 0.005194 |
| C6orf165 | -6.57189 | 0.031786 |
| PAQR3 | -13.9617 | 0.00091 |
| NME2P1 | 9.982709 | 0.002354 |
| FADS3 | 2.665618 | 0.03489 |
| CTDSP1 | 1.861846 | 0.007247 |
| AMZ2P1 | -2.02295 | 0.014801 |
| VWDE | -7.74656 | 0.049009 |
| C5orf28 | -1.68862 | 0.0179 |
| C5orf22 | -2.0079 | 0.003395 |
| C5orf24 | -9.11452 | 0.003814 |
| C5orf25 | -0.61896 | 0.045431 |
| C9orf68 | -4.86822 | 0.03413 |
| C9orf69 | 2.968518 | 0.0001 |
| BCL10 | -1.46637 | 0.004236 |
| UNC45A | 0.153367 | 0.00091 |
| DAND5 | 4.883759 | 0.025484 |
| ZCCHC17 | 4.293473 | 0.030164 |
| SUPT5H | 0.414388 | 0.009212 |
| TMEM87B | -5.14147 | 0.003081 |
| RPTN | 24.0332 | 0.000604 |
| ARHGEF19 | -11.1478 | 0.036283 |
| MOBKL1A | -2.50649 | 0.007247 |
| PAPPA | -4.46185 | 0.028181 |
| FXR2 | 0.083901 | 0.02462 |
| NEGR1 | -8.74586 | 0.002354 |
| SNAP91 | -0.37982 | 0.044634 |
| TNKS2 | -7.73488 | 0.039703 |
| CEP70 | -3.73617 | 0.022361 |
| C14orf43 | -1.36564 | 0.030408 |
| GPLD1 | 3.229165 | 0.027455 |
| RFK | -1.01297 | 0.00329 |
| ZNF511 | 6.375728 | 0.005194 |
| TRAPPC2P1 | -3.36625 | 0.016405 |
| NBLA00301 | -12.2741 | 0.00225 |
| WDR18 | 1.150706 | 0.001939 |
| GZMM | 2.367118 | 0.041137 |
| MSRB2 | 3.913869 | 0.008228 |
| ZNF26 | -1.75944 | 0.028054 |
| C20orf7 | -1.38606 | 0.01264 |
| LOC647288 | -9.61012 | 0.009875 |
| MAP4K5 | -3.84563 | 0.03489 |
| HMBS | 5.735009 | 0.006161 |
| HARS | 4.3484 | 0.049534 |
| GBF1 | 6.053685 | 0.018948 |
| TMEM63C | -12.0427 | 0.006813 |
| ZMYND17 | -1.16074 | 0.016866 |
| NAA30 | -3.06046 | 0.045294 |
| FITM2 | 2.207788 | 0.015833 |
| MIR17HG | -3.09652 | 0.044504 |
| RBM4 | 12.32132 | 0.003814 |
| CAT | 3.696713 | 0.047532 |
| ZBTB25 | -5.27709 | 0.019188 |
| AP1S3 | -11.7761 | 0.00329 |
| PGD | 4.347971 | 0.013088 |
| SNORA8 | -1.82099 | 0.036535 |
| LOC150786 | 4.048414 | 0.027578 |
| C21orf91 | -3.49445 | 0.031786 |
| NDUFC2 | 7.414664 | 0.021309 |
| C12orf63 | -3.13098 | 0.010097 |
| PTMA | 1.875262 | 0.047403 |
| ATP5J2 | 3.729589 | 0.031152 |
| SF4 | 1.618031 | 0.005944 |
| LOC100132831 | 6.872875 | 0.029165 |
| DYNLT3 | -0.05335 | 0.033259 |
| CBFB | -2.84956 | 0.001732 |
| TOMM7 | 3.398776 | 0.014113 |
| SIGLEC11 | -0.69116 | 0.007466 |
| GTF2F1 | 1.712078 | 0.034256 |
| CYLD | -5.67289 | 0.0179 |
| STARD10 | 2.345374 | 0.032644 |
| PROS1 | -12.3625 | 0.008117 |
| PPIP5K2 | -7.32289 | 0.001114 |
| HSBP1L1 | -1.43453 | 0.049145 |
| ZNF185 | -11.9983 | 0.004766 |
| TIFA | -6.52167 | 0.020005 |
| ZNF25 | -9.30553 | 0.000402 |
| BLVRB | 0.781153 | 0.023911 |
| GLTPD1 | 3.823307 | 0.000808 |
| PION | -2.73473 | 0.047815 |
| TCERG1 | -0.14972 | 0.023313 |
| SEMA4G | -3.04184 | 0.038409 |
| ZDHHC23 | -2.80424 | 0.038162 |
| RANBP9 | -1.43917 | 0.008228 |
| NAE1 | -2.30863 | 0.020122 |
| RANBP1 | 5.270991 | 0.014456 |
| RANBP3 | 4.420052 | 0.008775 |
| ZNF131 | -2.39773 | 0.031152 |
| ST7 | 8.354727 | 0.016754 |
| AGAP2 | 2.258928 | 0.042692 |
| STAB2 | -5.44849 | 0.006161 |
| ARNT | -1.63506 | 0.007357 |
| CCL4L2 | -0.1832 | 0.036283 |
| GDPD1 | -5.00666 | 0.002146 |
| C17orf96 | -7.53888 | 0.025743 |
| HOXD4 | -12.7865 | 0.009433 |
| UIMC1 | -4.08496 | 0.006051 |
| HDGFRP2 | 0.262181 | 0.019655 |
| HDGFRP3 | -3.86658 | 0.008665 |
| C6orf106 | 9.728116 | 0.017212 |
| ETS2 | -10.3181 | 0.003081 |
| LOC100302650 | -0.15407 | 0.045019 |
| PRR5-ARHGAP8 | -13.18 | 0.009656 |
| YWHAE | 3.218847 | 0.029659 |
| LOC100134259 | -11.4897 | 0.003395 |
| CHST15 | -5.76888 | 0.03591 |
| VAMP7 | -0.45669 | 0.038035 |
| PLDN | -6.77937 | 0.000604 |
| CREBZF | -1.85315 | 0.015374 |
| IER5 | -10.83 | 0.002769 |
| C9orf82 | -4.8702 | 0.003395 |
| WNK3 | -4.87575 | 0.009433 |
| USP37 | -2.85109 | 0.026974 |
| MORN5 | -1.28896 | 0.004236 |
| MORN4 | -7.88027 | 0.010422 |
| UBE2CBP | -6.88063 | 0.0226 |
| LOC342346 | -3.41189 | 0.014801 |
| PBXIP1 | 1.621771 | 0.013202 |
| EIF5AL1 | 4.860539 | 0.020829 |
| UCK1 | 2.261344 | 0.008228 |
| PLEKHB2 | -0.10877 | 0.011529 |
| SEC13 | 0.823797 | 0.039971 |
| TWF2 | 1.744917 | 0.006595 |
| C11orf58 | -1.05952 | 0.035142 |
| POLA2 | 4.475391 | 0.028676 |
| C18orf22 | 6.367254 | 0.000604 |
| ZMYND15 | -7.88586 | 0.024994 |
| NAV2 | -8.76698 | 0.021072 |
| TRUB1 | -2.85031 | 0.009544 |
| LOC401010 | 3.226172 | 0.01767 |
| C1orf9 | -3.74823 | 0.000604 |
| SLC44A3 | -9.81312 | 0.014801 |
| APEH | 6.203645 | 0.004343 |
| TRUB2 | 7.365351 | 0.00413 |
| CSNK1A1 | -4.71955 | 0.014227 |
| ATL3 | 1.557648 | 0.035785 |
| ATL2 | -2.10589 | 0.025987 |
| C14orf4 | -1.51333 | 0.043084 |
| RNMTL1 | 4.622187 | 0.001217 |
| CORO1C | 1.654348 | 0.039703 |
| TTC32 | -0.23498 | 0.045294 |
| FAS | -5.98222 | 0.019776 |
| NPAS2 | -9.06546 | 0.007682 |
| FAH | 2.323466 | 0.025867 |
| HNRNPA2B1 | 7.907859 | 0.000503 |
| TRIM9 | -8.60679 | 0.022841 |
| TPRXL | 2.967889 | 0.038162 |
| MON1A | 3.519041 | 0.000808 |
| NOP58 | -3.82073 | 0.025484 |
| MIDN | -3.80714 | 0.01503 |
| NCAPH2 | 0.860665 | 0.009323 |
| C14orf148 | -8.90507 | 0.00091 |
| TSPAN1 | 3.848212 | 0.008117 |
| ZMYM5 | -2.74128 | 0.013887 |
| HECTD2 | -2.52535 | 0.002977 |
| HECTD3 | 3.805184 | 0.009544 |
| MAN2B1 | 3.225421 | 0.009766 |
| TSPAN5 | -11.7313 | 0.01264 |
| FSTL4 | -3.94026 | 0.035785 |
| SLC41A1 | -1.81564 | 0.005408 |
| FLII | 2.491496 | 0.000808 |
| LRRC37B | -5.16722 | 0.008117 |
| COG3 | -0.80869 | 0.022361 |
| C12orf49 | -5.07911 | 0.008337 |
| MSTO1 | 11.50199 | 0.006161 |
| UBB | 6.272065 | 0.00329 |
| ANKRD50 | -2.27267 | 0.014227 |
| ANKRD57 | -12.9034 | 0.0001 |
| KBTBD2 | -1.98276 | 0.014 |
| NR4A2 | -5.57073 | 0.011418 |
| NR4A3 | -3.45532 | 0.001629 |
| RNF220 | 2.99555 | 0.004023 |
| C1QL3 | -9.81459 | 0.024499 |
| SIRT4 | 0.152703 | 0.006487 |
| WDR25 | 3.31152 | 0.007357 |
| ZMYM3 | -3.39081 | 0.014227 |
| RBM27 | -5.23784 | 0.024994 |
| SCN7A | -7.49615 | 0.002872 |
| EPHA3 | 6.146501 | 0.029911 |
| TRMT6 | -1.49231 | 0.020588 |
| FGF18 | 3.449384 | 0.028547 |
| RASSF3 | 0.231916 | 0.013088 |
| UGCG | -7.42066 | 0.001114 |
| USP18 | 6.865726 | 0.014456 |
| VAMP4 | -4.02368 | 0.005087 |
| GLB1L2 | -11.1799 | 0.045685 |
| SEMA3G | 0.03459 | 0.013088 |
| RAPGEF5 | -3.3536 | 0.032393 |
| ARL9 | -9.2846 | 0.003709 |
| ZC3H12A | -10.1119 | 0.004979 |
| SLC25A38 | 2.174376 | 0.015833 |
| SART3 | 0.33246 | 0.025241 |
| TMX1 | -2.39979 | 0.010206 |
| PI15 | -9.92007 | 0.027455 |
| C1QTNF7 | -3.11741 | 0.020829 |
| FAM175A | -6.78518 | 0.000604 |
| C17orf72 | 3.232728 | 0.022841 |
| C17orf75 | -1.79806 | 0.003186 |
| OTX1 | 4.13078 | 0.008665 |
| C17orf79 | 4.41828 | 0.003604 |
| MFAP1 | 9.577084 | 0.001423 |
| CDC42SE2 | -2.14128 | 0.021192 |
| CDC42SE1 | -2.15746 | 0.005408 |
| HEBP1 | 7.603507 | 0.00573 |
| LOC646851 | -8.17975 | 0.049404 |
| TMEM186 | 2.132385 | 0.009987 |
| ID2B | -5.56741 | 0.004448 |
| IWS1 | 4.459876 | 0.000301 |
| C19orf55 | -2.8108 | 0.030665 |
| C19orf54 | 8.161001 | 0.035142 |
| C22orf9 | 3.717351 | 0.012755 |
| PRR16 | -7.01297 | 0.006813 |
| TNFSF13 | 3.545605 | 0.002354 |
| RAD1 | -5.0165 | 0.000706 |
| SFXN1 | -2.15274 | 0.001012 |
| TIMM10 | 6.296875 | 0.01434 |
| C4orf47 | -9.9482 | 0.021431 |
| SPOPL | -2.26271 | 0.044253 |
| FAM50B | 1.267459 | 0.003604 |
| LOC645676 | -0.68202 | 0.037786 |
| ZC3H4 | 0.2709 | 0.012304 |
| 2-Mar | 3.415719 | 0.025241 |
| THOC2 | -4.58465 | 0.004236 |
| SLC4A5 | -7.49668 | 0.006487 |
| SLC4A4 | -2.35466 | 0.025609 |
| SLC4A7 | -4.84376 | 0.020829 |
| SLC4A1 | 10.93219 | 0.010641 |
| SLC4A3 | -11.2061 | 0.031285 |
| DCTN2 | 2.440533 | 0.049781 |
| ERC2 | -6.14087 | 0.015833 |
| TAF7 | -3.59526 | 0.035392 |
| ZBTB17 | 0.252613 | 0.009323 |
| PHTF2 | -5.35188 | 0.032148 |
| ANKRD45 | -5.48059 | 0.029407 |
| MYOC | 2.195062 | 0.032767 |
| ANKRD42 | -4.08637 | 0.019776 |
| APOL2 | 1.418482 | 0.017094 |
| ITGB5 | 7.887261 | 0.0001 |
| ITGB8 | -10.8194 | 0.041385 |
| YARS2 | 2.539944 | 0.028922 |
| SLC37A1 | -8.11149 | 0.029911 |
| NR2C1 | -4.22341 | 0.045948 |
| PLAC2 | -1.58357 | 0.040623 |
| URGCP | 1.9829 | 0.002042 |
| LBR | -4.39319 | 0.018829 |
| SLC30A2 | -1.82436 | 0.024383 |
| ST5 | -10.8792 | 0.021192 |
| VWCE | -6.56444 | 0.028304 |
| IMP3 | 5.942953 | 0.025609 |
| ATP1B1 | -11.78 | 0.02733 |
| TESK1 | 2.235959 | 0.049267 |
| TESK2 | -4.9495 | 0.043341 |
| LOC645332 | -8.21997 | 0.03591 |
| FAM164A | -8.41797 | 0.000808 |
| C14orf169 | 4.824766 | 0.00329 |
| C14orf162 | -0.97286 | 0.019071 |
| TCTA | 3.173044 | 0.011196 |
| RAB1B | 8.499561 | 0.000808 |
| RPL27A | 6.237883 | 0.023911 |
| TRMT1 | 1.219581 | 0.020471 |
| CASD1 | -1.76429 | 0.00573 |
| CCDC126 | -2.89511 | 0.000201 |
| CCDC125 | -2.26176 | 0.018483 |
| CCDC124 | 0.846864 | 0.025867 |
| CCDC122 | -9.42962 | 0.0001 |
| ZNF343 | 0.279849 | 0.025743 |
| C12orf24 | -2.82883 | 0.042306 |
| C12orf23 | -1.64334 | 0.014682 |
| TUBB2C | 2.977501 | 0.010861 |
| RNASEH2A | 7.946649 | 0.001423 |
| CEP170 | -10.0522 | 0.011082 |
| KIAA0284 | 0.152609 | 0.016521 |
| DTYMK | 10.55584 | 0.00413 |
| NEIL2 | 0.429563 | 0.000604 |
| MED28 | -2.39621 | 0.015143 |
| AHI1 | -7.87785 | 0.029911 |
| AKR1C3 | 0.506373 | 0.01343 |
| WHAMML2 | -10.5532 | 0.014227 |
| C1QBP | 3.621379 | 0.039836 |
| HNRNPL | 8.079997 | 0.019071 |
| ENAH | -1.42282 | 0.001939 |
| UFM1 | -2.81949 | 0.001114 |
| LAMP1 | 3.174792 | 0.003081 |
| TCF25 | 0.814376 | 0.041653 |
| NAT10 | 1.896696 | 0.029659 |
| ALDH2 | 3.985416 | 0.022009 |
| SCN9A | -5.19786 | 0.008447 |
| COQ4 | 3.049701 | 0.013542 |
| ASAH1 | 5.251546 | 0.042558 |
| PRR4 | 6.781256 | 0.026597 |
| PRR5 | 5.596639 | 0.00573 |
| ABCB6 | 4.787611 | 0.000301 |
| HMGA1 | 1.167922 | 0.035265 |
| RSPRY1 | -5.05585 | 0.038409 |
| ADH1A | -4.55502 | 0.039186 |
| SERPINF1 | -5.81769 | 0.010641 |
| KIAA1704 | -5.29739 | 0.004343 |
| MLEC | 2.411857 | 0.043724 |
| PSMC3IP | 5.633871 | 0.003604 |
| HLA-DPB2 | 3.289089 | 0.030408 |
| BZRAP1 | -9.25994 | 0.048352 |
| NUPL1 | -5.81343 | 0.001217 |
| NUPL2 | -2.07572 | 0.003604 |
| CHAC1 | 5.370379 | 0.00256 |
| CIAO1 | 3.847855 | 0.00413 |
| PARP8 | -6.57816 | 0.00573 |
| DPYS | -1.33036 | 0.018948 |
| TRPM4 | 3.191944 | 0.000301 |
| HERC4 | -3.41908 | 0.037536 |
| C19orf70 | 2.555797 | 0.046596 |
| APTX | -3.24249 | 0.004661 |
| FLJ22536 | -13.0809 | 0.002146 |
| C17orf59 | 2.811994 | 0.005087 |
| C11orf67 | 2.772047 | 0.024994 |
| C17orf55 | 1.583875 | 0.015833 |
| MKNK2 | 1.166312 | 0.01767 |
| LCLAT1 | -2.10784 | 0.038543 |
| ABCC6P2 | 4.284837 | 0.00091 |
| SEMA4F | 2.724629 | 0.006161 |
| SEC1 | 0.032962 | 0.048086 |
| LRP3 | 0.484763 | 0.045144 |
| LRP1 | 1.057922 | 0.011196 |
| CLCC1 | 0.20154 | 0.016059 |
| ANTXR2 | -3.80506 | 0.044634 |
| CCDC74A | 3.360438 | 0.013314 |
| MAP6 | -10.3829 | 0.009433 |
| TEAD2 | 1.222315 | 0.020829 |
| FAM180A | -11.3292 | 0.020122 |
| FAM180B | 3.281758 | 0.031285 |
| APEX1 | 6.998579 | 0.025987 |
| CYP4V2 | -2.37634 | 0.014456 |
| DYNC1H1 | 4.24651 | 0.008556 |
| THG1L | 6.372831 | 0.011418 |
| ZNF713 | -3.30517 | 0.003814 |
| VPS33A | 8.016691 | 0.013654 |
| AFG3L2 | 1.385072 | 0.015374 |
| TATDN3 | -0.21536 | 0.001423 |
| TROVE2 | -5.05387 | 0.006487 |
| AVIL | -4.28853 | 0.009766 |
| TLE2 | 0.480478 | 0.025241 |
| SERPINB8 | -2.38846 | 0.021431 |
| TYW1B | -2.82811 | 0.029284 |
| FAM19A2 | -3.43491 | 0.001526 |
| FAM19A1 | -2.3952 | 0.029532 |
| TUSC3 | -10.8934 | 0.01503 |
| ATP2B4 | -0.13952 | 0.006595 |
| CACNA2D4 | 2.556964 | 0.014113 |
| MTCH1 | 0.724545 | 0.042817 |
| ZNF80 | -0.30912 | 0.021778 |
| C6orf225 | -11.7569 | 0.015606 |
| LOC148413 | 1.093843 | 0.001835 |
| PIK3R2 | 5.371736 | 0.025241 |
| TIAL1 | -0.98543 | 0.00256 |
| HACE1 | -9.24692 | 0.047132 |
| HEXIM1 | 3.139761 | 0.023673 |
| DENND5A | 4.691797 | 0.034761 |
| ARMCX6 | 2.133484 | 0.048479 |
| AQP7P3 | -1.03312 | 0.041787 |
| C14orf104 | -10.041 | 0.002146 |
| C14orf101 | -1.28208 | 0.042172 |
| RAB3C | -5.43569 | 0.023313 |
| ARMCX5 | -6.43911 | 0.022841 |
| ATP8B5P | -3.63742 | 0.006269 |
| IDS | -5.98216 | 0.00413 |
| ARMC6 | 5.279742 | 0.001114 |
| NCAM2 | -1.78077 | 0.01343 |
| FAHD2B | 2.611497 | 0.040493 |
| CCDC103 | -5.72064 | 0.043209 |
| CCDC104 | -1.42394 | 0.030906 |
| CCDC107 | 1.917968 | 0.015143 |
| PML | 1.612328 | 0.010097 |
| FUBP1 | -4.38922 | 0.033383 |
| CECR5 | 1.207069 | 0.01377 |
| ARL6IP6 | -1.60383 | 0.009656 |
| FER | -6.83369 | 0.045431 |
| CHRNA3 | -3.17341 | 0.014 |
| ID2 | -10.419 | 0.0001 |
| ID3 | -4.78093 | 0.024032 |
| AARS | 5.042706 | 0.0001 |
| FAM18B2 | -3.91756 | 0.003814 |
| LOC100268168 | -5.96179 | 0.049921 |
| OPLAH | 2.226848 | 0.00225 |
| ANKRD2 | 7.26226 | 0.008665 |
| DMKN | -11.6646 | 0.042172 |
| SPATA2 | -5.59512 | 0.00413 |
| SPATA1 | -2.66945 | 0.027932 |
| LPHN1 | 1.140896 | 0.028676 |
| SPATA7 | -6.37431 | 0.035515 |
| ATPBD4 | -2.88741 | 0.007028 |
| RDBP | 5.035754 | 0.024994 |
| FOLR1 | -13.0616 | 0.029532 |
| RANGRF | 3.960631 | 0.029407 |
| TRAM1L1 | -5.56621 | 0.043591 |
| NR0B1 | -1.44881 | 0.041006 |
| PTPLA | -8.46739 | 0.011196 |
| TBC1D22A | 1.931493 | 0.037285 |
| RRBP1 | 1.175101 | 0.000808 |
| LIN52 | -5.94509 | 0.043341 |
| TRAPPC3 | 7.272473 | 0.005087 |
| PPAN-P2RY11 | -10.6343 | 0.04727 |
| SIM2 | -0.25521 | 0.028922 |
| PRRT1 | -0.14428 | 0.040493 |
| ST6GALNAC3 | -7.50942 | 0.009323 |
| LILRB5 | -5.62535 | 0.01434 |
| MTCP1 | -2.09493 | 0.036032 |
| PSMG4 | 5.222649 | 0.015488 |
| PSMG1 | -3.05168 | 0.023077 |
| KSR1 | -4.64332 | 0.024032 |
| DSEL | -5.45879 | 0.012755 |
| GRAPL | -5.31728 | 0.000808 |
| TACR2 | 2.375007 | 0.029783 |
| KCNH4 | -2.95238 | 0.048751 |
| KCNH3 | -7.09571 | 0.032027 |
| C9orf114 | 1.312456 | 0.014 |
| NDUFAF3 | 1.61301 | 0.009656 |
| GDAP1 | -12.2489 | 0.001217 |
| ZDHHC21 | -5.71667 | 0.000706 |
| DNALI1 | -6.28184 | 0.007357 |
| ZSWIM7 | 2.309992 | 0.010097 |
| AP3D1 | 1.702622 | 0.049781 |
| ZSWIM1 | -1.99537 | 0.038035 |
| ILDR1 | -15.4996 | 0.023553 |
| SULT1A2 | 4.230516 | 0.038677 |
| CABYR | -11.1778 | 0.003604 |
| C19orf10 | 1.17441 | 0.049009 |
| CDC40 | -4.6452 | 0.005087 |
| TECR | 4.474401 | 0.002977 |
| METAP1 | -4.61183 | 0.032895 |
| VPS53 | 0.2174 | 0.041261 |
| MUC4 | 2.243302 | 0.041916 |
| ZSCAN12P1 | -3.53935 | 0.001114 |
| CYB5A | 2.626335 | 0.028304 |
| TFEB | 3.59842 | 0.005087 |
| SS18L2 | -1.63733 | 0.039971 |
| IGDCC3 | -3.87211 | 0.042692 |
| DMRTC1B | -9.18781 | 0.015374 |
| SYTL3 | 1.726272 | 0.006487 |
| POMT1 | 0.63661 | 0.03227 |
| FAM182B | -5.44057 | 0.013542 |
| PRL | 8.603921 | 0.037536 |
| PRX | 0.192265 | 0.023673 |
| MRPL32 | -0.33435 | 0.04556 |
| FAM69A | 3.281905 | 0.04036 |
| THOC5 | 2.780835 | 0.001835 |
| PRRG1 | -3.62769 | 0.028054 |
| MTRR | -4.42463 | 0.004872 |
| LOC100129034 | 0.875726 | 0.025484 |
| YWHAG | 7.716031 | 0.032895 |
| FOXP3 | 2.772696 | 0.013654 |
| COPS8 | -0.41274 | 0.01175 |
| YTHDF3 | -6.58307 | 0.003395 |
| YTHDF1 | -0.78914 | 0.017094 |
| MYCN | -4.17967 | 0.035015 |
| TKT | 3.117524 | 0.031285 |
| VEZF1 | -1.3407 | 0.013887 |
| NUDT9 | 8.411294 | 0.006051 |
| TMED5 | -6.12988 | 0.005837 |
| NUDT1 | 4.517869 | 0.034005 |
| NUDT3 | 3.243949 | 0.046212 |
| NUDT6 | 3.368279 | 0.03894 |
| TFPI | -7.1741 | 0.018601 |
| TK2 | 3.914196 | 0.035641 |
| OR2B11 | 11.99271 | 0.020471 |
| PHLDB1 | 4.02826 | 0.015488 |
| LARS | -0.80652 | 0.040623 |
| MEF2A | -8.46608 | 0.015143 |
| SGSM3 | 1.339494 | 0.017212 |
| MAPKSP1 | -0.22048 | 0.01698 |
| ARID3A | -4.88564 | 0.004979 |
| MTMR11 | -7.65252 | 0.00132 |
| CELSR3 | -0.01504 | 0.045814 |
| ARID3C | 1.78383 | 0.037786 |
| NEK6 | 1.034547 | 0.047671 |
| CXADR | -12.8875 | 0.020829 |
| LRRC32 | -6.64242 | 0.022481 |
| MFN2 | 3.303788 | 0.007682 |
| MFN1 | -2.48097 | 0.025241 |
| THTPA | 4.686865 | 0.007682 |
| KTN1 | -9.80692 | 0.014 |
| C20orf199 | -0.72287 | 0.025867 |
| LRP2BP | -4.50475 | 0.035641 |
| FAM90A1 | 1.917754 | 0.041787 |
| PURB | -2.75816 | 0.007466 |
| RIC3 | -10.0089 | 0.026725 |
| ST3GAL5 | -2.4983 | 0.032027 |
| C7orf28A | -1.99428 | 0.025361 |
| TUFM | 3.200676 | 0.035785 |
| SLC16A2 | 2.145022 | 0.012191 |
| SLC16A7 | -2.45795 | 0.017094 |
| CEP135 | -3.86209 | 0.019655 |
| ARHGAP33 | -5.29158 | 0.007466 |
| CMKLR1 | 0.895678 | 0.016754 |
| LYVE1 | -9.3062 | 0.016175 |
| DYNLL2 | 1.427519 | 0.021072 |
| PIBF1 | -2.24079 | 0.036784 |
| LYPLA1 | -2.23702 | 0.001423 |
| KIF19 | -2.88006 | 0.01698 |
| PHB2 | 8.46161 | 0.004661 |
| RBM42 | 1.978859 | 0.041385 |
| RBM44 | -7.43864 | 0.006161 |
| RBM47 | -8.26921 | 0.030787 |
| TTC15 | 2.181177 | 0.0226 |
| TTC17 | -3.33942 | 0.018366 |
| FAM48A | -0.28477 | 0.000604 |
| C3orf62 | 0.4135 | 0.01264 |
| PPM1G | 8.558247 | 0.000301 |
| ABCF3 | 2.886826 | 0.000201 |
| ABCF1 | 10.2877 | 0.0001 |
| C4BPA | -7.25473 | 0.010206 |
| C4BPB | -0.01725 | 0.036032 |
| LOC728640 | -1.74193 | 0.016636 |
| DALRD3 | 7.498475 | 0.001526 |
| AHCYL1 | 7.121892 | 0.007247 |
| GRK6 | 1.190392 | 0.038677 |
| AGBL3 | -12.5629 | 0.000808 |
| AGBL2 | -4.42221 | 0.004555 |
| C15orf32 | 7.71033 | 0.025743 |
| SNAP25 | -3.94106 | 0.036913 |
| ZEB1 | -4.08443 | 0.025743 |
| C6 | -9.67275 | 0.001732 |
| C5 | -0.1003 | 0.013542 |
| FBXO41 | -9.09045 | 0.005194 |
| SNRPB2 | -0.17965 | 0.02462 |
| RPS6KA4 | 1.182641 | 0.025987 |
| NRBF2 | -4.33232 | 0.041916 |
| AGPAT5 | -6.29678 | 0.032027 |
| AGPAT9 | -0.92779 | 0.019655 |
| MCL1 | -1.5055 | 0.014682 |
| PPP2R1B | 9.568963 | 0.010861 |
| PEX10 | 4.525885 | 0.003814 |
| PEX13 | -3.54373 | 0.009987 |
| AP3B2 | -13.3325 | 0.0179 |
| FRAT1 | 5.411335 | 0.000503 |
| PCDHB12 | -11.8811 | 0.002042 |
| FAM154A | -2.5617 | 0.045294 |
| FAM154B | -5.4477 | 0.047 |
| PPP1CB | -1.96783 | 0.030665 |
| MPPED1 | -5.33691 | 0.047532 |
| C11orf20 | 5.059067 | 0.014456 |
| DCST2 | -5.92353 | 0.036535 |
| RFXANK | 6.61019 | 0.01264 |
| METTL2A | -2.37631 | 0.008005 |
| SPAG1 | -1.59924 | 0.002146 |
| RWDD4A | -1.37285 | 0.022481 |
| SLC34A2 | -11.129 | 0.045144 |
| C3orf37 | 6.799468 | 0.016754 |
| GPR25 | 5.148564 | 0.024383 |
| EIF3CL | 2.502534 | 0.006377 |
| NUCB1 | 1.37969 | 0.00573 |
| SOCS4 | -5.54537 | 0.020588 |
| SRPR | 1.629594 | 0.014227 |
| SOCS3 | -3.80152 | 0.00413 |
| CCL3L1 | -1.61 | 0.020951 |
| RBM7 | -7.66693 | 0.000808 |
| GREM1 | -3.55718 | 0.027578 |
| MAEA | 6.291628 | 0.020951 |
| PTS | -0.8681 | 0.03463 |
| MRPL11 | 9.956207 | 0.015488 |
| C16orf62 | 4.566203 | 0.020829 |
| DCUN1D3 | -0.47663 | 0.044634 |
| ITPR3 | 0.701572 | 0.030665 |
| CES1 | 1.345486 | 0.000301 |
| NOTCH3 | 1.875578 | 0.029659 |
| SNCG | 2.726189 | 0.001423 |
| TAF1D | -1.34454 | 0.00329 |
| AIFM2 | 4.451188 | 0.014801 |
| CES4 | 1.191969 | 0.034761 |
| POT1 | -4.52144 | 0.015606 |
| HBA1 | 1.634881 | 0.03227 |
| CD302 | -4.5485 | 0.005194 |
| OPN3 | -1.99393 | 0.026725 |
| SCAPER | -8.66018 | 0.020829 |
| HBA2 | 6.504917 | 0.008337 |
| REPS1 | -3.85826 | 0.029532 |
| RAB40AL | -4.57502 | 0.030164 |
| SIAH3 | -1.94163 | 0.001835 |
| EMILIN2 | 0.743362 | 0.027096 |
| PIAS2 | -4.43274 | 0.029911 |
| PIAS4 | 1.016329 | 0.025361 |
| TMEM98 | -10.0019 | 0.036784 |
| FAM115C | -8.13043 | 0.001939 |
| JAGN1 | 4.202483 | 0.025743 |
| ZNF317 | 1.697037 | 0.046874 |
| ZNF319 | 2.266676 | 0.001217 |
| P2RX1 | -2.77024 | 0.006595 |
| NRIP3 | -2.32635 | 0.012304 |
| LBXCOR1 | -2.64609 | 0.042043 |
| RPS19BP1 | 1.112939 | 0.015946 |
| LOC388955 | 2.863007 | 0.036155 |
| PGRMC1 | -0.62348 | 0.043984 |
| KIAA1530 | -0.03827 | 0.044253 |
| ALS2 | -6.39627 | 0.03489 |
| KIAA1024 | -8.29891 | 0.027578 |
| LRRC57 | -0.53529 | 0.021192 |
| DENND1A | 5.63999 | 0.039836 |
| SAC3D1 | 4.552545 | 0.004766 |
| CDRT4 | 1.640767 | 0.046874 |
| CCDC140 | 8.163485 | 0.031529 |
| CCDC148 | -12.2158 | 0.009212 |
| GRAMD3 | -9.18509 | 0.030164 |
| SRPK1 | -1.45674 | 0.046596 |
| GOLGA9P | -3.06024 | 0.022841 |
| GRWD1 | 1.635948 | 0.008665 |
| PTGR1 | 3.978597 | 0.00573 |
| IFRD1 | -4.74498 | 0.041137 |
| IFRD2 | 2.090859 | 0.002769 |
| OSBPL3 | -8.45796 | 0.003395 |
| RAB11FIP4 | -9.52332 | 0.048612 |
| SIRT2 | 1.322462 | 0.021192 |
| C1orf27 | -2.35707 | 0.015833 |
| GPR137C | -0.13417 | 0.031285 |
| BMS1 | 3.390438 | 0.009766 |
| ZDBF2 | -9.93439 | 0.042431 |
| SLC9A7 | -15.3475 | 0.003499 |
| TP53RK | -7.57305 | 0.001217 |
| THBD | -3.92899 | 0.042043 |
| SLC38A4 | -2.42476 | 0.016866 |
| UBE2D4 | 7.561311 | 0.009544 |
| SCN5A | -8.858 | 0.029284 |
| ME1 | -2.89243 | 0.033383 |
| QPCTL | -5.49132 | 0.013654 |
| SLC38A6 | -1.38441 | 0.001526 |
| TIPRL | -2.19119 | 0.002665 |
| ITPRIP | -3.15491 | 0.024383 |
| SLC22A20 | -7.09084 | 0.002457 |
| FLJ43663 | -11.5128 | 0.004661 |
| CCBE1 | -9.11242 | 0.00225 |
| AADACL2 | 11.53876 | 0.006703 |
| C4orf49 | -0.0031 | 0.040752 |
| ATPAF2 | 8.928819 | 0.007466 |
| LARP1B | -1.1799 | 0.045814 |
| MCRS1 | 4.296517 | 0.036409 |
| C10orf82 | -8.05531 | 0.018714 |
| GJB6 | 13.04079 | 0.004448 |
| PSMC2 | 7.945085 | 0.005408 |
| OR6B3 | 14.5354 | 0.012527 |
| ARFIP1 | -0.22366 | 0.031285 |
| ARFIP2 | 2.556975 | 0.017785 |
| KIF18A | -1.32118 | 0.015488 |
| IFT172 | 0.192002 | 0.040493 |
| DNAL1 | -6.1392 | 0.000706 |
| CLSTN2 | 3.497392 | 0.015606 |
| EIF3I | 12.79268 | 0.008883 |
| STYK1 | -7.69352 | 0.015374 |
| EIF3A | 0.502302 | 0.010641 |
| EIF3B | 7.461178 | 0.01175 |
| EIF3D | 4.281226 | 0.01629 |
| EIF3G | 9.481169 | 0.003918 |
| FADD | 7.780128 | 0.041137 |
| NOL10 | 2.398997 | 0.049404 |
| CDKN2B | 3.12585 | 0.014227 |
| EDEM3 | -4.75253 | 0.035785 |
| EDEM1 | -3.70454 | 0.015143 |
| SMG6 | 0.342752 | 0.004555 |
| NME2 | 7.702853 | 0.027932 |
| BRP44L | -1.66528 | 0.024864 |
| TRMT11 | -3.63189 | 0.004236 |
| CSTF2T | 0.178048 | 0.025241 |
| CCL2 | -1.95717 | 0.022009 |
| DHRS7C | 12.36385 | 0.028181 |
| ZNF75D | -3.6954 | 0.011418 |
| NUBP1 | 5.901036 | 0.009101 |
| SCML1 | -8.91497 | 0.00091 |
| SCOC | -1.63447 | 0.046212 |
| TCTE1 | -7.72763 | 0.001629 |
| SPEG | -8.8465 | 0.038284 |
| ALKBH4 | 3.06744 | 0.018601 |
| ALKBH2 | 3.190542 | 0.006703 |
| MPZL3 | -11.3387 | 0.008665 |
| ARHGDIG | 14.33268 | 0.000503 |
| DNAJB9 | -5.70407 | 0.002042 |
| EPB49 | 5.09031 | 0.002354 |
| FRY | -5.34763 | 0.041524 |
| TNIP1 | 6.681231 | 0.01503 |
| GRIK1 | -3.59604 | 0.028304 |
| GRIK4 | -1.02518 | 0.035515 |
| RNF149 | -2.66151 | 0.005301 |
| RASGRF1 | -4.27594 | 0.000201 |
| RNF19A | -2.46616 | 0.029165 |
| C16orf42 | 1.588244 | 0.023553 |
| COPG | 9.055787 | 0.001012 |
| SYBU | 2.269751 | 0.026476 |
| CDK20 | -10.7463 | 0.014 |
| SHC4 | -5.62261 | 0.044891 |
| TOP1P2 | -5.43721 | 0.01053 |
| POFUT1 | 8.95412 | 0.004023 |
| CDKN2AIP | -1.4529 | 0.01377 |
| LOC399744 | -3.29993 | 0.046212 |
| TIAM2 | -9.44104 | 0.002146 |
| TGS1 | -1.90096 | 0.011529 |
| RIBC2 | -3.64639 | 0.041524 |
| CHERP | 1.738435 | 0.003604 |
| GRINL1A | -2.78465 | 0.019776 |
| INPP5K | 2.362148 | 0.045294 |
| RAI14 | -2.98833 | 0.028054 |
| PID1 | -10.5961 | 0.00225 |
| CNST | -4.1513 | 0.010752 |
| HELQ | -3.53913 | 0.009212 |
| EPB42 | 1.348514 | 0.020951 |
| HELB | -2.27491 | 0.010641 |
| VPS37A | -7.49411 | 0.028054 |
| PNPLA6 | 1.28249 | 0.04727 |
| PNPLA4 | 3.670017 | 0.041787 |
| PNPLA8 | -5.7399 | 0.016405 |
| NCKAP1 | -7.21636 | 0.025867 |
| WEE1 | -10.0107 | 0.001423 |
| UNC5A | -7.80015 | 0.000301 |
| FOS | -4.47887 | 0.04727 |
| KATNAL1 | -2.92445 | 0.036283 |
| ATG12 | -1.58003 | 0.003918 |
| TSFM | 4.206694 | 0.013202 |
| PES1 | 5.107776 | 0.006813 |
| SNED1 | -4.6031 | 0.019071 |
| CASK | -1.63345 | 0.006377 |
| PKNOX1 | -2.10616 | 0.018016 |
| ZBTB41 | -3.98691 | 0.023313 |
| ZNF295 | -4.11895 | 0.01053 |
| RNPEPL1 | 1.478239 | 0.008665 |
| GSTM2 | 0.346202 | 0.035785 |
| C2orf7 | 1.334249 | 0.018948 |
| GSTM4 | 0.22548 | 0.007791 |
| PIP4K2C | -6.30139 | 0.037662 |
| TMSB15A | -5.84865 | 0.034005 |
| LPAR3 | -4.93401 | 0.026848 |
| PINX1 | 0.894409 | 0.00091 |
| AEBP2 | -4.57215 | 0.002042 |
| RCHY1 | -4.44431 | 0.004555 |
| CBR1 | 7.30992 | 0.00225 |
| LTB | 4.292579 | 0.002146 |
| ABHD12B | -0.15156 | 0.035142 |
| ANXA5 | 5.080619 | 0.04036 |
| TRAF3IP1 | 3.634494 | 0.011196 |
| MCM7 | 4.296433 | 0.001114 |
| MCM5 | 5.191722 | 0.00132 |
| MCM4 | -0.37352 | 0.047132 |
| DUSP16 | -6.07659 | 0.028922 |
| DUSP10 | -5.09517 | 0.001217 |
| SQSTM1 | 3.389704 | 0.014 |
| ZNF541 | 2.497241 | 0.044253 |
| C3orf23 | -0.98018 | 0.048086 |
| LOC341056 | 2.007676 | 0.015143 |
| MS4A7 | -1.53129 | 0.04036 |
| CCDC102A | 0.354528 | 0.044504 |
| DSC1 | -7.06586 | 0.046464 |
| PYGL | 3.49634 | 0.032895 |
| LOC283922 | -4.07715 | 0.005514 |
| RAB27A | -8.95184 | 0.001732 |
| C20orf29 | 3.331401 | 0.008228 |
| BAMBI | -9.75626 | 0.004343 |
| TYSND1 | 0.785689 | 0.038543 |
| CCDC68 | -13.3533 | 0.000604 |
| IL1A | -5.02502 | 0.000402 |
| YPEL5 | -2.08335 | 0.042306 |
| POLR3H | 0.415912 | 0.008117 |
| PIM1 | -2.25666 | 0.006813 |
| SLC25A40 | -6.80556 | 0.008228 |
| FAM150B | -4.0688 | 0.040878 |
| BEST1 | 4.775183 | 0.010641 |
| TNPO3 | 1.286372 | 0.006487 |
| GCN1L1 | 0.706319 | 0.027932 |
| MAPK6 | -4.01506 | 0.001217 |
| MAPK8 | -3.14697 | 0.027455 |
| SGPP2 | -7.18993 | 0.013314 |
| KIAA1804 | -10.0431 | 0.027455 |
| SERINC3 | -5.47346 | 0.004023 |
| THNSL2 | 0.400573 | 0.031285 |
| STAT5B | 1.656879 | 0.019071 |
| FDX1L | 3.914946 | 0.040111 |
| KCTD1 | -9.91774 | 0.041524 |
| MED9 | 2.401901 | 0.002457 |
| FOXRED1 | 0.689412 | 0.01264 |
| KCNT2 | -9.35026 | 0.000706 |
| LOC374443 | -3.42026 | 0.025361 |
| ZNF432 | -3.51491 | 0.016405 |
| CABLES2 | -4.73804 | 0.015946 |
| MTUS2 | -8.55351 | 0.000201 |
| ESF1 | -4.60323 | 0.043084 |
| COMT | 2.998604 | 0.028922 |
| RNF167 | 9.991828 | 0.005621 |
| SEC23A | -0.417 | 0.040878 |
| SYNPO | 2.185099 | 0.018601 |
| ANP32E | -5.35495 | 0.010971 |
| ANP32B | 1.717048 | 0.007138 |
| ANP32C | 4.116935 | 0.02474 |
| AGPHD1 | -5.54175 | 0.016636 |
| ATRNL1 | -0.35893 | 0.015143 |
| STK11 | 0.411098 | 0.011862 |
| ZNF680 | -2.18172 | 0.040111 |
| PRKG2 | -3.54204 | 0.008775 |
| ZNF684 | -2.05863 | 0.045431 |
| TMEM140 | 4.680841 | 0.015718 |
| PCDHGC5 | -6.33124 | 0.014913 |
| IQCH | -7.06489 | 0.023789 |
| MMP15 | 3.653812 | 0.046731 |
| DSTYK | -5.27371 | 0.013887 |
| MMP19 | -0.49729 | 0.046596 |
| PRG4 | -2.82745 | 0.017441 |
| PRG2 | -4.97204 | 0.017554 |
| NF2 | 0.025875 | 0.049921 |
| TMEM27 | -6.63983 | 0.049534 |
| CDAN1 | 2.006189 | 0.016754 |
| FAM133B | -1.63716 | 0.006921 |
| ZNF583 | -4.73458 | 0.007138 |
| FAM133A | -5.19223 | 0.000808 |
| C7orf25 | 0.191972 | 0.005194 |
| SRGAP3 | -4.42449 | 0.034505 |
| SRGAP2 | -2.76437 | 0.049404 |
| AP1AR | -11.6965 | 0.0001 |
| NEDD8 | 2.52612 | 0.048352 |
| NEDD4 | -5.00177 | 0.017441 |
| PCDHB14 | -8.62187 | 0.035785 |
| SLC7A2 | -4.55316 | 0.041261 |
| LEPR | -9.21256 | 0.002042 |
| CPOX | -4.84929 | 0.043084 |
| ALG12 | 7.877077 | 0.008117 |
| ALG13 | -4.39531 | 0.004023 |
| RIOK3 | -5.24087 | 0.005301 |
| RBMS1 | -9.4974 | 0.018829 |
| AHSP | 14.33229 | 0.016866 |
| ST7OT1 | -7.23671 | 0.001526 |
| WIPI2 | 2.674283 | 0.030037 |
| TMEM217 | -1.24365 | 0.023433 |
| CDC25B | -1.16959 | 0.040878 |
| TMEM214 | 6.580255 | 0.002042 |
| KLF10 | -4.95608 | 0.000201 |
| NKPD1 | -12.3312 | 0.01698 |
| EVPLL | 6.463333 | 0.006161 |
| CAMK2N1 | -12.5498 | 0.001114 |
| DAB1 | 6.219641 | 0.032027 |
| SYNJ2 | -8.20257 | 0.017325 |
| KIAA1468 | -1.59469 | 0.047815 |
| EEFSEC | 1.889962 | 0.014801 |
| TOR1AIP1 | -5.93165 | 0.011082 |
| RAB5A | -4.20372 | 0.029165 |
| KPTN | 4.560706 | 0.001939 |
| HNRPDL | -6.3797 | 0.0001 |
| SAMSN1 | -2.73829 | 0.037285 |
| PCOTH | 1.054472 | 0.011862 |
| ANXA2 | 5.845699 | 0.002457 |
| TPST2 | 3.41299 | 0.024994 |
| DDX51 | 1.57719 | 0.005837 |
| CSRNP1 | -7.17635 | 0.006487 |
| CSRNP2 | -0.04824 | 0.002665 |
| GATM | -2.60137 | 0.019893 |
| CDC42BPA | -9.68031 | 0.012304 |
| NUPR1 | 2.511333 | 0.006161 |
| EGLN1 | -7.73241 | 0.007466 |
| TP53BP2 | -3.89957 | 0.031659 |
| PSMD8 | 1.894049 | 0.01343 |
| MCM3 | 2.296631 | 0.016521 |
| MCM2 | 2.166275 | 0.034761 |
| TMEM136 | -3.21399 | 0.007466 |
| CCDC41 | -5.16224 | 0.001939 |
| HSD17B11 | -9.07409 | 0.001012 |
| UTY | 16.8069 | 0.025743 |
| CDKL3 | -3.36816 | 0.019541 |
| CDKL2 | -11.4595 | 0.014113 |
| FBXO21 | -1.56479 | 0.024032 |
| FBXO27 | 0.102411 | 0.046731 |
| GNB5 | -8.6874 | 0.028426 |
| AMBRA1 | 0.522574 | 0.034505 |
| UTP18 | -3.35163 | 0.032148 |
| THAP2 | -0.94647 | 0.017785 |
| RBMX | -4.91387 | 0.016754 |
| TRIM37 | -0.91529 | 0.042692 |
| HSPB11 | -0.77702 | 0.017785 |
| PCMTD2 | -1.48391 | 0.010097 |
| YKT6 | 5.590883 | 0.004343 |
| VPS18 | 1.58763 | 0.019541 |
| NUP62CL | -11.4022 | 0.013887 |
| CSTF2 | 3.491319 | 0.008883 |
| ILF3 | 1.93691 | 0.00329 |
| C1orf126 | -15.254 | 0.00091 |
| C1orf124 | -2.33025 | 0.008117 |
| C1orf123 | 3.234707 | 0.035785 |
| C1orf128 | 0.596082 | 0.012755 |
| UBXN4 | -6.26573 | 0.005301 |
| C6orf94 | 6.005862 | 0.029911 |
| CLN6 | 5.768312 | 0.006921 |
| XK | -11.34 | 0.011862 |
| UBE2K | -1.14097 | 0.01457 |
| EIF3IP1 | 3.658715 | 0.025109 |
| APBB1IP | 1.600803 | 0.04476 |
| CCDC163P | -4.16032 | 0.021309 |
| SYT13 | -6.80999 | 0.030787 |
| GEM | -9.24742 | 0.000706 |
| RIC8A | 1.809223 | 0.01503 |
| SPATA22 | 3.18951 | 0.022841 |
| WWC3 | -0.58362 | 0.032644 |
| ATP6V1G1 | -6.16414 | 0.011082 |
| GPR173 | -7.15654 | 0.01629 |
| GPR176 | 1.802009 | 0.035392 |
| C2CD4A | -3.4522 | 0.001629 |
| SERP2 | -9.84633 | 0.020829 |
| OBP2A | 9.238799 | 0.009656 |
| SS18L1 | -5.3329 | 0.005944 |
| KBTBD11 | -4.80578 | 0.049404 |
| OR1E1 | 7.909787 | 0.028676 |
| CHAF1A | 9.041368 | 0.0001 |
| HSD11B1L | 3.9703 | 0.036663 |
| GLE1 | 3.806926 | 0.026974 |
| SPTAN1 | 3.792604 | 0.001526 |
| CSAG1 | 15.0147 | 0.026725 |
| PRELID2 | -5.64186 | 0.003186 |
| DDX3Y | 17.7988 | 0.002665 |
| FAM171B | -8.49242 | 0.00132 |
| TERT | 9.562219 | 0.007791 |
| ZNF362 | 0.480793 | 0.007791 |
| KIAA0247 | -9.0607 | 0.013887 |
| TMBIM1 | 2.275713 | 0.01343 |
| DRD5 | 11.21378 | 0.030408 |
| PACRGL | -3.0423 | 0.005621 |
| FAM18B | -4.63101 | 0.029407 |
| WDR52 | -0.08728 | 0.027578 |
| PYGB | 12.50539 | 0.000201 |
| DOLPP1 | 8.660359 | 0.000706 |
| AKT1 | 1.460969 | 0.030665 |
| LOC90586 | -1.99916 | 0.025109 |
| MRC1 | -7.89972 | 0.007357 |
| CHML | -5.03157 | 0.007138 |
| SIAE | 3.614521 | 0.018601 |
| LOC149837 | 3.438214 | 0.022481 |
| ENTPD3 | 4.183745 | 0.016175 |
| SNAPC1 | -4.37473 | 0.002146 |
| SNAPC5 | 3.02031 | 0.016405 |
| TNFRSF10B | -3.24837 | 0.001217 |
| HYAL3 | 3.156962 | 0.03463 |
| SNAPIN | 2.375619 | 0.042306 |
| PKIA | -6.58287 | 0.007898 |
| DPRXP4 | -3.02499 | 0.002769 |
| DCTN1 | 0.49744 | 0.0001 |
| RRN3P2 | -2.69055 | 0.022481 |
| DCTN4 | -3.27564 | 0.041524 |
| RPS4Y1 | 30.4426 | 0.000201 |
| KIF3A | -10.5819 | 0.000503 |
| WNT5A | -3.11478 | 0.015488 |
| VEZT | -2.67076 | 0.008992 |
| USP38 | -3.54223 | 0.040493 |
| UBXN1 | 1.80842 | 0.021431 |
| PGM2L1 | -6.50581 | 0.0001 |
| MRPS30 | -1.35719 | 0.003918 |
| HSPA12A | 1.9925 | 0.026228 |
| SIGLEC15 | -1.80173 | 0.042043 |
| KIAA2013 | 6.28982 | 0.001732 |
| HNRNPUL2 | 2.716543 | 0.0001 |
| HNRNPUL1 | 4.341809 | 0.00413 |
| ZG16 | 12.40938 | 0.01698 |
| CNTLN | -8.26585 | 0.022481 |
| EMP1 | -4.62587 | 0.040111 |
| AKR1CL1 | 12.38491 | 0.015258 |
| CNTNAP1 | -3.7881 | 0.042172 |
| DCAF11 | 4.612888 | 0.004236 |
| ZNF556 | -1.27382 | 0.016175 |
| APOB48R | 4.944012 | 0.000301 |
| KCTD21 | 4.389672 | 0.01434 |
| TRIM17 | -9.62454 | 0.016521 |
| UTP23 | -4.61986 | 0.004236 |
| KIAA1430 | -8.30184 | 0.012527 |
| NMD3 | -3.58294 | 0.010097 |
| TRIM16 | 5.712752 | 0.003709 |
| OPRM1 | 13.0466 | 0.015258 |
| KIAA0528 | -1.09284 | 0.018948 |
| IFNAR1 | -2.16906 | 0.018136 |
| GPM6A | -4.13351 | 0.01503 |
| ANXA2P1 | 4.751666 | 0.001629 |
| ANXA2P3 | 2.236904 | 0.006813 |
| ANXA2P2 | 5.875377 | 0.001939 |
| IRAK4 | -5.54632 | 0.001012 |
| WDR5 | 10.31499 | 0.000604 |
| SATB2 | -4.96865 | 0.014227 |
| GATA6 | -2.46184 | 0.023673 |
| POLR3G | -4.51261 | 0.039836 |
| TMEM192 | -5.4694 | 0.019655 |
| CEP290 | -5.48827 | 0.037662 |
| TTC39B | -9.16551 | 0.002146 |
| TCF19 | 2.044407 | 0.023673 |
| UROD | 3.507362 | 0.01434 |
| SERPINE1 | -0.40426 | 0.023911 |
| SOX15 | -7.58881 | 0.022241 |
| DMAP1 | 4.264595 | 0.00132 |
| SNX33 | 4.522363 | 0.00132 |
| SNX32 | -0.90709 | 0.0179 |
| IRGQ | -0.64471 | 0.033013 |
| INSIG1 | -1.77205 | 0.024864 |
| LDLRAP1 | 4.271326 | 0.006161 |
| C8orf37 | -6.5028 | 0.001629 |
| KLHL25 | 0.287393 | 0.008228 |
| KLHL26 | 1.699211 | 0.009987 |
| CAPNS1 | 5.516127 | 0.01457 |
| KLHL20 | -3.404 | 0.026597 |
| CAPNS2 | 12.04044 | 0.003395 |
| POLG | 0.562793 | 0.01698 |
| POLB | -4.60057 | 0.010971 |
| TAF15 | 2.604865 | 0.002457 |
| MDK | -3.56872 | 0.046074 |
| NDUFV1 | 1.052721 | 0.01825 |
| LOC375190 | -2.49989 | 0.048479 |
| SNHG3-RCC1 | -5.62588 | 0.039836 |
| LOC100133991 | -6.08066 | 0.007247 |
| GPR153 | 1.094472 | 0.007574 |
| C13orf27 | -1.53533 | 0.00573 |
| GPR158 | -3.04099 | 0.028796 |
| COBRA1 | 2.436575 | 0.00256 |
| UBE2B | -3.79066 | 0.000503 |
| PPFIA4 | -1.61514 | 0.023313 |
| PPFIA2 | -4.57652 | 0.031659 |
| HSD17B8 | 3.234569 | 0.005944 |
| PRMT3 | -3.86435 | 0.006051 |
| HSD17B4 | 3.921351 | 0.020705 |
| RTN4 | 6.436143 | 0.038677 |
| HGF | -5.70246 | 0.036283 |
| LIN54 | -9.36039 | 0.001012 |
| C7orf60 | -3.11995 | 0.026848 |
| IER3 | -7.53073 | 0.000706 |
| CILP | 1.03113 | 0.024864 |
| TMEM41B | -3.73553 | 0.00091 |
| IL1RL1 | -0.44675 | 0.011082 |
| IL1RL2 | -11.0939 | 0.01175 |
| PKP2 | -14.7456 | 0.005837 |
| NUDT21 | -2.69628 | 0.025109 |
| FAM173B | 6.682583 | 0.039571 |
| STARD3NL | -1.95644 | 0.016175 |
| LYPD5 | -8.9562 | 0.040111 |
| LPO | -2.34995 | 0.038677 |
| DDAH2 | 2.338683 | 0.002769 |
| MBLAC1 | 2.83697 | 0.045948 |
| CRELD2 | 1.12851 | 0.031152 |
| SLC29A2 | 0.253949 | 0.008665 |
| NKAP | -2.03899 | 0.008775 |
| SPHAR | -5.32689 | 0.034005 |
| C6orf203 | 3.303721 | 0.039703 |
| GALNT4 | -2.40906 | 0.025609 |
| GALNT7 | -5.05291 | 0.036155 |
| PAAF1 | 10.00845 | 0.009875 |
| FAM103A1 | -2.99929 | 0.020829 |
| NLRP5 | 2.961389 | 0.021309 |
| NLRP7 | -4.30347 | 0.013088 |
| FASTKD3 | -2.79513 | 0.004343 |
| PSEN1 | 6.494415 | 0.029532 |
| NLRP9 | -3.71732 | 0.039186 |
| RAB18 | -2.34285 | 0.007247 |
| LRAT | -12.2978 | 0.016175 |
| MAPK4 | -8.19666 | 0.039971 |
| XRCC6 | 7.485556 | 0.012304 |
| CD101 | -3.8838 | 0.011971 |
| KIF1C | 4.306645 | 0.008992 |
| STX16 | -1.21126 | 0.02733 |
| SPATC1 | -2.3047 | 0.022241 |
| SNHG4 | -4.40364 | 0.012304 |
| ERCC3 | -3.98357 | 0.025609 |
| SPIRE2 | -7.07684 | 0.042431 |
| CD248 | 1.54688 | 0.002042 |
| PFKL | 0.272651 | 0.032516 |
| TNNI1 | -10.4654 | 0.043463 |
| LMAN2L | 1.539429 | 0.019776 |
| KCND2 | -5.68202 | 0.049145 |
| ABHD14A | 1.80898 | 0.020705 |
| SMPDL3A | -2.83606 | 0.011971 |
| USP46 | -1.79014 | 0.001012 |
| BHLHE40 | -8.41812 | 0.004979 |
| MAP4K3 | -7.04516 | 0.004236 |
| PYCARD | 4.931205 | 0.007247 |
| MAGI2 | -7.74373 | 0.048352 |
| LAS1L | 5.232016 | 0.008005 |
| SLC25A20 | 4.656799 | 0.004555 |
| SLC25A24 | -1.72449 | 0.006269 |
| POP5 | 0.11156 | 0.03489 |
| SMUG1 | 8.318244 | 0.001423 |
| COPB2 | 5.228635 | 0.001423 |
| ISG20L2 | -4.81612 | 0.01457 |
| KDELC2 | -3.94108 | 0.039971 |
| FLVCR1 | -2.29675 | 0.027692 |
| LOC100240734 | 6.472313 | 0.020237 |
| PCDHB6 | -6.31838 | 0.024994 |
| USP15 | -6.18271 | 0.00329 |
| HSPA4L | -11.8064 | 0.006703 |
| SDHAP1 | -3.09267 | 0.021545 |
| SMAGP | -6.27404 | 0.02474 |
| RPUSD4 | 0.679306 | 0.005087 |
| RPAP3 | -0.03202 | 0.026228 |
| RPUSD1 | 3.968808 | 0.013202 |
| RPUSD3 | 8.001897 | 0.005408 |
| OR10A3 | 2.113954 | 0.030037 |
| SNORA5A | 12.75966 | 0.042558 |
| C6orf57 | -4.92773 | 0.014682 |
| PCDHGA3 | -6.67456 | 0.028304 |
| DOCK10 | -3.0234 | 0.020122 |
| SNX17 | 3.200021 | 0.001732 |
| SNX13 | -6.50775 | 0.045948 |
| SNX19 | 2.03618 | 0.047815 |
| ICAM5 | -7.49463 | 0.043724 |
| ICAM3 | 1.664123 | 0.039836 |
| LOC152217 | 11.82407 | 0.002872 |
| EBNA1BP2 | 3.988955 | 0.003081 |
| CREG2 | -2.01482 | 0.000604 |
| CYCS | -6.86623 | 0.0001 |
| STK32A | -7.31239 | 0.007574 |
| LOC100170939 | -9.16081 | 0.005837 |
| PDCD2L | 1.570534 | 0.030284 |
| FAM36A | -1.92327 | 0.022481 |
| C2orf71 | 0.619375 | 0.002872 |
| C2orf77 | -7.62524 | 0.000503 |
| C2orf79 | 6.522552 | 0.020353 |
| NRG4 | -0.42919 | 0.032393 |
| NGFR | -9.37903 | 0.048612 |
| PARP1 | 5.4638 | 0.030665 |
| PON3 | -6.85119 | 0.004448 |
| COMMD10 | -2.68598 | 0.01377 |
| GFRAL | 13.27799 | 0.035515 |
| GOLGA6L10 | -6.27099 | 0.006487 |
| NRAP | 11.83865 | 0.048086 |
| UGP2 | 4.87339 | 0.043984 |
| SLC38A11 | -5.83443 | 0.01343 |
| NAPG | -1.67421 | 0.043724 |
| ITSN1 | 5.430163 | 0.028426 |
| ITSN2 | -6.39735 | 0.016059 |
| SCAMP1 | -3.65219 | 0.006595 |
| PDCD2 | -1.75654 | 0.02733 |
| CXADRP3 | -1.38733 | 0.031285 |
| C7orf40 | -4.46606 | 0.019071 |
| C7orf49 | 0.29993 | 0.000402 |
| L3MBTL2 | 3.43044 | 0.022361 |
| C9orf3 | -4.70515 | 0.006703 |
| NAV1 | 6.610586 | 0.020705 |
| CPA4 | -6.57783 | 0.019776 |
| OS9 | 1.284517 | 0.011418 |
| LNX2 | -4.22809 | 0.002769 |
| ASMTL | 0.497894 | 0.031029 |
| VCP | 2.201652 | 0.018829 |
| LOC284900 | -3.12865 | 0.045948 |
| C6orf195 | -0.26803 | 0.028181 |
| EHD2 | 1.145208 | 0.02462 |
| GMFB | -6.03316 | 0.008556 |
| VLDLR | 0.425867 | 0.031786 |
| TMED7 | -8.59009 | 0.002042 |
| HNRNPM | 5.810042 | 0.0001 |
| DIS3L | 1.644477 | 0.026476 |
| HNRNPF | -3.69561 | 0.009433 |
| GOLM1 | -4.06513 | 0.043857 |
| SLC6A15 | -7.19383 | 0.036155 |
| TADA3 | 2.084654 | 0.043591 |
| ZNF174 | 3.033883 | 0.031152 |
| LOC150381 | 0.459678 | 0.025987 |
| POMC | 1.962801 | 0.033013 |
| RAB35 | 0.943147 | 0.037411 |
| RAB39 | -4.87552 | 0.01434 |
| LCA5L | -1.36988 | 0.047671 |
| DOK1 | 5.569397 | 0.007574 |
| DOK6 | -6.96424 | 0.031285 |
| CD163L1 | 0.882199 | 0.032393 |
| PLS1 | -15.0511 | 0.004872 |
| CAMSAP1L1 | -8.74879 | 0.009987 |
| PDIA2 | 8.359769 | 0.014113 |
| TRIM26 | 1.83462 | 0.000706 |
| C1orf204 | -6.37414 | 0.00132 |
| RAB3D | 0.637682 | 0.038809 |
| ARMC4 | -4.44661 | 0.038677 |
| ARMC1 | -1.77016 | 0.029659 |
| ZDHHC8P1 | -14.5624 | 0.009212 |
| SPATA4 | -14.6072 | 0.000402 |
| HIST1H4C | 10.1115 | 0.004236 |
| HIST1H4A | 21.1288 | 0.024032 |
| KHSRP | 1.072798 | 0.025743 |
| SLC25A6 | 2.930405 | 0.008117 |
| PRKAG1 | 7.737134 | 0.024032 |
| PRKAG2 | -2.61087 | 0.021192 |
| GK | -0.54438 | 0.006487 |
| CLEC2B | -7.07302 | 0.008556 |
| CLEC2D | -4.06987 | 0.020951 |
| CTBP1 | 2.076007 | 0.026597 |
| C10orf26 | 3.604916 | 0.003814 |
| LOC338758 | -6.67241 | 0.008117 |
| GTF3C5 | 2.734931 | 0.002146 |
| CD99L2 | 2.336942 | 0.035142 |
| WDR26 | -3.31201 | 0.045948 |
| SPRR2E | 14.02327 | 0.004872 |
| ATAD5 | -2.19866 | 0.009544 |
| C22orf28 | 12.09987 | 0.000706 |
| FRAT2 | 1.365059 | 0.001217 |
| C22orf27 | 1.340195 | 0.018483 |
| SHROOM2 | -8.37483 | 0.037411 |
| VARS | 5.651562 | 0.001114 |
| PELI3 | 4.61922 | 0.026476 |
| AIG1 | 3.487622 | 0.037911 |
| NAPB | -2.8327 | 0.001939 |
| DBNDD2 | 3.870929 | 0.026228 |
| MYEOV | 0.359811 | 0.002977 |
| TIAM1 | -1.87615 | 0.032644 |
| FAM20B | -2.7832 | 0.011305 |
| YAF2 | -2.50981 | 0.015374 |
| SMEK1 | -3.65856 | 0.036283 |
| MAGEA2 | 17.96316 | 0.030906 |
| MAGEA1 | 19.12995 | 0.005621 |
| MAGEA6 | 12.24419 | 0.042817 |
| MAGEA4 | 20.03657 | 0.002665 |
| ZCCHC10 | -1.19704 | 0.018366 |
| RRAS | 2.550606 | 0.030665 |
| HOMER1 | -10.097 | 0.00573 |
| UCN2 | -7.77896 | 0.010752 |
| SLC40A1 | -2.90504 | 0.009212 |
| RPL29 | 3.799911 | 0.035392 |
| NIPAL2 | -8.78874 | 0.027455 |
| THAP8 | 0.512017 | 0.045814 |
| YRDC | -4.063 | 0.036032 |
| IL1F9 | 18.63539 | 0.003499 |
| EWSR1 | 4.400956 | 0.000503 |
| EFNB2 | -9.61919 | 0.00413 |
| NAP1L3 | -5.11869 | 0.021895 |
| FAM58B | 8.739224 | 0.00132 |
| DFNB31 | -10.4539 | 0.018829 |
| ASB3 | -3.87511 | 0.022717 |
| ASB6 | 3.775084 | 0.000402 |
| ASB4 | -2.55538 | 0.020829 |
| C2orf50 | -5.5155 | 0.007682 |
| C2orf55 | 0.769626 | 0.01457 |
| SLC16A13 | -5.4804 | 0.022717 |
| TMEM161B | -11.4253 | 0.000301 |
| DBI | 4.931105 | 0.005621 |
| WNK1 | 3.147715 | 0.041006 |
| TMEM47 | -8.43108 | 0.016636 |
| TMEM42 | 5.861109 | 0.00573 |
| PINK1 | 1.716651 | 0.010314 |
| MXD4 | 0.021247 | 0.004448 |
| CAMTA1 | -6.94651 | 0.011305 |
| POP4 | 5.894918 | 0.005301 |
| LRRC37B2 | -1.67045 | 0.021309 |
| PPP5C | 2.888997 | 0.003918 |
| BTD | 5.549824 | 0.026476 |
| AREG | -6.64864 | 0.004555 |
| PRPF40A | -1.46575 | 0.00132 |
| ENKUR | -1.29397 | 0.008117 |
| PRELP | 1.903685 | 0.019424 |
| LOC653653 | -0.95432 | 0.018136 |
| SEPHS1 | -4.32349 | 0.008447 |
| MOBKL3 | -0.51191 | 0.017212 |
| ISY1 | 1.51049 | 0.002872 |
| F2RL2 | -6.81439 | 0.039436 |
| F2RL1 | -13.2916 | 0.026104 |
| XAB2 | 1.141184 | 0.023911 |
| PITX3 | 14.04303 | 0.006161 |
| MED21 | -0.75945 | 0.039836 |
| NPTN | -5.67427 | 0.000402 |
| CROCC | 0.192044 | 0.011305 |
| NBPF15 | -5.17622 | 0.005301 |
| GMPPB | 2.549627 | 0.026974 |
| PAPOLA | -2.94024 | 0.008883 |
| PAPOLB | -6.14333 | 0.001423 |
| SCGB3A2 | 17.42713 | 0.006269 |
| DCAF4 | 3.28981 | 0.043857 |
| MTBP | -1.36518 | 0.007138 |
| PSD2 | -5.89061 | 0.045294 |
| CYP51A1 | -0.17959 | 0.028676 |
| TUT1 | 0.597527 | 0.006813 |
| WDR66 | -4.10644 | 0.004448 |
| HOXD8 | -3.49369 | 0.017325 |
| C1orf228 | -1.63449 | 0.020237 |
| PRMT6 | -3.95578 | 0.024032 |
| HOXD1 | -2.33477 | 0.047132 |
| HOXD3 | -9.42341 | 0.01377 |
| RETSAT | 3.307934 | 0.046212 |
| MTPAP | -0.44575 | 0.044891 |
| ORMDL2 | 5.489873 | 0.036663 |
| ORMDL1 | -1.2481 | 0.006921 |
| KIF5B | -4.58531 | 0.006813 |
| KIF5C | -4.00741 | 0.006813 |
| MED26 | 0.481647 | 0.033383 |
| MASP2 | -0.40633 | 0.024383 |
| SIRT1 | -5.0421 | 0.004979 |
| LAIR1 | 2.447723 | 0.042306 |
| FAM55B | 7.733613 | 0.013654 |
| ACADL | -4.88432 | 0.032767 |
| USP45 | -5.7715 | 0.01053 |
| PLIN2 | -4.25873 | 0.019188 |
| PPARGC1B | 0.469769 | 0.017212 |
| MAFG | -2.04888 | 0.03489 |
| CDK11B | 0.786339 | 0.001629 |
| NCAPD2 | 3.873105 | 0.038809 |
| SCRN1 | -5.96972 | 0.011196 |
| ACAD9 | 5.352808 | 0.006161 |
| NANOS3 | 3.818688 | 0.014 |
| TPRG1L | 0.077592 | 0.008005 |
| WSB1 | -15.003 | 0.0001 |
| PPBP | 16.86017 | 0.004023 |
| SLC6A12 | -1.78984 | 0.021072 |
| VPRBP | 0.362937 | 0.010861 |
| IGSF22 | -4.40015 | 0.019071 |
| GALC | -4.09639 | 0.028181 |
| NCRNA00081 | -4.64548 | 0.036155 |
| ALDH1A1 | -6.8036 | 0.012974 |
| CCNJL | -12.6711 | 0.005087 |
| NANS | 8.102277 | 0.005514 |
| RPL24 | 3.162 | 0.042949 |
| MPND | 3.970741 | 0.009544 |
| USE1 | 1.880249 | 0.038409 |
| LOC644145 | 23.9402 | 0.012191 |
| TMED9 | 0.960374 | 0.023789 |
| FXYD1 | -10.026 | 0.035142 |
| PPTC7 | -13.3948 | 0.005194 |
| KIAA0146 | -2.36723 | 0.002457 |
| DMGDH | -7.77224 | 0.002977 |
| MAFF | -8.37799 | 0.010097 |
| SNRNP35 | 4.682936 | 0.007574 |
| NCRNA00110 | -3.00971 | 0.027455 |
| MED14 | -1.27905 | 0.039836 |
| RBP3 | -2.82718 | 0.002665 |
| TUBA8 | 6.972851 | 0.007791 |
| MGC87042 | -3.03467 | 0.044253 |
| KRAS | -2.60161 | 0.003186 |
| EEF1G | 2.389224 | 0.008337 |
| ACSL6 | -2.32831 | 0.034381 |
| EGFL6 | 3.821038 | 0.017785 |
| IL20RB | 10.09596 | 0.006487 |
| NEBL | -12.6655 | 0.01767 |
| ACSL4 | -4.56964 | 0.007357 |
| WISP2 | -4.64112 | 0.048751 |
| GPRC5A | -0.7697 | 0.019188 |
| IL22RA1 | -11.4564 | 0.004766 |
| SPSB4 | 3.511877 | 0.039436 |
| MGC72080 | 4.842115 | 0.004343 |
| DNAJC30 | 3.2098 | 0.04036 |
| ANKRD22 | -9.07252 | 0.028304 |
| TMEM66 | -1.25155 | 0.033627 |
| TMEM65 | -2.98128 | 0.007138 |
| TMEM64 | -4.74266 | 0.018136 |
| TMEM61 | -12.4689 | 0.022841 |
| TNFRSF9 | -1.97998 | 0.005301 |
| TNFRSF11A | -4.91516 | 0.045294 |
| FJX1 | -6.21405 | 0.037662 |
| PYROXD2 | 5.719144 | 0.000201 |
| RPS14 | 4.194554 | 0.040111 |
| ZNF620 | -1.70189 | 0.034761 |
| MEIG1 | -4.21184 | 0.004236 |
| ZNF483 | -6.77204 | 0.003499 |
| JUNB | -2.78502 | 0.006051 |
| PRPF19 | 6.960954 | 0.000706 |
| TRAF2 | 1.56561 | 0.013314 |
| TRAF4 | -13.4254 | 0.011639 |
| HOOK2 | 1.581171 | 0.005621 |
| TMPRSS4 | 10.65979 | 0.001629 |
| TSC2 | 4.205665 | 0.017554 |
| KCNE1L | 1.148829 | 0.040752 |
| TGIF2 | -1.87419 | 0.04476 |
| EMG1 | 6.365384 | 0.003709 |
| TRIM16L | 15.89374 | 0.0001 |
| LASP1 | 6.133085 | 0.036913 |
| COMMD8 | -1.30967 | 0.039571 |
| COMMD1 | 5.703236 | 0.046874 |
| METTL12 | 0.391437 | 0.030037 |
| METTL13 | 9.960241 | 0.0001 |
| METTL10 | -1.02806 | 0.015488 |
| NPR3 | -0.70474 | 0.043209 |
| ATXN7L2 | -5.6995 | 0.023673 |
| CAMK2B | -7.08769 | 0.03894 |
| JAK2 | -6.1942 | 0.013088 |
| RPL19 | 5.579737 | 0.023193 |
| C6orf154 | -5.74443 | 0.013887 |
| SLC30A9 | -4.6101 | 0.013887 |
| ENOX1 | -1.25682 | 0.008117 |
| TCP11L1 | -12.1429 | 0.000201 |
| WDR46 | 3.71421 | 0.001012 |
| GRM5 | 7.511797 | 0.004661 |
| GRM7 | -3.29135 | 0.040111 |
| GRM2 | -0.46159 | 0.011196 |
| SLC25A26 | 0.262445 | 0.000808 |
| HOXB6 | 1.665484 | 0.01377 |
| CHUK | -5.1461 | 0.00413 |
| HOXB4 | 0.327813 | 0.045019 |
| HOXB5 | 2.939906 | 0.015833 |
| RHOU | -5.75919 | 0.023553 |
| CSGALNACT2 | -8.33558 | 0.00132 |
| POLR2E | 1.475256 | 0.043857 |
| C1QTNF9B | 0.855575 | 0.00132 |
| MAGOHB | -0.76962 | 0.035785 |
| MPP3 | -0.38062 | 0.046464 |
| CTAG2 | 19.7234 | 0.012527 |
| MORC2 | 3.333988 | 0.004872 |
| RHOC | 4.663465 | 0.026104 |
| DYRK2 | -1.79097 | 0.01457 |
| DYRK4 | -9.46806 | 0.002146 |
| ENPEP | -5.33581 | 0.03227 |
| TLL2 | -0.06604 | 0.001732 |
| DDB2 | -6.66777 | 0.021661 |
| ELL2 | -5.53088 | 0.019776 |
| LOC144438 | -0.20689 | 0.026476 |
| MCAT | 12.68088 | 0.000201 |
| APOBEC3G | -7.76812 | 0.010861 |
| APOBEC3F | -12.5189 | 0.007898 |
| TIGD1 | -4.39781 | 0.015258 |
| PPP1R15B | -5.1485 | 0.0001 |
| PRKD2 | 3.270266 | 0.011862 |
| SNX11 | 6.662202 | 0.00091 |
| FAM109B | 3.373737 | 0.040111 |
| FAM109A | 1.374884 | 0.016521 |
| LPIN2 | -2.36357 | 0.040623 |
| KLF4 | -4.26457 | 0.015946 |
| KLF3 | -4.8696 | 0.022127 |
| TMEM223 | 13.56248 | 0.003186 |
| KLF9 | -4.31855 | 0.034505 |
| TTC23L | -5.12769 | 0.028922 |
| ASCC3 | -1.85265 | 0.039571 |
| ATP13A1 | 2.874612 | 0.030787 |
| YEATS4 | -0.83274 | 0.015606 |
| ATP13A2 | 4.99103 | 0.002146 |
| RSPH4A | -4.65604 | 0.000706 |
| FAM60A | -12.3628 | 0.008117 |
| GNL3 | -4.84336 | 0.021309 |
| DHRS4 | 5.285174 | 0.00329 |
| DHRS2 | -0.12895 | 0.026974 |
| DHRS1 | 3.592048 | 0.005408 |
| DYRK1A | -0.57497 | 0.018136 |
| CCNL1 | -7.72571 | 0.0001 |
| BAZ1A | -1.61841 | 0.006377 |
| IMPA1 | -11.3755 | 0.0001 |
| IMPA2 | 1.367916 | 0.007028 |
| BAZ1B | 1.400746 | 0.019188 |
| CCRN4L | -0.73492 | 0.013542 |
| SNAI1 | -5.78031 | 0.004872 |
| HRC | 1.419713 | 0.041787 |
| PTGES2 | 0.853219 | 0.047671 |
| SLC2A3 | -3.2631 | 0.016521 |
| PLAGL2 | -2.59464 | 0.02635 |
| HKR1 | 1.368419 | 0.004448 |
| TBC1D3B | -1.7771 | 0.036409 |
| SLC1A5 | 0.243658 | 0.035785 |
| CCAR1 | -2.82857 | 0.007357 |
| MTDH | -2.12625 | 0.017325 |
| C1orf115 | 0.097057 | 0.0001 |
| ZBTB8B | -1.89036 | 0.049404 |
| CEACAM5 | 7.655629 | 0.008447 |
| IQUB | -3.65209 | 0.036663 |
| SLC27A1 | 0.657876 | 0.011196 |
| TRNAU1AP | 3.561574 | 0.032027 |
| TMEM67 | -7.5553 | 0.000808 |
| FLT1 | -6.06399 | 0.010314 |
| S1PR5 | 3.461735 | 0.011418 |
| COL25A1 | -7.72966 | 0.025241 |
| ZNF19 | 0.238066 | 0.023911 |
| MRPS18B | 4.270145 | 0.03141 |
| CLU | 2.010762 | 0.018483 |
| SLC24A3 | 4.893014 | 0.022841 |
| NTM | 2.26991 | 0.022958 |
| LOC407835 | 0.097933 | 0.037159 |
| NOP14 | 2.97396 | 0.023553 |
| PNPT1 | -4.15525 | 0.0001 |
| VPS52 | 14.57684 | 0.0001 |
| SF3B2 | 4.601133 | 0.018366 |
| SLC24A1 | -3.77226 | 0.006487 |
| MAGEF1 | 2.39855 | 0.001217 |
| SEMA6A | -9.56518 | 0.00413 |
| ELAC2 | 0.793846 | 0.000706 |
| ADCK2 | 8.890564 | 0.001114 |
| ADCK1 | 2.686529 | 0.017325 |
| DNAJC12 | -9.60108 | 0.008117 |
| DNAJC10 | -1.5527 | 0.044253 |
| LMAN1 | -5.992 | 0.00329 |
| SH3BGR | 0.335019 | 0.049921 |
| ACTG1 | -2.97747 | 0.017441 |
| MAP3K8 | -5.37898 | 0.008228 |
| MAP3K9 | -13.1966 | 0.030532 |
| MAP3K5 | -3.12048 | 0.005408 |
| FMOD | 4.136591 | 0.011639 |
| SETD7 | 7.691038 | 0.018948 |
| ITFG2 | 0.87907 | 0.006703 |
| PRSS21 | 1.256287 | 0.021309 |
| HBM | 16.65721 | 0.014456 |
| SURF6 | 3.739301 | 0.000503 |
| STX3 | -5.56138 | 0.008775 |
| RRM2B | -0.75582 | 0.005301 |
| TAPBPL | 8.026701 | 0.00329 |
| RASSF4 | 0.779204 | 0.032516 |
| MAN2A1 | -9.57272 | 0.032393 |
| SPRR2A | 18.59295 | 0.002146 |
| SPRR2C | 24.789 | 0.011196 |
| C6orf170 | -4.78478 | 0.007574 |
| PKM2 | 1.127418 | 0.037411 |
| FARS2 | 3.379247 | 0.008992 |
| STX1B | 0.247937 | 0.045294 |
| SIK1 | -9.81757 | 0.007138 |
| LSM3 | 3.715149 | 0.030037 |
| KRT80 | -13.6081 | 0.019071 |
| DUS3L | 0.900275 | 0.048751 |
| XPR1 | -5.20117 | 0.000706 |
| EDA2R | -11.3382 | 0.001423 |
| MAST3 | 2.803681 | 0.015258 |
| C5orf34 | -5.06603 | 0.007466 |
| C5orf33 | -3.2672 | 0.009323 |
| STX11 | -1.73715 | 0.015143 |
| C9orf72 | -10.1781 | 0.001217 |
| C9orf78 | 4.967783 | 0.008992 |
| CUTC | 3.902913 | 0.023789 |
| MOSPD2 | -11.8474 | 0.006269 |
| MOSPD1 | -3.67187 | 0.0001 |
| ATP11A | -9.53839 | 0.01698 |
| POLE4 | 2.896373 | 0.041006 |
| LIPH | -15.4865 | 0.01825 |
| SUOX | 4.654305 | 0.033503 |
| DPP7 | 0.931939 | 0.030408 |
| SLC7A11 | -7.60452 | 0.005087 |
| COQ10B | -2.09367 | 0.002769 |
| GLTSCR1 | 2.661019 | 0.008556 |
| GLTSCR2 | 5.956112 | 0.000808 |
| C11orf66 | -2.26153 | 0.020005 |
| SECISBP2 | -6.05079 | 0.002977 |
| PRPF18 | -3.91015 | 0.033133 |
| TRMT61A | 0.898407 | 0.015258 |
| WRAP53 | 3.294978 | 0.028426 |
| DHX16 | 2.98604 | 0.001939 |
| ZFYVE1 | 2.169869 | 0.00225 |
| CCDC88B | 0.186405 | 0.005944 |
| DGCR5 | -8.02704 | 0.039436 |
| DGCR6 | 8.919698 | 0.038677 |
| GOT2 | -4.78188 | 0.046874 |
| RNASE1 | -2.18685 | 0.035785 |
| CCDC86 | 2.578139 | 0.045019 |
| C18orf16 | -2.53815 | 0.031029 |
| TRAM1 | -3.34331 | 0.005408 |
| MRPL42 | -3.9859 | 0.009433 |
| MRPL40 | 2.169717 | 0.045431 |
| MTRF1 | -0.88302 | 0.008228 |
| APH1B | -3.88412 | 0.008337 |
| GCH1 | -1.09616 | 0.018366 |
| SDC4 | -13.3341 | 0.023193 |
| ELOVL1 | 4.063762 | 0.027578 |
| SNF8 | 3.468361 | 0.021192 |
| ZCCHC8 | -2.83172 | 0.043591 |
| TLN1 | 2.995156 | 0.042817 |
| NAA25 | -2.63452 | 0.040623 |
| RPL13A | 2.890824 | 0.038677 |
| RILP | 3.00609 | 0.031786 |
| ZNF691 | 0.291014 | 0.017441 |
| DUSP8 | -5.09828 | 0.00573 |
| DUSP5 | -10.8386 | 0.000402 |
| DUSP1 | -6.89388 | 0.019071 |
| DUSP2 | -6.0367 | 0.004661 |
| NKD2 | -9.02005 | 0.032895 |
| TAB3 | -6.97492 | 0.01377 |
| TBC1D16 | 2.371329 | 0.018948 |
| RPL34 | -2.03691 | 0.037536 |
| FBL | 1.862181 | 0.01767 |
| NUMA1 | 0.951405 | 0.035265 |
| HSPA1A | 11.35284 | 0.00091 |
| FAM83C | 11.79737 | 0.011862 |
| C8orf4 | -6.89456 | 0.00091 |
| VKORC1L1 | 5.420969 | 0.039436 |
| DGKK | 21.7105 | 0.03489 |
| DGKE | -9.17577 | 0.004448 |
| TMEM106C | -2.36366 | 0.00413 |
| CNP | 0.406809 | 0.029532 |
| METT5D1 | -3.72631 | 0.012304 |
| SRP72 | -1.5575 | 0.029042 |
| SUV39H1 | 6.201767 | 0.001114 |
| NPM1 | -3.02776 | 0.021661 |
| HSPA13 | -4.9446 | 0.001939 |
| PEMT | 7.679197 | 0.018601 |
| HSPA14 | -2.4317 | 0.010314 |
| RHCG | 9.624058 | 0.000301 |
| RUVBL2 | 7.98781 | 0.000301 |
| C1orf93 | 4.293377 | 0.001732 |
| DGCR14 | 1.304607 | 0.009875 |
| PTPN18 | 1.588082 | 0.009433 |
| SCFD2 | 1.62375 | 0.009323 |
| MEN1 | 4.559198 | 0.008883 |
| DDX54 | 1.488369 | 0.003709 |
| CTAGE5 | -1.45204 | 0.037411 |
| TMCO4 | 3.134038 | 0.025987 |
| CNDP2 | 4.804759 | 0.011196 |
| CXorf61 | 18.62768 | 0.009212 |
| LRP5 | 0.55894 | 0.042949 |
| GADD45GIP1 | 0.450915 | 0.041916 |
| NR2C2AP | 5.538726 | 0.002977 |
| PAICS | -1.81087 | 0.02415 |
| UBXN2B | -0.16761 | 0.032393 |
| KIAA0319 | 7.638913 | 0.025609 |
| DYNC2LI1 | -9.84101 | 0.00413 |
| ZNF212 | 1.289554 | 0.003081 |
| ZNF226 | -2.5503 | 0.020353 |
| ALDH8A1 | -0.44683 | 0.010206 |
| BNIP2 | -11.8795 | 0.000402 |
| KCTD15 | -6.45399 | 0.035015 |
| PDE6B | -8.46738 | 0.010752 |
| AKAP12 | -5.59744 | 0.024994 |
| AKAP11 | -7.39322 | 0.034256 |
| COL2A1 | -9.17012 | 0.047132 |
| MGC16121 | -0.06966 | 0.042172 |
| DNAH2 | -9.32316 | 0.00091 |
| C17orf88 | 4.16031 | 0.047532 |
| MAP2K1 | 12.2088 | 0.005621 |
| LOC100302640 | -11.0802 | 0.031786 |
| RCVRN | 3.458911 | 0.009212 |
| KIAA0649 | 0.300454 | 0.012527 |
| WDR81 | 0.581863 | 0.028054 |
| NLRC5 | -7.08002 | 0.010971 |
| WDR88 | -3.23655 | 0.04556 |
| AARSD1 | 2.743755 | 0.006813 |
| ESYT2 | -4.71991 | 0.047942 |
| CXCR7 | -3.01791 | 0.025484 |
| CXCR4 | 0.618326 | 0.047815 |
| RAP1GAP | -12.5861 | 0.040232 |
| EXOC3 | 2.390151 | 0.001835 |
| EXOC4 | 3.507864 | 0.017554 |
| UBE2Q2 | -1.58766 | 0.01175 |
| EXOC8 | -3.99542 | 0.005944 |
| SERTAD3 | -3.72343 | 0.002042 |
| ANK2 | -5.88117 | 0.030164 |
| MGAT4A | -4.14697 | 0.018016 |
| C9orf91 | 0.960846 | 0.018483 |
| ADAMTS1 | -6.61131 | 0.03894 |
| ARHGEF9 | -2.33313 | 0.012304 |
| ARHGEF2 | 3.103275 | 0.035015 |
| RPL23AP53 | -7.16336 | 0.027932 |
| CDH3 | -13.4265 | 0.034761 |
| LDB3 | -2.16041 | 0.030408 |
| CLCF1 | -2.04492 | 0.00413 |
| KAZALD1 | 2.235905 | 0.027096 |
| PALM | 1.054363 | 0.046874 |
| SPRED1 | -8.05957 | 0.00225 |
| PLEKHA5 | -6.91134 | 0.01264 |
| ATP5G3 | 6.423247 | 0.019071 |
| PLEKHA2 | -6.26632 | 0.014456 |
| PLEKHA3 | -6.94096 | 0.001217 |
| WDR34 | 1.693092 | 0.038809 |
| NETO2 | -7.69499 | 0.021778 |
| TRIB1 | -7.61926 | 0.0001 |
| TRIB2 | -3.9002 | 0.028676 |
| TRIB3 | -5.27815 | 0.030284 |
| DHX30 | 0.03344 | 0.047532 |
| DHX32 | -1.13883 | 0.025361 |
| DHX37 | 0.070075 | 0.018483 |
| ZNF765 | -3.25712 | 0.000808 |
| ECSIT | 5.424777 | 0.004555 |
| FAM185A | 0.681486 | 0.014801 |
| YBX1 | 6.257226 | 0.000402 |
| WDYHV1 | -0.21237 | 0.020829 |
| SMAD5 | -4.11913 | 0.019776 |
| RPL41 | 4.285293 | 0.030532 |
| MYH2 | 6.670322 | 0.035641 |
| THBS2 | -8.87579 | 0.029532 |
| MYH9 | 0.865807 | 0.012863 |
| DPH3B | -5.3409 | 0.043984 |
| VGLL4 | -8.4916 | 0.018601 |
| RLN1 | -3.33532 | 0.02635 |
| FAM149B1 | -2.76284 | 0.031285 |
| CNNM3 | -4.31618 | 0.026974 |
| CNNM4 | -12.6789 | 0.009987 |
| PIP5K1A | -2.97128 | 0.0001 |
| KEAP1 | 2.908196 | 0.000503 |
| TRIP6 | 2.645347 | 0.01208 |
| PHF15 | -4.96077 | 0.024994 |
| PHF19 | 0.701391 | 0.025241 |
| ZNF711 | -10.0798 | 0.011196 |
| CCNB1IP1 | -6.11776 | 0.008556 |
| TIA1 | -1.33906 | 0.007574 |
| UBN1 | 5.744932 | 0.029042 |
| RALGDS | -5.30573 | 0.006269 |
| LOC649330 | 3.215158 | 0.001526 |
| ATAD3A | 0.714486 | 0.005837 |
| NBR2 | -9.0406 | 0.007898 |
| HCG26 | 0.888158 | 0.033259 |
| CASC4 | -3.875 | 0.045294 |
| C20orf106 | -3.33633 | 0.031029 |
| NUDT16 | 0.302885 | 0.002977 |
| NDUFB7 | 1.980152 | 0.045431 |
| NDUFB3 | 2.640504 | 0.023077 |
| NDUFB1 | 2.569649 | 0.020951 |
| POM121L1P | -1.18106 | 0.038035 |
| GPR35 | -5.16327 | 0.041916 |
| TBC1D20 | -3.57698 | 0.026974 |
| AQP3 | 3.385888 | 0.025743 |
| NOP2 | 0.911597 | 0.002769 |
| PHB | 12.24202 | 0.000503 |
| GPR150 | 13.91282 | 0.003709 |
| ZNF516 | -2.56298 | 0.031029 |
| GSC | 0.956791 | 0.000402 |
| PGGT1B | -3.63725 | 0.023433 |
| IFIT1B | 9.154092 | 0.038543 |
| STX2 | -2.69565 | 0.030164 |
| CC2D1A | 0.623389 | 0.032767 |
| 7-Sep | -1.72582 | 0.012974 |
| BDNF | -3.57734 | 0.012863 |
| LOC100131551 | -0.20554 | 0.011639 |
| RBM39 | -0.65358 | 0.021192 |
| RBM38 | -6.64209 | 0.035641 |
| UGT1A7 | 21.01456 | 0.000706 |
| TP53INP1 | -0.42094 | 0.028181 |
| ALDH4A1 | 1.508421 | 0.0226 |
| LOC729375 | -10.5255 | 0.008556 |
| ATP8B2 | -7.60748 | 0.005944 |
| ING1 | -0.64755 | 0.01264 |
| FHL2 | -9.32861 | 0.007138 |
| FHL3 | -6.03805 | 0.034761 |
| PUS10 | -7.2084 | 0.000808 |
| UGGT2 | -2.62296 | 0.024864 |
| SULT1C4 | -7.19152 | 0.019306 |
| WWC1 | -12.5505 | 0.031659 |
| SFTPA1 | 22.47647 | 0.001217 |
| PAWR | -5.93282 | 0.01343 |
| CTDSPL2 | -5.04735 | 0.009656 |
| UGT1A10 | 22.6856 | 0.011529 |
| HMGXB3 | -5.8367 | 0.033753 |
| HMGXB4 | -2.59856 | 0.001217 |
| FLJ10661 | -7.5379 | 0.038162 |
| KIAA1712 | -4.4162 | 0.009101 |
| TBX4 | 8.473396 | 0.007138 |
| C17orf67 | -3.56525 | 0.022958 |
| C17orf69 | -5.78354 | 0.039436 |
| IPPK | -2.4126 | 0.046212 |
| C6orf130 | 0.153885 | 0.007357 |
| ADPRHL2 | 4.869621 | 0.000604 |
| SPG7 | 0.493947 | 0.03141 |
| KIAA0664 | 0.952081 | 0.009544 |
| C19orf42 | 5.121714 | 0.030665 |
| C19orf43 | 8.920264 | 0.021072 |
| CAPN1 | 1.332112 | 0.009101 |
| SPAG9 | -6.9417 | 0.013887 |
| SPAG7 | 0.808698 | 0.007682 |
| KLHL2 | -10.3582 | 0.008337 |
| FAM82A2 | 1.847109 | 0.004766 |
| ZNF696 | 2.391681 | 0.028547 |
| HIRIP3 | 0.543595 | 0.023077 |
| LOC730101 | -4.5325 | 0.025987 |
| WHAMML1 | -8.55506 | 0.009875 |
| TMEM11 | 7.605999 | 0.003081 |
| SPOCK3 | -0.88784 | 0.003918 |
| ZNF133 | 1.351858 | 0.004343 |
| PDE4DIP | 5.99713 | 0.02781 |
| CLDN12 | -10.7725 | 0.001629 |
| CLDN16 | -1.96846 | 0.000808 |
| PRDM13 | 12.40131 | 0.035392 |
| ZNF746 | 3.077864 | 0.004872 |
| MTHFD2L | -5.73907 | 0.002872 |
| NDUFA12 | 3.616088 | 0.034256 |
| LCE3A | 15.2596 | 0.022958 |
| LOC220429 | -2.5525 | 0.02635 |
| C14orf19 | 2.153429 | 0.008228 |
| VPS11 | 0.711599 | 0.000503 |
| OSMR | -4.61105 | 0.038284 |
| BIVM | -8.0853 | 0.033259 |
| DAPK1 | -1.65174 | 0.033259 |
| DPY19L4 | -9.39717 | 0.000503 |
| C21orf125 | -0.29931 | 0.018601 |
| SATL1 | 0.574742 | 0.017785 |
| SLC43A2 | 4.804253 | 0.002042 |
| HES1 | -7.38143 | 0.019071 |
| UBL3 | -6.06611 | 0.020951 |
| FKBP8 | 1.230865 | 0.024994 |
| PPME1 | 7.479749 | 0.021895 |
| IFT122 | 9.465338 | 0.012527 |
| FKBPL | 5.166209 | 0.006377 |
| MED11 | 5.27663 | 0.000503 |
| PRRC1 | -3.10805 | 0.022841 |
| ZNF460 | -2.40106 | 0.008447 |
| C12orf32 | -7.84537 | 0.028547 |
| ARNTL | -1.51695 | 0.048612 |
| LOC158696 | 0.749745 | 0.042558 |
| RPL19P12 | 6.137791 | 0.001732 |
| TMEM22 | -7.97299 | 0.002665 |
| LOC643008 | -2.11141 | 0.034505 |
| MYBBP1A | 1.509321 | 0.01377 |
| ZNRF2 | -1.73456 | 0.004979 |
| GPX3 | -6.39804 | 0.005837 |
| EFCAB6 | -4.97994 | 0.01503 |
| ANKRA2 | -5.51349 | 0.001835 |
| EFCAB3 | 4.52175 | 0.042949 |
| PRKCSH | 1.848519 | 0.026104 |
| ADCYAP1 | -1.36131 | 0.038543 |
| UBE2J2 | 6.634022 | 0.016521 |
| LOC100128573 | -1.71696 | 0.041653 |
| BRIX1 | -0.10652 | 0.015606 |
| HFM1 | -2.99997 | 0.0179 |
| ICA1L | -9.85438 | 0.007138 |
| RBM19 | 0.103255 | 0.048751 |
| RXRB | 0.671327 | 0.016405 |
| KCTD11 | 2.770094 | 0.001423 |
| ALDH9A1 | 7.931133 | 0.016636 |
| ZFYVE21 | 0.376196 | 0.041653 |
| ABCA6 | -11.4249 | 0.003709 |
| ABCA1 | -5.17006 | 0.004979 |
| TUBGCP2 | 3.971897 | 0.007138 |
| CIZ1 | 0.473228 | 0.031285 |
| PSMD1 | 7.782112 | 0.020122 |
| PSMD2 | 4.222982 | 0.004979 |
| ZNF12 | -0.6576 | 0.03894 |
| PLEKHG4B | -16.9047 | 0.018136 |
| BAT4 | 2.576438 | 0.013542 |
| BAT3 | 2.518608 | 0.000808 |
| NDUFA10 | 2.436191 | 0.044117 |
| NAF1 | -2.98045 | 0.047815 |
| TIMP2 | -7.10356 | 0.009875 |
| HPS6 | 3.186506 | 0.002042 |
| HPS1 | 1.271577 | 0.029783 |
| HPS3 | -0.97262 | 0.003604 |
| TMEM170A | -4.62122 | 0.005087 |
| C20orf96 | -4.53949 | 0.024383 |
| DOM3Z | -5.28451 | 0.047815 |
| NFE2L2 | -2.50444 | 0.00132 |
| KLF5 | -6.23799 | 0.033753 |
| MASTL | -5.51845 | 0.008228 |
| SCAND3 | -11.3834 | 0.026228 |
| DHRS4L2 | 6.40708 | 0.008337 |
| YIPF5 | -2.85619 | 0.014456 |
| YIPF4 | -2.80139 | 0.0179 |
| CYB5R4 | -4.07181 | 0.00225 |
| TM2D1 | -1.66284 | 0.004872 |
| MYO5A | -10.7073 | 0.00091 |
| RBBP6 | -4.10665 | 0.000808 |
| BTF3L4 | -1.896 | 0.01343 |
| C19orf62 | 5.300334 | 0.019541 |
| PPP2R4 | 1.86678 | 0.019776 |
| FTSJ2 | 7.525423 | 0.002457 |
| FTSJ3 | 3.494094 | 0.000301 |
| C11orf53 | -9.04205 | 0.002354 |
| C11orf51 | 5.719995 | 0.017441 |
| NLGN4X | -5.072 | 0.015833 |
| MLLT10 | -3.99743 | 0.022127 |
| EIF4H | 3.344613 | 0.000503 |
| EIF4E | -0.94875 | 0.048086 |
| WASF1 | -5.18153 | 0.041787 |
| OBP2B | 0.890323 | 0.005621 |
| EFNB3 | -14.8173 | 0.011529 |
| NHLH1 | -2.19973 | 0.039186 |
| ITIH5 | 0.029106 | 0.034005 |
| TRAPPC10 | -6.71805 | 0.032516 |
| AURKAPS1 | -6.91332 | 0.012414 |
| USP12 | -10.5306 | 0.010752 |
| USP4 | 0.063793 | 0.008775 |
| USP5 | 1.356832 | 0.039436 |
| SOX2 | 10.03559 | 0.006595 |
| RNF32 | -3.12752 | 0.016059 |
| SOX4 | -11.2625 | 0.006595 |
| TRIL | 2.384581 | 0.004766 |
| C14orf33 | -6.36724 | 0.00091 |
| INTS1 | 0.046228 | 0.041787 |
| INTS9 | 2.580145 | 0.015258 |
| CRNN | 15.58825 | 0.040111 |
| CTNNA1 | 2.69598 | 0.012304 |
| FAM181B | 2.050885 | 0.001114 |
| OR5AU1 | 11.62319 | 0.034005 |
| SLC1A1 | -5.62255 | 0.033873 |
| TGIF1 | -6.43073 | 0.023077 |
| FES | 0.499141 | 0.024994 |
| ZNF777 | 1.664235 | 0.000604 |
| RNF138P1 | -1.75698 | 0.023433 |
| MYL6 | 3.224864 | 0.024032 |
| BLCAP | 2.193248 | 0.03141 |
| CHSY1 | -0.90515 | 0.049145 |
| SFTPC | 21.93845 | 0.000402 |
| PRPSAP2 | -3.40579 | 0.020588 |
| MFI2 | 0.36713 | 0.005837 |
| LOC440354 | -5.16606 | 0.021661 |
| MAN1A1 | -4.52577 | 0.00413 |
| GORASP1 | 0.975486 | 0.006921 |
| PNMAL1 | -10.494 | 0.006161 |
| SUMF2 | 3.840384 | 0.041261 |
| SDHAF1 | 2.049685 | 0.026597 |
| SUMF1 | 1.505065 | 0.008775 |
| CRHBP | -2.17576 | 0.017785 |
| C14orf174 | -3.95306 | 0.046074 |
| C14orf176 | 0.679938 | 0.000201 |
| SURF4 | 4.071638 | 0.036535 |
| ACSF2 | 0.278127 | 0.009766 |
| PCDHGA8 | -9.23705 | 0.001423 |
| STK36 | -0.78047 | 0.015374 |
| PAPD5 | -7.1519 | 0.007357 |
| STARD4 | -1.23087 | 0.009544 |
| TMEFF1 | -4.793 | 0.02462 |
| CHRNB1 | 0.302129 | 0.025743 |
| HBEGF | -4.32133 | 0.044253 |
| RBM11 | -12.1795 | 0.003081 |
| LOC150622 | -0.15145 | 0.023077 |
| HTATSF1 | 4.230977 | 0.008447 |
| PODNL1 | -13.5778 | 0.011418 |
| GSTT2 | 8.401842 | 0.043084 |
| SLITRK5 | -7.21943 | 0.044117 |
| PDE4A | 2.789629 | 0.017212 |
| PDE4B | -6.80304 | 0.019424 |
| PDE4D | -1.3693 | 0.048219 |
| PEG3 | -4.66901 | 0.037035 |
| JAKMIP3 | -8.20685 | 0.004979 |
| MRPS25 | 2.712621 | 0.037411 |
| SLITRK3 | -1.03336 | 0.011305 |
| TSC22D1 | -7.18433 | 0.03141 |
| TPRG1 | 4.253732 | 0.046874 |
| TSC22D2 | -0.7081 | 0.03463 |
| TMEM231 | -11.0291 | 0.009544 |
| PHF6 | -5.17071 | 0.001939 |
| PPP2R5E | -3.73301 | 0.033133 |
| SMARCAL1 | 4.727964 | 0.041137 |
| PDSS1 | -4.58959 | 0.026848 |
| SLC2A4RG | 0.801037 | 0.045431 |
| ARC | -5.29468 | 0.013887 |
| ABCC9 | -3.23037 | 0.01503 |
| ABCC3 | -7.14203 | 0.039703 |
| DNAH14 | -6.23212 | 0.007247 |
| PPP2R5D | 3.590993 | 0.003499 |
| ZNF76 | 0.823262 | 0.048751 |
| RPL22L1 | -3.50483 | 0.022009 |
| GOLGA7 | -4.77293 | 0.006269 |
| EP400NL | -0.49457 | 0.01208 |
| TP53I11 | 1.99162 | 0.021895 |
| KIAA1715 | -5.6813 | 0.020122 |
| T | 12.65328 | 0.026476 |
| MEGF10 | -3.6963 | 0.038543 |
| KDM5D | 18.9438 | 0.018016 |
| PDGFA | -9.24415 | 0.01825 |
| UBE2L3 | 4.553784 | 0.021545 |
| SLC35E3 | -0.16235 | 0.020951 |
| ZNF630 | -6.03597 | 0.009766 |
| LOC440925 | 2.907535 | 0.015488 |
| STK25 | 0.371775 | 0.009323 |
| C9orf103 | 3.259953 | 0.025867 |
| AGXT2L2 | 3.595059 | 0.033873 |
| STRN4 | 1.159204 | 0.046874 |
| PPAPDC2 | -4.20004 | 0.028922 |
| CNIH | -0.30677 | 0.023673 |
| STRN3 | -7.08352 | 0.013088 |
| ETV4 | -8.50787 | 0.04727 |
| PCNP | -0.55864 | 0.016521 |
| BTBD1 | -0.61773 | 0.04556 |
| BTBD3 | -4.94283 | 0.016059 |
| IKZF4 | -2.17345 | 0.012527 |
| IKZF2 | -0.83176 | 0.043724 |
| TNFRSF10D | -7.84179 | 0.032767 |
| IL4I1 | 2.727298 | 0.048612 |
| SPATS2 | -3.56591 | 0.036409 |
| SLC13A3 | -3.73683 | 0.004555 |
| STON1 | -7.94681 | 0.00225 |
| TCEAL1 | -0.49303 | 0.007357 |
| RGS16 | -3.42674 | 0.000503 |
| FAAH | 1.333115 | 0.047671 |
| ZNF219 | 2.3447 | 0.0001 |
| PHF20L1 | -4.90408 | 0.001423 |
| TIMM22 | 5.115137 | 0.000706 |
| SMN2 | -1.7169 | 0.025743 |
| PRPS2 | -0.15852 | 0.007028 |
| CDR1 | -1.44072 | 0.030906 |
| BRPF3 | -3.2673 | 0.026228 |
| TTLL13 | -2.14059 | 0.043724 |
| GMPR2 | 5.665101 | 0.014913 |
| LUZP6 | -1.97816 | 0.034761 |
| CFL2 | -3.71143 | 0.012191 |
| C8orf83 | -0.04465 | 0.000201 |
| PLEKHG4 | -4.86276 | 0.024864 |
| SLC7A14 | -2.89758 | 0.043857 |
| BCL6B | -4.52888 | 0.035785 |
