# Supplementary material for: Identification of breast cancer recurrence risk factors based on functional pathways in tumor and normal tissues
Source: Oncotarget. 2016 Aug 23;8(13):20679–94. doi: 10.18632/oncotarget.11557 (PMC5400536; doi:10.18632/oncotarget.11557)
Supplement: Supplementary file 5 [file oncotarget-08-20679-s005.docx]

Additional file 4:

| pathway | CP1 | radius1 | CP2 | radius2 | precise1 | precise2 | fuzzy |
| --- | --- | --- | --- | --- | --- | --- | --- |
| hsa00010 | 1.24, -1.70 | 3.89 | -0.05, 0.06 | 1.16 | 0.44 | 0 | 34 |
| hsa00020 | 0.00, 0.00 | 0 | 0.00, 0.00 | 0 | 0 | 0 | 0 |
| hsa00030 | 0.00, 0.00 | 0 | 0.00, 0.00 | 0 | 0 | 0 | 0 |
| hsa00040 | 1.72, 0.00 | 1.92 | 0.07, 0.00 | 0.28 | 0.38 | 0.06 | 36 |
| hsa00051 | 1.29, 0.00 | 1.78 | 0.04, 0.00 | 0.37 | 0.46 | 0 | 33 |
| hsa00052 | 1.56, 0.00 | 2.31 | 0.04, 0.00 | 0.22 | 0.46 | 0 | 33 |
| hsa00053 | 1.88, 0.00 | 2.25 | 0.11, 0.00 | 0.13 | 0.36 | 0 | 10 |
| hsa00061 | 1.24, 0.00 | 2.27 | -0.04, 0.00 | 0.36 | 0.46 | 0 | 33 |
| hsa00062 | 1.38, 0.00 | 2.07 | -0.15, 0.00 | 0.88 | 0.26 | 0.12 | 41 |
| hsa00071 | 1.84, 0.00 | 2.49 | 0.01, 0.00 | 0.01 | 0.28 | 0 | 9 |
| hsa00072 | 0.00, 0.00 | 0 | 0.00, 0.00 | 0 | 0 | 0 | 0 |
| hsa00100 | 0.00, 0.00 | 0 | 0.00, 0.00 | 0 | 0 | 0 | 0 |
| hsa00120 | 0.00, -1.62 | 1.93 | 0.00, -0.01 | 0.51 | 0.38 | 0 | 37 |
| hsa00130 | 0.00, 0.00 | 0 | 0.00, 0.00 | 0 | 0 | 0 | 0 |
| hsa00140 | 1.88, 0.00 | 2.25 | 0.11, 0.00 | 0.13 | 0.36 | 0 | 10 |
| hsa00190 | 1.37, 0.00 | 1.99 | -0.04, 0.00 | 0.5 | 0.46 | 0 | 35 |
| hsa00230 | 1.76, 0.00 | 2.05 | -0.02, 0.00 | 0.36 | 0.49 | 0.06 | 33 |
| hsa00232 | 0.00, 0.00 | 0 | 0.00, 0.00 | 0 | 0 | 0 | 0 |
| hsa00240 | 1.69, 0.00 | 1.95 | -0.03, 0.00 | 0.71 | 0.23 | 0.12 | 40 |
| hsa00250 | 0.00, 0.00 | 0 | 0.00, 0.00 | 0 | 0 | 0 | 0 |
| hsa00260 | 0.00, -0.78 | 1.85 | 0.00, 0.03 | 0.19 | 0.64 | 0 | 26 |
| hsa00270 | 1.11, -1.70 | 3.79 | -0.01, 0.06 | 1.61 | 0.38 | 0 | 36 |
| hsa00280 | 0.00, -0.70 | 2.23 | 0.00, 0.09 | 0.41 | 0.51 | 0 | 32 |
| hsa00290 | 0.00, 0.00 | 0 | 0.00, 0.00 | 0 | 0 | 0 | 0 |
| hsa00300 | 0.00, 0.00 | 0 | 0.00, 0.00 | 0 | 0 | 0 | 0 |
| hsa00310 | 1.48, 0.00 | 1.72 | -0.15, 0.00 | 1.06 | 0.28 | 0.06 | 39 |
| hsa00330 | 0.00, 0.00 | 0 | 0.00, 0.00 | 0 | 0 | 0 | 0 |
| hsa00340 | 0.79, 0.00 | 2.74 | -0.16, 0.00 | 0.41 | 0.44 | 0 | 35 |
| hsa00350 | 1.18, -0.70 | 3.18 | -0.05, 0.09 | 0.55 | 0.67 | 0 | 25 |
| hsa00360 | 0.79, 0.00 | 2.74 | -0.16, 0.00 | 0.41 | 0.44 | 0 | 35 |
| hsa00380 | 0.00, -0.70 | 2.23 | 0.00, 0.09 | 0.41 | 0.51 | 0 | 32 |
| hsa00400 | 0.00, 0.00 | 0 | 0.00, 0.00 | 0 | 0 | 0 | 0 |
| hsa00410 | 0.79, 0.00 | 2.74 | -0.16, 0.00 | 0.41 | 0.44 | 0 | 35 |
| hsa00430 | 0.00, 0.00 | 0 | 0.00, 0.00 | 0 | 0 | 0 | 0 |
| hsa00450 | 0.00, 0.00 | 0 | 0.00, 0.00 | 0 | 0 | 0 | 0 |
| hsa00460 | 0.00, 0.00 | 0 | 0.00, 0.00 | 0 | 0 | 0 | 0 |
| hsa00471 | 0.00, 0.00 | 0 | 0.00, 0.00 | 0 | 0 | 0 | 0 |
| hsa00472 | 0.00, 0.00 | 0 | 0.00, 0.00 | 0 | 0 | 0 | 0 |
| hsa00480 | 0.00, -0.79 | 2.12 | 0.00, 0.06 | 0.52 | 0.41 | 0 | 35 |
| hsa00500 | 1.96, -1.71 | 2.57 | 0.01, 0.02 | 0.81 | 0.69 | 0.38 | 19 |
| hsa00510 | 1.23, 0.00 | 2.42 | -0.07, 0.00 | 0.44 | 0.44 | 0 | 35 |
| hsa00511 | 0.00, 0.00 | 0 | 0.00, 0.00 | 0 | 0 | 0 | 0 |
| hsa00512 | 0.00, 0.00 | 0 | 0.00, 0.00 | 0 | 0 | 0 | 0 |
| hsa00514 | 1.37, 0.00 | 2.68 | 0.05, 0.00 | 0.52 | 0.33 | 0 | 38 |
| hsa00520 | 1.15, 0.00 | 2.47 | 0.03, 0.00 | 0.4 | 0.41 | 0 | 35 |
| hsa00524 | 0.00, 0.00 | 0 | 0.00, 0.00 | 0 | 0 | 0 | 0 |
| hsa00531 | 0.00, -1.71 | 1.81 | 0.00, 0.23 | 0.63 | 0.46 | 0.25 | 28 |
| hsa00532 | 0.00, -1.84 | 2.24 | 0.00, 0.12 | 0.4 | 0.59 | 0 | 28 |
| hsa00533 | 0.00, 0.00 | 0 | 0.00, 0.00 | 0 | 0 | 0 | 0 |
| hsa00534 | 0.00, 0.00 | 0 | 0.00, 0.00 | 0 | 0 | 0 | 0 |
| hsa00561 | 1.66, -1.63 | 2.91 | 0.01, -0.03 | 0.68 | 0.72 | 0 | 24 |
| hsa00562 | 0.00, -1.73 | 1.64 | 0.00, -0.00 | 0.35 | 0.62 | 0.44 | 22 |
| hsa00563 | 0.00, -1.94 | 2.2 | 0.00, 0.09 | 1.22 | 0.36 | 0.12 | 36 |
| hsa00564 | 1.76, -1.67 | 3.02 | -0.02, 0.02 | 0.87 | 0.51 | 0 | 31 |
| hsa00565 | 0.00, 0.00 | 0 | 0.00, 0.00 | 0 | 0 | 0 | 0 |
| hsa00590 | 1.47, -1.53 | 2.92 | -0.08, 0.01 | 0.94 | 0.54 | 0.06 | 31 |
| hsa00591 | 0.00, 0.00 | 0 | 0.00, 0.00 | 0 | 0 | 0 | 0 |
| hsa00592 | 0.00, 0.00 | 0 | 0.00, 0.00 | 0 | 0 | 0 | 0 |
| hsa00600 | 0.00, -1.56 | 2.01 | 0.00, 0.12 | 0.82 | 0.31 | 0.06 | 39 |
| hsa00601 | 0.00, 0.00 | 0 | 0.00, 0.00 | 0 | 0 | 0 | 0 |
| hsa00603 | 0.00, 0.00 | 0 | 0.00, 0.00 | 0 | 0 | 0 | 0 |
| hsa00604 | 0.00, -0.81 | 2.06 | 0.00, -0.01 | 0.37 | 0.41 | 0 | 35 |
| hsa00620 | 1.56, -1.70 | 4.06 | 0.04, 0.06 | 1.12 | 0.41 | 0 | 35 |
| hsa00630 | 0.00, 0.00 | 0 | 0.00, 0.00 | 0 | 0 | 0 | 0 |
| hsa00640 | 0.00, -1.70 | 3.62 | 0.00, 0.06 | 0.96 | 0.41 | 0 | 35 |
| hsa00650 | 0.00, 0.00 | 0 | 0.00, 0.00 | 0 | 0 | 0 | 0 |
| hsa00670 | 0.00, -1.32 | 1.96 | 0.00, 0.21 | 0.47 | 0.38 | 0 | 36 |
| hsa00730 | 0.00, 0.00 | 0 | 0.00, 0.00 | 0 | 0 | 0 | 0 |
| hsa00740 | 0.00, 0.00 | 0 | 0.00, 0.00 | 0 | 0 | 0 | 0 |
| hsa00750 | 0.00, -0.70 | 2.23 | 0.00, 0.09 | 0.41 | 0.51 | 0 | 32 |
| hsa00760 | 0.00, -0.70 | 2.23 | 0.00, 0.09 | 0.41 | 0.51 | 0 | 32 |
| hsa00770 | 0.00, 0.00 | 0 | 0.00, 0.00 | 0 | 0 | 0 | 0 |
| hsa00780 | 0.00, 0.00 | 0 | 0.00, 0.00 | 0 | 0 | 0 | 0 |
| hsa00785 | 0.00, 0.00 | 0 | 0.00, 0.00 | 0 | 0 | 0 | 0 |
| hsa00790 | 0.00, 0.00 | 0 | 0.00, 0.00 | 0 | 0 | 0 | 0 |
| hsa00830 | 1.79, -0.70 | 3.08 | -0.05, 0.09 | 0.77 | 0.72 | 0 | 24 |
| hsa00860 | 1.49, 0.00 | 1.85 | -0.02, 0.00 | 0.35 | 0.56 | 0 | 30 |
| hsa00900 | 1.57, 0.00 | 2.39 | -0.02, 0.00 | 0.45 | 0.36 | 0 | 37 |
| hsa00910 | 0.00, -1.34 | 2.44 | 0.00, -0.01 | 0.52 | 0.26 | 0 | 41 |
| hsa00920 | 0.00, -1.60 | 2.1 | 0.00, -0.05 | 0.49 | 0.56 | 0 | 29 |
| hsa00970 | 1.47, 0.00 | 1.93 | -0.09, 0.00 | 0.49 | 0.72 | 0.06 | 24 |
| hsa00980 | 1.50, 0.00 | 1.67 | -0.04, 0.00 | 0.31 | 0.51 | 0.06 | 30 |
| hsa00982 | 1.51, -0.70 | 3.64 | -0.02, 0.09 | 0.54 | 0.69 | 0 | 24 |
| hsa00983 | 1.88, 0.00 | 2.25 | 0.11, 0.00 | 0.13 | 0.36 | 0 | 10 |
| hsa01040 | 0.00, 0.00 | 0 | 0.00, 0.00 | 0 | 0 | 0 | 0 |
| hsa01100 | 1.43, -1.51 | 1.79 | -0.04, 0.06 | 0.42 | 0.79 | 0.75 | 10 |
| hsa01200 | 1.10, 0.00 | 2.15 | 0.02, 0.00 | 0.55 | 0.41 | 0 | 36 |
| hsa01210 | 0.00, 0.00 | 0 | 0.00, 0.00 | 0 | 0 | 0 | 0 |
| hsa01212 | 1.31, 0.00 | 1.79 | -0.09, 0.00 | 0.54 | 0.56 | 0 | 30 |
| hsa01220 | 0.00, 0.00 | 0 | 0.00, 0.00 | 0 | 0 | 0 | 0 |
| hsa01230 | 1.10, 0.00 | 2.15 | 0.02, 0.00 | 0.55 | 0.41 | 0 | 36 |
| hsa02010 | 0.00, -0.89 | 2.04 | 0.00, -0.01 | 0.23 | 0.59 | 0 | 28 |
| hsa03008 | 1.21, 0.00 | 2.03 | 0.01, 0.00 | 0.71 | 0.44 | 0 | 35 |
| hsa03010 | 1.00, 0.00 | 2.13 | -0.02, 0.00 | 0.19 | 0.56 | 0 | 29 |
| hsa03013 | 1.32, -1.47 | 2.73 | -0.04, 0.06 | 0.79 | 0.59 | 0 | 31 |
| hsa03015 | 0.00, -0.76 | 2.2 | 0.00, -0.03 | 0.46 | 0.54 | 0 | 30 |
| hsa03018 | 1.10, 0.00 | 2.15 | 0.02, 0.00 | 0.55 | 0.41 | 0 | 36 |
| hsa03020 | 0.00, 0.00 | 0 | 0.00, 0.00 | 0 | 0 | 0 | 0 |
| hsa03022 | 1.54, 0.00 | 2.01 | 0.03, 0.00 | 0.73 | 0.36 | 0.12 | 37 |
| hsa03030 | 1.05, -2.11 | 3.4 | -0.10, -0.00 | 0.7 | 0.67 | 0 | 26 |
| hsa03040 | 1.25, 0.00 | 1.9 | -0.00, 0.00 | 0.37 | 0.51 | 0 | 32 |
| hsa03050 | 1.20, -1.19 | 3.82 | -0.10, 0.05 | 0.76 | 0.62 | 0 | 27 |
| hsa03060 | 0.00, 0.00 | 0 | 0.00, 0.00 | 0 | 0 | 0 | 0 |
| hsa03320 | 0.00, -1.20 | 2.29 | 0.00, -0.02 | 1.1 | 0.26 | 0 | 43 |
| hsa03410 | 1.78, 0.00 | 1.88 | -0.07, 0.00 | 0.93 | 0.31 | 0.25 | 36 |
| hsa03420 | 1.54, -1.48 | 3.43 | 0.03, 0.13 | 1.14 | 0.56 | 0 | 30 |
| hsa03430 | 0.00, 0.00 | 0 | 0.00, 0.00 | 0 | 0 | 0 | 0 |
| hsa03440 | 0.00, 0.00 | 0 | 0.00, 0.00 | 0 | 0 | 0 | 0 |
| hsa03450 | 0.00, -1.79 | 1.89 | 0.00, 0.13 | 1.17 | 0.26 | 0.12 | 39 |
| hsa03460 | 0.00, -1.68 | 1.68 | 0.00, 0.14 | 1.04 | 0.36 | 0.19 | 33 |
| hsa04010 | 1.37, -1.49 | 1.92 | -0.05, -0.00 | 0.86 | 0.64 | 0.5 | 19 |
| hsa04012 | 1.06, -1.09 | 3.72 | 0.03, 0.08 | 0.74 | 0.51 | 0 | 31 |
| hsa04014 | 0.88, -1.25 | 2.3 | 0.02, 0.06 | 0.4 | 0.69 | 0 | 24 |
| hsa04020 | 0.85, -1.22 | 3.01 | -0.01, 0.05 | 0.84 | 0.49 | 0 | 32 |
| hsa04060 | 1.47, -1.59 | 2.6 | -0.01, -0.04 | 0.54 | 0.59 | 0 | 29 |
| hsa04062 | 1.38, -1.47 | 3.72 | 0.00, 0.04 | 0.91 | 0.46 | 0 | 33 |
| hsa04064 | 0.00, -1.63 | 1.47 | 0.00, 0.01 | 0.75 | 0.54 | 0.69 | 18 |
| hsa04066 | 1.08, -1.23 | 2.27 | 0.03, -0.05 | 0.63 | 0.64 | 0 | 26 |
| hsa04070 | 0.00, -1.75 | 2.07 | 0.00, -0.01 | 0.49 | 0.49 | 0.12 | 29 |
| hsa04080 | 0.85, -1.13 | 2.98 | -0.01, 0.01 | 0.46 | 0.59 | 0 | 28 |
| hsa04110 | 1.18, -1.82 | 3.15 | -0.13, 0.01 | 0.84 | 0.69 | 0 | 26 |
| hsa04114 | 1.06, 0.00 | 2.4 | 0.03, 0.00 | 0.32 | 0.44 | 0 | 34 |
| hsa04115 | 0.00, -1.60 | 2.1 | 0.00, -0.05 | 0.49 | 0.56 | 0 | 29 |
| hsa04120 | 1.27, -1.74 | 3.2 | -0.06, -0.08 | 1.05 | 0.38 | 0 | 37 |
| hsa04122 | 0.00, 0.00 | 0 | 0.00, 0.00 | 0 | 0 | 0 | 0 |
| hsa04130 | 2.02, -1.25 | 3.68 | -0.07, -0.04 | 2.23 | 0.33 | 0 | 39 |
| hsa04140 | 0.00, 0.00 | 0 | 0.00, 0.00 | 0 | 0 | 0 | 0 |
| hsa04141 | 1.40, -1.54 | 2.75 | -0.07, 0.00 | 0.66 | 0.59 | 0 | 30 |
| hsa04142 | 1.09, -1.66 | 3.26 | -0.03, 0.13 | 0.69 | 0.67 | 0 | 26 |
| hsa04144 | 1.66, -1.38 | 2.98 | -0.06, 0.04 | 0.9 | 0.49 | 0.06 | 33 |
| hsa04145 | 1.42, -0.88 | 3.7 | 0.01, -0.01 | 0.47 | 0.72 | 0 | 23 |
| hsa04146 | 1.62, 0.00 | 2.3 | -0.07, 0.00 | 1.06 | 0.41 | 0 | 36 |
| hsa04150 | 0.00, -1.20 | 2.29 | 0.00, -0.02 | 1.1 | 0.26 | 0 | 43 |
| hsa04151 | 1.17, -1.34 | 2.02 | 0.01, -0.03 | 0.69 | 0.62 | 0.06 | 28 |
| hsa04210 | 0.00, -1.55 | 1.44 | 0.00, -0.01 | 0.62 | 0.59 | 0.69 | 17 |
| hsa04260 | 1.37, 0.00 | 1.99 | -0.04, 0.00 | 0.5 | 0.46 | 0 | 35 |
| hsa04270 | 1.31, -1.45 | 2.83 | 0.00, 0.00 | 0.63 | 0.41 | 0 | 36 |
| hsa04310 | 1.19, -1.46 | 4.02 | -0.12, -0.00 | 0.88 | 0.49 | 0 | 33 |
| hsa04320 | 1.06, -1.71 | 3.91 | 0.03, -0.03 | 0.71 | 0.51 | 0 | 31 |
| hsa04330 | 0.00, 0.00 | 0 | 0.00, 0.00 | 0 | 0 | 0 | 0 |
| hsa04340 | 0.00, 0.00 | 0 | 0.00, 0.00 | 0 | 0 | 0 | 0 |
| hsa04350 | 0.00, -1.81 | 1.88 | 0.00, 0.01 | 0.49 | 0.64 | 0.12 | 21 |
| hsa04360 | 0.00, -1.32 | 1.34 | 0.00, 0.03 | 0.21 | 0.56 | 0.25 | 23 |
| hsa04370 | 1.06, -2.26 | 3.52 | 0.03, -0.04 | 0.72 | 0.64 | 0 | 26 |
| hsa04380 | 1.06, -1.54 | 3.13 | 0.03, 0.07 | 0.85 | 0.62 | 0 | 27 |
| hsa04390 | 1.36, -1.74 | 4.21 | 0.04, 0.05 | 0.77 | 0.59 | 0 | 28 |
| hsa04510 | 1.17, -1.20 | 3.86 | 0.01, -0.02 | 1.1 | 0.51 | 0 | 31 |
| hsa04512 | 1.29, 0.00 | 1.92 | -0.01, 0.00 | 0.43 | 0.38 | 0 | 38 |
| hsa04514 | 0.00, -1.27 | 1.52 | 0.00, 0.04 | 0.32 | 0.59 | 0 | 29 |
| hsa04520 | 0.00, -1.55 | 1.73 | 0.00, 0.01 | 0.72 | 0.54 | 0.25 | 25 |
| hsa04530 | 1.55, -1.58 | 2.78 | -0.03, -0.01 | 0.82 | 0.36 | 0 | 38 |
| hsa04540 | 0.98, -1.35 | 3.14 | 0.02, 0.02 | 0.75 | 0.38 | 0 | 36 |
| hsa04610 | 0.00, -1.39 | 1.72 | 0.00, 0.02 | 0.32 | 0.33 | 0.06 | 38 |
| hsa04612 | 1.61, 0.00 | 1.77 | -0.02, 0.00 | 0.39 | 0.41 | 0.25 | 31 |
| hsa04614 | 0.00, 0.00 | 0 | 0.00, 0.00 | 0 | 0 | 0 | 0 |
| hsa04620 | 1.06, -1.39 | 3.48 | 0.03, -0.01 | 0.74 | 0.62 | 0 | 27 |
| hsa04621 | 0.00, -1.27 | 1.82 | 0.00, 0.01 | 0.35 | 0.51 | 0 | 33 |
| hsa04622 | 1.61, -1.18 | 2.91 | 0.02, 0.11 | 0.99 | 0.33 | 0 | 39 |
| hsa04623 | 0.00, -1.27 | 1.82 | 0.00, 0.01 | 0.35 | 0.51 | 0 | 33 |
| hsa04630 | 1.41, -1.47 | 3.21 | -0.04, -0.01 | 0.94 | 0.46 | 0 | 33 |
| hsa04640 | 0.00, -1.67 | 1.66 | 0.00, -0.08 | 0.61 | 0.54 | 0.44 | 22 |
| hsa04650 | 1.06, 0.00 | 2.4 | 0.03, 0.00 | 0.32 | 0.44 | 0 | 34 |
| hsa04660 | 1.06, -1.31 | 3.75 | 0.03, 0.02 | 0.71 | 0.64 | 0 | 26 |
| hsa04662 | 1.06, -1.18 | 4.39 | 0.03, 0.11 | 1.02 | 0.46 | 0 | 34 |
| hsa04664 | 1.31, -1.37 | 2.98 | 0.00, -0.02 | 0.96 | 0.41 | 0 | 35 |
| hsa04666 | 1.31, -1.71 | 3.3 | 0.00, -0.01 | 0.89 | 0.36 | 0 | 39 |
| hsa04668 | 1.06, -1.69 | 3.22 | 0.03, -0.01 | 0.73 | 0.69 | 0 | 24 |
| hsa04670 | 0.00, -1.61 | 2.21 | 0.00, 0.01 | 0.71 | 0.21 | 0.06 | 43 |
| hsa04672 | 0.00, -1.36 | 2.13 | 0.00, -0.08 | 0.43 | 0.41 | 0 | 36 |
| hsa04710 | 0.00, -1.20 | 1.49 | 0.00, 0.10 | 0.33 | 0.51 | 0 | 31 |
| hsa04713 | 0.00, -1.35 | 1.69 | 0.00, 0.02 | 0.75 | 0.18 | 0.06 | 43 |
| hsa04720 | 0.96, -1.35 | 3.15 | 0.01, 0.02 | 0.76 | 0.46 | 0 | 33 |
| hsa04721 | 0.00, -1.25 | 2.2 | 0.00, -0.04 | 0.99 | 0.21 | 0 | 44 |
| hsa04722 | 1.26, -1.32 | 3.02 | -0.00, -0.02 | 0.76 | 0.62 | 0 | 27 |
| hsa04723 | 0.85, -1.81 | 2.77 | -0.01, -0.01 | 0.81 | 0.62 | 0 | 28 |
| hsa04724 | 0.85, -1.54 | 2.77 | -0.01, 0.03 | 0.46 | 0.62 | 0 | 27 |
| hsa04725 | 1.06, -1.35 | 3.56 | 0.03, 0.02 | 0.89 | 0.46 | 0 | 33 |
| hsa04726 | 1.06, -1.81 | 2.71 | 0.03, -0.01 | 0.78 | 0.54 | 0 | 31 |
| hsa04727 | 0.00, 0.00 | 0 | 0.00, 0.00 | 0 | 0 | 0 | 0 |
| hsa04728 | 0.00, -1.35 | 1.69 | 0.00, 0.02 | 0.75 | 0.18 | 0.06 | 43 |
| hsa04730 | 1.06, -1.35 | 3.56 | 0.03, 0.02 | 0.89 | 0.46 | 0 | 33 |
| hsa04740 | 0.00, 0.00 | 0 | 0.00, 0.00 | 0 | 0 | 0 | 0 |
| hsa04742 | 0.00, 0.00 | 0 | 0.00, 0.00 | 0 | 0 | 0 | 0 |
| hsa04744 | 0.00, 0.00 | 0 | 0.00, 0.00 | 0 | 0 | 0 | 0 |
| hsa04810 | 1.17, -1.53 | 2.79 | 0.01, 0.02 | 0.79 | 0.38 | 0 | 37 |
| hsa04910 | 1.55, -1.14 | 2.14 | -0.03, 0.03 | 0.84 | 0.72 | 0.25 | 19 |
| hsa04911 | 0.00, -1.35 | 1.69 | 0.00, 0.02 | 0.75 | 0.18 | 0.06 | 43 |
| hsa04912 | 1.31, -1.35 | 3.32 | 0.00, 0.02 | 0.8 | 0.41 | 0 | 35 |
| hsa04913 | 0.00, -2.26 | 2.43 | 0.00, -0.04 | 0.63 | 0.64 | 0.38 | 19 |
| hsa04914 | 1.06, -1.45 | 3.82 | 0.03, 0.02 | 0.69 | 0.46 | 0 | 33 |
| hsa04915 | 1.46, -1.35 | 2.99 | -0.01, 0.02 | 0.78 | 0.49 | 0 | 33 |
| hsa04916 | 1.06, -1.56 | 3.6 | 0.03, 0.02 | 0.6 | 0.49 | 0 | 32 |
| hsa04917 | 1.06, 0.00 | 2.4 | 0.03, 0.00 | 0.32 | 0.44 | 0 | 34 |
| hsa04918 | 0.00, -1.07 | 1.18 | 0.00, 0.04 | 0.28 | 0.54 | 0.25 | 26 |
| hsa04920 | 1.08, -1.16 | 3.63 | -0.20, 0.06 | 1.02 | 0.51 | 0 | 33 |
| hsa04930 | 1.55, -1.55 | 2.82 | -0.03, -0.02 | 0.74 | 0.33 | 0 | 39 |
| hsa04932 | 1.37, -1.52 | 2.28 | -0.04, -0.05 | 1.1 | 0.56 | 0.31 | 26 |
| hsa04940 | 0.00, -1.97 | 2.76 | 0.00, -0.07 | 1.14 | 0.56 | 0.06 | 29 |
| hsa04950 | 0.00, 0.00 | 0 | 0.00, 0.00 | 0 | 0 | 0 | 0 |
| hsa04960 | 0.00, -1.20 | 2.29 | 0.00, -0.02 | 1.1 | 0.26 | 0 | 43 |
| hsa04961 | 0.00, -1.35 | 1.69 | 0.00, 0.02 | 0.75 | 0.18 | 0.06 | 43 |
| hsa04962 | 1.17, -1.74 | 2.97 | -0.02, -0.02 | 0.63 | 0.56 | 0 | 30 |
| hsa04964 | 0.00, -1.06 | 2.19 | 0.00, 0.03 | 0.53 | 0.28 | 0 | 41 |
| hsa04966 | 0.00, 0.00 | 0 | 0.00, 0.00 | 0 | 0 | 0 | 0 |
| hsa04970 | 0.00, -1.46 | 1.58 | 0.00, 0.01 | 0.6 | 0.46 | 0.38 | 25 |
| hsa04971 | 0.00, -1.35 | 1.69 | 0.00, 0.02 | 0.75 | 0.18 | 0.06 | 43 |
| hsa04972 | 1.25, -1.21 | 2.63 | 0.00, 0.02 | 0.91 | 0.41 | 0 | 36 |
| hsa04973 | 0.00, 0.00 | 0 | 0.00, 0.00 | 0 | 0 | 0 | 0 |
| hsa04974 | 0.00, -1.34 | 1.72 | 0.00, 0.01 | 0.38 | 0.18 | 0 | 46 |
| hsa04975 | 0.00, -0.68 | 2.13 | 0.00, -0.00 | 0.47 | 0.46 | 0 | 33 |
| hsa04976 | 0.00, -1.06 | 2.19 | 0.00, 0.03 | 0.53 | 0.28 | 0 | 41 |
| hsa04977 | 0.00, 0.00 | 0 | 0.00, 0.00 | 0 | 0 | 0 | 0 |
| hsa04978 | 0.00, 0.00 | 0 | 0.00, 0.00 | 0 | 0 | 0 | 0 |
| hsa05010 | 1.37, -1.48 | 2.8 | -0.04, -0.01 | 0.65 | 0.62 | 0 | 29 |
| hsa05012 | 1.56, -1.60 | 2.63 | -0.09, -0.05 | 1.31 | 0.54 | 0.06 | 31 |
| hsa05014 | 0.00, -1.60 | 2.1 | 0.00, -0.05 | 0.49 | 0.56 | 0 | 29 |
| hsa05016 | 1.11, -1.34 | 2.4 | -0.03, 0.07 | 0.77 | 0.62 | 0 | 28 |
| hsa05020 | 1.47, -1.51 | 2.44 | -0.02, -0.03 | 0.69 | 0.72 | 0.12 | 23 |
| hsa05030 | 0.00, 0.00 | 0 | 0.00, 0.00 | 0 | 0 | 0 | 0 |
| hsa05031 | 0.00, -1.38 | 2.29 | 0.00, -0.03 | 0.72 | 0.31 | 0 | 40 |
| hsa05032 | 0.00, 0.00 | 0 | 0.00, 0.00 | 0 | 0 | 0 | 0 |
| hsa05033 | 0.00, 0.00 | 0 | 0.00, 0.00 | 0 | 0 | 0 | 0 |
| hsa05034 | 1.15, -1.36 | 2.95 | -0.05, -0.06 | 0.6 | 0.41 | 0 | 35 |
| hsa05100 | 0.00, -1.88 | 2.18 | 0.00, 0.00 | 0.78 | 0.36 | 0.12 | 36 |
| hsa05110 | 0.00, 0.00 | 0 | 0.00, 0.00 | 0 | 0 | 0 | 0 |
| hsa05120 | 0.00, -1.18 | 2.13 | 0.00, 0.11 | 0.99 | 0.21 | 0 | 43 |
| hsa05130 | 1.06, -1.75 | 2.45 | 0.00, 0.00 | 0.97 | 0.56 | 0.06 | 30 |
| hsa05131 | 0.00, -1.53 | 1.82 | 0.00, 0.06 | 1.24 | 0.21 | 0.06 | 42 |
| hsa05132 | 0.00, -1.74 | 1.42 | 0.00, -0.05 | 0.61 | 0.67 | 0.69 | 13 |
| hsa05133 | 1.82, -1.59 | 3.1 | 0.05, -0.06 | 0.66 | 0.64 | 0 | 26 |
| hsa05134 | 1.69, -1.48 | 2.55 | -0.06, -0.07 | 0.95 | 0.46 | 0.19 | 33 |
| hsa05140 | 0.00, -1.89 | 1.65 | 0.00, -0.05 | 0.63 | 0.77 | 0.56 | 11 |
| hsa05142 | 0.00, -1.34 | 1.48 | 0.00, 0.00 | 0.53 | 0.54 | 0.31 | 24 |
| hsa05143 | 1.13, -1.36 | 2.75 | 0.05, -0.03 | 0.81 | 0.62 | 0 | 27 |
| hsa05144 | 1.13, -1.36 | 3.27 | 0.05, -0.08 | 0.85 | 0.64 | 0 | 27 |
| hsa05145 | 1.61, -1.36 | 2.77 | -0.02, 0.00 | 0.89 | 0.51 | 0 | 33 |
| hsa05146 | 0.00, -1.36 | 1.46 | 0.00, -0.03 | 0.54 | 0.44 | 0.06 | 31 |
| hsa05150 | 0.00, 0.00 | 0 | 0.00, 0.00 | 0 | 0 | 0 | 0 |
| hsa05152 | 0.00, -1.49 | 1.19 | 0.00, -0.04 | 0.43 | 0.74 | 0.75 | 8 |
| hsa05160 | 0.00, -1.33 | 1.62 | 0.00, 0.03 | 0.46 | 0.56 | 0.25 | 26 |
| hsa05161 | 1.34, -1.55 | 2.12 | 0.03, -0.06 | 0.58 | 0.72 | 0.25 | 21 |
| hsa05162 | 1.50, -1.49 | 2.38 | -0.01, -0.02 | 1.02 | 0.56 | 0.31 | 26 |
| hsa05164 | 1.46, -1.59 | 1.98 | 0.02, -0.06 | 0.58 | 0.79 | 0.62 | 11 |
| hsa05166 | 1.43, -1.52 | 2.73 | 0.11, -0.04 | 0.77 | 0.64 | 0 | 26 |
| hsa05168 | 0.76, -1.38 | 3.63 | -0.03, -0.01 | 0.71 | 0.62 | 0 | 27 |
| hsa05169 | 1.53, -1.32 | 2.7 | 0.02, 0.06 | 0.92 | 0.46 | 0 | 35 |
| hsa05200 | 1.12, -1.38 | 2.28 | 0.03, 0.01 | 0.64 | 0.67 | 0.06 | 26 |
| hsa05202 | 0.00, -1.73 | 1.68 | 0.00, -0.00 | 0.52 | 0.62 | 0.44 | 20 |
| hsa05203 | 1.54, -1.80 | 2.98 | 0.07, -0.14 | 0.64 | 0.72 | 0 | 23 |
| hsa05204 | 1.50, -2.26 | 3.81 | -0.03, -0.04 | 0.64 | 0.72 | 0 | 23 |
| hsa05205 | 1.17, -1.48 | 2.5 | 0.01, -0.00 | 0.61 | 0.54 | 0 | 31 |
| hsa05206 | 1.06, -1.65 | 3.52 | 0.03, -0.02 | 0.54 | 0.54 | 0 | 30 |
| hsa05210 | 1.06, -1.30 | 3.36 | 0.03, 0.04 | 0.72 | 0.59 | 0 | 28 |
| hsa05211 | 1.06, -1.13 | 3.77 | 0.03, -0.06 | 0.71 | 0.54 | 0 | 30 |
| hsa05212 | 1.12, -1.10 | 2.34 | 0.03, 0.12 | 0.87 | 0.49 | 0 | 33 |
| hsa05213 | 1.06, -1.20 | 3.8 | 0.03, -0.02 | 1.1 | 0.51 | 0 | 31 |
| hsa05214 | 1.06, 0.00 | 2.4 | 0.03, 0.00 | 0.32 | 0.44 | 0 | 34 |
| hsa05215 | 1.06, -1.03 | 3.3 | 0.03, 0.04 | 0.76 | 0.59 | 0 | 28 |
| hsa05216 | 1.06, 0.00 | 2.4 | 0.03, 0.00 | 0.32 | 0.44 | 0 | 34 |
| hsa05217 | 0.00, -1.76 | 1.81 | 0.00, 0.01 | 0.33 | 0.26 | 0.12 | 39 |
| hsa05218 | 1.06, 0.00 | 2.4 | 0.03, 0.00 | 0.32 | 0.44 | 0 | 34 |
| hsa05219 | 1.06, 0.00 | 2.4 | 0.03, 0.00 | 0.32 | 0.44 | 0 | 34 |
| hsa05220 | 1.06, -1.18 | 4.39 | 0.03, 0.11 | 1.02 | 0.46 | 0 | 34 |
| hsa05221 | 1.06, -1.32 | 3.57 | 0.03, 0.06 | 0.7 | 0.54 | 0 | 30 |
| hsa05222 | 0.00, -1.68 | 1.62 | 0.00, 0.01 | 0.43 | 0.77 | 0.69 | 10 |
| hsa05223 | 1.06, -1.20 | 3.8 | 0.03, -0.02 | 1.1 | 0.51 | 0 | 31 |
| hsa05310 | 0.00, 0.00 | 0 | 0.00, 0.00 | 0 | 0 | 0 | 0 |
| hsa05320 | 0.00, 0.00 | 0 | 0.00, 0.00 | 0 | 0 | 0 | 0 |
| hsa05321 | 0.00, -1.56 | 1.46 | 0.00, -0.02 | 0.59 | 0.51 | 0.62 | 21 |
| hsa05322 | 1.30, -1.42 | 2.46 | 0.02, -0.01 | 0.79 | 0.56 | 0 | 29 |
| hsa05323 | 0.00, -1.67 | 1.66 | 0.00, -0.08 | 0.61 | 0.54 | 0.44 | 22 |
| hsa05330 | 0.00, 0.00 | 0 | 0.00, 0.00 | 0 | 0 | 0 | 0 |
| hsa05332 | 0.00, -1.67 | 1.66 | 0.00, -0.08 | 0.61 | 0.54 | 0.44 | 22 |
| hsa05340 | 0.00, 0.00 | 0 | 0.00, 0.00 | 0 | 0 | 0 | 0 |
| hsa05410 | 1.29, -1.36 | 3 | -0.01, -0.08 | 1.07 | 0.56 | 0 | 30 |
| hsa05412 | 1.29, -1.39 | 2.45 | -0.01, -0.02 | 0.48 | 0.46 | 0 | 34 |
| hsa05414 | 1.29, 0.00 | 1.92 | -0.01, 0.00 | 0.43 | 0.38 | 0 | 38 |
| hsa05416 | 0.00, -1.60 | 2.1 | 0.00, -0.05 | 0.49 | 0.56 | 0 | 29 |
